# Initial nitrogen enrichment conditions determines variations in nitrogen substrate utilization by heterotrophic bacterial isolates

**DOI:** 10.1186/s12866-017-0993-7

**Published:** 2017-04-04

**Authors:** Suchismita Ghosh, Paul A. Ayayee, Oscar J. Valverde-Barrantes, Christopher B. Blackwood, Todd V. Royer, Laura G. Leff

**Affiliations:** 1 Department of Biological Sciences, Kent State University, Kent, OH 44242 USA; 2 International Center for Tropical Botany (ICTB), Florida International University, Miami, FL 33199 USA; 3 School of Public and Environmental Affairs, Indiana University, Bloomington, Bloomington, IN 47405 USA

**Keywords:** Dissolved organic nitrogen, Bacterial isolates, Nitrogen cycle

## Abstract

**Background:**

The nitrogen (N) cycle consists of complex microbe-mediated transformations driven by a variety of factors, including diversity and concentrations of N compounds. In this study, we examined taxonomic diversity and N substrate utilization by heterotrophic bacteria isolated from streams under complex and simple N-enrichment conditions.

**Results:**

Diversity estimates differed among isolates from the enrichments, but no significant composition were detected. Substrate utilization and substrate range of bacterial assemblages differed within and among enrichments types, and not simply between simple and complex N-enrichments.

**Conclusions:**

N substrate use patterns differed between isolates from some complex and simple N-enrichments while others were unexpectedly similar. Taxonomic composition of isolates did not differ among enrichments and was unrelated to N use suggesting strong functional redundancy. Ultimately, our results imply that the available N pool influences physiology and selects for bacteria with various abilities that are unrelated to their taxonomic affiliation.

**Electronic supplementary material:**

The online version of this article (doi:10.1186/s12866-017-0993-7) contains supplementary material, which is available to authorized users.

## Background

Bacterial nitrogen (N) uptake and assimilation are influenced by availability and nature of dissolved organic and inorganic forms of N [1]. Simple N compounds are readily available to heterotrophic bacteria [2–4], whereas more complex N compounds require enzymatic degradation prior to uptake and assimilation [5, 6]. Heterotrophic bacterial communities use a variety of dissolved organic nitrogen (DON) compounds, including amino acids [7], nucleic acids [8], and proteins [9, 10], as carbon, N, and/or energy sources, or directly as specific compounds, such as via salvage pathways for amino acids [11]. In addition to DON, dissolved inorganic (DIN) species, such as nitrate [12] and ammonium [3], are also used to meet N requirements.

The ability of bacterial communities to utilize particular N types (simple vs. complex, labile vs. recalcitrant) depends on taxonomic composition [13], biochemical capacities, and competition with other bacteria for N [14]. Interactions under differing conditions result in varied N-utilization profiles [15, 16] among members of a bacterial community and may lead to ecological specialization [17, 18]. Ultimately, although N-utilization differs among heterotrophic bacterial communities, there is uncertainty regarding the scale at which common metabolic capabilities are shared regardless of the dominant forms of available N.

In this study, we investigated utilization of N substrates, ranging from labile to recalcitrant, by heterotrophic bacteria isolated from stream sediments under different N-enrichments (simple and complex). We sought to determine: 1) whether bacteria isolated from complex and simple N-enrichments would be taxonomically and compositionally different, and 2) if N-substrate utilization by isolated bacteria was dependent on initial N-enrichment conditions.

## Methods

### Bacterial isolation

Stream sediment samples from three streams used in prior N studies: West Branch of Mahoning River near Ravenna, OH [19], Sycamore Creek located in Morgan County, IN [20], and Sugar Creek near Shirley, IN [20] were incubated in M9 minimal media, (amended with glucose as the carbon source) with 8 different N compounds. All final N concentrations were 94 mM. These included five single-source N treatments (nitrate in the form of NaNO_3_, ammonium, urea, glycine, and tryptophan), an equimolar mixture of these compounds (ammonium + nitrate + urea + glycine + tryptophan), a bacterial protein (undefined cellular extract) and nutrient broth (complex medium; Difco BD nutrient broth [Becton, Dickinson and Company, Franklin Lakes, NJ, USA]). The bacterial protein was obtained as described in Ghosh et al. (2013) [21]. Briefly, soluble bacterial proteins were extracted from cultures of *Bacillus subtilis*, *Pseudomonas aeruginosa*, and *Staphylococcus aureus* incubated at 27 °C for 24 h and proteins were obtained using the Qproteome Bacterial Protein Prep Kit (Qiagen, MD, USA) and total DON quantified using a Shimadzu TNM-1(Shimadzu Corporation, Columbia, MD). Among the enrichments, ammonium, nitrate, and glycine were considered simple N-enrichments. Nutrient broth and the bacterial protein extract were considered complex enrichments, as were tryptophan and urea. In this study, urea was considered a complex enrichment due to low bacterial uptake compared to inorganic N species, amino acids and carbohydrates in a study of freshwater bacterial N turnover [22]. Tryptophan was considered complex due to its large molar mass, and chemical composition [23]. The defined-N-mixture (ammonium, nitrate, glycine, urea and tryptophan) was considered a simple enrichment for three reasons. First, the abundance of simple compounds relative to urea and tryptophan. Second, repression of the nitrogen assimilation control (*nac*) operon for urea uptake in the presence of ammonium and other simpler N compounds [1], as is the case in the defined-N-mix. Third, the high affinity for electrophilic substitutions in the indole ring of tryptophan renders it readily deoxidized in the presence of other compounds (including nitrate, carbon dioxide, and ammonia) leading to modifications into other compounds that could be utilized by bacterial cells [23].

Enrichments were incubated at 25**°**C for 24 h to isolate fast-growing bacteria or for 72 h to isolate slow-growing bacteria. Samples from each enrichment were used to inoculate plates of the same composition mixed with agar. Distinct colonies from respective plates were selected for isolation into pure cultures.

### 16S rRNA gene amplification and sequence analyses

Genomic DNA was extracted from bacteria isolates using the CTAB method followed by phenol: chloroform extraction and ethanol precipitation as in Moore et al. (2004) [24]. Polymerase chain reaction (PCR) was carried out with the universal primers 27F (5′-AGAGTTTGATCMTGGCTCAG-3′) and 1552R (5′-AAGGAGGTGATCCARCCGCA-3′) [25] in a PTC 200 DNA Engine Cycler (Biorad, Hercules, CA) with a thermal profile of 94 °C for 3 min and 35 cycles of 94 °C for 30 s, 58 °C for 30 s and 72 °C for 90 s followed by a final extension of 72 °C for 5 min. Each 25 μl PCR reaction mixture consisted of 2 μl of template DNA, 12.5 μl of water, 0.5 μl of both forward and reverse primers, and 12.5 μl of GoTaq Pre- Mixed Green Master Mix (Promega Corporation, Madison, WI). Amplified products were visualized on a 1% agarose via gel electrophoresis, purified and submitted for Sanger sequencing at the Advanced Genetic Technologies Center, at the University of Kentucky (Lexington, KY), using the same primer pair.

Resulting amplicon sequences were quality checked in Sequencher (Gene Codes Corporation, Ann Arbor, MI) using default settings. Sequences were classified using the Classifier tool in the Ribosomal Database Project (RDP) server [26]. Taxonomic affiliations of the isolates were determined at a cut-off threshold of 80% in RDP, and an operational taxonomic unit (OTU) table generated summarizing the taxa and abundance of isolates from each enrichment at the class level. This table was subsequently used to determine within-enrichment alpha diversity estimates (Chao1) [27] in QIIME (version 1.9.0) [28] following rarefaction. The reliance of Chao1 estimates on singletons, makes it a more robust estimate. A non-metric multidimensional scaling (NMDS) [29] analysis was performed on the Bray-Curtis distance matrix and axes used to visualize relatedness among the enrichments. Compositional difference between enrichments was assessed using the analysis of similarity (ANOSIM) multivariate test in QIIME.

### Nitrogen substrate utilization assays

Substrate utilization by bacterial isolates was assessed spectrophotometrically in 96-well microtitre plates. 12 single-source N-substrates (94 mM each) ranging from labile to recalcitrant forms were used. The labile and recalcitrant designations are based on known resistance/refraction to degradation, bioavailability, and impacts on bacterial growth. The substrates were nitrate, ammonium, urea, glycine, proline, tryptophan, bacterial protein, peptidoglycan, nucleic acid (purified DNA), algal exudate, putrescine (polyamine), and humic matter. Humic matter, algal exudates and nucleic acids were obtained as described in Ghosh et al. (2013) [21]. Briefly, algal exudates were extracted from cultures of *Chlamydomona*s, *Chlorella*, and *Synedra* (Carolina Biological Supplies, Burlington, NC) grown in artificial stream water with 20 mg/L of NaNO_3_, under constant light for 35 days. Humic matter was extracted from senescent red oak (*Quercus rubra*), witch hazel (*Hamamelis virginiana*), and corn leaves (*Zea mays*) in 0.027% NaCl and pooled. Nucleic acids were obtained following DNA extraction from cultures of *Bacillus subtilis*, *Pseudomonas aeruginosa*, and *Staphylococcus aureus* incubated at 27 °C for 24 h; extractions were performed using the Power-Soil DNA extraction kit (MoBio Laboratories, Carlsbad, CA) and nucleic acids were pooled among the three cultures. Following initial cell lysis and precipitation of bacterial cultures during DNA extraction, cell debris was collected and quantified to represent peptidoglycan. Putrescine was purchased from MP Biomedicals (MP Biomedicals, Santa Ana, CA, USA). Of N treatments, algal exudates, ammonium, nitrate, glycine, tryptophan, and urea were considered labile [21, 30] whereas, bacterial proteins, nucleic acid, and humic matter were considered recalcitrant [31, 32]. Peptidoglycan, polyamine (putrescine) and proline (Amresco Biochemicals and Life Science Research Products, Solon, OH, USA) were considered intermediate compounds. The rationale for these designation is that proline, as a N source in the presence of glucose, is suboptimal for *E. coli* growth [33], and disproportionately accumulates in particulate residues following microbial exposure, suggesting proline utilization following degradation of more bioavailable N sources [34]. In contrast, bacterial growth is positively correlated with tryptophan availability [35]. Peptidoglycan is designated an intermediate compound because the efficiency of peptidoglycan degradation by bacteria has ranges from 49% - 58% depending on whether they were from gram negative or positive bacterial sources, respectively [36]. Each of the 12 single-source N media had the same amount of nitrogen (94 mM) as the standard minimal media used in Maheswaran and Forchhammer 2003 [37] with glucose as the only carbon source.

Before beginning the assays, bacterial cultures were incubated in their respective broth media for 24–48 h depending on growth rate. After cultures reached an optical density (OD) of 0.4, they were centrifuged and washed five times with N-free minimal media and diluted 1:10 with the N-free minimal media to minimize transfer of N to the test plates. Washed cultures were subsequently transferred to plates, making up 10% of the final assay volume. Plates were incubated at room temperature for 6 days and OD determined at 600 nm every 12 h for the first 48 h, and every 24 h for the remaining 4 days. Treatments were carried out in triplicates for each isolate. Bacterial growth rates (day^−1^) were calculated from OD_600_ values recorded at the different time points.

Assessment of substrate utilization and substrate range used by isolates was carried out by dividing the growth rates (day^−1^) into ranges as: −1 for growth rates <0, 0 for rates between 0 and 29, 1 for rates between 30 and 39, 2 for rates between 40 and 99, and 3 for growth rates >100. Substrate range for each isolate was calculated by determining the mean score for each isolate across all 12 substrates. The score difference (Δ score = total isolate score – mean) for each isolate was determined and then used to categorize the substrate range of each isolate. Isolates with positive score differences were categorized as having broad substrate ranges and those with negative score differences were categorized as having narrow substrate ranges.

### Statistical analysis

One-way analysis of variance (ANOVA) was used to examine differences among enrichments based on the Chao1 estimates without transformation. This was followed by visualization of the NMDS coordinates using the generated distance matrix, after the ANOSIM multivariate test of compositional differences. Differences in patterns of N-utilization by bacteria isolates were analyzed using a mixed-model analysis with actual growth rates as the dependent variable and N-enrichment and N-substrates as independent variables. Relationship between phylogenetic distance and substrate utilization (growth rates expressed as scores as described above) was examined using regression analysis, and the relationship between categorical bacterial N-utilization profiles (broad vs. narrow substrate ranges) and taxonomic affiliations was examined using contingency analysis followed by the Pearson’s chi-square test. Statistical analyses were carried out in JMP 10 (SAS Institute Inc., Cary, NC, USA) and QIIME (version 1.9.0).

## Results

### Composition and diversity of bacterial isolates from N-enrichments

A total of 266 isolates representing 24 families were obtained (Additional file 1: Table S1). The highest number of isolates were from the nutrient broth enrichment (58), followed by tryptophan (34), ammonium (32), defined-N-mixture (31), glycine (29), nitrate (28) and urea (28), with the bacterial protein enrichment yielding the least number of isolates (26).

**Fig. 1:**
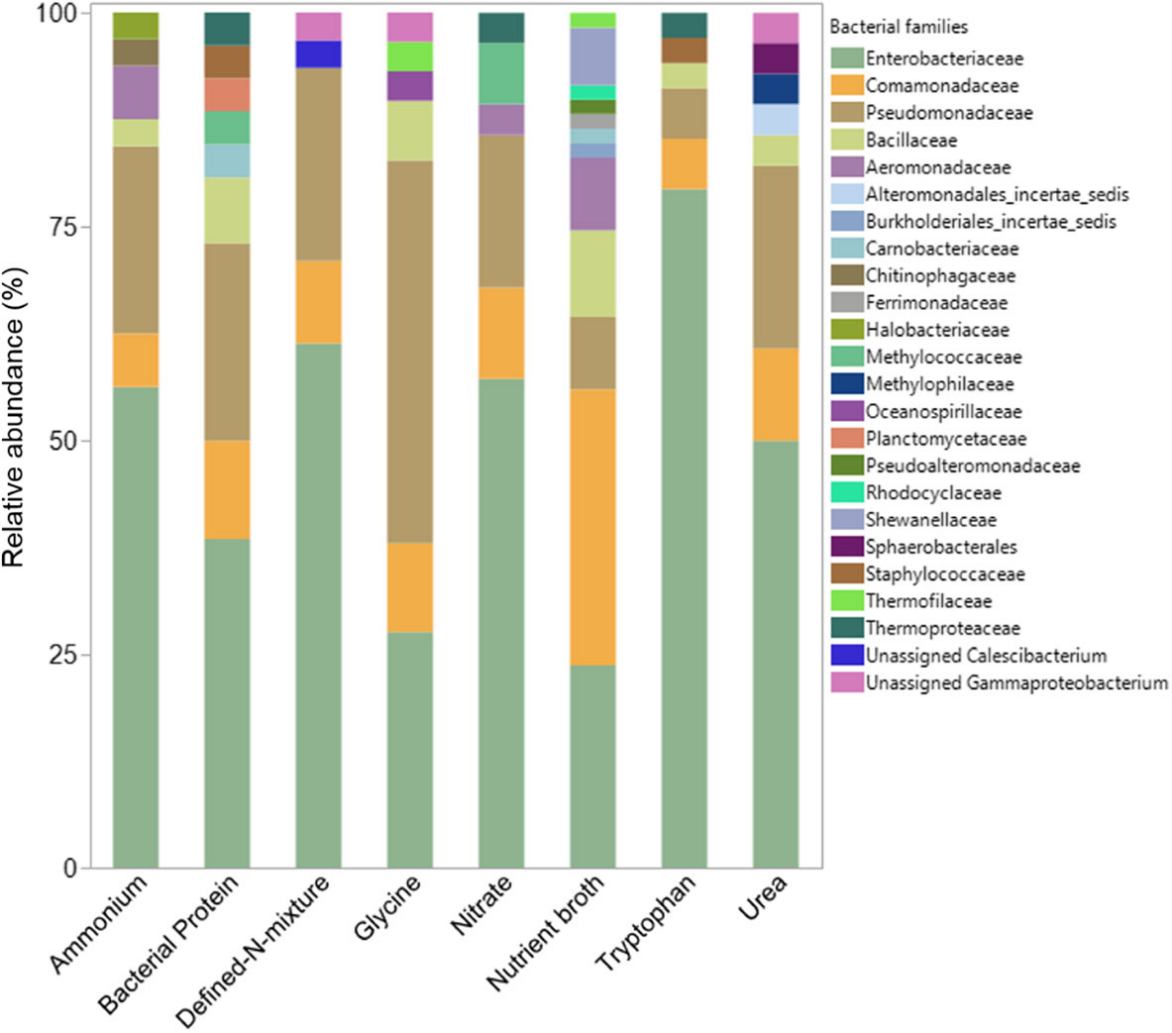
Relative abundances (%) of bacterial families of bacterial isolates from the eight initial N-enrichments

Taxonomically, four bacterial families, *Comamonadaceae*, *Enterobacteriaceae*, *Bacillaceae*, and *Pseudomonadaceae,* were most commonly isolated from complex N-enrichments (bacterial protein, nutrient broth, urea and tryptophan). In addition, 9 unique bacterial families (present in only one enrichment) were detected from these complex enrichments. Three families, *Alteromonadales incertae sedis* (relative abundance, 3.57%), *unassigned Sphaerobactereales* (3.57%), *and Methylophilaceae* (3.57%), were only detected from the urea enrichment. *Planctomycetaceae* (3.85%) was only detected from the bacterial protein enrichment and five bacterial families, *Burkholderiales incertae sedis* (1.69%), *Shewanellaceae* (6.78%), *Pseudoalteromonadaceae* (1.69%), *Ferrimonadaceae* (1.69%), and *Rhodocyclaceae* (1.69%), were only detected only from the nutrient broth enrichment (Fig. 1). Partial 16S rRNA sequence data for each isolate are provided in Additional file 2: Document 1.

Three bacterial families (*Comamonadaceae*, *Enterobacteriaceae,* and *Pseudomonadaceae*) were well represented in isolates from the simple N-enrichments (ammonium, glycine, nitrate and defined-N-mixture) and 4 unique bacterial isolates were detected. *Oceanospirillaceae* (3.45%) was only detected from the glycine enrichment. *Halobacteriaceae* (3.13%) and *Chitinophagaceae* (3.13%) were only detected from the ammonium enrichment and *Unassigned Calescibacterium (3.23%)* was only detected from the defined-N-mixture enrichments (Fig. 1).

Mean Chao1 diversity estimates at the family level differed significantly among N-enrichments (F = 2.22; df = 7, 136; *P* = 0.04), but there was no significant difference in alpha diversity between simple and complex N-enrichments when grouped together. Individually, the least diverse enrichments, tryptophan (1.83 ± 0.63, mean ± s.e.) and defined-N-mixture (1.94 ± 0.63), were significantly different from the most diverse enrichments, glycine (4.35 ± 0.63) and nutrient broth (4.24 ± 0.63). Bacterial protein (3.58 ± 0.63), urea (3.2 ± 0.63), nitrate (3.14 ± 0.63) and ammonium (2.7 ± 0.63) enrichments had comparable richness estimates.

**Fig. 2:**
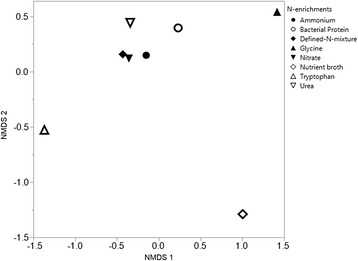
Displacement of bacterial communities within the NMDS plot (Stress <0.01). Complex N-enrichments were tryptophan (*open triangle*), nutrient broth (open diamond), urea (open inverted triangle), and bacterial protein (*open circle*). Simple N-enrichments were glycine (filled triangle), ammonium (filled circle), nitrate (filled inverted triangle), and defined-N mixture (filled diamond)

In spite of observed differences in Chao1 diversity estimates among N-enrichments and the presence of a few enrichment-specific isolates, overall community composition were very similar among N-enrichments (ANOSIM; Test statistic = −0.013, *P* = 0.55, number of permutations =1000, number of samples =24, number of groups =8). In the NMDS plot (Fig. 2, stress <0.01), the complex N-enrichments, tryptophan and nutrient broth grouped separately from each other and from the remaining two complex enrichments (urea and bacterial protein), and the simple N-enrichment glycine, was displaced from the other three simple N-enrichments, ammonium, nitrate, and defined-N mixture, which clustered closely together (Fig. 2). The observed displacements may be attributed to the presence of single unique isolates in several of the enrichments, but these were not sufficient to result in significant differences in overall community composition.

### Bacterial isolate N-utilization

**Fig. 3:**
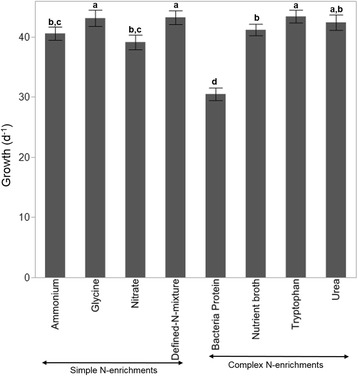
Actual growth rates averaged across all N substrates (day^−1^) (mean ± s.e.) for bacterial isolates from the eight initial N-enrichments (F _(7, 3184)_ = 36.2, *P* < 0.001). The N-enrichments were: Nitrate, Ammonium, Glycine, Tryptophan, Urea, Defined-N-mixture, Bacterial Protein, and Nutrient Broth. Different letters represent significantly different growth rates on each N-enrichment at *P* = 0.05

**Fig. 4:**
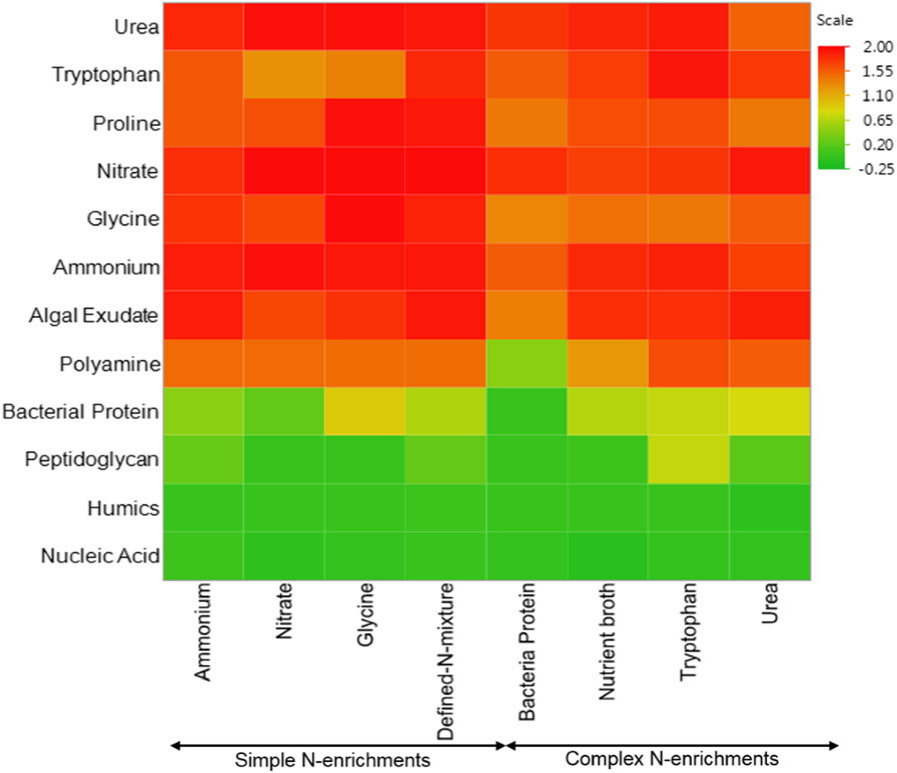
Substrate utilization by groups of isolates from the initial enrichments on the twelve substrates used in the substrate assay (F_(77, 3114)_ = 4, *P* < 0.001) depicted in a heat map. The color legend indicates the scaled scores from −0.25 to 2.00, with high and moderate substrate utilization shown as red and orange respectively, and the low and least substrate utilization shown as shades of yellow and green respectively

Substrate utilization by isolates differed significantly among the 8 initial source N-enrichments (F = 36.2; df = 7, 3184; *P* < 0.001). Overall, substrate utilization was lowest in bacteria obtained from the bacterial protein enrichment and highest in bacteria obtained from glycine, defined-N mix, and tryptophan (Fig. 3). There were significantly differences in substrate utilization by isolates among the 12 N-substrates used (F = 557.2; df = 11, 3180; *P* < 0.001), as well as significant N-enrichment by N-substrate differences in utilization by bacteria isolates (F = 3.9; df = 77, 3114; *P* < 0.001) (Fig. 4). Substrate utilizations were lowest on recalcitrant nucleic acid (6.02 ± 0.81) and humic matter substrates (11.74 ± 0.81) for bacteria from all enrichments, followed by peptidoglycan (17.9 ± 0.81) and bacterial protein (29.2 ± 0.81) substrates. On the other hand, all labile substrates, except for glycine and tryptophan were efficiently utilized by bacteria from all N-enrichments. Utilization of glycine, proline and tryptophan differed among bacteria in a N-enrichment driven manner; utilization of glycine and proline substrates were greater among bacteria from the simple enrichments, whereas utilization of tryptophan was greater among bacteria from the complex enrichments (Fig. 4). Growth rates for each of the 266 isolates are shown in Additional file 3: Table S2. The relationship between substrate range/utilization and N-enrichment was statistically significant (Pearson’s test; Chi-square = 32.5, *P* < 0.0001), demonstrating that initial enrichment influenced subsequent substrate utilization and the range of substrates used. However, there was no significant linear correlation between average phylogenetic distance and average substrate utilization (R-statistic = 0, *P* = 0.96).

**Fig. 5:**
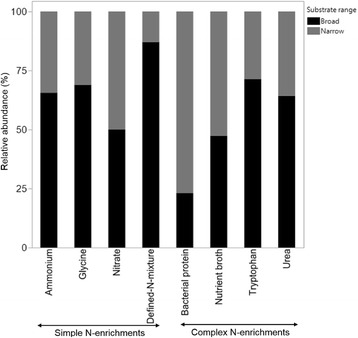
Proportion (%) of isolates with broad and narrow substrate ranges from each enrichment following the substrate utilization assay

Finally, among enrichments there were differences in the range of substrates that were effectively utilized by bacteria. Bacterial isolates from the simple defined-N-mixture and ammonium N-enrichments had comparatively broader substrate ranges, followed by isolates from the complex tryptophan and urea N-enrichments (Fig. 5) (Additional file 4: Table S3). The bacterial protein enrichment yielded isolates with the narrowest substrate range, whereas the proportions of isolates with narrow and broad substrate ranges were equivalent in the nitrate and nutrient broth enrichments.

## Discussion

Initial N-enrichments significantly impacted subsequent N substrate use. However, these differences were not related to taxonomy of the isolates. Likewise, bacteria isolated from each of the 8 initial N-enrichments did not differ in taxonomic composition in spite of differences in richness and the presence of a number of unique taxa in specific enrichments. In general, patterns of N substrate use were influenced by enrichment rather than taxonomy, suggesting there was enrichment-specific selection for organisms independent of 16S rRNA gene sequences. Thus the lack of a relationship between substrate utilization and taxonomic affiliations is most likely explained by taxon-independent capacity for N-utilization (functional redundancy) [38].

Bacterial functional traits, such as nitrogen utilization and substrate ranges are influenced by environmental factors leading to variations in metabolic capabilities and, ultimately, ecological specialization within microbial assemblages and are taxon-independent [17, 18]. Additionally, substrate utilization patterns may be a function of acclimation and physiological change rather than reflective of genotypic differences. Isolates from two complex enrichments (tryptophan and urea) and three simple enrichments (ammonium, glycine and defined-N-mixture) had similar substrate utilization profiles and greater proportions of broad substrate range isolates, suggestive perhaps of activated metabolic pathways enabling utilization of subsequent various N substrates regardless of the initial enrichments. The same explanation may be applied to the substrate utilization profiles of the defined-N-mixture enrichment, wherein a broad range of N compounds that can be utilized by bacteria is to be expected. As a result, the nitrogen-rich condition in this enrichment may have facilitated growth of metabolically versatile and broad substrate range. Utilization of other single N compounds and the production of intermediates, such as ammonium by bacteria isolates from these enrichments, may explain the breadth of N substrate use and similarities in N-profiles in the substrate assay [1].

Various operons within the bacterial nitrogen regulation system (*ntr*) enable degradation and/or uptake of diverse N sources [1, 5, 6]. Some of these operons are only activated by specific N sources leading to their rapid uptake, while others are repressed by certain N sources and only activated in their absence leading to instantaneous and primed N uptake pathways, respectively [1]. Priming may have contributed to the observed substrate ranges of isolates from complex enrichments. For example, one pathway for tryptophan use is non-oxidative degradation to ammonia, indole and pyruvate via the indole pathway [39]. The pyruvate and ammonia formed are then respectively used for respiration and amino acid biosynthesis [39]. Along these same lines, urea can be taken up by a variety of bacteria and hydrolyzed to ammonium and CO_2_ by urease; the resulting ammonia is subsequently used for biosynthesis and growth [40]. Finally, glycine is oxidatively degraded into ammonium, CO_2_ and a methylene group via the glycine cleavage system or glycine synthetase [41, 42]. Thus, among these bacterial communities, similar substrate utilization profiles may be attributed to shared/activated metabolic capacities by differently primed nitrogen utilization pathways selected by the various enrichments.

Although several bacteria families, including *Planctomycetaceae,* were obtained from the bacterial protein enrichment, isolates from this enrichment were predominantly narrow in their substrate ranges. The combined presence of refractory N compounds, such as membrane-bound proteins and histones in the protein extract [43], in addition to reported antimicrobial activity of histones [44] during the initial bacterial protein enrichment may have selected for bacteria with different traits. Thus, the reduced growth rate and subsequent narrow substrate range of these isolates during the substrate assay may be attributed to delayed or reduced activation of N scavenging enzymes in these bacteria.

Isolates from the nutrient broth enrichment had comparable proportions of members with broad and narrow substrate ranges. Some of the substrates effectively utilized by these isolates (labile free amino acids, algal exudate and ammonium) were also present in the nutrient broth enrichment (i.e. beef extract, labile and recalcitrant peptides and amino acids, nucleotide fractions, organic acids). Thus the subset of available and recalcitrant N compounds in the initial enrichment may have primed different nitrogen regulatory pathways in isolates from this enrichment, resulting in broad and narrow substrate ranges. Nitrate isolates also exhibited a similar profile as seen in nutrient broth. Lower growth rates of a variety of bacteria have been encountered when nitrate is provided as the only nitrogen source under aerobic conditions, due to lowered assimilatory nitrate reductase function [45, 46]. In addition, isolates under high nitrate conditions have been shown to reduce the production of N scavenging enzymes [32], and extracellular hydrolytic enzymes that degrade dissolved organic nitrogen species [47]. Thus the initial nitrate enrichment condition may have selected for isolates capable of effectively using some substrates but not others.

Nitrogen substrates examined inherently differed in their use regardless of the properties of the isolates. Specifically, the most complex, recalcitrant compounds (nucleic acids, peptidoglycan, and humics) were generally used poorly in comparison to other substrates. The crystalline and polymerized forms of these compounds makes them refractory to enzymatic degradation although degradation of humic matter [48], nucleic acids [49], and peptidoglycan [36] occurs under certain growth conditions. Thus, the observed low utilization of these recalcitrant substrates relative to the labile substrates may be a function of the minimal media conditions used in the substrate assay in this study.

## Conclusions

We observed differences in N substrate use patterns of bacteria from some complex and simple N-enrichments while others were unexpectedly similar. This is attributed to priming and metabolic flexibility. Taxonomic composition of bacterial isolate groups from the N-enrichments did not differ and was unrelated to N use, suggesting breadths of function and strong functional redundancy. Given the considerable functional variations among bacterial isolates, further studies examining expression of functional gene markers (transcripts) related to N utilization, quantification of gene abundances, and direct quantification of substrate utilization via stable isotope techniques may provide insights into the metabolic processes responsible for observed similar N utilization profiles from different enrichment conditions.

## Additional files

Additional file 1: Table S1. Taxonomical affiliations (to genus) of the 266 bacterial isolates from the 8 initial N-enrichments. Description of data: The name, and taxonomic identification to the genus level obtained for bacterial isolates obtained from this study using the Classifier tool in the Ribosomal Database Project (https://rdp.cme.msu.edu/classifier/classifier.jsp). (DOCX 33 kb)

Additional file 2: Document 1. Partial bacterial 16S rRNA sequence data. Description of data: The partial 16S rRNA bacterial sequence information for all 266 bacterial isolates obtained in this study. (DOCX 94 kb)

Additional file 3: Table S2. Title: Growth rates of all bacterial isolates from the initial N-enrichments on each of the 12 substrates. Description of data: The growth rates of each bacterial isolate from each N-enrichment on the 12 N-substrates used in the study. (DOCX 55 kb)

Additional file 4: Table S3. Title: Score differences (total isolate score – mean) and substrate range classification of bacterial isolates from the initial N-enrichments across substrates. Description of data: The score difference between mean scaled growth rates and total scaled growth rates for each isolate on the 12 N-substrates. Positive score differences represent isolates with broad substrate range and negative score differences represent isolates with narrow substrate range. (DOCX 28 kb)
