# Supplementary material for: Initial nitrogen enrichment conditions determines variations in nitrogen substrate utilization by heterotrophic bacterial isolates
Source: BMC Microbiol. 2017 Apr 4;17:87. doi: 10.1186/s12866-017-0993-7 (PMC5381026; doi:10.1186/s12866-017-0993-7)
Supplement: Supplementary file 1 — Taxonomical affiliations (to genus) of the 266 bacterial isolates from the 8 initial N-enrichments. Description of data: The name, and taxonomic identification to the genus level obtained for bacterial isolates obtained from this study using the Classifier tool in the Ribosomal Database Project (https://rdp.cme.msu.edu/classifier/classifier.jsp). (DOCX 33 kb) [file 12866_2017_993_MOESM1_ESM.docx]

**Table S1.** Taxonomical affiliations (to genus) of the 266 bacterial isolates from the 8 initial N-enrichments.

| **N-enrichment** | **Isolates** | **Kingdom** | **Phylum** | **Class** | **Order** | **Family** | **Genus** |
| --- | --- | --- | --- | --- | --- | --- | --- |
| Nutrient broth | J01 | Bacteria | Proteobacteria | Gammaproteobacteria | Alteromonadales | *Shewanellaceae* | *Shewanella* |
| Nutrient broth | J02 | Bacteria | Proteobacteria | Gammaproteobacteria | Enterobacteriales | *Enterobacteriaceae* | *Hafnia* |
| Nutrient broth | J03 | Bacteria | Proteobacteria | Gammaproteobacteria | Enterobacteriales | *Enterobacteriaceae* | *Hafnia* |
| Nutrient broth | J04 | Bacteria | Proteobacteria | Gammaproteobacteria | Alteromonadales | *Shewanellaceae* | *Shewanella* |
| Nutrient broth | J05 | Bacteria | Proteobacteria | Gammaproteobacteria | Pseudomonadales | *Pseudomonadaceae* | *Pseudomonas* |
| Nutrient broth | J06 | Bacteria | Proteobacteria | Gammaproteobacteria | Aeromonadales | *Aeromonadaceae* | *Aeromonas* |
| Nutrient broth | J07 | Bacteria | Proteobacteria | Gammaproteobacteria | Pseudomonadales | *Pseudomonadaceae* | *Pseudomonas* |
| Nutrient broth | J08 | Bacteria | Proteobacteria | Betaproteobacteria | Burkholderiales | *Comamonadaceae* | *Limnohabitans* |
| Nutrient broth | J09 | Bacteria | Proteobacteria | Gammaproteobacteria | Pseudomonadales | *Pseudomonadaceae* | *Pseudomonas* |
| Nutrient broth | J10 | Bacteria | Proteobacteria | Gammaproteobacteria | Alteromonadales | *Shewanellaceae* | *Shewanella* |
| Nutrient broth | J11 | Bacteria | Proteobacteria | Gammaproteobacteria | Aeromonadales | *Aeromonadaceae* | *Aeromonas* |
| Nutrient broth | J12 | Bacteria | Firmicutes | Bacilli | Bacillales | *Bacillaceae* |  |
| Nutrient broth | J13 | Bacteria | Proteobacteria | Gammaproteobacteria | Aeromonadales | *Aeromonadaceae* | *Aeromonas* |
| Nutrient broth | J14 | Bacteria | Proteobacteria | Gammaproteobacteria | Aeromonadales | *Aeromonadaceae* | *Aeromonas* |
| Nutrient broth | J15 | Bacteria | Proteobacteria | Betaproteobacteria | Burkholderiales | *Comamonadaceae* | *Comamonas* |
| Nutrient broth | J81 | Bacteria | Proteobacteria | Gammaproteobacteria | Enterobacteriales | *Enterobacteriaceae* | *Serratia* |
| Nutrient broth | J82 | Bacteria | Proteobacteria | Gammaproteobacteria | Enterobacteriales | *Enterobacteriaceae* | *Hafnia* |
| Nutrient broth | J83 | Bacteria | Proteobacteria | Gammaproteobacteria | Enterobacteriales | *Enterobacteriaceae* | *Serratia* |
| Nutrient broth | J84 | Bacteria | Proteobacteria | Gammaproteobacteria | Enterobacteriales | *Enterobacteriaceae* | *Pectobacterium* |
| Nutrient broth | J85 | Bacteria | Proteobacteria | Gammaproteobacteria | Enterobacteriales | *Enterobacteriaceae* | *Erwinia* |
| Nutrient broth | J86 | Bacteria | Proteobacteria | Gammaproteobacteria | Enterobacteriales | *Enterobacteriaceae* | *Erwinia* |
| Nutrient broth | J87 | Bacteria | Proteobacteria | Gammaproteobacteria | Enterobacteriales | *Enterobacteriaceae* | *Erwinia* |
| Nutrient broth | J88 | Bacteria | Proteobacteria | Gammaproteobacteria | Enterobacteriales | *Enterobacteriaceae* | *Pectobacterium* |
| Nutrient broth | J89 | Bacteria | Proteobacteria | Gammaproteobacteria | Enterobacteriales | *Enterobacteriaceae* | *Yersinia* |
| Nutrient broth | J90 | Bacteria | Firmicutes | Bacilli | Bacillales | *Bacillaceae* |  |
| Nutrient broth | J91 | Bacteria | Proteobacteria | Gammaproteobacteria | Enterobacteriales | *Enterobacteriaceae* | *Hafnia* |
| Nutrient broth | J92 | Bacteria | Firmicutes | Bacilli | Bacillales | *Bacillaceae* |  |
| Nutrient broth | J93 | Bacteria | Firmicutes | Bacilli | Bacillales | *Bacillaceae* |  |
| Nutrient broth | J94 | Bacteria | Firmicutes | Bacilli | Bacillales | *Bacillaceae* |  |
| Nutrient broth | J95 | Bacteria | Firmicutes | Bacilli | Bacillales | *Bacillaceae* |  |
| Nutrient broth | SS51 | Bacteria | Proteobacteria | Gammaproteobacteria | Alteromonadales | *Pseudoalteromonadaceae* | *Psychrosphaera* |
| Nutrient broth | SS52 | Bacteria | Proteobacteria | Gammaproteobacteria | Pseudomonadales | *Pseudomonadaceae* | *Serpens* |
| Nutrient broth | SS53 | Bacteria | Proteobacteria | Gammaproteobacteria | Alteromonadales | *Shewanellaceae* | *Shewanella* |
| Nutrient broth | SS54 | Bacteria | Proteobacteria | Gammaproteobacteria | Pseudomonadales | *Pseudomonadaceae* | *Pseudomonas* |
| Nutrient broth | SS55 | Bacteria | Proteobacteria | Betaproteobacteria | Burkholderiales | *Burkholderiales_incertae_sedis* | *Xylophilus* |
| Nutrient broth | SS56 | Bacteria | Proteobacteria | Betaproteobacteria | Burkholderiales | *Comamonadaceae* | *Comamonas* |
| Nutrient broth | SS57 | Bacteria | Proteobacteria | Betaproteobacteria | Burkholderiales | *Comamonadaceae* | *Pseudacidovorax* |
| Nutrient broth | SS58 | Bacteria | Proteobacteria | Betaproteobacteria | Burkholderiales | *Comamonadaceae* | *Pseudacidovorax* |
| Nutrient broth | SS59 | Bacteria | Proteobacteria | Gammaproteobacteria | Alteromonadales | *Ferrimonadaceae* | *Paraferrimonas* |
| Nutrient broth | SS6 | Bacteria | Proteobacteria | Gammaproteobacteria | Enterobacteriales | *Enterobacteriaceae* | *Yokenella* |
| Nutrient broth | SS60 | Bacteria | Proteobacteria | Gammaproteobacteria | Enterobacteriales | *Enterobacteriaceae* | *Rahnella* |
| Nutrient broth | SS61 | Bacteria | Proteobacteria | Betaproteobacteria | Burkholderiales | *Comamonadaceae* | *Comamonas* |
| Nutrient broth | SS62 | Bacteria | Proteobacteria | Betaproteobacteria | Burkholderiales | *Comamonadaceae* | *Comamonas* |
| Nutrient broth | SS63 | Archaea | Crenarchaeota | Thermoprotei | Thermoproteales | *Thermofilaceae* | *Thermofilum* |
| Nutrient broth | SS64 | Bacteria | Proteobacteria | Betaproteobacteria | Burkholderiales | *Comamonadaceae* | *Comamonas* |
| Nutrient broth | SYC53 | Bacteria | Proteobacteria | Betaproteobacteria | Burkholderiales | *Comamonadaceae* | *Hylemonella* |
| Nutrient broth | SYC54 | Bacteria | Proteobacteria | Betaproteobacteria | Burkholderiales | *Comamonadaceae* | *Comamonas* |
| Nutrient broth | SYC55 | Bacteria | Proteobacteria | Betaproteobacteria | Burkholderiales | *Comamonadaceae* | *Comamonas* |
| Nutrient broth | SYC56 | Bacteria | Proteobacteria | Betaproteobacteria | Burkholderiales | *Comamonadaceae* | *Comamonas* |
| Nutrient broth | SYC57 | Bacteria | Proteobacteria | Betaproteobacteria | Burkholderiales | *Comamonadaceae* | *Extensimonas* |
| Nutrient broth | SYC58 | Bacteria | Proteobacteria | Betaproteobacteria | Burkholderiales | *Comamonadaceae* | *Comamonas* |
| Nutrient broth | SYC59 | Bacteria | Proteobacteria | Betaproteobacteria | Burkholderiales | *Comamonadaceae* | *Comamonas* |
| Nutrient broth | SYC6 | Bacteria | Firmicutes | Bacilli | Lactobacillales | *Carnobacteriaceae* | *Isobaculum* |
| Nutrient broth | SYC60 | Bacteria | Proteobacteria | Betaproteobacteria | Rhodocyclales | *Rhodocyclaceae* | *Georgfuchsia* |
| Nutrient broth | SYC61 | Bacteria | Proteobacteria | Gammaproteobacteria | Aeromonadales | *Aeromonadaceae* | *Aeromonas* |
| Nutrient broth | SYC62 | Bacteria | Proteobacteria | Betaproteobacteria | Burkholderiales | *Comamonadaceae* | *Comamonas* |
| Nutrient broth | SYC63 | Bacteria | Proteobacteria | Betaproteobacteria | Burkholderiales | *Comamonadaceae* | *Comamonas* |
| Nutrient broth | SYC64 | Bacteria | Proteobacteria | Betaproteobacteria | Burkholderiales | *Comamonadaceae* | *Comamonas* |
| Glycine | J16 | Bacteria | Proteobacteria | Gammaproteobacteria | Pseudomonadales | *Pseudomonadaceae* | *Pseudomonas* |
| Glycine | J17 | Bacteria | Proteobacteria | Gammaproteobacteria | Pseudomonadales | *Pseudomonadaceae* | *Pseudomonas* |
| Glycine | J18 | Bacteria | Proteobacteria | Gammaproteobacteria | Pseudomonadales | *Pseudomonadaceae* | *Pseudomonas* |
| Glycine | J19 | Bacteria | Proteobacteria | Gammaproteobacteria | Enterobacteriales | *Enterobacteriaceae* | *Citrobacter* |
| Glycine | J20 | Bacteria | Proteobacteria | Gammaproteobacteria | Pseudomonadales | *Pseudomonadaceae* | *Pseudomonas* |
| Glycine | J21 | Bacteria | Proteobacteria | Gammaproteobacteria | Pseudomonadales | *Pseudomonadaceae* | *Pseudomonas* |
| Glycine | J22 | Bacteria | Proteobacteria | Gammaproteobacteria | Enterobacteriales | *Enterobacteriaceae* | *Enterobacter* |
| Glycine | J23 | Bacteria | Proteobacteria | Gammaproteobacteria | Pseudomonadales | *Pseudomonadaceae* | *Pseudomonas* |
| Glycine | J24 | Bacteria | Proteobacteria | Gammaproteobacteria | Pseudomonadales | *Pseudomonadaceae* | *Pseudomonas* |
| Glycine | SYC65 | Bacteria | Proteobacteria | Betaproteobacteria | Burkholderiales | *Comamonadaceae* | *Comamonas* |
| Glycine | SYC66 | Bacteria | Proteobacteria | Betaproteobacteria | Burkholderiales | *Comamonadaceae* | *Comamonas* |
| Glycine | SYC67 | Bacteria | Proteobacteria | Betaproteobacteria | Burkholderiales | *Comamonadaceae* | *Comamonas* |
| Glycine | SYC68 | Archaea | Crenarchaeota | Thermoprotei | Thermoproteales | *Thermofilaceae* | *Thermofilum* |
| Glycine | SYC69 | Bacteria | Proteobacteria | Gammaproteobacteria | Pseudomonadales | *Pseudomonadaceae* | *Azomonas* |
| Glycine | SYC70 | Bacteria | Proteobacteria | Gammaproteobacteria | Enterobacteriales | *Enterobacteriaceae* | *Yokenella* |
| Glycine | SYC71 | Bacteria | Firmicutes | Bacilli | Bacillales | *Bacillaceae* |  |
| Glycine | SYC72 | Bacteria | Proteobacteria | Gammaproteobacteria | Enterobacteriales | *Enterobacteriaceae* | *Rahnella* |
| Glycine | SYC73 | Bacteria | Proteobacteria | Gammaproteobacteria | Enterobacteriales | *Enterobacteriaceae* | *Rahnella* |
| Glycine | SYC74 | Bacteria | Firmicutes | Bacilli | Bacillales | *Bacillaceae* |  |
| Glycine | SS41 | Bacteria | Proteobacteria | Gammaproteobacteria | Pseudomonadales | *Pseudomonadaceae* | *Azomonas* |
| Glycine | SS42 | Bacteria | Proteobacteria | Gammaproteobacteria | Enterobacteriales | *Enterobacteriaceae* | *Obesumbacterium* |
| Glycine | SS43 | Bacteria | Proteobacteria | Gammaproteobacteria | Enterobacteriales | *Enterobacteriaceae* | *Raoultella* |
| Glycine | SS44 | Bacteria | Proteobacteria | Gammaproteobacteria | Pseudomonadales | *Pseudomonadaceae* | *Serpens* |
| Glycine | SS45 | Bacteria | Proteobacteria | Gammaproteobacteria | Enterobacteriales | *Enterobacteriaceae* | *Obesumbacterium* |
| Glycine | SS46 | Bacteria | Proteobacteria | Gammaproteobacteria | Pseudomonadales | *Pseudomonadaceae* | *Pseudomonas* |
| Glycine | SS47 | Bacteria | Proteobacteria | Gammaproteobacteria | Oceanospirillales | *Oceanospirillaceae* | *Oceanobacter* |
| Glycine | SS48 | Bacteria | Proteobacteria | Gammaproteobacteria | Pseudomonadales | *Pseudomonadaceae* | *Azomonas* |
| Glycine | SS49 | Bacteria | Proteobacteria | Gammaproteobacteria | Candidatus |  |  |
| Glycine | SS50 | Bacteria | Proteobacteria | Gammaproteobacteria | Pseudomonadales | *Pseudomonadaceae* | *Serpens* |
| Nitrate | J25 | Bacteria | Proteobacteria | Gammaproteobacteria | Pseudomonadales | *Pseudomonadaceae* | *Pseudomonas* |
| Nitrate | J26 | Bacteria | Proteobacteria | Gammaproteobacteria | Enterobacteriales | *Enterobacteriaceae* | *Enterobacter* |
| Nitrate | J27 | Bacteria | Proteobacteria | Gammaproteobacteria | Pseudomonadales | *Pseudomonadaceae* | *Pseudomonas* |
| Nitrate | J28 | Bacteria | Proteobacteria | Gammaproteobacteria | Enterobacteriales | *Enterobacteriaceae* | *Raoultella* |
| Nitrate | J29 | Bacteria | Proteobacteria | Gammaproteobacteria | Aeromonadales | *Aeromonadaceae* | *Aeromonas* |
| Nitrate | J30 | Bacteria | Proteobacteria | Betaproteobacteria | Burkholderiales | *Comamonadaceae* | *Comamonas* |
| Nitrate | J31 | Bacteria | Proteobacteria | Gammaproteobacteria | Pseudomonadales | *Pseudomonadaceae* | *Pseudomonas* |
| Nitrate | J32 | Bacteria | Proteobacteria | Gammaproteobacteria | Pseudomonadales | *Pseudomonadaceae* | *Pseudomonas* |
| Nitrate | SYC21 | Bacteria | Proteobacteria | Gammaproteobacteria | Methylococcales | *Methylococcaceae* | *Methylosphaera* |
| Nitrate | SYC22 | Bacteria | Proteobacteria | Gammaproteobacteria | Pseudomonadales | *Pseudomonadaceae* | *Azomonas* |
| Nitrate | SYC23 | Bacteria | Proteobacteria | Gammaproteobacteria | Enterobacteriales | *Enterobacteriaceae* | *Leclercia* |
| Nitrate | SYC24 | Bacteria | Proteobacteria | Gammaproteobacteria | Methylococcales | *Methylococcaceae* | *Methylomarinum* |
| Nitrate | SYC25 | Bacteria | Proteobacteria | Gammaproteobacteria | Enterobacteriales | *Enterobacteriaceae* | *Raoultella* |
| Nitrate | SYC26 | Bacteria | Proteobacteria | Gammaproteobacteria | Enterobacteriales | *Enterobacteriaceae* | *Rahnella* |
| Nitrate | SYC27 | Bacteria | Proteobacteria | Gammaproteobacteria | Enterobacteriales | *Enterobacteriaceae* | *Leclercia* |
| Nitrate | SYC28 | Bacteria | Proteobacteria | Betaproteobacteria | Burkholderiales | *Comamonadaceae* | *Extensimonas* |
| Nitrate | SYC29 | Archaea | Crenarchaeota | Thermoprotei | Thermoproteales | *Thermoproteaceae* | *Caldivirga* |
| Nitrate | SYC30 | Bacteria | Proteobacteria | Gammaproteobacteria | Enterobacteriales | *Enterobacteriaceae* | *Lelliottia* |
| Nitrate | SS31 | Bacteria | Proteobacteria | Gammaproteobacteria | Enterobacteriales | *Enterobacteriaceae* | *Leclercia* |
| Nitrate | SS32 | Bacteria | Proteobacteria | Gammaproteobacteria | Enterobacteriales | *Enterobacteriaceae* | *Rahnella* |
| Nitrate | SS33 | Bacteria | Proteobacteria | Gammaproteobacteria | Enterobacteriales | *Enterobacteriaceae* | *Lelliottia* |
| Nitrate | SS34 | Bacteria | Proteobacteria | Gammaproteobacteria | Enterobacteriales | *Enterobacteriaceae* | *Samsonia* |
| Nitrate | SS35 | Bacteria | Proteobacteria | Gammaproteobacteria | Enterobacteriales | *Enterobacteriaceae* | *Serratia* |
| Nitrate | SS36 | Bacteria | Proteobacteria | Betaproteobacteria | Burkholderiales | *Comamonadaceae* | *Extensimonas* |
| Nitrate | SS37 | Bacteria | Proteobacteria | Gammaproteobacteria | Enterobacteriales | *Enterobacteriaceae* | *Rahnella* |
| Nitrate | SS38 | Bacteria | Proteobacteria | Gammaproteobacteria | Enterobacteriales | *Enterobacteriaceae* | *Rahnella* |
| Nitrate | SS39 | Bacteria | Proteobacteria | Gammaproteobacteria | Enterobacteriales | *Enterobacteriaceae* | *Phaseolibacter* |
| Nitrate | SS40 | Bacteria | Proteobacteria | Gammaproteobacteria | Enterobacteriales | *Enterobacteriaceae* | *Obesumbacterium* |
| Urea | J33 | Bacteria | Proteobacteria | Gammaproteobacteria | Pseudomonadales | *Pseudomonadaceae* | *Pseudomonas* |
| Urea | J34 | Bacteria | Proteobacteria | Gammaproteobacteria | Pseudomonadales | *Pseudomonadaceae* | *Pseudomonas* |
| Urea | J35 | Bacteria | Proteobacteria | Gammaproteobacteria | Enterobacteriales | *Enterobacteriaceae* | *Buttiauxella* |
| Urea | J36 | Bacteria | Proteobacteria | Gammaproteobacteria | Enterobacteriales | *Enterobacteriaceae* | *Serratia* |
| Urea | J37 | Bacteria | Proteobacteria | Gammaproteobacteria | Enterobacteriales | *Enterobacteriaceae* | *Serratia* |
| Urea | J38 | Bacteria | Proteobacteria | Gammaproteobacteria | Enterobacteriales | *Enterobacteriaceae* | *Pectobacterium* |
| Urea | J39 | Bacteria | Proteobacteria | Gammaproteobacteria | Enterobacteriales | *Enterobacteriaceae* | *Rahnella* |
| Urea | J40 | Bacteria | Firmicutes | Bacilli | Bacillales | *Bacillaceae* | *Bacillus* |
| Urea | SYC11 | Bacteria | Proteobacteria | Gammaproteobacteria | Enterobacteriales | *Enterobacteriaceae* | *Leclercia* |
| Urea | SYC12 | Bacteria | Proteobacteria | Gammaproteobacteria | Enterobacteriales | *Enterobacteriaceae* | *Lelliottia* |
| Urea | SYC13 | Bacteria | Proteobacteria | Betaproteobacteria | Burkholderiales | *Comamonadaceae* | *Ottowia* |
| Urea | SYC14 | Bacteria | Proteobacteria | Betaproteobacteria | Burkholderiales | *Comamonadaceae* | *Pseudacidovorax* |
| Urea | SYC15 | Bacteria | Proteobacteria | Betaproteobacteria | Burkholderiales | *Comamonadaceae* | *Comamonas* |
| Urea | SYC16 | Bacteria | Proteobacteria | Gammaproteobacteria | Pseudomonadales | *Pseudomonadaceae* | *Azomonas* |
| Urea | SYC17 | Bacteria | Proteobacteria | Gammaproteobacteria | Gammaproteobacteria_incertae_sedis | *Unassigned gammaproteobacterium* |  |
| Urea | SYC18 | Bacteria | Proteobacteria | Gammaproteobacteria | Enterobacteriales | *Enterobacteriaceae* | *Buttiauxella* |
| Urea | SYC19 | Bacteria | Proteobacteria | Gammaproteobacteria | Enterobacteriales | *Enterobacteriaceae* | *Lelliottia* |
| Urea | SYC20 | Bacteria | Proteobacteria | Gammaproteobacteria | Pseudomonadales | *Pseudomonadaceae* | *Azomonas* |
| Urea | SS1 | Bacteria | Proteobacteria | Betaproteobacteria | Methylophilales | *Methylophilaceae* | *Methylotenera* |
| Urea | SS2 | Bacteria | Proteobacteria | Gammaproteobacteria | Pseudomonadales | *Pseudomonadaceae* | *Serpens* |
| Urea | SS3 | Bacteria | Proteobacteria | Gammaproteobacteria | Enterobacteriales | *Enterobacteriaceae* | *Sodalis* |
| Urea | SS4 | Bacteria | Proteobacteria | Gammaproteobacteria | Alteromonadales | *Alteromonadales_incertae_sedis* | *Teredinibacter* |
| Urea | SS5 | Bacteria | Proteobacteria | Gammaproteobacteria | Enterobacteriales | *Enterobacteriaceae* | *Yokenella* |
| Urea | SS6 | Bacteria | Proteobacteria | Gammaproteobacteria | Enterobacteriales | *Enterobacteriaceae* | *Yokenella* |
| Urea | SS7 | Bacteria | Proteobacteria | Gammaproteobacteria | Enterobacteriales | *Enterobacteriaceae* | *Lelliottia* |
| Urea | SS8 | Bacteria | Proteobacteria | Gammaproteobacteria | Enterobacteriales | *Enterobacteriaceae* | *Rahnella* |
| Urea | SS9 | Bacteria | Chloroflexi | Thermomicrobia | Sphaerobacteridae | *Sphaerobacterales* | *Sphaerobacterineae* |
| Urea | SS10 | Bacteria | Proteobacteria | Gammaproteobacteria | Pseudomonadales | *Pseudomonadaceae* | *Pseudomonas* |
| Ammonium | J41 | Bacteria | Proteobacteria | Gammaproteobacteria | Aeromonadales | *Aeromonadaceae* | *Aeromonas* |
| Ammonium | J42 | Bacteria | Proteobacteria | Betaproteobacteria | Burkholderiales | *Comamonadaceae* | *Comamonas* |
| Ammonium | J43 | Bacteria | Proteobacteria | Gammaproteobacteria | Pseudomonadales | *Pseudomonadaceae* | *Pseudomonas* |
| Ammonium | J44 | Bacteria | Proteobacteria | Gammaproteobacteria | Pseudomonadales | *Pseudomonadaceae* | *Pseudomonas* |
| Ammonium | J45 | Bacteria | Proteobacteria | Gammaproteobacteria | Pseudomonadales | *Pseudomonadaceae* | *Pseudomonas* |
| Ammonium | J46 | Bacteria | Proteobacteria | Gammaproteobacteria | Pseudomonadales | *Pseudomonadaceae* | *Pseudomonas* |
| Ammonium | J47 | Bacteria | Proteobacteria | Gammaproteobacteria | Enterobacteriales | *Enterobacteriaceae* | *Buttiauxella* |
| Ammonium | J48 | Bacteria | Proteobacteria | Gammaproteobacteria | Enterobacteriales | *Enterobacteriaceae* | *Serratia* |
| Ammonium | J49 | Bacteria | Proteobacteria | Gammaproteobacteria | Enterobacteriales | *Enterobacteriaceae* | *Serratia* |
| Ammonium | J50 | Bacteria | Proteobacteria | Gammaproteobacteria | Enterobacteriales | *Enterobacteriaceae* | *Serratia* |
| Ammonium | J51 | Bacteria | Proteobacteria | Gammaproteobacteria | Enterobacteriales | *Enterobacteriaceae* | *Rahnella* |
| Ammonium | SYC31 | Bacteria | Proteobacteria | Gammaproteobacteria | Enterobacteriales | *Enterobacteriaceae* | *Pragia* |
| Ammonium | SYC32 | Bacteria | Proteobacteria | Gammaproteobacteria | Enterobacteriales | *Enterobacteriaceae* | *Rahnella* |
| Ammonium | SYC33 | Bacteria | Proteobacteria | Betaproteobacteria | Burkholderiales | *Comamonadaceae* | *Alicycliphilus* |
| Ammonium | SYC34 | Bacteria | Proteobacteria | Gammaproteobacteria | Enterobacteriales | *Enterobacteriaceae* | *Arsenophonus* |
| Ammonium | SYC35 | Bacteria | Bacteroidetes | Sphingobacteriia | Sphingobacteriales | *Chitinophagaceae* | *Parasegetibacter* |
| Ammonium | SYC36 | Bacteria | Proteobacteria | Gammaproteobacteria | Enterobacteriales | *Enterobacteriaceae* | *Rahnella* |
| Ammonium | SYC37 | Bacteria | Proteobacteria | Gammaproteobacteria | Pseudomonadales | *Pseudomonadaceae* | *Azorhizophilus* |
| Ammonium | SYC38 | Bacteria | Proteobacteria | Gammaproteobacteria | Enterobacteriales | *Enterobacteriaceae* | *Obesumbacterium* |
| Ammonium | SYC39 | Bacteria | Proteobacteria | Gammaproteobacteria | Enterobacteriales | *Enterobacteriaceae* | *Leclercia* |
| Ammonium | SYC40 | Bacteria | Proteobacteria | Gammaproteobacteria | Pseudomonadales | *Pseudomonadaceae* | *Serpens* |
| Ammonium | SS65 | Bacteria | Proteobacteria | Gammaproteobacteria | Enterobacteriales | *Enterobacteriaceae* | *Raoultella* |
| Ammonium | SS66 | Bacteria | Proteobacteria | Gammaproteobacteria | Pseudomonadales | *Pseudomonadaceae* | *Serpens* |
| Ammonium | SS67 | Bacteria | Firmicutes | Bacilli | Bacillales | *Bacillaceae* | *Falsibacillus* |
| Ammonium | SS68 | Bacteria | Proteobacteria | Gammaproteobacteria | Enterobacteriales | *Enterobacteriaceae* | *Rahnella* |
| Ammonium | SS69 | Bacteria | Proteobacteria | Gammaproteobacteria | Enterobacteriales | *Enterobacteriaceae* | *Rahnella* |
| Ammonium | SS70 | Bacteria | Proteobacteria | Gammaproteobacteria | Enterobacteriales | *Enterobacteriaceae* | *Lelliottia* |
| Ammonium | SS71 | Bacteria | Proteobacteria | Gammaproteobacteria | Enterobacteriales | *Enterobacteriaceae* | *Leminorella* |
| Ammonium | SS72 | Bacteria | Proteobacteria | Gammaproteobacteria | Enterobacteriales | *Enterobacteriaceae* | *Samsonia* |
| Ammonium | SS73 | Archaea | Euryarchaeota | Halobacteria | Halobacteriales | *Halobacteriaceae* | *Halorussus* |
| Ammonium | SS74 | Bacteria | Proteobacteria | Gammaproteobacteria | Enterobacteriales | *Enterobacteriaceae* | *Leclercia* |
| Ammonium | SS75 | Bacteria | Proteobacteria | Gammaproteobacteria | Aeromonadales | *Aeromonadaceae* | *Aeromonas* |
| Defined mixture of N | J52 | Bacteria | Proteobacteria | Gammaproteobacteria | Enterobacteriales | *Enterobacteriaceae* | *Raoultella* |
| Defined mixture of N | J53 | Bacteria | Proteobacteria | Gammaproteobacteria | Enterobacteriales | *Enterobacteriaceae* | *Serratia* |
| Defined mixture of N | J54 | Bacteria | Proteobacteria | Gammaproteobacteria | Enterobacteriales | *Enterobacteriaceae* | *Serratia* |
| Defined mixture of N | J55 | Bacteria | Proteobacteria | Gammaproteobacteria | Enterobacteriales | *Enterobacteriaceae* | *Lelliottia* |
| Defined mixture of N | J56 | Bacteria | Proteobacteria | Gammaproteobacteria | Enterobacteriales | *Enterobacteriaceae* | *Rahnella* |
| Defined mixture of N | J57 | Bacteria | Proteobacteria | Gammaproteobacteria | Enterobacteriales | *Enterobacteriaceae* | *Erwinia* |
| Defined mixture of N | J58 | Bacteria | Proteobacteria | Gammaproteobacteria | Enterobacteriales | *Enterobacteriaceae* | *Erwinia* |
| Defined mixture of N | J59 | Bacteria | Proteobacteria | Gammaproteobacteria | Pseudomonadales | *Pseudomonadaceae* | *Pseudomonas* |
| Defined mixture of N | J60 | Bacteria | Proteobacteria | Gammaproteobacteria | Enterobacteriales | *Enterobacteriaceae* | *Serratia* |
| Defined mixture of N | SYC41 | Bacteria | Proteobacteria | Gammaproteobacteria | Enterobacteriales | *Enterobacteriaceae* | *Phaseolibacter* |
| Defined mixture of N | SYC42 | Bacteria | Proteobacteria | Gammaproteobacteria | Enterobacteriales | *Enterobacteriaceae* | *Rahnella* |
| Defined mixture of N | SYC43 | Bacteria | Proteobacteria | Gammaproteobacteria | Enterobacteriales | *Enterobacteriaceae* | *Obesumbacterium* |
| Defined mixture of N | SYC44 | Bacteria | Candidatus Calescamantes | Calescibacterium |  | *Unassigned Calescibacterium* |  |
| Defined mixture of N | SYC45 | Bacteria | Proteobacteria | Gammaproteobacteria | Enterobacteriales | *Enterobacteriaceae* | *Pragia* |
| Defined mixture of N | SYC46 | Bacteria | Proteobacteria | Betaproteobacteria | Burkholderiales | *Comamonadaceae* | *Delftia* |
| Defined mixture of N | SYC47 | Bacteria | Proteobacteria | Gammaproteobacteria | Pseudomonadales | *Pseudomonadaceae* | *Azomonas* |
| Defined mixture of N | SYC48 | Bacteria | Proteobacteria | Gammaproteobacteria | Pseudomonadales | *Pseudomonadaceae* | *Azomonas* |
| Defined mixture of N | SYC49 | Bacteria | Proteobacteria | Gammaproteobacteria | Enterobacteriales | *Enterobacteriaceae* | *Raoultella* |
| Defined mixture of N | SYC50 | Bacteria | Proteobacteria | Betaproteobacteria | Burkholderiales | *Comamonadaceae* | *Comamonas* |
| Defined mixture of N | SYC51 | Bacteria | Proteobacteria | Gammaproteobacteria | Pseudomonadales | *Pseudomonadaceae* | *Serpens* |
| Defined mixture of N | SYC52 | Bacteria | Proteobacteria | Betaproteobacteria | Burkholderiales | *Comamonadaceae* | *Comamonas* |
| Defined mixture of N | SS21 | Bacteria | Proteobacteria | Gammaproteobacteria | Pseudomonadales | *Pseudomonadaceae* | *Azomonas* |
| Defined mixture of N | SS22 | Bacteria | Proteobacteria | Gammaproteobacteria | Gammaproteobacteria_incertae_sedis | *Unassigned gammaproteobacterium* |  |
| Defined mixture of N | SS23 | Bacteria | Proteobacteria | Gammaproteobacteria | Enterobacteriales | *Enterobacteriaceae* | *Leclercia* |
| Defined mixture of N | SS24 | Bacteria | Proteobacteria | Gammaproteobacteria | Enterobacteriales | *Enterobacteriaceae* | *Obesumbacterium* |
| Defined mixture of N | SS25 | Bacteria | Proteobacteria | Gammaproteobacteria | Enterobacteriales | *Enterobacteriaceae* | *Lelliottia* |
| Defined mixture of N | SS26 | Bacteria | Proteobacteria | Gammaproteobacteria | Pseudomonadales | *Pseudomonadaceae* | *Azomonas* |
| Defined mixture of N | SS27 | Bacteria | Proteobacteria | Gammaproteobacteria | Enterobacteriales | *Enterobacteriaceae* | *Obesumbacterium* |
| Defined mixture of N | SS28 | Bacteria | Proteobacteria | Gammaproteobacteria | Enterobacteriales | *Enterobacteriaceae* | *Raoultella* |
| Defined mixture of N | SS29 | Bacteria | Proteobacteria | Gammaproteobacteria | Enterobacteriales | *Enterobacteriaceae* | *Leclercia* |
| Defined mixture of N | SS30 | Bacteria | Proteobacteria | Gammaproteobacteria | Pseudomonadales | *Pseudomonadaceae* | *Azomonas* |
| Bacterial Protein | J61 | Bacteria | Firmicutes | Bacilli | Bacillales | *Bacillaceae* | *Bacillus* |
| Bacterial Protein | J62 | Bacteria | Firmicutes | Bacilli | Bacillales | *Staphylococcaceae* | *Staphylococcus* |
| Bacterial Protein | J64 | Bacteria | Proteobacteria | Gammaproteobacteria | Enterobacteriales | *Enterobacteriaceae* | *Erwinia* |
| Bacterial Protein | J65A | Bacteria | Proteobacteria | Gammaproteobacteria | Enterobacteriales | *Enterobacteriaceae* | *Erwinia* |
| Bacterial Protein | J65B | Bacteria | Firmicutes | Bacilli | Bacillales | *Bacillaceae* | *Bacillus* |
| Bacterial Protein | J66 | Bacteria | Proteobacteria | Gammaproteobacteria | Enterobacteriales | *Enterobacteriaceae* | *Serratia* |
| Bacterial Protein | SYC1 | Bacteria | Proteobacteria | Gammaproteobacteria | Enterobacteriales | *Enterobacteriaceae* | *Rahnella* |
| Bacterial Protein | SYC2 | Bacteria | Proteobacteria | Gammaproteobacteria | Enterobacteriales | *Enterobacteriaceae* | *Rahnella* |
| Bacterial Protein | SYC3 | Bacteria | Proteobacteria | Gammaproteobacteria | Pseudomonadales | *Pseudomonadaceae* | *Azorhizophilus* |
| Bacterial Protein | SYC4 | Bacteria | Proteobacteria | Gammaproteobacteria | Pseudomonadales | *Pseudomonadaceae* | *Pseudomonas* |
| Bacterial Protein | SYC5 | Bacteria | Proteobacteria | Gammaproteobacteria | Pseudomonadales | *Pseudomonadaceae* | *Pseudomonas* |
| Bacterial Protein | SYC6 | Bacteria | Firmicutes | Bacilli | Lactobacillales | *Carnobacteriaceae* | *Isobaculum* |
| Bacterial Protein | SYC7 | Bacteria | Proteobacteria | Gammaproteobacteria | Enterobacteriales | *Enterobacteriaceae* | *Rahnella* |
| Bacterial Protein | SYC8 | Bacteria | Proteobacteria | Gammaproteobacteria | Enterobacteriales | *Enterobacteriaceae* | *Rahnella* |
| Bacterial Protein | SYC9 | Bacteria | Proteobacteria | Gammaproteobacteria | Pseudomonadales | *Pseudomonadaceae* | *Pseudomonas* |
| Bacterial Protein | SYC10 | Bacteria | Proteobacteria | Gammaproteobacteria | Pseudomonadales | *Pseudomonadaceae* | *Serpens* |
| Bacterial Protein | SS11 | Bacteria | Proteobacteria | Betaproteobacteria | Burkholderiales | *Comamonadaceae* | *Ottowia* |
| Bacterial Protein | SS12 | Bacteria | Planctomycetes | Planctomycetia | Planctomycetales | *Planctomycetaceae* | *Zavarzinella* |
| Bacterial Protein | SS13 | Bacteria | Proteobacteria | Gammaproteobacteria | Enterobacteriales | *Enterobacteriaceae* | *Rahnella* |
| Bacterial Protein | SS14 | Bacteria | Proteobacteria | Gammaproteobacteria | Enterobacteriales | *Enterobacteriaceae* | *Rahnella* |
| Bacterial Protein | SS15 | Archaea | Crenarchaeota | Thermoprotei | Thermoproteales | *Thermoproteaceae* | *Caldivirga* |
| Bacterial Protein | SS16 | Bacteria | Proteobacteria | Gammaproteobacteria | Pseudomonadales | *Pseudomonadaceae* | *Azorhizophilus* |
| Bacterial Protein | SS17 | Bacteria | Proteobacteria | Gammaproteobacteria | Methylococcales | *Methylococcaceae* | *Methylosphaera* |
| Bacterial Protein | SS18 | Bacteria | Proteobacteria | Gammaproteobacteria | Enterobacteriales | *Enterobacteriaceae* | *Lelliottia* |
| Bacterial Protein | SS19 | Bacteria | Proteobacteria | Betaproteobacteria | Burkholderiales | *Comamonadaceae* | *Pseudacidovorax* |
| Bacterial Protein | SS20 | Bacteria | Proteobacteria | Betaproteobacteria | Burkholderiales | *Comamonadaceae* | *Comamonas* |
| Tryptophan | J67 | Bacteria | Proteobacteria | Gammaproteobacteria | Enterobacteriales | *Enterobacteriaceae* | *Serratia* |
| Tryptophan | J68 | Bacteria | Proteobacteria | Gammaproteobacteria | Enterobacteriales | *Enterobacteriaceae* | *Erwinia* |
| Tryptophan | J69 | Bacteria | Proteobacteria | Gammaproteobacteria | Enterobacteriales | *Enterobacteriaceae* | *Erwinia* |
| Tryptophan | J70 | Bacteria | Proteobacteria | Gammaproteobacteria | Enterobacteriales | *Enterobacteriaceae* | *Erwinia* |
| Tryptophan | J71 | Bacteria | Proteobacteria | Gammaproteobacteria | Enterobacteriales | *Enterobacteriaceae* | *Serratia* |
| Tryptophan | J72 | Bacteria | Firmicutes | Bacilli | Bacillales | *Staphylococcaceae* | *Staphylococcus* |
| Tryptophan | J73 | Bacteria | Proteobacteria | Gammaproteobacteria | Pseudomonadales | *Pseudomonadaceae* | *Pseudomonas* |
| Tryptophan | J74 | Bacteria | Proteobacteria | Gammaproteobacteria | Enterobacteriales | *Enterobacteriaceae* | *Serratia* |
| Tryptophan | J75 | Bacteria | Proteobacteria | Gammaproteobacteria | Enterobacteriales | *Enterobacteriaceae* | *Erwinia* |
| Tryptophan | J76 | Bacteria | Firmicutes | Bacilli | Bacillales | *Bacillaceae* | *Bacillus* |
| Tryptophan | J77 | Bacteria | Proteobacteria | Gammaproteobacteria | Enterobacteriales | *Enterobacteriaceae* | *Serratia* |
| Tryptophan | J78 | Bacteria | Proteobacteria | Gammaproteobacteria | Enterobacteriales | *Enterobacteriaceae* | *Rahnella* |
| Tryptophan | J79 | Bacteria | Proteobacteria | Gammaproteobacteria | Enterobacteriales | *Enterobacteriaceae* | *Rahnella* |
| Tryptophan | J80 | Bacteria | Proteobacteria | Gammaproteobacteria | Pseudomonadales | *Pseudomonadaceae* | *Azomonas* |
| Tryptophan | SYC75 | Bacteria | Proteobacteria | Gammaproteobacteria | Enterobacteriales | *Enterobacteriaceae* | *Rahnella* |
| Tryptophan | SYC76 | Bacteria | Proteobacteria | Gammaproteobacteria | Enterobacteriales | *Enterobacteriaceae* | *Rahnella* |
| Tryptophan | SYC77 | Bacteria | Proteobacteria | Gammaproteobacteria | Enterobacteriales | *Enterobacteriaceae* | *Leclercia* |
| Tryptophan | SYC78 | Bacteria | Proteobacteria | Gammaproteobacteria | Enterobacteriales | *Enterobacteriaceae* | *Lelliottia* |
| Tryptophan | SYC79 | Bacteria | Proteobacteria | Gammaproteobacteria | Enterobacteriales | *Enterobacteriaceae* | *Rahnella* |
| Tryptophan | SYC80 | Bacteria | Proteobacteria | Gammaproteobacteria | Enterobacteriales | *Enterobacteriaceae* | *Serratia* |
| Tryptophan | SYC81 | Bacteria | Proteobacteria | Gammaproteobacteria | Enterobacteriales | *Enterobacteriaceae* | *Leclercia* |
| Tryptophan | SYC82 | Bacteria | Proteobacteria | Gammaproteobacteria | Enterobacteriales | *Enterobacteriaceae* | *Rahnella* |
| Tryptophan | SYC83 | Bacteria | Proteobacteria | Gammaproteobacteria | Enterobacteriales | *Enterobacteriaceae* | *Rahnella* |
| Tryptophan | SYC84 | Bacteria | Proteobacteria | Gammaproteobacteria | Enterobacteriales | *Enterobacteriaceae* | *Rahnella* |
| Tryptophan | SS76 | Bacteria | Proteobacteria | Betaproteobacteria | Burkholderiales | *Comamonadaceae* | *Comamonas* |
| Tryptophan | SS77 | Bacteria | Proteobacteria | Betaproteobacteria | Burkholderiales | *Comamonadaceae* | *Comamonas* |
| Tryptophan | SS78 | Bacteria | Proteobacteria | Gammaproteobacteria | Enterobacteriales | *Enterobacteriaceae* | *Rahnella* |
| Tryptophan | SS79 | Archaea | Crenarchaeota | Thermoprotei | Thermoproteales | *Thermoproteaceae* | *Caldivirga* |
| Tryptophan | SS80 | Bacteria | Proteobacteria | Gammaproteobacteria | Enterobacteriales | *Enterobacteriaceae* | *Rahnella* |
| Tryptophan | SS81 | Bacteria | Proteobacteria | Gammaproteobacteria | Enterobacteriales | *Enterobacteriaceae* | *Obesumbacterium* |
| Tryptophan | SS82 | Bacteria | Proteobacteria | Gammaproteobacteria | Enterobacteriales | *Enterobacteriaceae* | *Rahnella* |
| Tryptophan | SS83 | Bacteria | Proteobacteria | Gammaproteobacteria | Enterobacteriales | *Enterobacteriaceae* | *Rahnella* |
| Tryptophan | SS84 | Bacteria | Proteobacteria | Gammaproteobacteria | Enterobacteriales | *Enterobacteriaceae* | *Rahnella* |
| Tryptophan | SS85 | Bacteria | Proteobacteria | Gammaproteobacteria | Enterobacteriales | *Enterobacteriaceae* | *Lelliottia* |
