# Supplementary material for: Initial nitrogen enrichment conditions determines variations in nitrogen substrate utilization by heterotrophic bacterial isolates
Source: BMC Microbiol. 2017 Apr 4;17:87. doi: 10.1186/s12866-017-0993-7 (PMC5381026; doi:10.1186/s12866-017-0993-7)
Supplement: Supplementary file 2 — Partial bacterial 16S rRNA sequence data. Description of data: The partial 16S rRNA bacterial sequence information for all 266 bacterial isolates obtained in this study. (DOCX 94 kb) [file 12866_2017_993_MOESM2_ESM.docx]

**Partial bacterial 16S rRNA sequence data**

>J01

CCTTCCGCGATTGGATGAACCTAGGTGGGATTAGCTAGTTGGTGAGGTAATGGCTCACCAAGGCGACGATCCCTAGCTGTTCTGAGAGGATGATCAGCCACACTGGGACTGAGACACGGCCCAAACTCCTACGGGAGGCAGCAGTGGGGAATATTGCACAATGGGGGAAACCCTGATGCAGCCATGCCGCGTGTGTGAAGAAGGCCTTCGGGTTGTAAAGCACTTTCAGTAGGGAGGAAAGGGTGTAACTTAATACGTTATATCTGTGACGTTACCTACAGAAGAAGGACCGGCTAACTCCGTGCCAGCAGCCGCGGTAATACGGAGGGTCCGAGCGTTAATCGGAATTACTGGGCGTAAAGCGTGCGCAGGCGGTTTGTTAAGCGAGATGTGAAAGCCCTGGGCTCAACCTAGGAATAGCATTTCGAACTGGCGAACTAGAGTCTTGTAGAGGGGGGTAGAATTCCAGGTGTA

>J02

ACGGGTGAGTAATGTCTGGGAAACTGCCTGATGGAGGGGGATAACTACTGGAAACGGTAGCTAATACCGCATGACGTCTTCGGACCAAAGTGGGGGACCTTCGGGCCTCACGCCATCAGATGTGCCCAGATGGGATTAGCTAGTAGGTGGGGTAATGGCTCACCTAGGCGACGATCTCTAGCTGGTCTGAGAGGATGACCAGCCACACTGGAACTGAGACACGGTCCAGACTCCTACGGGAGGCAGCAGTGGGGAATATTGCACAATGGGCGCAAGCCTGATGCAGCCATGCCGCGTGTATGAAGAAGGCCTTCGGGTTGTAAAGTACTTTCAGCGAGGAGGAAGGCATTAAGGTTAATAACCTTAGTGATTGACGTTACTCGCAGAAGAAGCACCGGCTAACTCCGTGCCAGCAGCCGCGGTAATACGGAGGGTGCAAGCGTTAATCGGAATTACTGGGCGTAAAGCGCACGCAGGCGGTTGATTAAGTCAGATGTGAAATCCCCGAGCTTAACTTGGGAACTGCATTTGAAACTGGTCAGCTAGAGTCTTGTAGAGGGGGGTAGAATTCCAGGTGTAGCGGTGAAATGCGTAGAGATCTGGAGGAATACCGGTGGCGAAGGCGGCCCCCTGGACAAAGACTGACGCTCAGGTGCGAAAGCGTGGGG

>J03

GGTGTGTACAAGGCCCGGGAACGTATTCACCGTAGCATTCTGATCTACGATTACTAGCGATTCCGACTTCATGGAGTCGAGTTGCAGACTCCAATCCGGACTACGACATACTTTATGAGGTCCGCTTGCTCTCGCGAGTTCGCTTCTCTTTGTATATGCCATTGTAGCACGTGTGTAGCCCTACTCGTAAGGGCCATGATGACTTGACGTCATCCCCACCTTCCTCCGGTTTATCACCGGCAGTCTCCTTTGAGTTCCCGACATGACTCGCTGGCAACAAAGGATAAGGGTTGCGCTCGTTGCGGGACTTAACCCAACATTTCACAACACGAGCTGACGACAGCCATGCAGCACCTGTCTCAGAGTTCCCGAAGGCACTAAGCTATCTCTAGCAAATTCTCTGGATGTCAAGAGTAGGTAAGGTTCTTCGCGTTGCATCGAATTAAACCACATGC

>J04

GGAACGTATTCACCGTGGCATTCTGATCCACGATTACTAGCGATTCCGACTTCATGGAGTCGAGTTGCAGACTCCAATCCGGACTACGACGAGCTTTGTG

AGATTAGCTCCACCTCGCGGCTTTGCAACCCTCTGTACTCGCCATTGTAG

CACGTGTGTAGCCCTACTCGTAAGGGCCATGATGACTTGACGTCGTCCCC

ACCTTCCTCCGGTTTATCACCGGCAGTCTCCCTAGAGTTCCCACCATTAC

GTGCTGGCAAATAAGGATAGGGGTTGCGCTCGTTGCGGGACTTAACCCAA

CATTTCACAACACGAGCTGACGACAGCCATGCAGCACCTGTCTCAGAGTT

CCCGAAGGCACTAAGCTATCTCTAGCGAATTCTCTGGATGTCAAGAGTAG

GTAAGGTTCTTCGCGTTGCATCGAATTAAACCACATGCTCCACCGCTTGT

GCGGGCCCCCGTCAATTCATTTGAGTTTTAACCTTGCGGCCGTACTCCCC

AGGCGGTCTACTTAATGCGTTAGCTTGAGAGCCCAGTGTTCAAGACACCA

AACTCCGAGTAGACATCGTTTACGGCGTGGACTACCAGGGTATCTAATCC

>J05

TTCAACTCTTCTACGGGAGGCAGCAGTGGGGAATATTGGACAATGGGCGA

AAGCCTGATCCAGCCATGCCGCGTGTGTGAAGAAGGTCTTCGGATTGTAA

AGCACTTTAAGTTGGGAGGAAGGGCAGTTACCTAATACGTGACTGTCTTG

ACGTTACCGACAGAATAAGCACCGGCTAACTCTGTGCCAGCAGCCGCGGT

AATACAGAGGGTGCAAGCGTTAATCGGAATTACTGGGCGTAAAGCGCGCG

TAGGTGGTTTGTTAAGTTGAATGTGAAATCCCCGGGCTCAACCTGGGAAC

TGCATCCAAAACTGGCAAGCTAGAGTATGGTAGAGGGTAGTGGAATTTCC

TGTGTAGCGGTGAAATGCGTAGATATAGGAAGGAACACCAGTGGCGAAGG

CGACTACCTGGACTGATACTGACACTGAGGTGCGAAAGCGTGGG

>J06

TACGACGCGCTTTTTGGGATTCGCTCACTATCGCTAGCTTGCAACCCTCT

GTACGCGCCATTGTAGCACGTGTGTAGCCCTGGCCGTAAGGGCCATGATG

ACTTGACGTCATCCCCACCTTCCTCCGGTTTATCACCGGCAGTCTCCCTT

GAGTTCCCACCATTACGTGCTGGCAACAAAGGACAGGGGTTGCGCTCGTT

GCGGGACTTAACCCAACATCTCACGACACGAGCTGACGACAGCCATGCAG

CACCTGTGTTCTGATTCCCGAAGGCACTCACGCATCTCTGCAGGATTCCA

GACATGTCAAGGCCAGGTAAGGTTCTTCGCGTTGCATCGAATTAAACCAC

ATGCTCCACCGCTTGTGCGGGCCCCCGTCAATTCATTTGAGTTTTAACCT

>J07

TCACACTGGAACTGACCCTGGACCAACTCTTCTACGGGAGGCAGCAGTGG

GGAATATTGGACAATGGGCGAAAGCCTGATCCAGCCATGCCGCGTGTGTG

AAGAAGGTCTTCGGATTGTAAAGCACTTTAAGTTGGGAGGAAGGGCAGTT

ACCTAATACGTGACTGTCTTGACGTTACCGACAGA

>J08

TGTGTACAAGACCCGGGAACGTATTCACCGTGACATTCTGATCCACGATT

ACTAGCGATTCCGACTTCACGCAGTCGAGTTGCAGACTGCGATCCGGACT

CTTGACGTCATCCCCACCTTCCTCCGGTTTGTCACCGGCAGTCCCATTAG

AGTGCTCAACTAAATGTAGCAACTAATGGCAAGGGTTGCGCTCGTTGCGG

GACTTAACCCAACATCTCACGACACGAGCTGACGACAGCCATGCAGCACC

TGTGTTACGGCTCTCTTTCGAGCACGAAGCTATCTCTAGCGACTTCCGTA

CATGTCAAAGGTGGGTAAGGTTTTTCGCGTTGCATCGAATTAAACCACAT

CATCCACCGCTTGTGCGGGTCCCCGTCAATTCCTTTGAGTTTCAACCTTG

CGGCCGTACTCCCCAGGCGGTCAACTTCACGCGTTAGCTTCGTTACTGAG

>J09

GACCTTCGGGCCTTGCGCTATCAGATGAGCCTAGGTCGGATTAGCTAGTT

GGTGGGGTAATGGCTCACCAAGGCGACGATCCGTAACTGGTCTGAGAGGA

TGATCAGTCACACTGGAACTGAGACACGGTCCAGACTCCTACGGGAGGCA

GCAGTGGGGAATATTGGACAATGGGCGAAAGCCTGATCCAGCCATGCCGC

GTGTGTGAAGAAGGTCTTCGGATTGTAAAGCACTTTAAGTTGGGAGGAAG

GGCAGTAAGCGAATACCTTGCTGTTTTGACGTTACCGACAGAATAAGCAC

CGGCTAACTCTGTGCCAGCAGCCGCGGTAATACAGAGGGTGCAAGCGTTA

ATCGGAATTACTGGGCGTAAAGCGCGCGTAGGTGGTTTGTTAAGTTGAAT

GTGAAAGCCCCGGGCTCAACCTGGGGAACTGCATCCAAAACTGGCAAGCT

AGAGTACGGTAGAGGGTGGTGGAATTTCCTGTGTAGCGGTGAAATGCGTA

>J10

TCTTTTGCAGCCCACTCCCATGGTGTGACGGGCGGTGTGTACAAGGCCCG

GGAACGTATTCACCGTGGCATTCTGATCCACGATTACTAGCGATTCCGAC

TTCATGGAGTCGAGTTGCAGACTCCAATCCGGACTACGACGAGCTTTGTG

AGATTAGCTCCACCTCGCGGCTTTGCAACCCTCTGTACTCGCCATTGTAG

CACGTGTGTAGCCCTACTCGTAAGGGCCATGATGACTTGACGTCGTCCCC

ACCTTCCTCCGGTTTATCACCGGCAGTCTCCCTAGAGTTCCCACCATTAC

GTGCTGGCAAATAAGGATAGGGGTTGCGCTCGTTGCGGGACTTAACCCAA

CATTTCACAACACGAGCTGACGACAGCCATGCAGCACCTGTCTCAGAGTT

CCCGAAGGCACTAAGCTATCTCTAGCGAATTCTCTGGATGTCAAGAGTAG

GTAAGGTTCTTCGCGTTGCATCGAATTAAACCACATGCTCCACCGCTTGT

GCGGGCCCCCGTCAATTCATTTGAGTTTTAACCTTGCGGCCGTACTCCCC

AGGCGGTCTACTTAATGCGTTAGCTTGAGAGCCCAGTGTTCAAGACACCA

AACTCCGAGTAGACATCGTTTACGGCGTGGACTACCAGGGTATCTAATCC

TGTTTGCTCCCCACGCTTTCGTGCATGAGCGTCAGTCTTTGTCCAGGGGG

GCCGCCTTCGCC

>J11

GTGTGTACAAGGCCCGGGAACGTATTCACCGCAACATTCTGATTTGCGAT

TACTAGCGATTCCGACTTCACGGAGTCGAGTTGCAGACTCCGATCCGGAC

TACGACGCGCTTTTTGGGATTCGCTCACTATCGCTAGCTTGCAACCCTCT

GTACGCGCCATTGTAGCACGTGTGTAGCCCTGGCCGTAAGGGCCATGATG

ACTTGACGTCATCCCCACCTTCCTCCGGTTTATCACCGGCAGTCTCCCTT

GAGTTCCCACCATTACGTGCTGGCAACAAAGGACAGGGGTTGCGCTCGTT

GCGGGACTTAACCCAACATCTCACGACACGAGCTGACGACAGCCATGCAG

CACCTGTGTTCTGATTCCCGAAGGCACTCACGCATCTCTGCAGGATTCCA

GACATGTCAAGGCCAGGTAAGGTTCTTCGCGTTGCATCGAATTAAACCAC

ATGCTCCACCGCTTGTGCGGGCCCCCGTCAATTCATTTGAGTTTTAACCT

TGCGGCCGTACTCCCCAGGC

>J12

GGTGTGTACAAGGCCCGGGAACGTATTCACCGCGGCATGCTGATCCGCGA

TTACTAGCGATTCCAGCTTCACGCAGTCGAGTTGCAGACTGCGATCCGAA

CTGAGAACAGATTTGTGGGATTGGCTAAACCTTGCGGTCTCGCAGCCCTT

TGTTCTGTCCATTGTAGCACGTGTGTAGCCCAGGTCATAAGGGGCATGAT

GATTTGACGTCATCCCCACCTTCCTCCGGTTTGTCACCGGCAGTCACCTT

AGAGTGCCCAACTGAATGCTGGCAACTAAGATCAAGGGTTGCGCTCGTTG

CGGGACTTAACCCAACATCTCACGACACGAGCTGACGACAACCATGCACC

ACCTGTCACTCTGTCCCCGAAGGGAAAGCCCTATCTCTAGGGTTGTCAGA

GGATGTCAAGACCTGGTAAGGTTCTTCGCGTTGCTTCGAATTAAACCACA

TGCTCCACCGCTTGTGCGGGCCCCCGTCAATTCCTTTGAGTTTCAGTCTT

GCGACCGTACTCCCCAGGCGGAGTGCTTAATGCGTTAGCTGCAGCACTAA

GGGGCGGAAACCCCCTAACACTTAGCACTCATCGTTTACGGCGTGGACTA

CCAGGGTATCTAATCCTGTTCGCTCCCCACGCTTTCGCTCCTCAGCGTCA

>J13

TGTGTACAAGGCCCGGGAACGTATTCACCGCAACATTCTGATTTGCGATT

ACTAGCGATTCCGACTTCACGGAGTCGAGTTGCAGACTCCGATCCGGACT

ACGACGCGCTTTTTGGGATTCGCTCACTATCGCTAGCTTGCAGCCCTCTG

TACGCGCCATTGTAGCACGTGTGTAGCCCTGGCCGTAAGGGCCATGATGA

CTTGACGTCATCCCCACCTTCCTCCGGTTTATCACCGGCAGTCTCCCTTG

AGTTCCCACCATTACGTGCTGGCAACAAAGGACAGGGGTTGCGCTCGTTG

CGGGACTTAACCCAACATCTCACGACACGAGCTGACGACAGCCATGCAGC

ACCTGTGTTCTGATTCCCGAAGGCACTCCCGTATCTCTACAGGATTCCAG

ACATGTCAAGGCCAGGTAAGGTTCTTCGCGTTGCATCGAATTAAACCACA

TGCTCCACCGCTTGTGCGGG

>J14

ACTAGCGATTCCGACTTCACGGAGTCGAGTTGCAGACTCCGATCCGGACT

TACGCGCCATTGTAGCACGTGTGTAGCCCTGGCCGTAAGGGCCATGATGA

CTTGACGTCATCCCCACCTTCCTCCGGTTTATCACCGGCAGTCTCCCTTG

AGTTCCCACCATTACGTGCTGGCAACAAAGGACAGGGGTTGCGCTCGTTG

CGGGACTTAACCCAACATCTCACGACACGAGCTGACGACAGCCATGCAGC

ACCTGTGTTCTGATTCCCGAAGGCACTCCCGTATCTCTACAGGATTCCAG

ACATGTCAAGGCCAGGTAAGGTTCTTCGCGTTGCATCGAATTAAACCACA

>J15

TGTGTACAAGACCCGGGAACGTATTCACCGTGACATTCTGATCCACGATT

ACTAGCGATTCCGACTTCACGCAGTCGAGTTGCAGACTGCGATCCGGACT

ACGACTGGCTTTATGGGATTAGCTCCCCCTCGCGGGTTGGCAACCCTTTG

TACCAGCCATTGTATGACGTGTGTAGCCCCACCTATAAGGGCCATGAGGA

CTTGACGTCATCCCCACCTTCCTCCGGTTTGTCACCGGCAGTCCCATTAG

AGTGCTCAACTAAATGTAGCAACTAATGGCAAGGGTTGCGCTCGTTGCGG

GACTTAACCCAACATCTCACGACACGAGCTGACGACAGCCATGCAGCACC

TGTGTTACGGCTCTCTTTCGAGCACGAAGCTATCTCTAGCGACTTCCGTA

CATGTCAAAGGTGGGTAAGGTTTTTCGCGTTGCATCGAATTAAACCACAT

CATCCACCGCTTGTGCGGGTCCCCGTCAATTCCTTTGAGTTTCAACCTTG

CGGCCGTACTCCCCAGGCGGTCAACTTCACGCGTTAGCTTCGTTACTGAG

TCAGTTAAGACCCAAC

>J16

GTTTCGAAAGGAACGCTAATACCGCATACGTCCTACGGGAGAAAGCAGGG

GACCTTCGGGCCTTGCGCTATCAGATGAGCCTAGGTCGGATTAGCTAGTT

GGTGGGGTAATGGCTCACCAAGGCGACGATCCGTAACTGGTCTGAGAGGA

TGATCAGTCACACTGGAACTGAGACACGGTCCAGACTCCTACGGGAGGCA

GCAGTGGGGAATATTGGACAATGGGCGAAAGCCTGATCCAGCCATGCCGC

GTGTGTGAAGAAGGTCTTCGGATTGTAAAGCACTTTAAGTTGGGAGGAAG

GGCAGTAAGCGAATACCTTGCTGTTTTGACGTTACCGACAGAATAAGCAC

CGGCTAACTCTGTGCCAGCAGCCGCGGTAATACAGAGGGTGCAAGCGTTA

ATCGGAATTACTGGGCGTAAAGCGCGCGTAGGTGGTTTGTTAAGTTGAAT

GTGAAAGCCCCGGGCTCAACCTGGGGAACTGCATCCAAAACTGGCAAGCT

AGAGTACGGTAGAGGGTGGTGGAATTTCCTGTGTAGCGGTGAAATGCGTA

GATATA

>J17

GTGTGTACAAGGCCCGGGAACGTATTCACCGCGACATTCTGATTCGCGAT

TACTAGCGATTCCGACTTCACGCAGTCGAGTTGCAGACTGCGATCCGGAC

TACGATCGGTTTTGTGAGATTAGCTCCACCTCGCGGCTTGGCAACCCTCT

GTACCGACCATTGTAGCACGTGTGTAGCCCAGGCCGTAAGGGCCATGATG

ACTTGACGTCATCCCCACCTTCCTCCGGTTTGTCACCGGCAGTCTCCTTA

GAGTGCCCACCATAACGTGCTGGTAACTAAGGACAAGGGTTGCGCTCGTT

ACGGGACTTAACCCAACATCTCACGACACGAGCTGACGACAGCCATGCAG

CACCTGTGTCAGAGTTCCCGAAGGCACCAATCCATCTCTGGAAAAGTTCT

CTGC

>J18

AAGAGCTTGCTCTTCGATTCAGCGGCGGACGGGTGAGTAATGCCTAGGAA

TCTGCCTGGTAGTGGGGGACAACGTTTCGAAAGGAACGCTAATACCGCAT

ACGTCCTACGGGAGAAAGCAGGGGACCTTCGGGCCTTGCGCTATCAGATG

AGCCTAGGTCGGATTAGCTAGTTGGTGGGGTAATGGCTCACCAAGGCGAC

GATCCGTAACTGGTCTGAGAGGATGATCAGTCACACTGGAACTGAGACAC

GGTCCAGACTCCTACGGGAGGCAGCAGTGGGGAATATTGGACAATGGGCG

AAAGCCTGATCCAGCCATGCCGCGTGTGTGAAGAAGGTCTTCGGATTGTA

AAGCACTTTAAGTTGGGAGGAAGGGCAGTAAGCGAATACCTTGCTGTTTT

GACGTTACCGACAGAATAAGCACCGGCTAACTCTGTGCCAGCAGCCGCGG

TAATACAGAGGGTGCAAGCGTTAATCGGAATTACTGGGCGTAAAGCGCGC

GTAGGTGGTTTGTTAAGTTGGATGTGAAAGCCCCGGGCTCAACCTGGGAA

CTGCATCCAAAACTGGCAAGCTAGAGTACGGTAGAGGGTGGTGGAATTTC

CTGTGTAGCGGTGAAATGCGTAGATATAGGAAGGAACACCAGTGGCGAAG

GCGACCACCTGGACTGATACTGACACTGAGGTGCGAAAGCGTGGGGAGCA

AACAGGA

>J19

CTACTTCTTTTGCAACCCACTCCCATGGTGTGACGGGCGGTGTGTACAAG

GCCCGGGAACGTATTCACCGTAGCATTCTGATCTACGATTACTAGCGATT

CCGACTTCATGGAGTCGAGTTGCAGACTCCAATCCGGACTACGACGTACT

TTATGAGGTCCGCTTGCTCTCGCGAGGTCGCTTCTCTTTGTATACGCCAT

TGTAGCACGTGTGTAGCCCTACTCGTAAGGGCCATGATGACTTGACGTCA

TCCCCACCTTCCTCCAGTTTATCACTGGCAGTCTCCTTTGAGTTCCCGGC

CGAACCGCTGGCAACAAAGGATAAGGGTTGCGCTCGTTGCGGGACTTAAC

CCAACATTTCACAACACGAGCTGACGACAGCCATGCAGCACCTGTCTCAC

GGTTCCCGAAGGCACTAAAGCATCTCTGCTAAATTCCGTGGATGTCAAGA

GTAGGTAAGGTTCTTCGCGTTGCATCGAATTAAACCACATGCTCCACCGC

TTGTGCGGGCCCCCGTCAATTCATTTGAGTTTTAACCTTGCGGCCGTACT

CCCCAGGCGGTCGACTTAACGCGTTAGCTCCGGAAGCCACTCCTCAAGGG

AACAA

>J20

CAGGATCAACACTTCTACGGGAGGCAGCAGTGGGGAATATTGGACAATGG

GCGAAAGCCTGATCCAGCCATGCCGCGTGTGTGAAGAAGGTCTTCGGATT

GTAAAGCACTTTAAGTTGGGAGGAAGGGCAGTAAGCGAATACCTTGCTGT

TTTGACGTTACCGACAGAATAAGCACCGGCTAACTCTGTGCCAGCAGCCG

CGGTAATACAGAGGGTGCAAGCGTTAATCGGAATTACTGGGCGTAAAGCG

CGCGTAAGTGGTTTGTTAAGTTGAATGTGAAAGCCCCGGGCTCAACCTGG

GAACTGCATCCAAAACTGGCAAGCTAGAGTACGGTAGAGGGTGGTGGAAT

TTCCTGTGTAGCGGTGAAATGCGTAGATATAGGAAGGAACACCAGTGGCG

AAGG

>J21

AGGAACGCTAATACCGCATACGTCCTACGGGAGAAAGCAGGGGACCTTCG

GGCCTTGCGCTATCAGATGAGCCTAGGTCGGATTAGCTAGTTGGTGGGGT

AATGGCTCACCAAGGCGACGATCCGTAACTGGTCTGAGAGGATGATCAGT

CACACTGGAACTGAGACACGGTCCAGACTCCTACGGGAGGCAGCAGTGGG

GAATATTGGACAATGGGCGAAAGCCTGATCCAGCCATGCCGCGTGTGTGA

AGAAGGTCTTCGGATTGTAAAGCACTTTAAGTTGGGAGGAAGGGCAGTAA

GCGAATACCTTGTTGTTTTGACGTTACCGACAGAATAAGCACCGGCTAAC

TCTGTGCCAGCAGCCGCGGTAATACAGAGGGTGCAAGCGTTAATCGGAAT

TACTGGGCGTAAAGCGCGCGTAGGTGGTTTGTTAAGTTGAATGTGAAAGC

CCCGGGCTCAACCTGGGAACTGCATCCAAAACTGGCAAGCTAGAGTACGG

TAGAGGGTGGTGGAATTTCCTGTGTAGCGGTGAAATGCGTAGATATAGGA

AGGAACACCAGTGGCGAAGGCGACCACCTGGACTGATACTGACACTGAGG

TGCGAAAGCGTGGGG

>J22

ATAACTACTGGAAACGGTAGCTAATACCGCATAACGTCGCAAGACCAAAG

AGGGGGACCTTCGGGCCTCTTGCCATCAGATGTGCCCAGATGGGATTAGC

TAGTAGGTGGGGTAATGGCTCACCTAGGCGACGATCCCTAGCTGGTCTGA

GAGGATGACCAGCCACACTGGAACTGAGACACGGTCCAGACTCCTACGGG

AGGCAGCAGTGGGGAATATTGCACAATGGGCGCAAGCCTGATGCAGCCAT

GCCGCGTGTATGAAGAAGGCCTTCGGGTTGTAAAGTACTTTCAGCGAGGA

GGAAGGCATTAAGGTTAATAACCTTAGTGATTGACGTTACTCGCAGAAGA

AGCACCGGCTAACTCCGTGCCAGCAGCCGCGGTAATACGGAGGGTGCAAG

CGTTAATCGGAATTACTGGGCGTAAAGCGCACGCAGGCGGTCTGTCAAGT

CGGATGTGAAATCCCCGGGCTCAACCTGGGAACTGCATTCGAAACTGGCA

>J23

AAGAGCTTGCTCTTCGATTCAGCGGCGGACGGGAGAGTAATGCCTAGGAA

TCTGCCTGGTAGTGGGGGACAACGTTTCGAAAGGAACGCTAATACCGCAT

ACGTCCTACGGGAGAAAGCAGGGGACCTTCGGGCCTTGCGCTATCAGATG

AGCCTAGGTCGGATTAGCTAGTTGGTGGGGTAATGGCTCACCAAGGCGAC

GATCCGTAACTGGTCTGAGAGGATGATCAGTCACACTGGAACTGAGACAC

GGTCCAGACTCCTACGGGAGGCAGCAGTGGGGAATATTGGACAATGGGCG

AAAGCCTGATCCAGCCATGCCGCGTGTGTGAAGAAGGTCTTCGGATTGTA

AAGCACTTTAAGTTGGGAGGAAGGGCAGTAAGCGAATACCTTGCTGTTTT

GACGTTACCGACAGAATAAGCACCGGCTAACTCTGTGCCAGCAGCCGCGG

TAATACAGAGGGTGCAAGCGTTAATCGGAATTACTGGGCGTAAAGCGCGC

GTAGGTGGTTTGTTAAGTTGAATGTGAAAGCCCCGGGCTCAACCTGGGAA

CTGCATCCAAAACTGGCAAGCTAGAGTACGGTAGAGGGTGGTGGAATTTC

CTGTGTAGCGGTGAAATGCGTAGATATA

>J24

GAAAGCAGGGGACCTTCGGGCCTTGCGCTATCAGATGAACCTAGGTCGGA

TTAGCTAGTTGGTGGGGTAATGGCTCACCAAGGCGACGATCCGTAACTGG

TCTGAGAGGATGATCAGTCACACTGGAACTGAGACACGGTCCAGACTCCT

ACGGGAGGCAGCAGTGGGGAATATTGGACAATGGGCGAAAGCCTGATCCA

GCCATGCCGCGTGTGTGAAGAAGGTCTTCGGATTGTAAAGCACTTTAAGT

TGGGAGGAAGGGCAGTAAGCGAATACCTTGCTGTTTTGACGTTACCGACA

GAATAAGCACCGGCTAACTCTGTGCCAGCAGCCGCGGTAATACAGAGGGT

GCAAGCGTTAATCGGAATTACTGGGCGTAAAGCGCGCGTAGGTGGTTTGT

TAAGTTGGATGTGAAAGCCCCGGGCTCAACCTGGGAACTGCA

>J25

GGCCTTGCGCTATCAGATGAGCCTAGGTCGGATTAGCTAGTTGGTGGGGT

AATGGCTCACCAAGGCGACGATCCGTAACTGGTCTGAGAGGATGATCAGT

CACACTGGAACTGAGACACGGTCCAGACTCCTACGGGAGGCAGCAGTGGG

GAATATTGGACAATGGGCGAAAGCCTGATCCAGCCATGCCGCGTGTGTGA

AGAAGGTCTTCGGATTGTAAAGCACTTTAAGTTGGGAGGAAGGGCAGTAA

GCGAATACCTTGTTGTTTTGACGTTACCGACAGAATAAGCACCGGCTAAC

TCTGTGCCAGCAGCCGCGGTAATACAGAGGGTGCAAGCGTTAATCGGAAT

CCCGGGCTCAACCTGGGAACTGCATCCAAAACTGGCAAGCTAGAGTACGG

TAGAGGGTGGTGGAATTTCCTGTGTAGCGGTGAAATGCGTAGATATAGGA

AGGAACACCAGTGGCGAAGGCGACCACCTGGACTGATACTGACACTGAGG

>J26

AGGGGGACCTTCGGGCCTCTTGCCATCAGATGTGCCCAGATGGGATTAGC

TAGTAGGTGGGGTAATGGCTCACCTAGGCGACGATCCCTAGCTGGTCTGA

GAGGATGACCAGCCACACTGGAACTGAGACACGGTCCAGACTCCTACGGG

GCCGCGTGTATGAAGAAGGCCTTCGGGTTGTAAAGTACTTTCAGCGAGGA

GGAAGGCATTAAGGTTAATAACCTTAGTGATTGACGTTACTCGCAGAAGA

AGCACCGGCTAACTCCGTGCCAGCAGCCGCGGTAATACGGAGGGTGCAAG

CGTTAATCGGAATTACTGGGCGTAAAGCGCACGCAGGCGGTCTGTCAAGT

CGGATGTGAAATCCCCGGGCTCAACCTGGGAACTGCATTCGAAACTGGCA

>J27

TCTGCCTGGTAGTGGGGGACAACGTTTCGAAAGGAACGCTAATACCGCAT

ACGTCCTACGGGAGAAAGCAGGGGACCTTCGGGCCTTGCGCTATCAGATG

AGCCTAGGTCGGATTAGCTAGTTGGTGGGGTAATGGCTCACCAAGGCGAC

GATCCGTAACTGGTCTGAGAGGATGATCAGTCACACTGGAACTGAGACAC

GGTCCAGACTCCTACGGGAGGCAGCAGTGGGGAATATTGGACAATGGGCG

AAAGCCTGATCCAGCCATGCCGCGTGTGTGAAGAAGGTCTTCGGATTGTA

AAGCACTTTAAGTTGGGAGGAAGGGCAGTAAGCGAATACCTTGCTGTTTT

GACGTTACCGACAGAATAAGCACCGGCTAACTCTGTGCCAGCAGCCGCGG

TAATACAGAGGGTGCAAGCGTTAATCGGAATTACTGGGCGTAAAGCGCGC

GTAGGTGGTTTGTTAAGTTGAATGTGAAAGCCCCGGGCTCAACCTGGGAA

CTGCATCCAAAACTGGCAAGCTAGAGTACGGTAGAGGGT

>J28

ATGGGATTAGCTAGTAGGTGGGGTAATGGCTCACCTAGGCGACGATCCCT

AGCTGGTCTGAGAGGATGACCAGCCACACTGGAACTGAGACACGGTCCAC

ACTCCTACGGGAGGCAGCAGTGGGGAATATTGCACAATGGGCGCAAGCCT

GATGCAGCCATGCCGCGTGTATGAAGAAGGCCTTC

>J29

GCTAGTTGGTGGGGTAATGGCTCACCAAGGCGACGATCCCTAGCTGGTCT

GAGAGGATGATCAGCCACACTGGAACTGAGACACGGTCCAGACTCATACG

GGAGGCAGCAGTGGGGAATATTGCACAATGGGGGAAACCCTGATGCAGCC

ATGCCGCGTGTGTGAAGAAGGCCTTCGGGTTGTAAAGCACTTTCAGCGAG

GAGGGAAGGTTGATGCCTAATACCTATCAGCTGTGACGTTACTCGCACAA

GAAGCACCGGCTAACTCCGTGCCAGCAGCCGCGGTAATACGGAGGGTGCA

AGCGTTAATCGGAATTACTGGGCGT

>J30

TCGGAACGTGCCTAGTAGTGGGGGATAACTACTCGAAAGAGTAGCTAATA

CCGCATGAGATCTACGGATGAAAGCAGGGGATCGCAAGACCTTGTGCTAC

TAGAGCGGCTGATGGCAGATTAGGTAGTTGGTGGGATAAAAGCTTACCAA

GCCGACGATCTGTAGCTGGTCTGAGAGGACGATCAGCCACACTGGGACTG

AGACACGGCCCAGACTCCTACGGGAGGCAGCAGTGGGGAATTTTGGACAA

TGGGCGAAAGCCTGATCCAGCAATGCCGCGTGCAGGATGAAGGCCTTCGG

GTTGTAAACTGCTTTTGTACGGAACGAAAAGCTTTGGGTTAATACCCTGG

AGTCATGACGGTACCGTAAGAATAAGCACCGGCTAACTACGTGCCAGCAG

CCGCGGTAATACGTAGGGTGCAAGCGTTAATCGGAATTACTGGGCGTAAA

GCGTGCGCAGGCGGTTTTGTAAGACAGAGGTGAAATCCCCGGGCTCAACC

TGGGAACTGCCTTTGTGACTGCAAGGCTAGAGTACGGCAGAGGGGGGTGG

AATTCCGCGTGTAGCAGTGAAATGCGTAGATATGCGGAG

>J31

TGATCAGTCACACTGGAACTGAGACACGGTCCAGACTAATACGGGAGGCA

GCAGTGGGGAATATTGGACAATGGGCGAAAGCCTGATCCAGCCATGCCGC

GTGTGTGAAGAAGGTCTTCGGATTGTAAAGCACTTTAAGTTGGGAGGAAG

GGCAGTAAGCTAATACCTTGCTGTTTTGACGTTACCGACAGAATAAGCAC

CGGCTAACTCTGTGCCAGCAGCCGCGGTAATACAGAGGGTGCAAGCGTTA

ATCGGAATTACTGGGCGTAAAGCGCGCGTAGGTGGTTTGTTAAGTTGGAT

GTGAAAGCCCC

>J32

CCGGGAACGTATTCACCGCGACATTCTGATTCGCGATTACTAGCGATTCC

GACTTCACGCAGTCGAGTTGCAGACTGCGATCCGGACTACGATCGGTTTT

GTGAGATTAGCTCCACCTCGCGGCTTGGCAACCCTCTGTACCGACCATTG

TAGCACGTGTGTAGCCCAGGCCGTAAGGGCCATGATGACTTGACGTCATC

CCCACCTTCCTCCGGTTTGTCACCGGCAGTCTCCTTAGAGTGCCCACCAT

AACGTGCTGGTAACTAAGGACAAGGGTTGCGCTCGTTACGGGACTTAACC

CAACATCTCACGACACGAGCTGACGACAGCCATGCAGCACCTGTGTCAGA

GTTCCCGAAGGCACCAATCTATCTCTAGAAAGTTCTCTGCATGTCAAGGC

>J33

AGCCTAGGTCGGATTAGCTAGTTGGTGGGGTAATGGCTCACCAAGGCGAC

GATCCGTAACTGGTCTGAGAGGATGATCAGTCACACTGGAACTGAGACAC

GGTCCAGACTCCTACGGGAGGCAGCAGTGGGGAATATTGGACAATGGGCG

AAAGCCTGATCCAGCCATGCCGCGTGTGTGAAGAAGGTCTTCGGATTGTA

AAGCACTTTAAGTTGGGAGGAAGGGCAGTAAGCGAATACCTTGCTGTTTT

GACGTTACCGACAGAATAAGCACCGGCTAACTCTGTGCCAGCAGCCGCGG

TAATACAGAGGGTGCAAGCGTTAATCGGAATTACTGGGCGTAAAGCGCGC

GTAGGTGGTTTGTTAAGTTGAATGTGAAAGCCCCGGGCTCAACCTGGGAA

CTGCATCCAAAACTGGCAAGCTAGAGTACAGTAGAGGGTGGTGGAATTTC

CTGTGTAGCGGTGAAAATGCGTAGATATAGGAAGGAACACCAGTGGCGAA

GGCGACCACCTGGACTGATACTGACACTGAGGTGCGAAAGCG

>J34

CGGTTTTGTGAGATTAGCTCCACCTCGCGGCTTGGCAACCCTCTGTACCG

ACCATTGTAGCACGTGTGTAGCCCAGGCCGTAAGGGCCATGATGACTTGA

CGTCATCCCCACCTTCCTCCGGTTTGTCACCGGCAGTCTCCTTAGAGTGC

CCACCATAACGTGCTGGTAACTAAGGACAAGGGTTGCGCTCGTTACGGGA

CTTAACCCAACATCTCACGACACGAGCTGACGACAGCCATGCAGCACCTG

TGTCAGAGTTCCCGAAGGCACCAATCTATCTCTAGAAAGTTCTCTGCATG

TCAAGGCCTGGTAAGGTTCTTCGCGTTGCTTCGAATTAAACCACATGCTC

CACCGCTTGTGCGGGCCCCCGTCAATTCATTTGAGTTTTAACCTTGCGGC

CGTACTCCCCAGGCGGTCAACTTAATGCGTTAGCTGCGCCACTAAAATCT

CAAGGATTCCAACGGCTAGTTGACATCGTTTACGGCGTGGACTACCAGGG

TATCTAATCCTGTTTGCTCCCCCACGCTTT

>J35

GGGCCTCTTGCCATCAGATGTGCCCAGATGGGATTAGCTAGTAGGTGGGG

TAATGGCTCACCTAGGCGACGATCCCTAGCTGGTCTGAGAGGATGACCAG

CCACACTGGAACTGAGACACGGTCCAGACTCCTACGGGAGGCAGCAGTGG

GGAATATTGCACAATGGGCGCAAGCCTGATGCAGCCATGCCGCGTGTATG

AAGAAGGCCTTCGGGTTGTAAAGTACTTTCAGCGAGGAGGAAGGCATTGT

GGTTAATAACCACAGTGATTGACGTTACTCGCAGAAGAAGCACCGGCTAA

CTCCGTGCCAGCAGCCGCGGTAATACGGAGGGTGCAAGCGTTAATCGGAA

TTACTGGGCGTAAAGCGCACGCAGGCGGTCTGTCAAGTCGGATGTGAAAT

CCCCGGGCTCAACCTGGGAACTGCATTCGAAACTGGCAGGCTAGAGTCTT

GTAGAGGGGGGTAGAATTCCAGGTGTAGCGGTGAAATGCGTAGAG

>J36

TGGGGTAATGGCTCACCTAGGCGACGATCCCTAGCTGGTCTGAGAGGATG

ACCAGCCACACTGGAACTGAGACACGGTCCAGACTCCTACGGGAGGCAGC

AGTGGGGAATATTGCACAATGGGCGCAAGCCTGATGCAGCCATGCCGCGT

GTGTGAAGAAGGCCTTAGGGTTGTAAAGCACTTTCAGCGAGGAGGAAGGG

TTCAGTGTTTTTTTCTTTTTTTCATTGACGTTACTCGCAGAAGAAGCACC

GGCTAACTCCGTGCCAGCAGCCGCGGTAATACGGAGGGTGCAAGCGTTAA

TCGGAATTACTGGGCGTAAAGCGCACGCAGGCGGTTTGTTAAGTCAGATG

TGAAATCCCCGCGCTTAACGTGGGAACTGCATTTGAAACTGGCAAGCTAG

AGTCTTGTAGAGGGGGGTAGAATTCCAGGTGTAGCGGTGAAATGCGTAGA

GATCTGGAGGAATACCGGTGGCGAAGGCGGCCCCCTG

>J37

TGGGGTAATGGCTCACCTAGGCGACGATCCCTAGCTGGTCTGAGAGGATG

ACCAGCCACACTGGAACTGAGACACGGTCCAGACTCCTACGGGAGGCAGC

AGTGGGGAATATTGCACAATGGGCGCAAGCCTGATGCAGCCATGCCGCGT

GTGTGAAGAAGGCCTTAGGGTTGTAAAGCACTTTCAGCGAGGAGGAAGGG

TTCAGTGTTTTTTTCTTTTTTTCATTGACGTTACTCGCAGAAGAAGCACC

GGCTAACTCCGTGCCAGCAGCCGCGGTAATACGGAGGGTGCAAGCGTTAA

TCGGAATTACTGGGCGTAAAGCGCACGCAGGCGGTTTGTTAAGTCAGATG

TGAAATCCCCGCGCTTAACGTGGGAACTGCATTTGAAACTGGCAAGCTAG

AGTCTTGTAGAGGGGGGTAGAATTCCAGGTGTA

>J38

TACTAGCGATTCCGACTTCATGGAGTCGAGTTGCAGACTCCAATCCGGAC

TACGACGTACTTTATGAGGTCCGCTTGCTCTCGCGAGTTCGCTTCTCTTT

GTATACGCCATTGTAGCACGTGTGTAGCCCTACTCGTAAGGGCCATGATG

ACTTGACGTCATCCCCACCTTCCTCCGGTTTATCACCGGCAGTCTCCTTT

GAGTTCCCGACCGAATCGCTGGCAACAAAGGATAAGGGTTGCGCTCGTTG

CGGGACTTAACCCAACATTTCACAACACGAGCTGACGACAGCCATGCAGC

GGATGTCAAGAGTAGGTAAGGTTCTTCGCGTTGCATCGAATTAAACCACA

TGCTCCACCGCTTGTGCGGGCCCCCGTCAATTCT

>J39

TCTGGGAAACTGCCTGATGGAGGGGGATAACTACTGGAAACGGTAGCTAA

TACCGCATGACCTCGAAAGAGCAAAGTGGGGGATCTTCGGACCTCACGCC

ATCGGATGTGCCCAGATGGGATTAGCTAGTAGGTGAGGTAATGGCTCACC

TAGGCGACGATCCCTAGCTGGTCTGAGAGGATGACCAGCCACACTGGAAC

TGAGACACGGTCCAGACTCCTACGGGAGGCAGCAGTGGGGAATATTGCAC

AATGGGCGCAAGCCTGATGCAGCCATGCCGCGTGTGTGAAGAAGGCCTTA

GGGTTGTAAAGCACTTTCAGCGAGGAGGAAGGCATCACACTTAATACGTG

TGGTGATTGACGTTACTCGCAGAAGAAGCACCGGCTAACTCCGTGCCAGC

AGCCGCGGTAATACGGAGGGTGCAAGCGTTAATCGGAATTACTGGGCGTA

AAGCGCACGCAGGCGGTTTGTTAAGTCAGATGTGAAATCCCCGCGCTTAA

CGTGGGAACTGCATTTGAAACTGGCAAGCTAGAGTCTTGTAGAGGGGGGT

AGAATTCCAGGTGTAGCGGTGAAATGCGTAGAGATCTGGAGGAATACCGG

GGGGAGCAAACAGGATTA

>J40

ACACGTGGGTAACCTGCCTGTAAGACTGGGATAACTCCGGGAAACCGGAG

CTAATACCGGATAGTTCCTTGAACCGCATGGTTCAAGGATGAAAGACGGT

TTCGGCTGTCACTTACAGATGGACCCGCGGCGCATTAGCTAGTTGGTGAG

GTAACGGCTCACCAAGGCGACGATGCGTAGCCGACCTGAGAGGGTGATCG

GCCACACTGGGACTGAGACACGGCCCAGACTCCTACGGGAGGCAGCAGTA

GGGAATCTTCCGCAATGGACGAAAGTCTGACGGAGCAACGCCGCGTGAGT

GATGAAGGTTTTCGGATCGTAAAGCTCTGTTGTTAGGGAAGAACAAGTGC

AAGAGTAACTGCTTGCACCTTGACGGTACCTAACCAGAAAGCCACGGCTA

ACTACGTGCCAGCAGCCGCGGTAATACGTAGGTGGCAAGCGTTGTCCGGA

ATTATTGGGCGTAAAGGGCTCGCAGGCGGTTTCTTAAGTCTGATGTGAAA

GCCCCCGGCTCAACCGGGGAGGGTCATTGGAAACTGGGAAACTTGAGTGC

AGAAGAGGA

>J41

GGATAACAGTTGGAAACGACTGCTAATACCGCATACGCCCTACGGGGGAA

AGGAGGGGACCTTCGGGCCTTTCGCGATTGGATGAACCCAGGTGGGATTA

GCTAGTTGGTGGGGTAATGGCTCACCAAGGCGACGATCCCTAGCTGGTCT

GAGAGGATGATCAGCCACACTGGAACTGAGACACGGTCCAGACTCATACG

GGAGGCAGCAGTGGGGAATATTGCACAATGGGGGAAACCCTGATGCAGCC

ATGCCGCGTGTGTGAAGAAGGCCTTCGGGTTGTAAAGCACTTTCAGCGAG

GAGGGAAGGTTGATGCCTAATACCTATCAGCTGTGACGTTACTCGCACAA

GAAGCACCGGCTAACTCCGTGCCAGCAGCCGCGGTAATACGGAGGGTGCA

AGCGTTAATCGGAATTACTGGGCGT

>J42

CGGTAACAGGTCTTCGGATGCTGACGAGTGGCGAACGGGTGAGTAATACA

TCGGAACGTGCCTAGTAGTGGGGGATAACTACTCGAAAGAGTAGCTAATA

CCGCATGAGATCTACGGATGAAAGCAGGGGATCGCAAGACCTTGTGCTAC

TAGAGCGGCTGATGGCAGATTAGGTAGTTGGTGGGATAAAAGCTTACCAA

GCCGACGATCTGTAGCTGGTCTGAGAGGACGATCAGCCACACTGGGACTG

AGACACGGCCCAGACTCCTACGGGAGGCAGCAGTGGGGAATTTTGGACAA

TGGGCGAAAGCCTGATCCAGCAATGCCGCGTGCAGGATGAAGGCCTTCGG

GTTGTAAACTGCTTTTGTACGGAACGAAAAGCTTTGGGTTAATACCCTGG

AGTCATGACGGTACCGTAAGAATAAGCACCGGCTAACTACGTGCCAGCAG

CCGCGGTAATACGTAGGGTGCAAGCGTTAATCGGAATTACTGGGCGTAAA

GCGTGCGCAGGCGGTTTTGTAAGACAGAGGTGAAATCCCCGGGCTCAACC

TGGGAACTGCCTTTGTGACTGCAAGGCTAGAGTACGGCAGAGGGGGGTGG

AATTCCGCGTGTAGCAGTGAAATGCGTAGATATGCGGAGGAACACCGATG

GCGAAGGCAACCCCCTGGGCCTGTACTGACGCTCATGCACGAAAGCGTGG

GGAGCAAACAGGA

>J43

GACCTTCGGGCCTTGCGCTATCAGATGAGCCTAGGTCGGATTAGCTAGTT

GGTGGGGTAATGGCTCACCAAGGCGACGATCCGTAACTGGTCTGAGAGGA

TGATCAGTCACACTGGAACTGAGACACGGTCCAGACTAATACGGGAGGCA

GCAGTGGGGAATATTGGACAATGGGCGAAAGCCTGATCCAGCCATGCCGC

GTGTGTGAAGAAGGTCTTCGGATTGTAAAGCACTTTAAGTTGGGAGGAAG

GGCAGTAAGCTAATACCTTGCTGTTTTGACGTTACCGACAGAATAAGCAC

CGGCTAACTCTGTGCCAGCAGCCGCGGTAATACAGAGGGTGCAAGCGTTA

ATCGGAATTACTGGGCGTAAAGCGCGCGTAGGTGGTTTGTTAAGTTGGAT

GTGAAAGCCCC

>J44

CCGGGAACGTATTCACCGCGACATTCTGATTCGCGATTACTAGCGATTCC

GACTTCACGCAGTCGAGTTGCAGACTGCGATCCGGACTACGATCGGTTTT

GTGAGATTAGCTCCACCTCGCGGCTTGGCAACCCTCTGTACCGACCATTG

TAGCACGTGTGTAGCCCAGGCCGTAAGGGCCATGATGACTTGACGTCATC

CCCACCTTCCTCCGGTTTGTCACCGGCAGTCTCCTTAGAGTGCCCACCAT

AACGTGCTGGTAACTAAGGACAAGGGTTGCGCTCGTTACGGGACTTAACC

CAACATCTCACGACACGAGCTGACGACAGCCATGCAGCACCTGTGTCAGA

GTTCCCGAAGGCACCAATCTATCTCTAGAAAGTTCTCTGCATGTCAAGGC

CTGGTAAGGTTCTTCGCGTTGCTTCGAATTAAACCACATGCTCCACCCGC

TTG

>J45

AAGAGCTTGCTCTTCGATTCAGCGGCGGACGGGAGAGTAATGCCTAGGAA

TCTGCCTGGTAGTGGGGGACAACGTTTCGAAAGGAACGCTAATACCGCAT

ACGTCCTACGGGAGAAAGCAGGGGACCTTCGGGCCTTGCGCTATCAGATG

AGCCTAGGTCGGATTAGCTAGTTGGTGGGGTAATGGCTCACCAAGGCGAC

GATCCGTAACTGGTCTGAGAGGATGATCAGTCACACTGGAACTGAGACAC

GGTCCAGACTCCTACGGGAGGCAGCAGTGGGGAATATTGGACAATGGGCG

AAAGCCTGATCCAGCCATGCCGCGTGTGTGAAGAAGGTCTTCGGATTGTA

AAGCACTTTAAGTTGGGAGGAAGGGCAGTAAGCGAATACCTTGCTGTTTT

GACGTTACCGACAGAATAAGCACCGGCTAACTCTGTGCCAGCAGCCGCGG

TAATACAGAGGGTGCAAGCGTTAATCGGAATTACTGGGCGTAAAGCGCGC

GTAGGTGGTTTGTTAAGTTGAATGTGAAAGCCCCGGGCTCAACCTGGGAA

CTGCATCCAAAACTGGCAAGCTAGAGTACAGTAGAGGGTGGTGGAATTTC

CTGTGTAGCGGTGAAAATGCGTAGATATAGGAAGGAACACCAGTGGCGAA

GGCGACCACCTGGACTGATACTGACACTGAGGTGCGAAAGCG

>J46

ACAAGGCCCGGGAACGTATTCACCGCGACATTCTGATTCGCGATTACTAG

CGATTCCGACTTCACGCAGTCGAGTTGCAGACTGCGATCCGGACTACGAT

CGGTTTTGTGAGATTAGCTCCACCTCGCGGCTTGGCAACCCTCTGTACCG

ACCATTGTAGCACGTGTGTAGCCCAGGCCGTAAGGGCCATGATGACTTGA

CGTCATCCCCACCTTCCTCCGGTTTGTCACCGGCAGTCTCCTTAGAGTGC

CCACCATAACGTGCTGGTAACTAAGGACAAGGGTTGCGCTCGTTACGGGA

CTTAACCCAACATCTCACGACACGAGCTGACGACAGCCATGCAGCACCTG

TGTCAGAGTTCCCGAAGGCACCAATCTATCTCTAGAAAGTTCTCTGCATG

TCAAGGCCTGGTAAGGTTCTTCGCGTTGCTTCGAATTAAACCACATGCTC

CACCGCTTGTGCGGGCCCCCGTCAATTCATTTGAGTTTTAACCTTGCGGC

CGTACTCCCCAGGCGGTCAACTTAATGCGTTAGCTGCGCCACTAAAATCT

CAAGGATTCCAACGGCTAGTTGACATCGTTTACGGCGTGGACTACCAGGG

TATCTAATCCTGTTTGCTCCCCCACGCTTT

>J47

GGGTGAGTAATGTCTGGGAAACTGCCTGATGGAGGGGGATAACTACTGGA

AACGGTAGCTAATACCGCATAACGTCGCAAGACCAAAGAGGGGGACCTTC

GGGCCTCTTGCCATCAGATGTGCCCAGATGGGATTAGCTAGTAGGTGGGG

TAATGGCTCACCTAGGCGACGATCCCTAGCTGGTCTGAGAGGATGACCAG

CCACACTGGAACTGAGACACGGTCCAGACTCCTACGGGAGGCAGCAGTGG

GGAATATTGCACAATGGGCGCAAGCCTGATGCAGCCATGCCGCGTGTATG

AAGAAGGCCTTCGGGTTGTAAAGTACTTTCAGCGAGGAGGAAGGCATTGT

GGTTAATAACCACAGTGATTGACGTTACTCGCAGAAGAAGCACCGGCTAA

CTCCGTGCCAGCAGCCGCGGTAATACGGAGGGTGCAAGCGTTAATCGGAA

TTACTGGGCGTAAAGCGCACGCAGGCGGTCTGTCAAGTCGGATGTGAAAT

CCCCGGGCTCAACCTGGGAACTGCATTCGAAACTGGCAGGCTAGAGTCTT

GTAGAGGGGGGTAGAATTCCAGGTGTAGCGGTGAAATGCGTAGAGATCTG

GAGGAATACCGGTGGCGAAGGCGGCCCCCTGGACAAAGACTGACGCTCAG

GTGCGAAAGCG

>J48

CTGGAAACGGTAGCTAATACCGCATAACGTCTACGGACCAAAGTGGGGGA

CCTTCGGGCCTCACGCCATCAGATGTGCCCAGATGGGATTAGCTAGTAGG

TGGGGTAATGGCTCACCTAGGCGACGATCCCTAGCTGGTCTGAGAGGATG

ACCAGCCACACTGGAACTGAGACACGGTCCAGACTCCTACGGGAGGCAGC

AGTGGGGAATATTGCACAATGGGCGCAAGCCTGATGCAGCCATGCCGCGT

GTGTGAAGAAGGCCTTAGGGTTGTAAAGCACTTTCAGCGAGGAGGAAGGG

TTCAGTGTTTTTTTCTTTTTTTCATTGACGTTACTCGCAGAAGAAGCACC

GGCTAACTCCGTGCCAGCAGCCGCGGTAATACGGAGGGTGCAAGCGTTAA

TCGGAATTACTGGGCGTAAAGCGCACGCAGGCGGTTTGTTAAGTCAGATG

TGAAATCCCCGCGCTTAACGTGGGAACTGCATTTGAAACTGGCAAGCTAG

AGTCTTGTAGAGGGGGGTAGAATTCCAGGTGTAGCGGTGAAATGCGTAGA

GATCTGGAGGAATACCGGTGGCGAAGGCGGCCCCCTGGACAAAGACTGAC

GCTC

>J49

CCTTCGGGCCTCACGCCATCAGATGTGCCCAGATGGGATTAGCTAGTAGG

TGGGGTAATGGCTCACCTAGGCGACGATCCCTAGCTGGTCTGAGAGGATG

ACCAGCCACACTGGAACTGAGACACGGTCCAGACTCCTACGGGAGGCAGC

AGTGGGGAATATTGCACAATGGGCGCAAGCCTGATGCAGCCATGCCGCGT

GTGTGAAGAAGGCCTTAGGGTTGTAAAGCACTTTCAGCGAGGAGGAAGGG

TTCAGTGTTTTTTTCTTTTTTTCATTGACGTTACTCGCAGAAGAAGCACC

GGCTAACTCCGTGCCAGCAGCCGCGGTAATACGGAGGGTGCAAGCGTTAA

TCGGAATTACTGGGCGTAAAGCGCACGCAGGCGGTTTGTTAAGTCAGATG

TGAAATCCCCGCGCTTAACGTGGGAACTGCATTTGAAACTGGCAAGCTAG

AGTCTTGTAGAGGGGGGTAGAATTCCAGGTGTAGCGGTGAAATGCGTAGA

>J50

GTGTGTACAAGGCCCGGGAACGTATTCACCGTAGCATTCTGATCTACGAT

TACTAGCGATTCCGACTTCATGGAGTCGAGTTGCAGACTCCAATCCGGAC

TACGACGTACTTTATGAGGTCCGCTTGCTCTCGCGAGTTCGCTTCTCTTT

GTATACGCCATTGTAGCACGTGTGTAGCCCTACTCGTAAGGGCCATGATG

ACTTGACGTCATCCCCACCTTCCTCCGGTTTATCACCGGCAGTCTCCTTT

GAGTTCCCGACCGAATCGCTGGCAACAAAGGATAAGGGTTGCGCTCGTTG

CGGGACTTAACCCAACATTTCACAACACGAGCTGACGACAGCCATGCAGC

ACCTGTCTCAGAGTTCCCGAAGGCACTAAGCTATCTCTAGCGAATTCTCT

GGATGTCAAGAGTAGGTAAGGTTCTTCGCGTTGCATCGAATTAAACCACA

TGCTCCACCGCTTGTGCGGGCCCCCGTCAATTCATTTGAGTT

>J51

GCGGAAGTAGCTTGCTACTTTGCCGGCGAGCGGCGGACGGGTGAGTAATG

TCTGGGAAACTGCCTGATGGAGGGGGATAACTACTGGAAACGGTAGCTAA

TACCGCATGACCTCGAAAGAGCAAAGTGGGGGATCTTCGGACCTCACGCC

ATCGGATGTGCCCAGATGGGATTAGCTAGTAGGTGAGGTAATGGCTCACC

TAGGCGACGATCCCTAGCTGGTCTGAGAGGATGACCAGCCACACTGGAAC

TGAGACACGGTCCAGACTCCTACGGGAGGCAGCAGTGGGGAATATTGCAC

AATGGGCGCAAGCCTGATGCAGCCATGCCGCGTGTGTGAAGAAGGCCTTA

GGGTTGTAAAGCACTTTCAGCGAGGAGGAAGGCATCACACTTAATACGTG

TGGTGATTGACGTTACTCGCAGAAGAAGCACCGGCTAACTCCGTGCCAGC

AGCCGCGGTAATACGGAGGGTGCAAGCGTTAATCGGAATTACTGGGCGTA

AAGCGCACGCAGGCGGTTTGTTAAGTCAGATGTGAAATCCCCGCGCTTAA

CGTGGGAACTGCATTTGAAACTGGCAAGCTAGAGTCTTGTAGAGGGGGGT

AGAATTCCAGGTGTAGCGGTGAAATGCGTAGAGATCTGGAGGAATACCGG

TGGCGAAGGCGGCCCCCTGGACAAAGACTGACGCTCAGGTGCGAAAGCGT

GGGGAGCAAACAGGATTA

>J52

CTGGAAACGGTAGCTAATACCGCATAACGTCGCAAGACCAAAGTGGGGGA

CCTTCGGGCCTCATGCCATCAGATGTGCCCAGATGGGATTAGCTAGTAGG

TGGGGTAATGGCTCACCTAGGCGACGATCCCTAGCTGGTCTGAGAGGATG

ACCAGCCACACTGGAACTGAGACACGGTCCAGACTCCTACGGGAGGCAGC

AGTGGGGAATATTGCACAATGGGCGCAAGCCTGATGCAGCCATGCCGCGT

GTATGAAGAAGGCCTTCGGGTTGTAAAGTACTTTCAGCGAGGAGGAAGGC

GTTAAGGTTAATAACTTTAGTGATTGACGTTACTCGCAGAAGAAGCACCG

GCTAACTCCGTGCCAGCAGCCGCGGTAATACGGAGGGTGCAAGCGTTAAT

CGGAATTACTGGGCGTAAAGCGCACGCAGGCGGTTTGTTAAGTCAGATGT

GAAATCCCCGGGCTCAACCTGGGAACTGCATTTGAAACTGGCAAGCTTGA

GTCTTGTAGAGGGGGGTAGAATTCC

>J53

TGTGTACAAGGCCCGGGAACGTATTCACCGTAGCATTCTGATCTACGATT

ACTAGCGATTCCGACTTCATGGAGTCGAGTTGCAGACTCCAATCCGGACT

ACGACGTACTTTATGAGGTCCGCTTGCTCTCGCGAGTTCGCTTCTCTTTG

TATACGCCATTGTAGCACGTGTGTAGCCCTACTCGTAAGGGCCATGATGA

CTTGACGTCATCCCCACCTTCCTCCGGTTTATCACCGGCAGTCTCCTTTG

AGTTCCCGACCGAATCGCTGGCAACAAAGGATAAGGGTTGCGCTCGTTGC

GGGACTTAACCCAACATTTCACAACACGAGCTGACGACAGCCATGCAGCA

CCTGTCTCAGAGTTCCCGAAGGCACTAAGCTATCTCTAGCGAATTCTCTG

GATGTCAAGAGTAGGTAAGGTTCTTCGCGTTGCATCGAATTAAACCACAT

GCTCCACCGCTTGTGCGGGCCCCCGTCAATTCATTTGAGTTTTAACCTTG

CGGCCGTACTCCCCAGGCGGTCGATTTAACGCGTTAGCTCCGGAAGCCAC

GCCTCAAGGG

>J54

AGTAATGTCTGGGAAACTGCCTGATGGAGGGGGATAACTACTGGAAACGG

TAGCTAATACCGCATAACGTCTACGGACCAAAGTGGGGGACCTTCGGGCC

TCACGCCATCAGATGTGCCCAGATGGGATTAGCTAGTAGGTGGGGTAATG

GCTCACCTAGGCGACGATCCCTAGCTGGTCTGAGAGGATGACCAGCCACA

CTGGAACTGAGACACGGTCCAGACTCCTACGGGAGGCAGCAGTGGGGAAT

ATTGCACAATGGGCGCAAGCCTGATGCAGCCATGCCGCGTGTGTGAAGAA

GGCCTTAGGGTTGTAAAGCACTTTCAGCGAGGAGGAAGGGTTCAGTGTTA

ATAGCACTGTTCATTGACGTTACTCGCAGAAGAAGCACCGGCTAACTCCG

TGCCAGCAGCCGCGGTAATACGGAGGGTGCAAGCGTTAATCGGAATTACT

GGGCGTAAAGCGCACGCAGGCGGTTTGTTAAGTCAGATGTGAAATCCCCG

CGCTTAACGTGGGAACTGCATTTGAAACTGGCAAGCTAGAGTCTTGTAGA

GGGGGGTAGAATTCCAGGTGTAGCGGTGAAATGCGTAGAGATCTGG

>J55

GTGTGTACAAGGCCCGGGAACGTATTCACCGTAGCATTCTGATCTACGAT

TACTAGCGATTCCGACTTCATGGAGTCGAGTTGCAGACTCCAATCCGGAC

TACGACGTACTTTATGAGGTCCGCTTGCTCTCGCGAGGTCGCTTCTCTTT

GTATACGCCATTGTAGCACGTGTGTAGCCCTACTCGTAAGGGCCATGATG

ACTTGACGTCATCCCCACCTTCCTCCAGTTTATCACTGGCAGTCTCCTTT

GAGTTCCCGGCCGAACCGCTGGCAACAAAGGATAAGGGTTGCGCTCGTTG

CGGGACTTAACCCAACATTTCACAACACGAGCTGACGACAGCCATGCAGC

ACCTGTCTCACAGTTCCCGAAGGCACCAAAGCATCTCTGCTAAATTCTGT

GGATGTCAAGAGTAGGTAAGGTTCTTCGCGTTGCATCGAATTAAACCACA

TGCTCCACCGCTTGTGCGGGCCCCCGTCAATTCATTTGAGTTTTAACCTT

GCGGCCGTACTCCCCAGGCGGTCGACTTAACGCGTTAGCTCCGGAAGCCA

CTCCTCAAGGGAACAACCTCCAAGTCGACATCGTTTACGGCGTGGACTAC

CAGGGTATCTAATCCTGTTTGCTCCCCACGCTTTCGCAC

>J56

CACAAAGTGGTAAGCGCCCTCCCGAAGGTTAAGCAACCTACTTCTTTTGC

AACCCACTCCCATGGTGTGACGGGCGGTGTGTACAAGGCCCGGGAACGTA

TTCACCGTAGCATTCTGATCTACGATTACTAGCGATTCCGACTTCATGGA

GTCGAGTTGCAGACTCCAATCCGGACTACGACATACTTTATGAGGTCCGC

TTGCTCTCGCGAGTTCGCTTCTCTTTGTATATGCCATTGTAGCACGTGTG

TAGCCCTACTCGTAAGGGCCATGATGACTTGACGTCATCCCCACCTTCCT

CCGGTTTATCACCGGCAGTCTCCTTTGAGTTCCCACCATTACGTGCTGGC

AACAAAGGATAAGGGTTGCGCTCGTTGCGGGACTTAACCCAACATTTCAC

AACACGAGCTGACGACAGCCATGCAGCACCTGTCTCACGGTTCCCGAAGG

CACTAAGCCATCTCTGGCGAATTCCGTGGATGTCAAGAGTAGGTAAGGTT

CTTCGCGTTGCATCGAATTAAACCACATGCTCCACCGCTTGTGCGGGCCC

CCGTCAATTCATTTGAGTTTTAACCTTGCGGCCGTACTCCCCAGGCGGTC

GACTTAACGCGTTAGCTCCGGAAGCCACGCCTCAAGGGCACAACCTCCAA

GTCGACATCGTTTAC

>J57

ACGGTAGCTAATACCGCATAACCTCGTAAGAGCAAAGTGGGGGACCTTCG

GGCCTCACGCTATCGGATGTGCCCAGATGGGATTAGCTAGTAGGTGGGGT

AATGGCTCACCTAGGCGACGATCCCTAGCTGGTCTGAGAGGATGACCAGC

CACACTGGAACTGAGACACGGTCCACACTCCTACGGGAGGCAGCAGTGGG

GAATATTGCACAATGGGCGCAAGCCTGATGCAGCCATGCCGCGTGTATGA

AGAAGGCCTTCGGGTTGTAAAGTACTTTCAGCGGGGAGGAAGGCGATGAG

GTTAATAACCTTGTCGATTGACGTTACCCGCAGAAGAAGCACCGGCTAAC

TCCGTGCCAGCAGCCGCGGTAATACGGAGGGTGCAAGCGTTAATCGGAAT

TACTGGGCGTAAAGCGCACGCAGG

>J58

TCTTTTGCAACCCACTCCCATGGTGTGACGGGCGGTGTGTACAAGGCCCG

GGAACGTATTCACCGTAGCATTCTGATCTACGATTACTAGCGATTCCGAC

TTCACGGAGTCGAGTTGCAGACTCCGATCCGGACTACGACGCACTTTATG

AGGTCCGCTTGCTCTCGCGAGTTCGCTTCTCTTTGTATGCGCCATTGTAG

CACGTGTGTAGCCCTGGCCGTAAGGGCCATGATGACTTGACGTCATCCCC

ACCTTCCTCCGGTTTATCACCGGCAGTCTCCTTTGAGTTCCCGACCGAAT

CGCTGGCAACAAAGGATAAGGGTTGCGCTCGTTGCGGGACTTAACCCAAC

ATTTCACAACACGAGCTGACGACAGCCATGCAGCACCTGTCTCACGGTTC

CCGAAGGCACTAAGGCATCTCTGCCGAATTCCGTGGATGTCAAGGCCAGG

TAAGGTTCTTCGCGTTGCATCGAATTAAACCACATGCTCCACCGCTTGTG

CGGGCCCCCGTCAATTCATTTGAGTTTTAACCTTGCGGCCGTACTCCCCA

GGCGGTCGACTTAACGCGTTAGCTCCGGAAGCCACGCCTCAAGGGCACAA

>J59

AAGGAACGCTAATACCGCATACGTCCTACGGGAGAAAGCAGGGGACCTTC

GGGCCTTGCGCTATCAGATGAGCCTAGGTCGGATTAGCTAGTTGGTGGGG

TAATGGCTCACCAAGGCGACGATCCGTAACTGGTCTGAGAGGATGATCAG

TCACACTGGAACTGAGACACGGTCCAGACTCCTACGGGAGGCAGCAGTGG

GGAATATTGGACAATGGGCGAAAGCCTGATCCAGCCATGCCGCGTGTGTG

AAGAAGGTCTTCGGATTGTAAAGCACTTTAAGTTGGGAGGAAGGGCAGTA

AGTTAATACCTTGCTTTTTTTTACGTTACCGACAGAATAAGCACCGGCTA

ACTCTGTGCCAGCAGCCGCGGTAATACAGAGGGTGCAAAGCGTTAATCGG

AATTACTGGGCGTAAAGCGCGCGTAGGTGGTTTGTTAAGTTGGATGTGAA

AGCCCCGGG

>J60

TGTGTACAAGGCCCGGGAACGTATTCACCGTAGCATTCTGATCTACGATT

ACTAGCGATTCCGACTTCATGGAGTCGAGTTGCAGACTCCAATCCGGACT

ACGACGTACTTTATGAGGTCCGCTTGCTCTCGCGAGTTCGCTTCTCTTTG

TATACGCCATTGTAGCACGTGTGTAGCCCTACTCGTAAGGGCCATGATGA

CTTGACGTCATCCCCACCTTCCTCCGGTTTATCACCGGCAGTCTCCTTTG

AGTTCCCGACCGAATCGCTGGCAACAAAGGATAAGGGTTGCGCTCGTTGC

GGGACTTAACCCAACATTTCACAACACGAGCTGACGACAGCCATGCAACA

CCTGTCTCAGAGTTCCCGAAGGCACCAATCCATCTCTGGAAAGTTCTCTG

GATGTCAAGAGTAGGTAAGGTTCTTCGCGTTGCATCGAATTAAACCACAT

GCTCCACCGCTTGTGCGGGCCCCCGTCAATTCATTTGAGT

>J61

CCGGGAAACCGGGGCTAATACCGGATAACATTTTGAACTGCATGGTTCGA

AATTGAAAGGCGGCTTCGGCTGTCACTTATGGATGGACCCGCGTCGCATT

AGCTAGTTGGTGAGGTAACGGCTCACCAAGGCAACGATGCGTAGCCGACC

TGAGAGGGTGATCGGCCACACTGGGACTGAGACACGGCCCAGACTCCTAC

GGGAGGCAGCAGTAGGGAATCTTCCGCAATGGACGAAAGTCTGACGGAGC

AACGCCGCGTGAGTGATGAAGGCTTTCGGGTCGTAAAACTCTGTTGTTAG

GGAAGAACAAGTGCTAGTTGAATAAGCTGGCACCTTGACGGTACCTAACC

AGAAAGCCACGGCTAACTACGTGCCAGCAGCCGCGGTAATACGTAGGTGG

CAAGCGTTATCCGGAATTATTGGGCGTAAAGCGCGCGCAGGTGGTTTCTT

AAGTCTGATGTGAAAGCCCACGGCTCAACCGTGGAGGGTCATTGGAAACT

GGGAGACTTGAGTGCAGAAGAGGAAAGTGGAATTCCATGTGTAGCGGTGA

AATGCGTAGAGATATGGAGGAACACCAGTGGCGAAGGCGACTTTCTGGTC

TGTAACTGACACTGAGGCGCGAAAGCGTGGGGAGCAAACAGGAT

>J62

GTAACACGTGGATAACCTACCTATAAGACTGGGATAACTTCGGGAAACCG

GAGCTAATACCGGATAATATTTTGAACCGCATGGTTCAAAAGTGAAAGAC

GGTCTTGCTGTCACTTATAGATGGATCCGCGCTGCATTAGCTAGTTGGTA

AGGTAACGGCTTACCAAGGCAACGATGCATAGCCGACCTGAGAGGGTGAT

CGGCCACACTGGAACTGAGACACGGTCCAGACTCCTACGGGAGGCAGCAG

TAGGGAATCTTCCGCAATGGGCGAAAGCCTGACGGAGCAACGCCGCGTGA

GTGATGAAGGTCTTCGGATCGTAAAACTCTGTTATTAGGGAAGAACATAT

GTGTAAGTAACTGTGCACATCTTGACGGTACCTAATCAGAAAGCCACGGC

TAACTACGTGCCAGCAGCCGCGGTAATACGTAGGTGGCAAGCGTTATCCG

GAATTATTGGGCGTAAAGCGCGCGTAGGCGGTTTTTTAAGTCTGATGTGA

AAGCCCACGGCTCAACCGTGGAGGGTCATTGGAAACTGGAAAACTTGAGT

GCAGAAGAGGAAAGTGGAATTCCATGTGTAGCGGTGAAATGCGCAGAGAT

ATGGAGGAACACCAGTGGCGAAAGCGACTTTCTGGTCTGTAACTGACGCT

GATGTGCGAAAGCGTGGGGATCAAACAGGATTAGATACC

>J64

ACAAGGCCCGGGAACGTATTCACCGTAGCATTCTGATCTACGATTACTAG

CGATTCCGACTTCACGGAGTCGAGTTGCAGACTCCGATCCGGACTACGAC

GCACTTTATGAGGTCCGCTTGCTCTCGCGAGTTCGCTTCTCTTTGTATGC

GCCATTGTAGCACGTGTGTAGCCCTGGCCGTAAGGGCCATGATGACTTGA

CGTCATCCCCACCTTCCTCCGGTTTATCACCGGCAGTCTCCTTTGAGTTC

CCGACCGAATCGCTGGCAACAAAGGATAAGGGTTGCGCTCGTTGCGGGAC

TTAACCCAACATTTCACAACACGAGCTGACGACAGCCATGCAGCACCTGT

CTCACGGTTCCCGAAGGCACTAAGGCATCTCTGCCGAATTCCGTGGATGT

CAAGGCCAGGTAAGGTTCTTCGCGTTGCATCGAATTAAACCACATGCTCC

ACCGCTTGTGCGGGCCCCCGTCAATTCATTTGAGTTTTAACCTTGCGGCC

GTACTCCCC

>J65A

ACGGTAGCTAATACCGCATAACCTCGTAAGAGCAAAGTGGGGGACCTTCG

GGCCTCACGCTATCGGATGTGCCCAGATGGGATTAGCTAGTAGGTGGGGT

AATGGCTCACCTAGGCGACGATCCCTAGCTGGTCTGAGAGGATGACCAGC

CACACTGGAACTGAGACACGGTCCAGACTCCTACGGGAGGCAGCAGTGGG

GAATATTGCACAATGGGCGCAAGCCTGATGCAGCCATGCCGCGTGTATGA

AGAAGGCCTTCGGGTTGTAAAGTACTTTCAGCGGGGAGGAAGGCGATGAG

GTTAATAACCTTGTCGATTGACGTTACCCGCAGAAGAAGCACCGGCTAAC

TCCGTGCCAGCAGCCGCGGTAATACGGAGGGTGCAAGCGTTAATCGGAAT

TACTGGGCGTAAAGCGCACGCAGG

>J65B

CGGGTGAGTAACACGTGGGTAACCTGCCCATAAGACTGGGATAACTCCGG

GAAACCGGGGCTAATACCGGATAACATTTTGAACTGCATGGTTCGAAATT

GAAAGGCGGCTTCGGCTGTCACTTATGGATGGACCCGCGTCGCATTAGCT

AGTTGGTGAGGTAACGGCTCACCAAGGCAACGATGCGTAGCCGACCTGAG

AGGGTGATCGGCCACACTGGGACTGAGACACGGCCCAGACTCCTACGGGA

GGCAGCAGTAGGGAATCTTCCGCAATGGACGAAAGTCTGACGGAGCAACG

CCGCGTGAGTGATGAAGGCTTTCGGGTCGTAAAACTCTGTTGTTAGGGAA

GAACAAGTGCTAGTTGAATAAGCTGGCACCTTGACGGTACCTAACCAGAA

AGCCACGGCTAACTACGTGCCAGCAGCCGCGGTAATACGTAGGTGGCAAG

CGTTATCCGGAATTATTGGGCGTAAAGCGCGCGCAGGTGGTTTCTTAAGT

CTGATGTGAAAGCCCACGGCTCAACCGTGGAGGGTCATTGGAAACTGGGA

GACTTGAGTGCAGAAGAGGAAAGTGGAATTCCATGTGTAGCGGTGAAATG

CGTAGAGATATGGAGGAACACCAGTGGCGAAAGCGACTTTCTGGTCTGTA

ACTGACACTGAGGCGCGAAAGCGTGGGGAGCAAAC

>J66

CAAGGCCCGGGAACGTATTCACCGTAGCATTCTGATCTACGATTACTAGC

GATTCCGACTTCATGGAGTCGAGTTGCAGACTCCAATCCGGACTACGACG

TACTTTATGAGGTCCGCTTGCTCTCGCGAGTTCGCTTCTCTTTGTATACG

CCATTGTAGCACGTGTGTAGCCCTACTCGTAAGGGCCATGATGACTTGAC

GTCATCCCCACCTTCCTCCGGTTTATCACCGGCAGTCTCCTTTGAGTTCC

CGACCGAATCGCTGGCAACAAAGGATAAGGGTTGCGCTCGTTGCGGGACT

TAACCCAACATTTCACAACACGAGCTGACGACAGCCATGCAGCACCTGTC

TCAGAGTTCCCGAAGGCACCAATCCATCTCTGGAAAGTTCTCTGGATGTC

AAGAGTAGGTAAGGTTCTTCGCGTTGCATCGAATTAAACCACATGCTCCA

CCGCTTGTGCGGGCCCCCGTCAATTCATTTGAGTTTTAACCTTGCGGCCG

TACTCCCCAGGCGGTCGATTTAACGCGTTAGCTCCGGAAGCCACGCCTCA

AGGGCACAACCTCCAAATCGACATCG

>J67

GACCCAGGATCAACACTCCTACGGGAGGCAGCAGTGGGGAATATTGCACA

ATGGGCGCAAGCCTGATGCAGCCATGCCGCGTGTGTGAAGAAGGCCTTAG

GGTTGTAAAGCACTTTCAGCGAGGAGGAAGGGTTCAGTGTTAATAGTACT

GTTCATTGACGTTACTCGCAGAAGAAGCACCGGCTAACTCCGTGCCAGCA

GCCGCGGTAATACGGAGGGTGCAAGCGTTAATCGGAATTACTGGGCGTAA

AGCGCACGCAGGCGGTTTGTTAAGTCAGATGTGAAATCCCCGCGCTTAAC

GTGGGAACTGCATTTGAAACTGGCAAGCTAGAGTCTTGTAGAGGGGGGTA

GAATTCCAGGTGTAGCGGTGAAATGCGTAGAGATCTGGAGGAATACCGGT

GGCGAAGGCGG

>J68

GACACGGTCCACACTCCTACGGGAGGCAGCAGTGGGGAATATTGCACAAT

GGGCGCAAGCCTGATGCAGCCATGCCGCGTGTATGAAGAAGGCCTTCGGG

TTGTAAAGTACTTTCAGCGGGGAGGAAGGCGATGAGGTTAATATCCTCGC

CGATTGACGTTACCCGCAGAAGAAGCACCGGCTAACTCCGTGCCACCAGC

CGCGGTAATACGGAGGGTGCAAGCGTTAATCGCAATTACTGGGCGTAAAG

CGCACGCAGGCGGTCTGTCAAGTCAGATGTGAAATCCCCGGGCTTAACCT

GGGAACTGCATTTGAAACTGGCAGGCTA

>J69

GAACTGAGACAGGGTCCACACTTCTACGGGAGGCAGCAGTGGGGAATATT

GCACAATGGGCGCAAGCCTGATGCAGCCATGCCGCGTGTATGAAGAAGGC

CTTCGGGTTGTAAAGTACTTTCAGCGGGGAGGAAGGCGATGAGGTTATTT

TTTCTCGTTTTATTGACGTTACCCGCAGAAGAAGCACCGGCTAACTCCGT

GCCAGCAGCCGCGGTAATACGGAGGGTGCAAGCGTTAATCGGAATTACTG

GGCGTAAAGCGCACGCAGGCGGTCTGTCAAGTCAGATGTGAAATCCCCGG

GCTTAACCTGGGAACTGCATTTGAAACTGGCAGGCTAGAGTCTTGTAGAG

GGGGGTAGAATTCCAGGTGTAGCGGTGAAATGCGTA

>J70

ACGTATTCACCGTAGCATTCTGATCTACGATTACTAGCGATTCCGACTTC

ACGGAGTCGAGTTGCAGACTCCGATCCGGACTACGACGCACTTTATGAGG

TCCGCTTGCTCTCGCGAGTTCGCTTCTCTTTGTATGCGCCATTGTAGCAC

GTGTGTAGCCCTGGCCGTAAGGGCCATGATGACTTGACGTCATCCCCACC

TTCCTCCGGTTTATCACCGGCAGTCTCCTTTGAGTTCCCGACCGAATCGC

TGGCAACAAAGGATAAGGGTTGCGCTCGTTGCGGGACTTAACCCAACATT

TCACAACACGAGCTGACGACAGCCATGCAGCACCTGTCTCACGGTTCCCG

AAGGCACTAAGGCATCTCTGCCGAATTCCGTGGATGTCAAGGCCAGGTAA

GGTTCTTCGCGTTGCATCGAATTAAACCACATGCTCCACCGCTTGTGCGG

GCCCCCGTCAATTCATTTGAGTTTTAACCTTGCGGCCGTACTCCCCAGGC

GGTCGACTTAACGCGTTAGCT

>J71

GAGTAATGTCTGGGAAACTGCCTGATGGAGGGGGATAACTACTGGAAACG

GTAGCTAATACCGCATAACGTCTACGGACCAAAGTGGGGGACCTTCGGGC

CTCACGCCATCAGATGTGCCCAGATGGGATTAGCTAGTAGGTGGGGTAAT

GGCTCACCTAGGCGACGATCCCTAGCTGGTCTGAGAGGATGACCAGCCAC

ACTGGAACTGAGACACGGTCCAGACTCCTACGGGAGGCAGCAGTGGGGAA

TATTGCACAATGGGCGCAAGCCTGATGCAGCCATGCCGCGTGTGTGAAGA

AGGCCTTAGGGTTGTAAAGCACTTTCAGCGAGGAGGAAGGGTAGTGTGTT

AATAGTACATTGCATTGACGTTACTCGCAGAAGAAGCACCGGCTAACTCC

GTGCCAGCAGCCGCGGTAATACGGAGGGTGCAAGCGTTAATCGGAATTAC

TGGGCGTAAAGCGCACGCAGGCGGTTTGTTAAGTCAGATGTGAAATCCCC

GCGCTTAACGTGGGAACTGCATTTGAAACTGGCAAGCTAGAGTCTTGTAG

AGGGGGGTAGAATTCCAGGTGTAGCGGTGAAATGCGTAGAGATCTGGA

>J72

AACTGAGAACAACTTTATGGGATTTGCTTGACCTCGCGGTTTCGCTGCCC

TTTGTATTGTCCATTGTAGCACGTGTGTAGCCCAAATCATAAGGGGCATG

ATGATTTGACGTCATCCCCACCTTCCTCCGGTTTGTCACCGGCAGTCAAC

TTAAAGTGCCCAACTTAATGATGGCAACTAAGCTTAAGGGTTGCGCTCGT

TGCGGGACTTAACCCAACATCTCACGACACGAGCTGACGACAACCATGCA

CCACCTGTCACTTTGTCCCCCGAAGGGGAAGGCTCTATCTCTAGAGTTGT

C

>J73

AAAGGATGATCAGTCACACTGGAACTGACCCAGGATCAAATCTTGAACGG

GAGGCAGCAGTGGGGAATATTGGACAATGGGCGAAAGCCTGATCCAGCCA

TGCCGCGTGTGTGAAGAAGGTCTTCGGATTGTAAAGCACTTTAAGTTGGG

AGGAAGGGTTGTAGATTAATACTCTGCTATTTTGACGTTACCGACAGAAT

AAGCACCGGCTAACTCTGTGCCAGCAGCCGCGGTAATACAGAGGGTGCAA

GCGTTAATCGGAATTACTGGGCGTAAAGCGCGCGTAGGTGGTTTGTTAAG

TTGGATGTGAAAGCCCCGGGCTCAACCTGGG

>J74

GACACGGTCCACACTCCTACGGGAGGCAGCAGTGGGGAATATTGCACAAT

GGGCGCAAGCCTGATGCAGCCATGCCGCGTGTGTGAAGAAGGCCTTAGGG

TTGTAAAGCACTTTCAGCGAGGAGGAAGGGTTCAGTGTTAATAGCACTGT

GCATTGACGTTACTCGCAGAAGAAGCACCGGCTAACTCCGTGCCAGCAGC

CGCGGTAATACGGAGGGTGCAAGCGTTAATCGGAATTACTGGGCGTAAAG

CGCACGCAGGCGGTTTGTTAAGTCAGATGTGAAATCCCCGCGCTTAACGT

GGGAACTGCATTTGAAACTGGCAAGCTAGAGTCTTGTAGAGGGGGGTAGA

ATTCCAGGTGTAGCGGTGAAATGCGTAGAGATCTGGAGGAATA

>J75

AGGCCCGGGAACGTATTCACCGTAGCATTCTGATCTACGATTACTAGCGA

TTCCGACTTCACGGAGTCGAGTTGCAGACTCCGATCCGGACTACGACGCA

CTTTATGAGGTCCGCTTGCTCTCGCGAGGTCGCTTCTCTTTGTATGCGCC

ATTGTAGCACGTGTGTAGCCCTGGCCGTAAGGGCCATGATGACTTGACGT

CATCCCCACCTTCCTCCGGTTTATCACCGGCAGTCTCCTTTGAGTTCCCG

ACATTACTCGCTGGCAACAAAGGATAAGGGTTGCGCTCGTTGCGGGACTT

AACCCAACATTTCACAACACGAGCTGACGACAGCCATGCAGCACCTGTCT

CACGGTTCCCGAAGGCACTAAGGCATCTCTGCCGAATTCCGTGGATGTCA

AGGCCAGGTAAGGTTCTTCGCGTTGCATCGAATTAAACCACATGCTCCAC

CGCTTGTGCGGGCCCCCGTCAATTCATTTGAGTTTTAACCTTGCGG

>J76

CCTTAGGCGGCTGGCTCCAAAAAGGTTACCCCACCGACTTCGGGTGTTAC

AAACTCTCGTGGTGTGACGGGCGGTGTGTACAAGGCCCGGGAACGTATTC

ACCGCGGCATGCTGATCCGCGATTACTAGCGATTCCAGCTTCATGTAGGC

GAGTTGCAGCCTACAATCCGAACTGAGAACGGTTTTATGAGATTAGCTCC

ACCTCGCGGTCTTGCAGCTCTTTGTACCGTCCATTGTAGCACGTGTGTAG

CCCAGGTCATAAGGGGCATGATGATTTGACGTCATCCCCACCTTCCTCCG

GTTTGTCACCGGCAGTCACCTTAGAGTGCCCAACTTAATGATGGCAACTA

AGATCAAGGGTTGCGCTCGTTGCGGGACTTAACCCAACATCTCACGACAC

GAGCTGACGACAACCATGCACCACCTGTCACTCTGCTCCCGAAGGAGAAG

CCCTATCTCTAGGGTTTTCAGAGGATGTCAAGACCTGGTAAGGTTCTTCG

CGTTGCTTCGAATTAAACCACATGCTCCACCGCTTGTGCGGGCCCCCGTC

AATTCCTTTGAGTTTCAGCCTTGCGGCCGTACTCCCCAGGCGGAGTGCTT

AATGCGTTAACTTCAGCACTAAAGGGCGGAAACCCTCTAACACTTAGCAC

TCATCGTTTA

>J77

CTCACGCCATCAGATGTGCCCAGATGGGATTAGCTAGTAGGTGGGGTAAT

GGCTCACCTAGGCGACGATCCCTAGCTGGGCTGAGAGGATGACCAGCCAC

ACTGGAACTGAGACAGGATCCACACTTCTACGGGAGGCAGCAGTGGGGAA

TATTGCACAATGGGCGCAAGCCTGATGCAGCCATGCCGCGTGTGTGAAGA

AGGCCTTAGGGTTGTAAAGCACTTTCAGCGAGGAGGAAGGG

>J78

ATTCTGATCTACGATTACTAGCGATTCCGACTTCATGGAGTCGAGTTGCA

GACTCCAATCCGGACTACGACATACTTTATGAGGTCCGCTTGCTCTCGCG

AGTTCGCTTCTCTTTGTATATGCCATTGTAGCACGTGTGTAGCCCTACTC

GTAAGGGCCATGATGACTTGACGTCATCCCCACCTTCCTCCGGTTTATCA

CCGGCAGTCTCCTTTGAGTTCCCACCATTACGTGCTGGCAACAAAGGATA

AGGGTTGCGCTCGTTGCGGGACTTAACCCAACATTTCACAACACGAGCTG

ACGACAGCCATGCAGCACCTGTCTCACGGTTCCCGAAGGCACTAAGCCAT

CTCTGGCGAATTCCGTGGATGTCAAGAGTAGGTAAGGTTCTTCGCGTTGC

ATCGAATTAAACCACATGCTCCACCGCTTGTGCGGGCCCCCGTCAATTCA

TTTGAGTTTTAACCTTGCGGCCGTACTCCCCAGGCGGTCGACTTAACGCG

TTAGCTCCGGAAGCCACGCCTCAAGGGCACAACCTCCAAGTCGACATCGT

TTACAG

>J79

TTCTGATCTACGATTACTAGCGATTCCGACTTCATGGAGTCGAGTTGCAG

ACTCCAATCCGGACTACGACATACTTTATGAGGTCCGCTTGCTCTCGCGA

GTTCGCTTCTCTTTGTATATGCCATTGTAGCACGTGTGTAGCCCTACTCG

TAAGGGCCATGATGACTTGACGTCATCCCCACCTTCCTCCGGTTTATCAC

CGGCAGTCTCCTTTGAGTTCCCACCATTACGTGCTGGCAACAAAGGATAA

GGGTTGCGCTCGTTGCGGGACTTAACCCAACATTTCACAACACGAGCTGA

CGACAGCCATGCAGCACCTGTCTCACGGTTCCCGAAGGCACTAAGCCATC

TCTGGCGAATTCCGTGGATGTCAAGAGTAGGTAAGGTTCTTCGCGTTGCA

TCGAATTAAACCACATGCTCCACCGCTTGTGCGGGCCCCCGTCAATTCAT

TTGAGTTTTAACCTTGCGGCCGTACTCCCCAGGCGGTCGACTTAACGCGT

TAGCTCCGGAAGCCACGCCTCAAGGGCACAACCTCCAAGTCGACATCGTT

TACAGCGTGACTACCAGGTATCTAATCCTGTTTGCTCCCCACGCTTTCGC

AC

>J80

AGATGAGCCTAGGTCGGAGTAGCTAGTTGGTGAGGTAAGGGCGCACCAAG

GCGACGATCGACAACTGGGCGGAAAGGATGATCAGTCACACTGGAACTGA

CCCTGGATCAACCTTTCAACGGGAGGCAGCAGTGGGGAATATTGGATAAT

GGGCGAAAGCCTGATCCAGCCATGCCGCGTGTGTGAAGAAGGTCTTCGGA

TTGTAAAGCACTTTAAGTTGGGAGGAAGGGCAGTAAGTTAATACCTTGCT

GTTTTGACGTTACCGACAGAATAAGCACCGGCTAACTCTGTGCCAGCAGC

CGCGGTAATACAGAGGGTGCAAGCGTTAATCGGAATTACTGGGCGTAA

>J81

GGTCCACACTTCTACGGGAGGCAGCAGTGGGGAATATTGCACAATGGGCG

CAAGCCTGATGCAGCCATGCCGCGTGTGTGAAGAAGGCCTTCGGGTTGTA

AAGCACTTTCAGCGAGGAGGAAGGGTTCGGTGTTAATATCACTGTTCATT

GACGTTACTCGCAGAAGAAGCACCGGCTAACTCCGTGCCAGCAGCCGCGG

TAATACGGAGGGTGCAAGCGTTAATC

>J82

CGATTCCGACTTCATGGAGTCGAGTTGCAGACTCCAATCCGGACTACGAC

ATACTTTATGAGGTCCGCTTGCTCTCGCGAGTTCGCTTCTCTTTGTATAT

GCCATTGTAGCACGTGTGTAGCCCTACTCGTAAGGGCCATGATGACTTGA

CGTCATCCCCACCTTCCTCCGGTTTATCACCGGCAGTCTCCTTTGAGTTC

CCGACATTACTCGCTGGCAACAAAGGATAAGGGTTGCGCTCGTTGCGGGA

CTTAACCCAACATTTCACAACACGAGCTGACGACAGCCATGCAGCACCTG

TCTCAGAGTTCCCGAAGGCACTAAGCTATCTCTAGCAAATTCTCTGGATG

TCAAGAGTAGGTAAGGTTCTTCGCGTTGCATCGAATTAAACCACATGCTC

CACCGCTTGTGCGGGCCCCCGTCAATTCATTTGAGTTTTAACCTTGCGGC

CGTACTCCCCAGGCGGTCGACTTAACGCGTTAGCTCCGGAAGCCACGCCT

>J83

GAGCCAGGGTCAACACTCCTACGGGAGGCAGCAGTGGGGAATATTGCACA

ATGGGCGCAAGCCTGATGCAGCCATGCCGCGTGTGTGAAGAAGGCCTTAG

GGTTGTAAAGCACTTTCAGCGAGGAGGAAGGGTAGTGTGTTAATAGCACA

TTGCATTGACGTTACTCGCAGAAGAAGCACCGGCTAACTCCGTGCCAGCA

GCCGCGGTAATACGGAGGGTGCAAGCGTTAATCGGAATTACTGGGCGTAA

AGCGCACGCAGGCGGTTTGTTAAGTCAGATGTGAAATCCCCGCGCTTAAC

GTGGGAACTGCATTTGAAACTGGCAAGCTAGAGTCTTG

>J84

TCCAATCCGGACTACGACGTACTTTATGAGGTCCGCTTGCTCTCGCGAGT

TCGCTTCTCTTTGTATACGCCATTGTAGCACGTGTGTAGCCCTACTCGTA

AGGGACATGATGACTTGACGTCATCCCCACCTTCCTCCGGTTTATCACCG

GCAGTCTCCTTTGAGTTCCCGACCGAATCGCTGG

>J85

TACGATTACTAGCGACTCCGACTTCACGGAGTCGAGTTGCAAACTCCGAT

CCGGACTACGACGCACTTTATGAGGTCCGCTTGCTCTCGCGAGTTCGCTT

CTCTTTGTATGCGCCATTGTAGCACGTGTGTAGCCCTGGCCGTAAGGACC

ATGATGACTTGACGTCATCCCCACCTTCCTCCGGTTTATCACCG

>J86

GCTAATACCGCATAACCTCGTAAGAGCAAAGTGGGGGACCTTCGGGCCTC

ACGCTATCGGATGTGCCCAGATGGGATTAGCTAGTAGGTGGGGTAATGGC

TCACCTAGGCGACGATCCCTAGCTGGTCTGAGAGGATGACCAGCCACACT

GGAACTGAGACACGGTCCAGACTCCTACGGGAGGCAGCAGTGGGGAATAT

TGCACAATGGGCGCAAGCCTGATGCAGCCATGCCGCGTGTATGAAGAAGG

CCTTCGGGTTGTAAAGTACTTTCAGCGGGGAGGAAGGCGATGAGGTTAAT

AACCTCGTCGATTGACGTTACCCGCAGAAGAAGCACCGGCTAACTCCGTG

CCAGCAGCCGCGGTAATACGGAGGGTGCAAGCGTTAATCGGAATTACTGG

GCGTAAAGCGCACGCAGGCGGTCTGTCAAGTCAGATGTGAAATCCCCGGG

CTTAACCTGGGAACTGCATTTGAAACTGGCAGGCTAGAGTCTTGTAGAGG

GGGGTAGAATTCCAGGTGTAGCGGTGAAATGCGTAGAGATCTGGAGGAAT

ACCGGTGGCGAAGGCGGCCCCCTGGACAAAGACTGACGCTCAGGTGCGAA

AGCGTGGGGAGCAAACAGGATTAGATACCCTGGTAGTCCAC

>J87

AACTACTGGAAACGGTAGCTAATACCGCATAACGTCTTCGGACCAAAGTG

GGGGACCTTCGGGCCTCACACCATCGGATGTGCCCAGGTGGGATTAGCTA

GTAGGTGGGGTAATGGCTCACCTAGGCGACGATCCCTAGCTGGTCTGAGA

GGATGACCAGCCACACTGGAACTGAGACACGGTCCAGACTCATACGGGAG

GCAGCAGTGGGGAATATTGCACAATGGGCGCAAGCCTGATGCAGCCATGC

CGCGTGTATGAAGAAGGCCTTCGGGTTGTAAAGTACTTTCAGTGGGGAGG

AAGGCAATGAGGTTAATATCTTCGCTGATTGACGTTACCCGCAGAAGAAG

CACCGGCTAACTCCGTGCCAGCAGCCGCGGTAATACGGAGGGTGCAAGCG

TTAATCGGAATTACTGGGCGTAAAGCGCACGCAGGCGGTCTGTCAAGTCG

GATGTGAAATCCCCGGGCTCAACCTGGGAACTGCATTCGAAACTGGCAGA

CTAGA

>J88

TGCTCTCGCGAGTTCGCTTCTCTTTGTATACGCCATTGTAGCACGTGTGT

AGCCCTACTCGTAAGGGACATGATGACTTGACGTCATCCCCACCTTCCTC

CGGTTTATCACCGGCAGTCTCCTTTGAGTTCCCGACCGAATCGCTGGCAA

CAAAGGATAAGGGTTGCGCTCGTTGCGGGACTTAACCC

>J89

CTGGAAACGGTAGCTAATACCGCATGACCTCGAAAGAGCAAAGTGGGGGA

TCTTCGGACCTCACGCCATCGGATGTGCCCAGATGGGATTAGCTAGTAGG

TGAGGTAATGGCTCACCTAGGCGACGATCCCTAGCTGGTCTGAGAGGATG

ACCAGCCACACTGGAACTGAGACACGGTCCAGACTCCTACGGGAGGCAGC

AGTGGGGAATATTGCACAATGGGCGCAAGCCTGATGCAGCCATGCCGCGT

GTGTGAAGAAGGCCTTAGGGTTGTAAAGCACTTTCAGCGAGGAGGAAGGC

ATCATACTTTTTACGTGTTTTGATTGACGTTACTCGCAGAAGAAGCACCG

GCTAACTCCGTGCCAGCAGCCGCGGTAATACGGAGGGTGCAAGCGTTAAT

CGGAATTACTGGGCGTAAAGCGCACGCAGGCGGTTTGTTAAGTCAGATGT

GAAATCCCCGCGCTTAACGTGGGAACTGCATTTGAAACTGGCAAGCTAGA

GTCTTGTAGAGGGGGGTAGAATTCCAGGTGTAGCGGTGAAATGC

>J90

GGTGTGTACAAGGCCCGGGAACGTATTCACCGCGGCATGCTGATCCGCGA

TTACTAGCGATTCCAGCTTCATGTAGGCGAGTTGCAGCCTACAATCCGAA

CTGAGAACGGTTTTATGAGATTAGCTCCACCTCGCGGTCTTGCAGCTCTT

TGTACCGTCCATTGTAGCACGTGTGTAGCCCAGGTCATAAGGGGCATGAT

GATTTGACGTCATCCCCACCTTCCTCCGGTTTGTCACCGGCAGTCACCTT

AGAGTGCCCAACTTAATGATGGCAACTAAGATCAAGGGTTGCGCTCGTTG

CGGGACTTAACCCAACATCTCACGACACGAGCTGACGACAACCATGCACC

ACCTGTCACTCTGCTCCCGAAGGAGAAGCCCTATCTCTAGGGTTTTCAGA

GGATGTCAAGACCTGGTAAGGTTCTTCGCGTTGCTTCGAATTAAACCACA

TGCTCCACCGCTTGTGCGGGCCCCCGTCAATTCCTTTGAGTTTCAGCCTT

GCGGCCGTACTCCCCAGGCGGAGTGCTTAATGCGTTAACTTCAGCACTAA

AGGGCGGAAACCCTCTAACACTTAGCACTCATCGTTTACGGCG

>J91

TGAGTAATGTCTGGGAAACTGCCTGATGGAGGGGGATAACTACTGGAAAC

GGTAGCTAATACCGCATGACGTCTTCGGACCAAAGTGGGGGACCTTCGGG

CCTCACGCCATCAGATGTGCCCAGATGGGATTAGCTAGTAGGTGGGGTAA

TGGCTCACCTAGGCGACGATCTCTAGCTGGTCTGAGAGGATGACCAGCCA

CACTGGAACTGAGACACGGTCCAGACTCCTACGGGAGGCAGCAGTGGGGA

ATATTGCACAATGGGCGCAAGCCTGATGCAGCCATGCCGCGTGTATGAAG

AAGGCCTTCGGGTTGTAAAGTACTTTCAGCGAGGAGGAAGGCATTAAGGT

TAATAACCTTAGTGATTGACGTTACTCGCAGAAGAAGCACCGGCTAACTC

CGTGCCAGCAGCCGCGGTAATACGGAGGGTGCAAGCGTTAATCGGAATTA

CTGGGCGTAAAGCGCACGCAGGCGGTTGGTTAAGTCAGATGTGAAATCCC

CGAGCTTAACTTGGGAACTGCATTTGAAACTGGTCAGCTAGAGTCTTGTA

GAGGGGGGTAGAATTCCAGGTGTAGCGGTGAAATGCGTAGAGATCTGGAG

GAATACCGGTGGCGAAGGCGGCCCCCTGGACAAAGACTGACGCTCA

>J92

ATAACTCCGGGAAACCGGGGCTAATACCGGATAACATTTTGAACTGCATG

GTTCGAAATTGAAAGGCGGCTTCGGCTGTCACTTATGGATGGACCCGCGT

CGCATTAGCTAGTTGGTGAGGTAACGGCTCACCAAGGCAACGATGCGTAG

CCGACCTGAGAGGGTGATCGGCCACACTGGGACTGAGACACGGCCCAGAC

TCCTACGGGAGGCAGCAGTAGGGAATCTTCCGCAATGGACGAAAGTCTGA

CGGAGCAACGCCGCGTGAGTGATGAAGGCTTTCGGGTCGTAAAACTCTGT

TGTTAGGGAAGAACAAGTGCTAGTTGAATAAGCTGGCACCTTGACGGTAC

CTAACCAGAAAGCCACGGCTAACTACGTGCCAGCAGCCGCGGTAATACGT

AGGTGGCAAGCGTTATCCGGAATTATTGGGCGTAAAGCGCGCGCAGGTGG

TTTCTTAAGTCTGATGTGAAAGCCCACGGCTCAACCGTGGAGGGTCATTG

GAAACTGGGAGACTTGAGTGCAGAAGAGGAAAGTGGAATTCCATGTGTAG

CGGTGAAATGCGTAGAGATATGGAGGAACACCAGT

>J93

AGGCGAGTTGCAGCCTACAATCCGAACTGAGAACGGTTTTATGAGATTAG

CTCCACCTCGCGGTCTTGCAGCTCTTTGTACCGTCCATTGTAGCACGTGT

GTAGCCCAGGTCATAAGGGGCATGATGATTTGACGTCATCCCCACCTTCC

TCCGGTTTGTCACCGGCAGTCACCTTAGAGTGCCCAACTTAATGATGGCA

ACTAAGATCAAGGGTTGCGCTCGTTGCGGGACTTAACCCAACATCTCACG

ACACGAGCTGACGACAACCATGCACCACCTGTCACTCTGCTCCCGAAGGA

GAAGCCCTATCTCTAGGGTTTTCAGAGGATGTCAAGACCTGGTAAGGTTC

TTCGCGTTGCTTCGAATTAAACCACATGCTCCACCG

>J94

GGGAAACCGGGGCTAATACCGGATAACATTTTGAACTGCATGGTTCGAAA

TTGAAAGGCGGCTTCGGCTGTCACTTATGGATGGACCCGCGTCGCATTAG

CTAGTTGGTGAGGTAACGGCTCACCAAGGCAACGATGCGTAGCCGACCTG

AGAGGGTGATCGGCCACACTGGGACTGAGACACGGCCCAGACTCCTACGG

GAGGCAGCAGTAGGGAATCTTCCGCAATGGACGAAAGTCTGACGGAGCAA

CGCCGCGTGAGTGATGAAGGCTTTCGGGTCGTAAAACTCTGTTGTTAGGG

AAGAACAAGTGCTAGTTGAATAAGCTGGCACCTTGACGGTACCTAACCAG

AAAGCCACGGCTAACTACGTGCCAGCAGCCGCGGTAATACGTAGGTGGCA

AGCGTTATCCGGAATTATTGGGCGTAAAGCGCGCGCAGGTGGTTTCTTAA

GTCTGATGTGAAAGCCCACGGCTCAACCGTGGAGGGTCATTGGAAACTGG

GAGACTTGAGTGCAGAAGAGGAAAGTGGAATTCCATGTGTAGCGGTGAAA

TGCGTAGAGATATGGAGGAACACCAGTGGCGAAGGCGACTTTCTGGTCTG

TA

>J95

CGGGAAACCGGGGCTAATACCGGATAACATTTTGAACTGCATGGTTCGAA

ATTGAAAGGCGGCTTCGGCTGTCACTTATGGATGGACCCGCGTCGCATTA

GCTAGTTGGTGAGGTAACGGCTCACCAAGGCAACGATGCGTAGCCGACCT

GAGAGGGTGATCGGCCACACTGGGACTGAGACACGGCCCAGACTCCTACG

GGAGGCAGCAGTAGGGAATCTTCCGCAATGGACGAAAGTCTGACGGAGCA

ACGCCGCGTGAGTGATGAAGGCTTTCGGGTCGTAAAACTCTGTTGTTAGG

GAAGAACAATTTCTTTTTTGAATAAGCTGGCACCTTGACGGTACCTAACC

AGAAAGCCACGGCTAACTAC

>SYC1

CCTTGCCGGCGTCTTCTCGAGCGCTCGAGCGGTCGCGGAGGTGGGTTGCT

ACTTTGCCGGCGAGCGGCGGACGGGTGAGTATGTGAGGGAAACTGGCTGG

TGGAGGGGGACTTTACTGGAAACGGTAGCTAATACCGCATGACCTCGAAA

GAGCAAAGTGGGGGATCTTCGGACCTCACGCCATCGGATGTGCCCAGATG

GGATTAGCTAGTAGGTGAGGTAATGGCTCACCTAGGCGACGATCCCTAGC

TGGGCTGAGAGGATGACCAGCCACACTGGAACTGAGACATGATCCAACTC

TCAACGGGAGGCAGCAGTGGGGAATATTGCACAATGGGCGCAAGCCTGAT

GCAGCCATGCCGCGTGTGTGAAGAAGGCCTTAGGGTTGTAAAGCACTTTC

AGCGAGGAGGAAGGCATCACTCTTAATACGTGTGGTGATTTTTTTTACTC

GCAGAAGAAGCAGCGGCTAACTCCGTGCCAGCAGCCGCGGTAATACGGAG

GGTGCAAGCGTTAATCGGAATTACTGGGCGTAAAGCGCACGCAAGCGGTT

TGTTAAGTCAGATGTGAAATCCCCGCGCTTAACGTGGGAACTGCATTTGA

AACTGGCAAGCTAGAGTCTTGTAGAGGGGGGTAGAATTCCAGGTGTAGCG

GTGAAATGCGTAGAGATCTGGAGGAATACCGGTGGCGAAGGCGGCCCCCT

GGACAAAGACTGACGCTCAGGTGCGAAAAGCGTGGGGAGCAAACAGGATT

AGATACCCTGGTAGTCCACGCTGTAAACGATGTCGACTTGGAGTTGTGCC

CTTTGAGGCGTGGCTTCCGGAGCTAACGCGTTAAGTCGACCGCCTGGGGA

GTACGGCCGCAAGGGTAAAACTCAAATGAATTGACGGGGGCCCGCACAAG

CGGTGGGAGCATGTGGGTTAATTCGATGCAACCCGGAGAACCCTTACCTA

CTTCTTGACATCCACCGGAAATTCCGCCAGAGATGGCCTAATTGCCCTTC

GGGAACCCGTGAGACAGGTGCTGCATGGCTGTCTGCCAGCTCGTGTATTG

TGAAATGGTTGGGGTTAAGTCCCGCAACGAGGCGCACCCTCTTATTCCCT

TTGTTGGCCAGCCCGTTATGGGTGGTAACTTCAAAGGAGACTGGCCTGTG

ATAATCGGGAAGTAGGGTGGGGAATGACGTCAAGTCATTCATGGCCCGTT

ACAGATAGGCTACCCACCGGTGCTCACATTTGTCGTTATACCAAGAAGGA

AAAATCCACATACGTCTTC

>SYC2

ACCCGGTGGGTCCTTGGTAGGACTTCCCCCAGTCATGAATCACAAAGTGG

TAAGCGCCCTCCCGAAGGTTAAGCTACCTACATCTTTTGCAACCCACTCC

CATGGTGTGACGGGCGGTGTGTACAAGGCCCGGGAACGTATTCACCGTAG

CATTCTGATCTACGATTACTAGCGATTCCGACTTCATGGAGTCGAGTTGC

AGACTCCAATCCGGACTACGACATACTTTATGAGGTCCGCTTGCTCTCGC

GAGTTCGCTTCTCTTTGTATATGCCATTGTAGCACGTGTGTAGCCCTACT

CGTAAGGGCCATGATGACTTGACGTCATCCCCACCTTCCTCCGGTTTATC

ACCGGCAGTCTCCTTTGAGTTCCCACCATTACGTGCTGGCAACAAAGGAT

AAGGGTTGCGCTCGTTGCGGGACTTAACCCAACATTTCACAACACGAGTT

GACGACAGCCATGCAGCACCTGTCTCACGGTTCCCGAAGGCACTAAGCCA

TCTCTGGCGAATTCCGTGGATGTCAAGAGTAGGTAAGGTTCTTCGCGTTG

CATCGAATTAAACCACATGCTCCACCGCTTGTGCGGGCCCCCGTCAATTC

ATTTGAGTTTTAACCTTGCGGCCGTACTCCCCAGGCGGTCGACTTAACGC

GTTAGCTCCGGAAGCCACGCCTCAAGGGCACAACCTCCAAGTCGACATCG

TTTACAGCGTGGACTACCAGGGTATCTAATCCTGTTTGCTCCCCACGCTT

TCGCACCTGAGCGTCAGTCTTTGTCCAGGGGGCCGCCTTCGCCACCGGTA

TTCCTCCAGATCTCTACGCATTTCACCGCTACACCTGGAATTCTACCCCC

CTCTACAAGACTCTAGCTTTGCCAGTTTCAAATGCAGTTCCCACGTTAAG

CGCGGGGGATTTCACATCTGACTTAACAAACCGCCTGCGTGCGCTTTTAC

GCCCCAGTAATTCCGATTAAACGCTTTGCACCCTCCGTATTACCGCGGCT

TGCTGGCCACGGAGTTAACCGGTGCTTCTTTCTGGCGAGTACACGTCAAT

CCACACACGTAAATAAGTGGTGATGCCTTCCTTCCTCCGCCTGAAGTGCT

TTAACAACCCTAAGGCCTTCTTCACACACACGCGGGTCATGGGCTGCATC

AAGGCTTGGACCCATTTGTCCAAAATTATCCCCCAACTGGCCTGCCCTCC

CCGTAGGAATCTGGCACGTGTTCTCGATTCAAGGTGTGTGCTTGGGTCAT

CTCTCTCTCTGATGACAACCAAGACTATAGAGGGAA

>SYC3

ACTTGGGGGGGGGCTTCCCATGCGGTCGAGCGGTCGACAGAGAGAGCGTC

TCCTGACACGAAGGTCGGCCGCCAAGGCATATCTGACTGCCTGGGAGAGG

GGGAGGCGACTTTGGCGAACGCGGTACCGCGTAATTCCCACCGCAAAACG

CAAGTGAGCTTCCTTCCTTGCCCTAGCAAATGAGCCTGGGTACCATTCCG

CCATTGGTGAGGGAGGGGCTCACCCAGGCGGGATCCGGATCTGGTCGGAG

AGGAAGATCAGTCACACTGCTCTGATGAGAGTCCAGACTAATCTGGAAGG

CAGCAGTGGGGAATATTGGACAATGGGCGAAAGCCTGATCCAGCCATGCC

GCGTGTGTGAAGAAGGTCTTCGGATTGTAAAGCACTTTAAGTTGGGAGGA

AGGGCAGTTACCTAATACATGATTGTTTTGACGTTATTAACAGAATAAGC

ACCGGCTAACTCTGTGCCAGCAGCCGCGGCATACAGAGGGTGCAAGCGTT

AATCGGAATTACTGGGCGGAGAGCGCGCGTAGGTGGTTTGTTAACTTGGA

TGTGAAATCCCCGGGCTCAACCCTGGGAACTGCTTTCTAAACTGACTGAC

TAGAATATGGTAGAGGGAGGGGGGAATTTTCCTGTGTAGCGGTGAGTGCG

TAGATATAGGAAGGAACACCACTGGCGGAAGGAGACCACCCGGACTACTA

A

>SYC4

AAAGCCGCGGGCCTTACAATGCAAGTCCGAGCGGTAGAGAGAAGCTTGCT

TCTCTTGAGAGCGGCGGACGGGTGAGAAAAGGCTAGGAATCTGCCTGGCA

GTGGGGGATAACGCTCGTTAACGGACGCTAATACCGCATACGTCCTACGG

GAGAAAGCAGGGGACCTTCGGGCCTTGCGCTATCAGATGAGCCTAGGTCG

GATTAGCTAGTTGGTGAGGTAATGGCTCACCAAGGCGACGATCCGTAACT

GGTCTGAGAGGATGATCAGTCACACTGGAACTGAGACACGGTCCAGACTC

CTACAGGAGGCAGCAGTGGGGAATATTGGACAATGGGCGAAAGCCTGATC

CAGCCATGCCGCGTGTGTGAAGAAGGTCTTCGGATTGTAAAGCACTTTAA

GTTGGGAGGAAGGGTTGAAACCTAATACGTTGCAATCTTGACGTTTTTTA

TAGAATAAGCACCGGCTAACTCTGTGCCAGCAGCCGCGGTAATACAGAGG

GTGCAAGCGTTAATCGGAATTACTGGGCGTAAAGCGCGCGTAGGTGGTTC

GTTAAGTTGGT

>SYC5

GGCTGGGCTATTTTGTTACAACTTCCCCACCAGTCATGAATCACACCGTG

GTAACCGTCCTCCCGAAGGTTAGACTAGAAACAGGGGGGGCAACCCACTC

CCATGGTGTGACGGGCGGCGTGATAAAAGGCCCGGGAACGTATTCACCGC

GACATTCTGATTCGCGATTACTAGCGATTCCGACTTCACGCAGTCGAGTT

GCAGACTGCGATCCGGACTACGATCGGTTTTCTGGGATTAGCTCCACCTC

GCGGCTTGGCAACCCTCTGTACCGACCATTGTAGCACGTGTGTAGCCCAG

GCCGTAAGGGCCATGATGACTTGACGTCATCCCCACCTTCCTCCGGTTTG

TCACCGGCAGTCTCCTTAGAGTGCCCACCATGACGTGCTGGTAACTAAGG

ACAAGGGTTGCGCTCGTTACGGGACTTAACCCTACATCTCACGACACGAT

CTGAAGACAGCCATGCAGCACCTGTCTCAATGTTCCCGAAGGCACCAATC

CATCTCTGGAAAGTTCATTGGATGTCAAGGCCTGGTAAGGTTCTTCGCGT

TGCTTCGAATTAAACCACATGCTCCACCGCTTGTGCGGGCCCCCGTCAAT

TCATTTGAGTTTTAACCTTGCGGCCGTACTCCCCAGGCGGTCAACTTTAA

TGGCGTTAGCTGCGCCACTAATAGCTCAAGGCTCCCAACGGCTAGTTGAC

ATCGTTTACGGCCGTGGACTACCAGGGTATCTAATCCTGTTTGCTCCCCA

CGCTTTCGCACCTCAGTGTCAGTATCAGTCCAGGTGGTCGGCCTTCGCAT

TGGTGTTCCTTCCTATATCTACGCATTTTCACCGCTACACAGGAAAATTC

CACCACCCTCTAACCATACTCTAGCTTGTC

>SYC6

AGGAGGGGACTTTGTTTCGACTTTCACCCAGTCATTTGTCACACCTTGGA

AAGCGAGCTCCTAAAAGGTAACTCCAATGGATAAGGGGGTTACAAACTCT

CTGGGGGGGAGGGGGGGGGTGAACAAGACCCGGAAACGAATTCACCGTAC

CATGCTGATCTACGATTACTAGCGATTCCAACTTCATGTAGTCGAGTTGC

AAACTACAATCCGAACTGAGAACAACTTTATGGGATTGGCTTGACCTCGC

GGTTTCGCTGCCCTTTGTATTGTCCATTGTAGCACGTGTGTAGCCCAGAT

CATAAGGGGCATGATGATTTGACGTCATCCCCACCTTCCTCCGGTTTGTC

ACCGGCAGTCACCTTAAAGTGCCCAACTTAATGATGGGAACTAAACTTAA

GGGGTGCGCTCGTTGCGGGACTTAACCCAACATCTCACGACACGAGCTGA

CAACAACCATGCACCACCTGGGACTTTGTCTCCCGAAGGGGAAAGCTCTA

TCTCTAGAGTTGTCAAAGGATGTCAAGATTTGGTAAGGTTCTTCGCGTTG

CTTCGAATTAAACCACATGCTCCACCGCTTGTGCGGGTCCCCGTCAATTC

CTTTGAGTTTCAACCTTGCGGTCGTACTCCCCAGGCGGAGAGCTTAATGC

GTTAGCTGCAACACTAAGGGGCGGAAACCCCCTAACACTTAGCACTCATC

GTTTACGGCGTGGACTACCAGGGTATCTAATCCTGTTTTGATCCCCACGC

TTTCGCACATCAGCGTCAGTTACAGACCAGAAAGTCGCCTTCGCCACTGG

TGTTCCTCCATATCTCTGCGCATTTCACCGCTACACATGGAATTCCACTT

CTCCTCTTCTGCACTCAAGTTTTCCAGTTTCCAATGACCCTCCACGGTGA

GCCGTGAGCTTTCACATCAGACTTAAAACACCGCCTACGCGCGCTTTACG

CCCAATATTCAGGATACGCTTGCCACTACGTATTACCGCGGCTGCTGGCA

ACGATAGTAGGCGGTGGCTTTCTGATGAGTACCGTCAAAAAGTGCACATT

TACTAAAGCTGCTGTTCTTTCACTAAAAACAGAGTTTTACAACCGAAAGA

CTTCATTCACTCACGCGTGATTGGCTACGATCAGGCTTGCGCCAATGTGC

GAAAAATTTCCCTACTGGCTGCTTCGTAAGGAACTGGAACCGTGTCTCAG

TCCGGTGTGCCCGAATCACCCCTTCCAGAACGCACATAGCATCGCTTGCG

CCGTG

>SYC7

CGCTTCCGGCACTCTATCGTGCGCTCGAGCGGTCCGGAAGTGACTTGCTA

CTTTGCCGGCGAGCGGTGGATCTGGAGTATGTCAGGGAAACTGCCTGATG

GAGGGGGACTCTACTGGAAACGGTAGCTAATACCGCATGACCTCGAAAGA

GCAAAGTGGGGGATCTTCGGACCTCACGCCATCGGATGTGCCCAGATGGG

ATTAGCTAGTAGGTGAGGTAATGGCTCACCTAGGCGACGATCCCTAGCTG

GTCTGAGAGGATGACCAGCCACACTGGAACTGAGACATGGTCCAACTCTC

TACGGGAGGCAGCAGTGGGGAATATTGCACAATGGGCGCAAGCCTGATGC

AGCCATGCCGCGTGTGTGAAGAAGGCCTTAGGGTTGTAAAGCACTTTCAG

CGAGGAGGAAGGCATCACACTTAATACGTGTGGTGATTGATGTTACTCGC

AGAAGAAGCACCGGCTAACTCCGTGCCAGCAGCCGCGGTAATACGGAGGG

TGCAAGCGTTAATCGGAATTACTGGGCGTAAAGCGCACGCAGGCGGTTTG

TTAAGTCAGATGTGAAATCCCCGCGCTTAACGTGGGAACTGCATTTGAAA

CTGGCAAGCTAGAGTCTTGTAGAGGGGGGTAGAATTCCAGGTGTAGCGGT

GAAATGCGTAGAGATCTGGAGGAATACCGGTGGCGAAGGCGGCCCCCTGG

ACAAAGACTGACGCTCAGGTGCGAAAGCGTGGGGAGCAAACAGGATTAGA

TACCCTGGTAGTCCACGCTGTAAACGATGTCGACTTGGAGGTTGTGCCCT

TGAGGCGTGGCTTCCGGAGCTAACGCGTTAAGTCGACCGCCTGGGAGTAC

GGCCGCAAGGTTAAAACTCAAATGAATTGACGGGGGCCCGCACAAGCGGT

GGAGCATGTGGTTTAATTCGATGCACGCGAGAACCCTTACCTACTCTTGA

CATCCACGGAATTCGCCTAGAGATGGCTTAGTGCCTTCCGGGACCCGTGA

GAACAGTGCTGCATGGGCTGTCGTCAGCTCGTGTTGTGAAATTGTTGGGT

TTAAGTCCCGCAACGAGCGCCACCCTTATCCTTGGTGGCCAGCCCCGTAT

GGCTGGACTCCAAGGAACCTGGCCGGTGGTAACCGAAGGAAGGTGGGGGG

ATTGACGCTCAGTCACTACTGCCTTTCCAGAATAGGCCTACCACTGGCTT

CAATGCTTATACTAGGAGAGCCGGACTCCCGCGAGGGACGCAAGCGCGGC

AACT

>SYC8

CTGGCGGGGGCCTCCAATGCGCTCGAGCGGTCGCGTAAGTGAGCTGCTCC

TTTGCCGGCGAGCGGCGCCCGCTGGAGTATCTCATGGCGTGTGCCTGGTG

GAGGGGGACTTTACTGGAAACGGTAGCTAATACCGCATGACCTCGAAAGA

GCAGAGAGCGGGATCTTCGGACCTCACGCCATCGGATGTGCAAGATGGGA

TTAGCTAGTAGGTGAGGTAATGGCTCACCTAGGCGACGATCCCTAGCTGG

TCTGAGAGGATGACCAGCCACACTGGAACTGAGACATGATCAAAACTCAG

CGGGCGGCGGGGGTGGGTGGTATTGGGGCGTGGGCCTGATCCTGATGCGC

CCATGCCGCGAGTGTGAACATCGCCTTGTGGATGTATTTAACTTTCAGCG

AAGAGGAAGGACCCACACTTAATACGTGTGGACATTATTTTTTACTCGCA

GAACAAGCACCGGCTGTGCCCGTGCCCGCAGCTAATACTAATACGTGAGG

GCGTTACGTTAAATTACAGTTACGGGGCGTCGCGCGCACGCATGCGTTTT

GTTAAATCAGATGTCACCTCCCTCAGCTTAGCATGTGAACTGCATTTGAA

CCTGGTAAACTAGAGTCTAGTAGAGGGGGGTATCATGCGAAGCGTAGCGA

TGAAATGAGTAAAGATCTAGACAATACCCGAGGGCGACCACCTCCCCCTG

ATCTAGACTGACGCTGCGGTAGCGAAAGCGAGCAGAGCAGACTGGATTAC

ATGCCCTGCTAGTCCGCGCAGTATGTGATGTCGACGTGGAGGTTGTTGGC

CTTCATACTTGCCTTCTGGAGCTAT

>SYC9

GCTCACGGGGGGAGCCTCCCGTGCAAGTCGAGCGGTAGAGAGAAGCTTGC

TTCTCTTGAGAGCGGCGGACGGGTGAGAAAAGAGTAGGAATCTGCCTGGG

AGTGGGGGATAACGCTCGGAAACGGACGCTAATACCGCATACGTCCTACG

GGAGAAAGCAGGGGACCTTCGGGCCTTGCGCTATCAGATGAGCCTAGGTC

GGATTAGCTAGTTGGTGAGGTAATGGCTCACCAAGGCGACGATCCGTAAC

TGGTCTGAGAGGATGATCAGTCACACTGGAACTGAGACACGGTCCAGACT

CATACGGGAGGCAGCAGTGGGGAATATTGGACAATGGGCGAAAGCCTGAT

CCAGCCATGCCGCGTGTGTGAAGAAGGTCTTCGGATTGTAAAGCACTTTA

AGTTGGGAGGAAGGGTTGTAACCTAATACGTTGCAATCTTGACGTTTTCG

ACAGAATAAGCACCGGCTAACTCTGTGCCAGCAGCCGCGGTAATACAGAG

GGTGCAAGCGTTAATCGGAATTACTGGGCGTAAAGCGCGCGTAGGTGGTT

CGTTAAGTTGGATGTGAAATCCCCGGGCTCAACCTGGGAACTGCATTCAA

AACTGACAAGCTAGAGTATGGTAGAGGGTGGTGGAATTTCCTGTGTAGCG

GTGAAATGCGTAGATATAGGAAGGAACACCAGTGGCGAAGGCGACCACCT

GGACTGATACTGACACTG

>SYC10

GGCGGCCGGGGGCCTTGTTTCGATTTCACCCCAATTCATGATCACACCGT

GGTAACCGTCCTCCCGAAGGTTAAACTAGAACATCGGGTGCAACCCACTC

CCATGGTGTGACGGGCGGTGTGTACAAGGCCCGGGAACGTATTCACCGCG

ACATTCTGATTCGCGATTACTAGCGATTCCGACTTCACGCAGTCGAGTTG

CAGACTGCGATCCGGACTACGATCGGTTTTCTGGGATTAGCTCCACCTCG

CGGCTTGGCAACCCTCTGGACCGACCATTGGAGCACGTGTGGAGCCCAGG

CCGTAAGGGCCATGATGACTTGACGTCATCCCCACCTTCCTCCGGTTTGT

CACCGGCAGTCTCCTTAAAGTGCCCACCATAACGTGGTGGTAACTAAGGA

CAAGGGTTGCGCTCGTTACGGGACTTAACCCAACATCTCAAGACTCGAAC

TGAAGACAGCCATGCAGCACCTGTCGCAATGTTCCCGAAGGCACCAATCC

ATCTCTGGAAAGGTCATTGGATGTCAAGGACTGGGAAGGTTCTTCGCGTT

GCTTCGAATTAAACCACATGCTCCACCGCTTGTGCGGGCCCCCGTCAATT

CATTTGAGTTTTAACCTTGCGGCCGTACTCCCCAGGGGGTCAACTTAATG

CGTTAGCTGCCCCACTAGCAGCTCAAGGCTCCCCACGGCTAGTTGACATC

TTTTTACGGCGTGGACTACCAGGGTATCTAAATCCTGTTTGCTTCCCCCC

GCTTTCACACCTCAGTGTCAGTATCAGTCCAGGGGGTCCGCCTTCGCCAC

TGGTGTGCTTCCTATATCTACGCGTTTTACCGCTACACAGGGAAATTCCA

CCACCCTCTACCATACTCCTAGCTTGTCG

>SYC11

CGCCTGTGGGTACCTGTGTTTCGACCTTCACCCCAGTCATGAATCACAAA

AGTGGTAAGCGCCCTCCCGAAGGTTAAGCTACCAAAATAATGTTGCAACC

CACTCCCATGGTGTGACGGGCGGTGTGTACAAGGCCCGGGAACGTATTCA

CCGTGGCATTCTGATCCACGATTACTAGCGATTCCGACTTCACGGAGTCG

AGTTGCAGACTCCGATCCGGACTACGACATACTTTATGAGGTCCGCTTGC

TCTCGCGAGGTGGCTTCTCTTTGTATATGCCATTGTAGCACGTGTGTAGC

CCTACTCGTAAGGGCCATGATGACTTGACGTCATCCCCACCTTCCTCCGG

TTTATCACTGGCAGTCTCCTTTGAGTTCCCGGCCGAACCGGTGGGAACAA

AGGAAAAAGGTTGGGCTCCTTGCGGGAATTAACCCAACATTTCACAACAC

GAATTGAAGACAGCCATGGAGCACCTGGCTCAAAGGTCCCGAAAGGACCA

AAGCATCTCTGCTAAGTTCTCTGGATGTCAAGAGTAGGTAAGGGTCTTCG

CGGTGCATCCAATTAAACCCCATGGTCCACCGCTTGGGCGGGCCCCCGTC

AATTCATTTGAATTTTAACCTTGGGGGCGTACTCCCCAAGGGGGCGACTT

AACGCGTTAACTCCGGAAGCCACTCCTCAAGGGAACAACCTCCATATCTA

CCTCGTTTACCGCGTGGACTACCAAGGTATCTAATCCTGGTTGCTCCCCA

CGCTTTCCCACCTGAGCGGCAGTCTTTGTCCAGGGGGCCGCCTTCGCCAC

CGGTAGTCCTCCAGATCTCTACGCATTTCACCGCTACACCTGGAAATCTA

CCCCCTCTACAAGACTCTAACCTGGCAGTTTCGAATGCCGTTTCAGGGTG

AGCCCGGGGATTTACATCCGACTTGACAGACCGACTGACTGCGCTTTACG

CCCCAATAATTCCGAATTACGCTTTGCACCCCTTCCGTATTTACCGCGGC

TGGCTGGACGAGAGTTAGCCGGGGGCCTTCATTCTGGTACGTACCGGTCA

ATGAAATGCTGGTTATTAACGAAATTCGCCCTTCCTCTCCGACTGTAAGG

TAGTTAACACTCGGAGGGACCTCTTTAATAACCGCGCCATGGCTGCGATC

AGCTTTGCCGCCATTGGGCCAAAATTTCCCCAATGTCTGCCTCCGTTAGA

GGTCTTGAACCGGTTTCAGTCCAATTGGACTGACATCTCTCTCTGAATCA

CAGTATAGCATCTGTCTCTCGCACTTTAGTGGTGATGC

>SYC12

GGGTGGGGGACGTTGGTTCGAATTTCCCTCAGTCATGAATCAAAAGTGGT

AAGCGCCCTCCCGAAGGTTAAGCTAAAACAGCTTTTGCAACCCACTCCCA

TGGTGTGACGGGCGGTGTGTACAAGGCCCGGGAACGTATTCACCGTAGCA

TTCTGATCTACGATTACTAGCGATTCCGACTTCACGGAGTCGAGTTGCAG

ACTCCGATCCGGACTACGACATACTTTGTGAGGTCCGCTTGCTCTCGCGA

GGTCGCTTCTCTTTGTATATGCCATTGTAGCACGTGTGTAGCCCTACTCG

TAAGGGCCATGATGACTTGACGTCATCCCCACCTTCCTCCAGTTTATCAC

TGGCAGTCTCCTTTGAGTTCCCGGCCGAACCGCTGGCAACAAAGGATAAG

GGTTGCGCTCGTTGCGGGACTTAACCCAACATTTCACAACACTATCTGAC

GACAGCCATGCAGCACCTGTCTCAGAGTTCCCGAAGGCACCAAAGCATCT

CTGCTAAATTCTCTGGATGTCAAGAGTAGGTAAGGTTCTTCGCGTTGCAT

CGAATTAAACCACATGCTCCACCGCTTGTGCGGGCCCCCGTCAATTCATT

TGAGTTTTAACCTTGCGGCCGTACTCCCCAGGCGGTCGACTTAACGCGTT

AGCTCCGGAAGCCACTCCTCAAGGGAACAACCTCCAAGTCGACATCGTTT

ACGGCGTGGACTACCAGGGTATCTAATCCTGTTTGCTCCCCACGCTTTCG

CACCTGAGCGTCAGTCTTTGTCCAGGGGGGCGCCTTCGCCACCGGTATTC

CTCCAGATCTCTACGCATTTCACCGCTACACCTGGGAATTCTACCCCCCT

CTACAAGACTCTAGCCTGCCAGTTTCGAATGCAGTTCCCAGGTTGAGCCC

CGGGGATTTCACATCTCGACTTGACAGACCCGCCCTGCGTGGCGCTTTAC

GCCCAAGTAATTTCCCGATAACGCTTTGGACCCTCCGTTATTACCGCGGC

CTGCTGGCACCGGAGTTAGCCGGTGCTTCTTTCTGCGAGTAACGTCAATC

ACTGGGGTTATTAACCACAAATGCCCTTCCTCCTCGCTGAAAGGTACTTA

CAGCCGGAAGGCCTCTTCATAACCCGCGGCATGGCTTGCATCAGGCTGCG

CCCATTGTGCAATATTCCCATTGGCGTGCCTCCGTAGAGATTTGGAACCG

TGTCTCTCAGTTCCAGTGGTGGGCGCGTGGTACAT

>SYC13

CCCAATTTGGAAAGGAAAAAGCGGTAACAGGGCTTAGGTGCTGACTAGTG

GCGACGGGTGAGTAACAAAACGGACCCTGCCTAAAGTGGGGGATACTACT

CCCTATAGAGCTAATACCGCATGAGATCTATGGATGAAAGCAGGGGACCT

TCGGGCCTTGTGCTACTACACCGGCTGATGGCACATTATGTAGTTGGTGG

GGTAAAGGGTTACCGAGCCTGCGATCTGTCGATGGTCTGAGAGGACGACC

AGCCACACTGGGACAGAGACACGGACCCAACTCCTACAGGAGGCAACAAT

GGGGAATTTTGGACAATGGGCGAAAGCCTGATCCCCCAATGCCCCCTGCA

GGATGAAGGCCCTCGGGTCGTAAGCTGCTTTTGTACTGTCCGAAAAGCGT

GGGGCTAATACCCCCGGGTCATGACGGAAATATAATAATAACCACCGACT

AACTACGTGTCACCATCCGCGCTAATACCTAGGGTGCGAGCGTGAATCCG

AATTACTGGGCGTAAAGCCTGTGCCGCGGTTTTGTAACACTTGGTGAAAT

CCCCGCTCCCCACTGTGATCTGCCATTGTGATGGAAACTAAAGAGCAGAC

ACGGGGATAGGATTCCACGTGTACCGTGTACTGCGTATATGTGCACAGAA

ACACCGATGGCC

>SYC14

TCCCTGAAAATCCCCCGGCCCGGGGGGAAGCGCCCTCCTTTGAGGTTAGG

TTAGAAACGGGCGGGGGGAGACCCGTTCCATGGTGTGACGGGCGCTGCCG

TAAAAGGACCCGGGAACGTATTCACCGTGACATTCTGATCCCGATTACTA

GCGATTCCGACTTCACGCAGTCGAGTTGCAGACTGCGATCCGGACTACGA

CTGGTTTTATGGGATTAGCTCCCCCTCGCGGGTCGGCAACCCTTTGTACC

AGCCATTGTATGACGTGTGTAGCCCCACCTATAAGGGCCATGAGGACTTG

ACGTCATCCCCACCTTCCTCCGGTTTGTCACCGGCAGTCCCATTAGAGTG

CTCAACTGAATGTATGAACTAATGGGAAGGGTTGCGCTCTTTGTGGGACT

TATCCCAACATCTCACGACACGATTAGATAACAGCCATGCGGCACCGGTG

TGCAGGTTCTCTTTAAAGCACCAAACCATCTCTGGTAAGTTCCTGCCATG

TCAAAAGTGGGTAAGGTTTTTCGCGTTGCATCCAATTAAACCACATCATC

CACCGCTTGTGCGGGTCCCCGTCAATTCCTTTGAGGTTCAATCTTGGGGC

CGTACTCCTCAGGCGGGCAACTTCACTCTTTAGCTTCTTTACTGACTCAT

TTAAGACTCAACAACCAATTGACATCTT

>SYC15

CGGCATTGGGGACTTGGTTTGGACTTCCCCCAGTCACGAATCCCGCCGTG

GTAAGCGCCCTCCTTGAGGTTAGGCTAAAAAAAGGGCGAGACCCGCTCCC

AGGGGGGGACGGGGGGTGTGTACAAGACCCGGGAACGTATTCACCGTGAC

ATTCTGATCCACGATTACTAGCGATTCCGACTTCACGCAGTCGAGTTGCA

GACTGCGATCCGGACTACGACTGGCTTTATGGGATTAGCTCCCCCTCGCG

GGTTGGCAACCCTTTGTACCAGCCATTGTATGACGTGTGTAGCCCCACCT

ATAAGGGCCATGAGGACTTGACGTCATCCCCACCTTCCTCCGGTTTGTCA

CCGGCAGTCCCATTAGAGTGCTCAGCTGAATGTAGCAACTAATGGGAAGG

GTTGCGCTCGTTGCGGGACTTAACCCAACATCTCACGACACGAGCTGACA

ACAGCCATGCAGCACCTGTGGGGAGGTTCTCTTTCAAGCACCAAACCATC

TCTGGTAAGTTCCTGCCATGTCAAAGGTGGGTAAGGTTTTTCGCGTTGCA

TCGAATTAAACCACATCATCCACCGCTTGTGCGGGTCCCCGTCAATTCCT

TTGAGTTTCAACCTTGCGGCCGTACTCCCCAGGCGGGTCAACTTCACGCG

TTAGCTTCGTTACTGAGTCATTTAAGAACCAACAACCAGTTGACATCGTT

TTAGGGCGTGGACTACCAGGGTATCTAATCCTGTTTGCTCCCCACGCTTT

CGTGCATGAACGTCAGTGCAGGCCCAGGGGATTGCCTTCGCCATCGGGGG

TTCCTCCGCATATCTACGCATTTCACTGCTACACCGCGGAATTCCATCCC

CCTCTGCCGGACTCTAGCTTTGCAGTCACAGTGGCAGTTCCCAGGTTGAG

CCCGGGGATTTCACCACTGTCTTACAAAAACGGCCTGCGCACGGCTTTAC

GCCCAGT

>SYC16

GGCCCTGTGGTTCCTTTGTTTAGACTTCACCCAGTCATGAATCACACCGT

GGTAACCGTCCTCCCGAAGGTTAGACTAGCTACAAACTGGTGCAACCCAC

TCCCATGGTGTGACGGGCGGTGTGTACAAGGCCCGGGAACGTATTCACCG

CGACATTCTGATTCGCGATTACTAGCGATTCCGACTTCACGCAGTCGAGT

TGCAGACTGCGATCCGGACTACGATCGGTTTTATGGGATTAGCTCCACCT

CGCGGCTTGGCAACCCTCTGTACCGACCATTGTAGCACGTGTGTAGCCCA

GGCCGTAAGGGCCATGATGACTTGACGTCATCCCCACCTTCCTCCGGTTT

GTCACCGGCAGTCTCCTTAGAGTGCCCACCATTACGTGCTGGTAACTAAG

GACAAGGGTTGCGCTCGTTACGGGACTTAACCCAACATCTCACGACACGA

GTTGACAACAGCCATGCAGCACCTGTCTCAATGTTCCCAAAGGCACCAAT

CTATCTCTAGAAAGTTCATTGGATGTCAAGGCCTGGTAAGGTTCTTCGCG

TTGCTTCAAATTAAACCACATGCTCCACCGCTTGGGCGGGCCCCCGTCAA

TTCATTTGAGTTTTAACCTTGCGGCCGTACTCCCCAGGCGGTCAACTTAA

TGCGTTAGCTGCGCCACTAAAAGCTCAAGGCTTCCAACGGCTAGTTGACA

TCGTTTACGGCGTGAACTACCAGGGTATCTAATCCTGTTTGCTCCCCACG

CTTTCGCACCTCAGTGTCAGTATTAGTCCAGGTGGTCCCCTTCGCCACTG

GTGTTCCTTCCTATATCTACGCATTTCACCGCTACACAGGAAATTCCACC

ACCCCTCTACCATACTCTAGCTCAGTCATTTTTGAATGCAGTTCCCAGGT

TAGACCCCGGGGTATTTCACATCCAATTTAACAAACCACCTGACGCGCGG

CTTTACGCCCAGAAATTCCGAATTAACCGCTTGCACCCTCTCTGTATTAC

CGGCGGCCTGCTGGACACAGATTAGCGCGGTGTTTATTTCTTGTCGGAAC

GTCAAAACAATCGACGTAATAACGTAACTTGCCCCTTTCTCTCCCACTTA

AAGATGCTTACAACTCGAAAAGAACCTTCTTCCACACCCCGGCCATGGCC

TCGAAATCAGGGCTTCGCCAATTGCATTATTCCCACATTGCTGCCTCCGG

TAAGAAGTTCGGAACCGGTCCCAAGTTCCAGTGTGACGTGAACATCCCTC

GGAACGATTAACGGATCGTTCGCCTTTGTGGAGGAC

>SYC17

GGGGTCCACTTTGGTTAGAATTTTCCCCCCAGTCATGAATCACACCGTGG

TAAGCGCCCTCCCGAAGGTTAGACTAGCAAAACGGGCGCGACCCACTCCA

TGGGGGGACGGGCGGTGTGTACAAGGCCCGGGAACGTATTCACCGCGACA

TTCTGATTCGCGATTACTAGCGATTCCGACTTCACGCAGTCGAGTTGCAG

ACTGCGATCCGGACTACGATCGGTTTTGTGAGATTAGGTCCGCCTCGCGG

CTTGGCAACCCTCTGTACCAACCATTGTATCACGTGTGTAGCCCCAGCCA

TAAGGGCCATGATGACTTGACGTCATCCCCACCTTCCTCCGGTTTGTCAC

CGGCAGTCTCCTTAGAGTGCCCAACTGAATGTGCTGGCAACTAGGGACGG

GGGTGGTCCTCGTTAGGGTAATTACCCCATCATCTCACGACATTAGTTAA

TAACAGCCCTGCAGCCCGTGGGTCGAAGTTCCCGAAGGCACCAACCCTCT

TTTAGAAATTTCTGCGCAGGTAAAGGTGTGGAAGGGTTCTTCCCGTTGCT

TCAAATTAAACACCATGCCCCACCCCTGGGGGGGTCCCCCGCAATTCCAT

TTAAGTTAAAACTTGCCGGCGGAACCCCCAAGGGGTCACTTCAATGGGTT

ACTTGCGAACGACTCAGTTAGGATCCACCCGATATGTGACCGTTTAAGGG

CGGGAACCCGGAATCATTCTCTGTGCTTCCCCGCTCTCCCCCCTGCC

>SYC18

ACCACATTGCCAAGTTCGAGCGGGTAGGCACAGGGAGCTTGCTCCTGGGT

GACGAGCGGCGGACGGGGAGGAAGGTCTGGGAAACTGCCTGATGGAGGGG

GATAACTACTGGAAACGGTAGCTAATACCGCATAACGTCGCAAGACCAAA

GAGGGGGACCTTCGGGCCTCTTGCCATCAGATGTGCCCAGATGGGATTAG

CTAGTAGGTGGGGTAATGGCTCACCTAGGCGACGATCCCTAGCTGGTCTG

AGAGGATGACCAGCCACACTGGAACTGAGACACGGTCCAGACTCCTACGG

GAGGCAGCAGTGGGGAATATTGCACAATGGGCGCAAGCCTGATGCAGCCA

TGCCGCGTGTATGAAGAAGGCCTTCGGGTTGTAAAGTACTTTCAGCGAGG

AGGAAGGCATTGTGGTTAATAACCGCAGTGATTGACGTTACTCTCAGAAG

AAGCACCGGCTAACTCCGTGCCAGCAGCCGCGGTAATACGGAGGGTGCAA

GCGTTAATCGGAATTACTGGGCGTAAAGCGCACGCAGGCGGTCTGTCAAG

TCGGATGTGAAATCCCCGGGCTCAACCTGGGAACTGCATTCGAAACTGGC

AGGCTAGAGTCTTGTAGAGGGGGGGTAGAATTCCAGGTGTAGCGGTGAAA

TGCGTAGAGATCTGGAGGAATACCGGTGGCGAAGCGGCCCCCTGGACAAA

GACTGACGCTCAGGTGCGAAAGCGTGGGGAGCAAACAGGATTAGATACCC

CTGGTAGTCCCACGCCTGAAACTGATGTCCACTTTGGAGGGTTGTTCCCT

TGAGGAGTGGCTTCCGGAGCTAACGCGTTAAGTCGACCGCCTGGGAGTAC

GGCCCGCAATGTTAAACCTCAATGAATTGACCGGGGCCCGCACAAGCCGG

TGCGAGCATGTG

>SYC19

CCCGGGTGGGTCCTTGTTTCAACATTCACCCCAGTCATGAATCACAAAGT

GGTAAGCGCCCTCCCGAAGGTTAAGCTACTACAACTTTTGCAACCCACTC

CCATGGTGTGACGGGCGGTGTGTACAAGGCCCGGGAACGTATTCACCGTA

GCATTCTGATCTACGATTACTAGCGATTCCGACTTCACGGAGTCGAGTTG

CAGACTCCGATCCGGACTACGACATACTTTGTGAGGTCCGCTTGCTCTCG

CGAGGTCGCTTCTCTTTGTATATGCCATTGTAGCACGTGTGTAGCCCTAC

TCGTAAGGGCCATGATGACTTGACGTCATCCCCACCTTCCTCCAGTTTAT

CACTGGCAGTCTCCTTTGAGTTCCCGGCCGAACCGCTGGCAACAAAGGAT

AAGGGTTGCGCTCGTTGCGGGACTTAACCTTACATTTCACAACACGAGTT

GACGACAGCCATGCAGCACCTGTCTCAGAGTTCCCGAAGGCACTAAAGCA

TCTCTGCTAAATTCTCTGGATGTCAAGAGTAGGTAAGGTTCTTCGCGTTG

CATCGAATTAAACCACATGCTCCACCGCTTGTGCGGGCCCCCGTCAATTC

ATTTGAGTTTTAACCTTGCGGCCGTACTCCCCAGGCGGTCGACTTAACGC

GTTAGCTCCGGAAGCCACTCCTCAAGGGAACAACCTCCAAGTCGACATCG

TTTACGGCGTGGACTACCAGGGTATCTAATCCTGTTTGCTCCCCACGCTT

TCGCACCTGAGCGTCAGTCTTTGTCCAGGGGGCCGCCTTCGCCACCGGTA

TTCCTCCAGATCTCTACGCATTTCACCGCTACACCTGGAATTCTACCCCC

CTCTACAAGACTCTAGCCTGCCAGTTTCGAATGCAGTTCCCACGTTGAGC

CCGGGGATTTCACATCCGACTTGACAGACCGCCTGCGTGCCGCTTTACGC

CCAGTAATTTCCGATTAACGCTTGCACCCTCCGTATTACCGCGGCTGCTG

GCACGGAAGTTAGCCCGGTGCTTCTTCTGGGAGTAACGTCAAATCACTTG

GTGGGTTATTAACCCACCAATGCCCTTCCCTCCTCGCTGAAAGGTACCTT

TACAACCTCGGAAGGCCCTTTCTTTCATTAAACGCGGGCAATGGCTGGAA

TCCAGCTTTGCGCCCCATTTGAGCAAATATTTCCCCACTGCCTGGCTCCC

GTTAGGAGATCTTGCACGGTCTTCAGTTCAGTGTGGCGTTGGACTATCCT

CTTCTCCAAGAACCAGCTTATGCGGAGAATCCGTGC

>SYC20

TGCCCCAAGTTCGAAGCGGTAGAGAGAAGCTTGCTTCTCTTGAGAGCGGC

GGACGGGTGAGTAAAGCCTAGGAATCTGCCTGGTAGTGGGGGATAACGTT

CGGAAACGAACGCTAATACCGCATACGTCCTACGGGAGAAAGCAGGGGAC

CTTCGGGCCTTGCGCTATCAGATGAGCCTAGGTCGGATTAGCTAGTTGGT

GAGGTAATGGCTCACCAAGGGGGCGATCCGTAACTGGTCTGAGAGGATGA

TCAGTCACACTGGAACTGAGACACGGTCCAGACTCCTACGGGAGGCAGCA

GTGGGGAATATTGGACAATGGGCGAAAGCCTGATCCAGCCATGCCGCGTG

TGTGAAGAAGGTCTTCGGATTGTAAAGCACTTTAAGTTGGGAGGAAGGGC

AGTTACCTAATACGTAATTGTTTTGACGTTACCTTCAGAATAAGCACCGG

CTAACTCTGTGCCAGCAGCCGCGGTAATACAGAGGGTGCAAGCGTTAATC

GGAATTACTGGGCGTAAAGCGCGCGTAGGTGGTTTGTTAAGTTGAATGTG

AAATCCCCCGGGCTCAACCTGGGAACTGCATCCAAAACTGGCAAGCTAGA

GTATGGTAGAGGGTGGTGGAATTTCCTGTGTAGCGGTGAAATGCGTAGAT

ATAGGAAGGAACACCAGTGGCGAAAGGCGACCACCTGCACTGATACTGAC

ACTGAGGTGCGAAAGCGTGGGGAGCAAACAGGATTACATACCTCTGGTAG

TCCACGCCGTAAACGATGTCAACTAGCCGTTGGGAGCCTTGATCTCTTAT

GGGCAGCTAACGCATTAAGTGACCGCCTGGCGATACGGCGCAGGTTAAAC

TCAAATGAATTGACGGGGCCCGCACAGCGGGGACACGTGGCTAATTCGAG

CACGCGAAAGAAACTTACCAGGCCTTGACATCCAAGAACTTTCCTAGAGA

TAAATTGGTTGCCTTCCGGAGCATTGAAACAGTGCTGGAATGGCTTGTCG

TCAGCTTCG

>SYC21

TTTTTGCAATTTCCAAACGCGAAGAACAATAGTTTGCTCTTCCCTTCTGC

GGCGGGCGGGTGAGAAAAAAAAGGCTCTGTCTGGTAGTGGGAGACACGTT

CAAAAGGAACGGTAACCCCGTATACTTTTTACGGGAAAACGGGGGGATCT

TCGGGCTTTGCTCTATCAGATGATCCTAGGTCGTATTGTCTACCTGGTGG

GGTAAGGGCTCCCCCCCGGGGGGGAGGCGTCTCTGGTCTGAGAGGAAGAA

CACTCACTCTGGCTCTGACACACCCTCCAGACTCCTACGGGAGGCAGCTG

GGGGAATTTTTGCACTGTGGGTGAAAGCCTGCCCCCCCCGTCCCGCGCGC

GTGAAAAACCCCTCCCGATTGTACAGCACTTTTACTTGGGAGGAAGGCTG

GTGCTCTCATACTTTTTTGTTGTCACGTTTTATTTTAATATACGCAGCGG

CTGTCTCTGTGCCCCCACCGCTGCATACAGATTGTGTTTCGTTTTTTATC

TTTTTTTATTGCG

>SYC22

ACCAAGGGTAGCGGTTTTCAACTTACCCCAGTCATGAATCACAACCGTGG

TAACCGTCCCCCCGAAGGTTAGACTAGCAACATCTGGTGCAACCCACTCC

CATGGTGTGACGGGCGGTGTGTACAAGGCCCGGGAACGTATTCACCGCGA

CATTCTGATTCGCGATTACTAGCGATTCCGACTTCACGCAGTCGAGTTGC

AGACTGCGATCCGGACTACGATCGGTTTTATGGGATTAGCTCCACCTCGC

GGCTTGGCAACCCTTTGTACCGACCATTGTAGCACGTGTGTAGCCCAGGC

CGTAAGGGCCATGATGACTTGACGTCATCCCCACCTTCCTCCGGTTTGTC

ACCGGCAGTCTCCTTAGAGTGCCCACCATTACGTGCTGGTAACTAAGGAC

AAGGGTTGCGCTCGTTACGGGACTTAACCCAACATCTCACGACACGAGCT

GACGACAGCCATGCAGCACCTGTCTCAATGCTCCCGAAGGCACCAATCTA

TCTCTAGAAAGTTCATTGGATGTCAAGGCCTGGTAAGGTTCTTCGCGTTG

CTTCGAATTAAACCACATGCTCCACCGCTTGTGCGGGCCCCCGTCAATTC

ATTTGAGTTTTAACCTTGCGGCCGTACTCCCCAGGCGGTCAACTTAATGC

GTTAGCTGCGCCACTAAGAGCTCAAGGCTCCCAACGGCTAGTTGACATCG

TTTACGGCGTGGACTACCAGGGTATCTAATCCTGTTTTGCTCCCCACGCT

TTCGCACCTCAGTGTCAGTATCAGTCCAGGTGGTCGCCTTTCGCCACTGG

TGTTCCTTCCTATATCTACGCATTTCACCGCTACACAGGAAATTCCACCA

CCCTCTACCATACTCTAGCTTGCCAGTTTTGGATGCAGTTTCCCAGGTTG

AGCCCGGGGATTTTCACATTTCAACTTAAACAACCACCTACGCGCGCTTT

ACGCCCAGTAATTCCGAATAACGGCTTGCACCCTCTGTATTACCGCGGCT

GCTTGCACAGAGTTAGCCGGGGGCTTATTTCTGTCGTAACGTCCAAAACC

AATTTACGTAATTAAGGTAACTGCCCTTTCCTCCCAACTTTAAGGTTGCT

TTTACAAATCCGGAGAACTTTCTTTCCACAAACGCGGGCATGGGCTGGGA

TACGGCTTTTCGACCCAATTGGTACCAATATTTCCCCACTGGCTGCTTCC

GTAAGAGACTGTGGAACCGGGTCTCAAGCTTCCAGTGTGTGACCTGAAAC

TACTTCCTCTTTCGAGACCAGCTATACGGGAATTGAGATGGGCGTTTTGT

TGA

>SYC23

CTGGGGGGTCCTCTCGACCGCTAGGAGCGGTCGCCTAGGTGAGTGTTACC

CCACCTACTAGCTAATCCCATCTGGTCACATCTGATGGCCTGAGGCCCGA

AGGTCCGACTCTTTGGTCTTGCGACGTTATGCGGGATTAGCTACCGTTTA

CCGAAGAGATCCCCCTCCATCAGGCTTTTCCCAGACATTACTCACCCGTC

CGCCGCTCGTCACCCAAAAATGTGTCACCTGTGCGACAATATCTATTGGT

GCTGAGAGAATGACCGCCCACACTCTGACAGAGCCATGATCTAACTCTCT

GAGGAGGGGTGGGTGTGGAGATTGCGCGCTGGGCGCCCGCCTCTTACATC

CCCGCCGCGTGTATAAAAAAAGCCCCCTGGGTGTGAAGAACTTTTTCCGA

GAAGGAAAGCATTGTGGTTTATCACCCCACTGAGTTTTATTATTCTCACA

AAAGACGCCGGGGAAGGCTGCGCCCCCAGCCGCGGGAAAACACAGAGGGT

AATCGTTAATCTTACTTTTTCGGGGGAGAGAGCACACACGCGGGGTGTCA

CAACTGATATGTGAAATCCCCGGTCACCCTGGGAACTGCGTTCTCAACTC

TGATGCGATAGACTTGTAGAGGGGGGGGATACATTTCCGCGTGGCGCAGA

AATATGTATAGATATGGAGGAATATCGCTGGCGAAAACGCCCCCCCCTGC

AATACAGAGG

>SYC24

GGCCTGGGGGGGCCTAACCATGCATGTCGAGCGGTAGAGAGAGGCTTGCT

TCTCTTGGGAGCGGCGGAGGGGTGAGGAAGGCCTAGGAATCTGCCTGGGA

GTGGGGGAGAACGTTCACTACTGAACGCTAATACCGCATACGTCCAACGG

CAAAAAGCAAGGGAGCTTCAGCCCTTGGCCTCTCGCATTAGCATGTGTCG

GATTAGCAATATGTAGAGGGAGGGGCACTGGCTGGCCACGAGGACGATCC

GGACCTGGACGGAGATCATGCCCACTGGCACTGAAACTGAGACCCGACCC

ATACTCCTACCGGAGGTGGCGAAGGTTGATATTGGACAATGGGCCTGATC

CAGATGTGGCCATGACGCGTGTGAGGACTACGCCTTATAGATGACTTGCA

CTTGCGAGGAAGAGCAATTACCTAGTGTTTATATGACATTGCGTTGCCGT

TACTCTAAGAACAGGCTAACTCTGTGCCGTGCCCGCAGCCGCGGTAATAC

GGAGGGTGTTAATGTTAATTACAATTACTGGGCGTCGCGCGCACGCGTTT

GTTTTGTTAATGTGATGTCCCCTCCCTCGCGCTTGACATGTGAATTGCAT

TTTGGACTGACTAAACTATAGTCTAGTATAGTGGGATTTCAATGCCTGGT

GTAGCGGTGAAATGCTATAAGATCTAGAAGAATAGCCGTGGGCAACGCCT

CCCCCTAGTACAAAAACTGACGCTCCGGTGCCATGGCGAGCGGACCAGAT

TAGATACCATAGTCTGCCACTCCGTACAGTAAATCATGTAGATTTGGAAG

GTCTGCCCCTTATGCGGGCTTACCGGAAGCATTGCGTTAAATCCACCGGC

CAGGGCAGTCCCGACGCTAAAGATTCAACTCAAATTGACTTGACGGCGAG

ACCAGCCGTGCAGCAGTGATCATGTGGATTAATTCGATGCATCGAAACGT

ATACCATGGCCTTCGTCATGCACATGCACATAACTTTTCCATAGATGG

CC

>SYC25

CGTAAGGTTCCTTTGGTGAAGACCTTCCCCCAGTCCTGAATCACAAGGTG

GGAAGCGCCCTCCCGAAGGTTAAGCTAAAAAAGGGGGGGTGCGACCCACT

CCCATGGGGGGACGGGCGGCGCCAACAAAGGCCCGGGAACGTATTCACCG

TGGCATTCTGATCCACGATTACTAGCGATTCCGACTTCACGGAGTCGAGT

TGCAGACTCCGATCCGGACTACGACATACTTTATGAGGTCCGCTTGGTCT

CGCGAGGTCGCTTCTCTTTGTATATGCCATTGGAGCACGTGTGTAGCCCT

ACTCGTAAGGGCCATGATGACTTGACGTCATCCCCACCTTCCTCCAGTTT

ATCACTGGCAGTCTCCTTTGAGTTCCCGGCCGAACCGCTGGCAACAAAGG

ATAAGGGTTGCGCTCGTTGCGGGACTTAACTTAACATTTCACAACTCTAT

CTGACGACAGCCATGCATCGGGGGTCTCAGAGTTCCCGAAGGCACCAAAG

CATCTCTGCTAAGTTCTCTGGATGTCAAGAGTAGGGAAGGTTCTTCGCGT

TGCATCGAATTAAACCACATGCTCCACCGCTTGTGCGGGCCCCCGACAAT

TCATTTGAGTTTTAACCTTGCGCCGTACTCCCCAGGCGGTCGAC

>SYC26

GGGGGGGGTTCTTTGTTTTCGACTTGACCCCAGTCATGAATCACAAAGTG

GTAAGCGCCCTCCCGAAGGTTAAGCTAATAAAAAATTTTGCAACCCACTC

CCATGGTGTGACGGGCGGTGTGTACAAGGCCCGGGAACGTATTCACCGTA

GCATTCTGATCTACGATTACTAGCGATTCCGACTTCATGGAGTCGAGTTG

CAGACTCCAATCCGGACTACGACGTACTTTATGAGGTCCGCTTGCTCTCG

CGAGTTCGCTTCTCTTTGTATACGCCATTGTAGCACGTGTGTAGCCCTAC

TCGTAAGGGCCATGATGACTTGACGTCATCCCCACCTTCCTCCGGTTTAT

CACCGGCAGTCTCCTTTGAGTTCCCGACCGAATCGCTGGCAACAAAGGAT

AAGGGTTGCGCTCGTTGCGGGACTTAACCCAACATTTCACAACACGAGCT

GACGACAGCCATGCAGCACCTGTCTCAGAGTTCCCGAAGGCACCAATCCA

TCTCTGGAAAGTTCTCTGGATGTCAAGAGTAGGTAAGGTTCTTCGCGTTG

CATCGAATTAAACCACATGCTCCACCGCTTGTGCGGGCCCCCGTCAATTC

ATTTGAGTTTTAACCTTGCGGCCGTACTCCCCAGGCGGTCGATTTAACGC

GTTAGCTCCGGAAGCCACGCCTCAAGGGCACAACCTCCAAATCGACATCG

TTTACAGCGTGGACTACCAGGGTATCTAATCCTGTTTGCTCCCCACGCTT

TCGCACCTGAGCGTCAGTCTTTGTCCAGGGGGCCGCCTTCGCCACCGGTA

TTCCTCCAGATCTCTACGCATTTCACCGCTACACCTGGAATTCTACCCCC

CTCTACAAGACTCTAGCTTGCCAGTTTCAAATGCAGTTCCACGGTAAGCG

CGGGATTTCACATCTGACTTAACAAACCGCCTGCGTGCGCTTTACGCCAG

TAATTTCCGATTAACCGCTTGCCCCCTCCGTATTACGGCGGCTGCTGGCA

CGGAGTTAGCCGGTGCTACTTCTGCCGAGTACCGTCAATGCAATGGGGCT

ATTAGCACCACTACCCTTTCGTCTTGGCTGAAAGTCTTAACATCCTGAAG

GCTTTCTCACACACCCGGCATGCTGCATTCAGGCCTTGGCCCATGGGGCA

ATATTCCCCCATTGCGTGCCCTCCGGTAGGAGCTTCTGGGACCGGTCTCA

AGTCAGGGTGGCTGGCATCTTCTTGCAAACACAGCATTAGGATACG

>SYC27

ACGGCATGGGGTCTTGGTTTCGACTTCCCCCAGTCATGAATCACAAAGTG

GTAAGCGCCCTCCCGAAGGTTAAGCTAGGAAACTGTTGCAACCCACTCCA

TGGTGTGACGGGCGGTGTGCACAAGGCCCGGGAACGTATTCACCGTGGCA

TTCTGATCCACGATTACTAGCGATTCCGACTTCACGGAGTCGAGTTGCAG

ACTGCGATCCGGACTACGACATACTTTATGAGGTCCGCTTGCTCTCGCGA

GGTGGCTTCCCTTTGTATATGCCATTGTAGCACGTGTGTAGCCCTACTCG

TAAGGGCCATGATGACTTGACGTCATCCCCACCTTCCTCCAGTTTATCAC

TGGCAGTCTCCTTTGAGTTCCCGGCCTAACCGCTGGCAACAAAAGAAAAG

GGTTGGCCTCGTTGCGGGAATTAACCCAACCTTTCCCAACACGAACTTAA

TAACGCCCTGGAGCACCTGGCTCAGAGTTCCCGAAAGCACCAAATCATCT

CTGCTAAAGTTCCTGGATGTCAAGAGTAGGTAAGGGTCTTCGCGTTGGCT

CCAATTAAACCACATGGTCCACCGCTTGGGGCGGCCCCCGGCAATTCATT

TGAGTTTTAACCTTGCGGCCGTACTCCCCAAGGGGGCGACTTAACGCGTT

AACTCGCGAAGCCACTCCTCCAAGGAATAACCTCCAAGTCGACATCGTTT

ACAGCGTGGACTACCAGGGTATCTAATCCTGGTTGCTCCCCAAGCTTTCC

ACCTGAGCGTCAGTCATTGTCCAGGGTGCCCCCCTCCCCCCCGGGATGTC

CTCTACAACACTACGCATTTCAACCGCTACACCTGGAAATTCTACCCCCC

TCCACAAGACTCTAACCTGCCAGTTTCGGATGGAGATTCCAGGGTGAACC

CGGGGATTTTCAATTCGACCTGACAGACCGACTGCGTGCGGCCTTACCGC

CCATAATTTCCGAATTAACGCCTTGGCACCCTTCGTATTACCGGGGCTGG

TTGGACGAATTAGCCGGGGGTCATCTGCGAGTAACGCCATCACAAGGGTA

TTTAACCTTATCGGCCTTCTCTCTCACTGAAAGGAGTTACCACCGAAGGA

CCTTCTTCCAACCACCGGCATGGCTGCGAACAGGCGTTGGCGCCAATTTG

TGCCATAATTCCCCCTTGGGGCCTTCCGGAAGGATCTCGGAACGGGGCTC

AATTTCCGGGGTGTGGATGGGGTAACCAT

>SYC28

CAGGAAGGGTTTCTTTGTTTAGACTTTCCCCCCAGTCATGACCACGAACG

TGGTAAGCGCCCTCCTTGCGGTTAGGCTAAAACATCTTGGCGAGACCCGT

TCCCAGGGGGGGAGGGGGGGGGTGTACAAGACCCGGGAACGTATTCCCCG

TGAATTCTGACTACGATTACTGCGATTCCGACTTCTGGAGTCGAGTTGCA

GACTGCGATCCGACTACGACTGGTTTTATGGGATTACGTGGTGTCGCGGT

TGGTTCCCTTTGTTAACCATTGTATGACGTGTGTAGCCCCACCTATAAGG

GCCATGATGACTTGACGTCATCCCCACCTTCCTCCGGTTTGTCACCGGCA

GTCCCCTTTGAGTGCTCAACTGAATGGATGAACTAATGGGAAAGGGTGCG

CTCTCTGTGGGACATAAACCCCCATCTTTCAACACAAGATGAGAACACCC

CTGTGCGACGTGTGTGTCAGATCTCTTTAAAGCACCAAACCATCTCTGGA

AAGTTCCTGCCATGTCAAAAGTAGGTAAGGTTCTTCGCGTTGCATCGAAT

TAAACCACATCATCCACCGCTTGTGCGGGTCCCCGTCAATTCCTTTGAGT

TTTAACCTTGCGGCCGTACTCCCCAGGCGGTCAACTTCACGCGTTAGCTT

CGTAACTGAGTCCGCTAAGACACAACCACCAGATCACATCGTTTACGGCG

TGGACTACCAGGGTATCTAATCCTGTTTGCTCCCCACGCTTTCGTGCATG

AGCGTCAGTGTTTGCCCAGGGGATCGCCTTCGCCATCGGTGTTCCTCCGC

ATATCTACGCATTTCACTGCTACACGCGGAATTCCATCCCCCTCTGCCAC

ACTCTAGCTTTGCAGTCACAAAGGCAGTTCCCACGTTGAGCGCGGGGATT

TCACCTCTGACTTACAAACCGCCTGCGCGCGCTTTACGCCCAGTAATTCC

GATTAACGCTTGCACCCTACGTATTACCGCGGCTGCTGGCACGTAGTTAG

GCGGTGCTTCATTTCTTGACAGTTACCGTCATGACACTGGAGCTATTAGC

CACCTTAGCTTTTCCTCTCTCCGCTCGAAGTGCATTTAAACCTGAGGCAT

CTATCATGCACGCGCATTGCTGCGATTCAGGCCTTGTCGCGCTATTGCAT

ATTCCCCACTGGCTGGCTTCCGTAGAGTCTGACGGGTTCTCAGTCAGTGG

CTGACTCTCCTCAGAACGTTACGGATTCGCCCGGCTATGTGGTAGGCCTA

ATCGTCA

>SYC29

TTGGGGGGGCCTAACCATGGCAAGTCGAGCGGTAGCACAAAGAGGGTTGC

TTCCTGAAGCGAGGGACGGGTGCGTAAAGAATAGGAGTCTGACTGGGAGT

GGGGGAGGGGGATCCCTTTTGAACGCTAGTACCGAATACGTATTACGGCA

GAAAACAAGGGGGGGTCCTTCTGGGCCCTAGCGCATGAATATGCGCCGAT

ATGGTTAGATCTTGAGGGAGAGGCTCACTCACGCGACGGACAATATCTGG

CCGGGGAGAGAGATCATGCACACTCACTCTGACTCAGAGACAGGACCCAT

ACCCCTAGGAGGGGTGGGGAATATTGATTGATCACTGTGGGCCTGATCCT

GACATGCCGCTCGCGTGTATAAGGACATCGGATTGTATATGACTTACAGT

TTCCGAGGAGGAAAGCGACCTAGTATAAAACACCATCGACTTAGGTTATT

CTCACAAAACAGGCGGGTATGTCCGCGTCACCCGCGGCGCACAAAGGGGG

GGGGCGTTAATCTTCTCACTTTACTGAGTAAAACGTCGCTGGTTTGCTGT

GTTGTATGAGGTGACATCCCCCGCCTCTCCCTGACTGCACTCCACACTGA

CTGACTACACTATAGTCGAGGATGGGGGAATTTCCTGTGGGGTGGAGCAA

TGATTCGCTTAGAGATGGAGCACTATCGCTGTAGAAAACCCCCTCCCCTA

CAACTAATCATGCTCTCCTGCGACAAGGCGAGCAGACAACACTAGATTAC

ATGCTGGGACACTCCGCTGTATATGATATCTACCCGTTGGAAGCGCTCAT

CTTGAGAGGGTGCTGCAGATCACATGAGTTGACCCCCCCCGCGGAGGACA

CCCGCCGCGCTAGTATTACTCTGATTTGATTGGGGGCGCGCCCCACACGC

GGGGGAGGGTGGTGTATTTCTATTGCACGCGAAGAAAATTACCTTGCTCC

TTCGTTTTCCTATGCAAAATTCTTTAACAAAATATTGTTTGCGTCCCTTG

AAAGAATACTGACAGAGGCTGGCTTCGACTGGTCGTTCACATCCGCTGCC

GTG

>SYC30

CGTAGGGTTCCTTTGGTACGACTTCACCCCAGTCATGAATCACAAAGTGG

GTAAGCGCCCTCCCGAAGGTTAAGCTAGGAAAGCGGGTGGGCAAGCCACT

CCCATGGGGGGACGGGGGGTGTGCACAAAGGCCCGGGAACGTATTCACCG

TATCATTCTGATCTACGATTACTAGCGATTCCGACTTCACGGAGTCGAGT

TGCAGACTCCGATCCGGACTACGACATACTTTGTGAGGTCCGCTTGCTCT

CGCGAGGTCGCTTCTCTTTGTATATGCCATTGTAGCACGTGTGTAGCCCT

ACTCGTAAGGGCCATGATGACTTGACGTCATCCCCACCTTCCTCCAGTTT

ATCACTGGCAGTCTCCTTTGAGTTCCCGGCCGAACCGCTGGCAACAAAGG

ATAAGGGTTGCGCTCGTTGCGGGACTTAACCCTACATTTCACAACATGAG

TTTACGACAGCCATGCAGCACCTGTCTCAGAGTTCCCGAAGGCACCAAAG

CATCTCTGCTAAGTTCCCTGGATGTCAAGAGTAGGGAAGGGTCTTCGCGT

TGCATCGAATTAAACCAAATGCTCCACCGCTTGTGCGGGCCCCCGTCAAT

TCATTTGAGTTTTAACCTTGCGGCCGTACTCCCCAGGCGGTCGACTTAAC

GCGTTACTCCGGAAGCCACTCCTCAAGGGAACAACCTCCAAGTCGACATC

GTTTACCGCGTGGACTACCAGGGTATCTAATCCTGTTTGCTCCCCACGCT

TTCGCACCTGAGCGTCAGTCTTTGTCCAGGGGGGCGCCTTCGCCACCGGG

ATTCCTCCGATCTCTACGCATTTCACCGCTACACCTGGAAATCTACCCCC

CTTCTAAAAGACTCTAGCCTGGCAGTTTCGAATTCAGTTCCCAGGTTGAG

CCCCGGGGATTTCACATCCGACTTGATAGACCGCCTGCGGCCGCTTTACG

CCCCATAATTCCGATTTAACGCTTGCACCCCTCCGATTATCCCCGGGCTG

CCTGGCACGAATTAGCCGGGGCCTTCTTCCTGC

>SYC31

AGGGTGGTACCTTGCTTACGACTTCACCCCAGTCATGAATCACACAGTGG

TAAGCGCCCTCCCGAAGGTTAAACTACCTACTTCTTTTGCAACCCACTCC

CATGGTGTGACGGGCGGTGTGTACAAGGCCCGGGAACGTATTCACCGTAA

CATTCTGATCTACGATTACTAGCGATTCCGACTTCATGGAGTCGAGTTGC

AGACTGCGATCCGGACTACGACGTACTTTATGAGGTTAGCTTGCTCTCGC

GAGTTGGCTTCTCTTTGTATACGCCATTGTAGCACGTGTGTAGCCCTACT

CGTAAGGGCCATGATGACTTGACGTCATCCCCACCTTCCTCCGGTTTATC

ACCGGCAGTCTCCTTTGAGTGCCCGACCTAATCTGTGGGTAACAAAGAAA

AAGGTTGGGCTCCGTGAGGGAATTAACCCAACATTTCCCAAAACGAATTT

TAGAAAGCCATTGAAGGCCTTGTTCAAAGGTTCCGAAAGGACCCATCCTT

CTCTGGAAAGTTCTCTGGATGTCAAAAGTAGGTAAGGGTCTTCCCGTTGG

CTTCAAATAAACCACATGGCCCACCGGTTGGGCGGGCCCCCGTCCATTCA

TTTGGAGTTTAAACTTGGCGGCGGTATCCCCAAGGCGGCGATCTAAAGCG

TTAGCTCCCGAAGCCACGCCTCCAAGGCACACCATCGCTATCGAACTCCT

TTACAGGCTGGAATAACAAGGTAACTAAACCTGGTTGGTCCCCACGGTTT

CCAACTGAGCGGCAGTCATTGTCCAAGGTGGCGCCTTCCCCACCTGGAGT

CCTCCCCATCTCTACGCATTTCCCCGCTACACCAGGAATTCTACCCCCCC

CCTACACGACTCTAGCTTGGTCATTTCTAAGCAGGTTCCCGTTAGAGCCG

GGGATTTCTCATCTGAATTAACCAACCCACTGTGTGCGCGTTTAGCCCAA

AGAATTCCCATAAAGCTTGACCCTCTCGTATAACCCGGGCTGCTGGACGA

GTTAGACGGGGCTTCTTTCTGCGAGTAAGCTATGACAAGTGCTATTAAAG

CAAATGCCTTTCTCCTCCCACGAAGGTGCTTTTACAAACCTCAAAGACTT

TCTTCACACACGGCCGGAGTGGGCTTGCGTACAGGCTGTGTCCGCATGTG

GCCAAAATGTCCCCACTGGCGTGCCCTCCCGTAGGAGAGTTGGGAACGGT

GTTCTCAAGTCAGTGGCTGGATTCCTCTCGACACGTTAGGGAATCGTCCG

CCCATAGTGTGGAGAGCATA

>SYC32

ACGCTGTGGTGGGCATACCATGCTTGTCGAGCGTGTAGCGCAGGGGAGCT

TGCTCTCTGCGTGAGGGGCGGAGGACGGGAGAGTAATGTCTGGGACCTGC

CTGATGGAGGGGGATATTACTGAAAACGGTAGCTAATACCGCATAACGTC

TACAGACCAAAGTGGGGGACCTTCGGGCCTCACGCCATCAGATGTGCCCA

GATGGGATTAGCTAGTAGGTGGGGTAATGGATCACCTAGGCGACGATCCC

TAGCTGGACTGAGACGAGGCCCACTCCTACTGGGACTGAGCCCCGGCCCC

CACTACAACGGGAAGGGGCTATGATTGATATTGGCGCGAAAGCCCGAGCC

TGATGCAACCCTTCCGCGTGTGTGAATAAGGCCTTAGGGTTGTAAAGCAC

TTTCAGCGAGGAGGAAGGGTAGTGTGTTAATAGCACATTGCATTTTTTGT

TACTCGCAGAAGAAGCACCGGGTAACTCCGTGCCAGCAGCCGCGGTAATA

CGGAGGGTGCAAGCGTTAATCGGAATTACTGGGCGTAAAGCGCACGCAGG

CGGTTTGTTAAGTCAGATGTGAAATCCCCGCGCTTAACGTGGGAACTGCA

TTTGAAACTGGCAAGCTAGAGTCTTTGTAGAGGGGGGTAGAATTCCAGGT

GTAGCGGTGAAATGCGTAGAGATCTGGAGGAATACCCGGTGGCGAAGGCG

GGCCCCCTGGACAAAGACTGACGCTCAGTGCGAAAGCGTGGGGAGCAACA

GGATTAGATACCCTGGTACTCCACGCTGGACACGATGTCGATTTGGATGT

TGTGCCCTTGAGCGTGGCTTCCGGAGCTAACGCGTAATCGACCGCCTGGG

GAGTACGGCCGCAAGGTTAAACTCAAATGAATGACGGGGGCCCGCACAGC

CGTGGAGCATGTGGTTTAATTCGATGCAACCTGAAGAACCTTACCTACTC

CTGACATCCAAAAACTTTCCAGAGATGGATTGGTGGCCTTCGGAATCCTG

AACAGTGCTGCATGGCCTGCCGCACCTCGTGTTTGGAACTGTTTGGGGTT

AAGTCCCCGCCACAGACCGCCAACCCTTTATCTCTTATGGTTTGCCAGCG

AATTCCGGCTCGGGGAACTTCAAGCGGGAACTGGCCGGTAATAAACTCGT

GAGAAAGGGGGGGGGATAGGCACCTCTAGAGGCCTC

>SYC33

ACATTCGGTTGCTTTCACATGCAAGTCGAACGGTAACACGTGATAGGATG

CTGACGAGTGGAGAAGGGGTGAAGAGAAGCGGAGCGTGGGAGCAGTGGGG

GAGAGGGGGAAAATTAGGGATCGGCCGTTGACATCTACGGATGAAAGCAG

GGGACCGTCGGGCCTTGTGCTACTACACCGGCTGATGGGACATTATGGAA

TTGGCGGGGTAAAGGCTTACCGAGCCTGCGATGTGACGATGGCCAGATAG

GATGACCAGCCGACCTGGCACTGAGACACGGACAAAACTCCTACAGTCCG

ACGCGAGGGGGAATTTTGGACTATGGGCCAAAGCCTGATCCCCCGATGCC

GCCTGGACGATGAAGGGCCTCAGGTTGTAAACTGCTTTTGTACTTTCCGA

AAAGACTGGGGCTAATATGTCCGGGTCATGACGCATTCATAATAATAACC

AACGACTAACTACGTGCCTCCATCCCCGGTAATACGTAAGGTGCAAGGGT

TAATCGGAATTACTGAATTACTGGGGTGCGCGCGCGGTTTTGTAATACGT

TGGTGAAAACCCCGGGCTCAACCTGGGAACTGCCATTGTGACTGCAAAGC

TAGAGTGCGGCAGATTGGGATGGAATTCCGCGTTCCAGGTGAAATGCGTA

GATGTGCAGAGGAACACCGATGGCGAAGTGGATCACGTGGGCCCGTGCTG

ACGCTCATGCACTAAAGCGTGAGAGAGCAGACGAGATTACATACCCTGAT

ACTCTGCTCCCTAAACCAGGTCACCTGGTTGATTTGTCTGATCTGACTCT

GAAGCGAGGCTACCGCATGTAGTTGACTGCCTGGACAGTACGGGCACTAC

GGTCGACACTGATAAGATTTGACTGAGATCCCGCACAAGCCGGCAGCATG

CTGTGGATTATGTCGATGCATCGCGAAACAACCTTACCCAACCTTTGCAC

ACTCCTAGGAAATTCTACCAGACATTGCTATAGAGGGCATTGATGGCATC

TGCACACTCGTGCCGCATGGCTGATCGTCTGCTCGTCTGTCTCGAGATTG

TGAGGTGTAGTCCTCAATCCAGGCCACAACACTTGACCCCTTATGCCTTA

CGTGCGTCGAATTCCTCTATGGAAACCTGCCCGGAGAACTACCGGGAAAA

GTGCGGGGGGGGGAATGGACGTGCAAGTCCTCTATGAGCCATTTATGGGT

GCCGGTCCACACAGGCCTAACCACGTGGCGTGACCAGAAGGTTACAACCA

CGCGAGAGGCTGACG

>SYC34

ATTCAACAGGTGGTAGAAGCGATTCTCTCTGGGGGACAACGGGTGAAAAA

AAAGAGAAAGAGCCTGGTAACAGTGGGAGAGAGGGGGATCTCTACTGGCT

ACGGTAGCTAATACATCTTAGGATGTACGGACGGGAGTGGGGGACCTTCT

GGCCTCACACCGTCTGATGTGACAATATGGAATTAGCGAGGTAGTGGCTT

ACTGGCTCTCCTATCCGACGATGGCCAGCTGGGATGACCGGACGACCTGC

CACACTGAAACTGACAAAACGCCTACACTCCGACGGGATGCGGCATTGTG

GAATATTGCCCAATGCCCGCTCCCCTGATGCAGCCTTGCCGATTGTGGGC

CTAAGGCCGTAAACTGGTATTGTACTTTCCGAAAGGACGGAGGCTAATGT

GTCCAGGTCATCTTGGATTGATGTTACTCACACACGGCGCAGTAGGTAAC

TCCGTGCCCGCAACCACGGAAGGTGCGAGGGTGCATCTGTTTATCTGAAT

TACTGGCGGTAAAACGCACGTTTGCAGTTTGTTAATGCAAACCCCAAATC

CCCCCGCTTAAACTGCCAACTGCATTTGAAACTGGCAGTGCTAGAGACTG

GGATGGGGATTGTAGAATTTCCCGGTGAAACGGTGAAAATGCGCAGAGAT

CCACAGAAATACCAGTGGCTCCCGTGGGCCCCCTGGAGACACACTGACGC

TCAAGTGCGAGGAGCAAGGGAGATACATGCTTAGATACCCTGCGAGTCCA

CGCAGGTCACCAGGTTGATTTGGATGTAGTGACTTTGAGACGAGGCTTAC

GCATGCAACGCACTACCTCGACCGCCCGGGCACTACGGTCAAACTGTTAA

AACTCGAATGAAATCCTCCACAGCCGCGCGATGAGGTGGATCATGTCGAT

TGAATTCCATGCACGCTTACCAACCTTTAGCTTACTGCTGGAACTTCTAC

AAGAACATTGCTATAGATGGCGACTTGAGTACCATTCT

>SYC35

AAGGCGGGGGCTAAACATGCAGTCGAGCGGCTGCGGGGAGGTTGCTTGTT

ACTTTTGCGGGGGAGGGGGGGAAGAAGGAGAAATGTCTGGGAGATAGCGG

GTCGAGGGGTACCTAAGGTGCACACGACCGGTAATACCGCAACCGCCCTA

CGGGGGAGAGCTTGGGACCTTGGGCTCTTGCACGATTGTATGTCCCCATG

GGGAATTAGCTTGTTGGTGACTCACCGGCGCGACGAGGCGAAAATCCCTA

GCTGGGATGATAAGATGATCTGCCACTGTGGCACTGACACGACTCCCACA

CTCCTACGGGAGGGGGCATAGGGGAATATGGCACTGTGCCTGAACCCCTG

ATGCCGCCATGGCGCGGGAGGGAATAAGGTTTTCGGGTTGTTTAACTTTT

TCGGAAAGGAAGAAAGGTTGATACCTGATGTTTTGACGTTGTCACGATAC

TCACAAAAGACGCACTCTGTGCCTCCGTGCCCGGTAATACGGTAATAGCG

AGGGTGCATCCGAATTACTGAATTACTGGGCCTCGTGCGCACTTTGTTAA

TTTGAATGTGAAAATGTGAAAGCCCCGCTCTCAACCTGGATCATTGCCTG

GAAAACTGTCAATATAGAATATTGTGGAGGAATTTCCTGTTTCGCGTGTA

ACTGTGAAATGTATAGAGATATGGAAGAATACGAAGTGCGAAACCTGCAC

CCCTACAGAAACACTGACTCTCAAGTGCTGAAGAGTGGCAGGATACAAGA

TTAGATACCTGATACCCCACACCATGTCCACTATCCATTGGAATCCCTGT

GTCTTTGAGACGTGGTCTTCACGAGCTAACGCGTACAGTCTGCCGCCTAG

GGCCTAAGGCCTAAACTTAAATCTCATATCGATGTGACCGGGCCCCCGCA

CGACCATGGAGTTTGATTTCTAATCTTCGACGCACACGTAAAAACGTCTT

TGCACGTAAACAAGTCTTCCATACATGGAATAGATGCCGTCGGGCATTCG

AACATCAGAAGCTGCGTGGCTTGCATGCCTGCTCGTCGTCCTTGAGATGG

TGGAGATTAGTTCTAAATCCCGCCCCAACCTTCGACCCTCTAGTGTCCTT

TGCTTCCTATCAGTAAGTAGCTCGGAAGCCTACCTGCCAAGATGGCAGAG

CCGATTAAACACGGGAGTGGAAAGTGAGAGCCTATAAGCCTTCCCAAGGT

CCCTTAGGCGCCCCTA

>SYC36

TTCTTTGGGCCTAACCTGCCAGGTCGAGCGGGAGACAGGGGAGCGTGCTC

TATGGGCGGCAGAGGGGGAAGGAAAAAGATGACTGGGATGTGCCTGAGGG

AGGGGGATTCCTACTGGACGCGGTACCGATACCGCATAACGGCAAAGGAC

CAGAGAGCGGGAACCTCTGGCCTCACGCAATCAGATGTGCCCAAATGGGA

TTATGGAGAAGGTGGGGTACTGCCTGGCCAAGATCACGATCCCTACCTGG

ACTGAGAGGATGACCACTGGCACTGAAACTGAGACCCGATCCCTACTCCA

AGGGGAGGCAGCGAAGGGTGATATTGGACAATGGGCGTGAGCCTGACGTG

CCCCTGGCGCGAGGGAGAACAACGCATTATGGTTGTATTGCACTTTCAAC

GAGGAGGAATGGTATTGTGCTAATAGCACATTGCATTGACGATACTCGAA

GAACAGGCACCGGCTAACTCCGTGCCAGCAGCCGCGGTAATACGGAGGGT

GCAAGCGTTAATCGCAATTACTGGACGTAAAGCGCACGCAGGCGGTTTGT

TAAATCAGATGTGAAATCCCCGCGCTTAACGTGGGAACTGCATTTGAAAC

TGCTAAGCTACAGTCTTGTTGGTGGGGTAGAATGCCAGGTGTAGCGATGA

AATGCGTAGAGATCTGAACGATACCGGAGGGCGAGGCGGCCCCCTGGACA

AACACTGACGCTCAGGTGCGAAAGCGTGCAGAGCAAATAGATTACATACT

CTGCCACGCCACACTGTATGCCATGTCGACTTGGAAGTTGTGCCCATGAA

GCGGGCTTCTGAGCTATCGCGTTGAAATCCACCGCCTGTCGAGTCCCGCC

GGTAAGACTCAAACTCATTGAATTGACCCCGCCCCGCGGAGCAGCAGGAG

CATGTAGTTTAATTCAATGCGACGCACATTACCTTACCTACTCTTGACAT

CAATTTAACTTATCATGAAATGGATTCGTCCCTTACGCTAACTTCTGAGA

CTGCTGCGGCATGGCTTGTCGTCAGTCGTGGTAATGTAATGGTTAGGTTC

ACAGTACCCGCACCAACCCCATAGCCCTTTATCCTTTTCTGTCTCCATCT

AATTCCGTCCGTCTAACTCGAAAGCTGACCTTGCCGAGTAACTAAACCGG

AAGGTAAGGGTAGGAAATTAAAGCTCAATCTCCTCCCCTGGTCTAT

>SYC37

ACGGGCGCCTGGCCTCACCATGGCGCTTCGAGCGGCTGCCGGGAGGTAGC

TTGTTGACTCAGCGGGGGAGGGGTGAGAGATAAAATCGAGTCTGGCAGAT

AGCGGGGGAAACGGTTCTAAAGGAACGCTCGTATGGTATAAGTCCTACGG

GATAAAGCAGGGAACCTTCGGGCCTTGCGCTATCGCATGAGCCTATGTCG

GATTATGTAGTTGGTGAGGTAATGGGTCACCGGGGCGACGAGCCGAAACT

GGTCTGATAGGATGATCAGACGCACTGGAACTGAGACACGGACCCAACTC

CTACAGTAAGCAGCAGTGGGGAATATTGGACAATGGGCGAAAGCCTGATC

CAGCCATGCCGCGTGTGTGAAGAAGGTCTTCGGATTGTAAAGCACTTTAA

GTTGGGAGGAAGGGCAGTAAGCTAATACCTTGCTGTTTTGATTTTTCCTA

CAGAATAAGCACCGGCTAACTCTGTGCCAGCAGCCGCGGTAATACAGAGG

GTGCAAGCGTTAATCGGAATTACTGGGCGTAAAGCGCGCGTAGGTGGTTC

GGTTAAGTTGGATGTGAAATCCCCGGGCTCAACCTGGGAACTGCATCCAA

AACTGGCGAGCTACAGTATGGAAGAGGGTGGAGGAATTTCCTGTGTAGCG

GGTGAAATGCGTAGATATAGGAAGGAACACCAGTGGCGAATGCGACCACC

TGGGCTCATACTGACACTGAGTGCGAAAGCGTGGGGGAGCAAACAGGATT

AGATACCCTGTTAGTCCACGCCGTAAAACGATGTCCACTAGCCGTTGGAA

TCCTTGATATTTTAGTGGCGCAGCTAACGCATTAAGGTTGACGGCTGGGG

AGTACGGCCGCAGGTAAACTCAAATGATTTGACGGGTGCCCGCACAACCG

TGGAACATGTGGTTTAATTTCGTAAACACGCGAAAACCTTAACACGTCCT

TGGCATCAAGAACTTTGCAAAAAATGGATTGATGCCTTTC

>SYC38

CCTGGTGGCTGCCTTGGTTCGACTTCACCCCAGTCATGAATCACACCGTG

GTAAAACGCCCTCCCGAAGGTTAAACTAAAACATCTTAGTGCAACCCACT

CCCATGGTGTGACGGGCGGTGTGTACAAGGCCCGGGAACGTATTCACCGT

AACATTCTGATCTACGATTACTAGCGATTCCGACTTCACGGAGTCGAGTT

GCAGACTCCGATCCGGACTACGACATACTTTATGAGGGTAGCTTGCTCTC

GCGAGTTGGCTTCCCTCTGTATATGCCATTGTAGCACGTGTGTAGCCCTA

CTCGTAAGGGCCATGATGACTTGACGTCATCCCCACCTTCCTCCGGTTTA

TCACCGGCAGTCTCCTTTGAGTTCCCGACATTACGTGCTGGCAACAAAGG

AAAAGGGTTGCGCTCGTTGCGGGACTTAACCCAACATTTCACAACACAAT

TTGACGACAGCCATGCAGCACCTGTGTCAAAATTCCCGAAGGCACTAACC

TATCTCTAGAAAATTCTCTGGATGTCAAAGCCAGGTAAGGTTCTTCGCGT

TGCTTCGAATTAAACCACATGCTCCACCGCTTGTGCGGGCCCCCGTCAAT

TCATTTGAGTTTTAACCTTGCGGCCGTACTCCCCAGGCGGCCAACTTAAC

GCGTTAGCTCCGGAAGCAACGTCTCAAGGATACACCCTCCAAGTTCGACA

TCGTTTACGGCGTGAACTACCAGGGTATCTAATCCTGTTTGCTCCCCACG

CTTTCGCACCTGAGCGTCAGTATTTTGTCCAGGGGGGCCGCCTTCCCCAC

CGGTATTCCCTCCCAGATCTCTACGCATTTCACCGCTACACCGGGAATTT

CCACCCCCCTCTACAAGACTCTAGCTGGCCAGTTTTGATGCAGTCCCAAG

GTTGAGCTCGGGGATTTCCCAT

>SYC39

TGGGGGGGGTCTACAATGCGCTCGAGCGGTCGACTGGGAGCTTGGTCACG

GGCGAACAGGGTAATCCCATCTGGTCACATCTGATGGCCTGAGGGATGAA

GGGGGACACTTTGGTCTTGGGACGCTTGCGGGATTAACTACCGTTTACCG

AAGTGATCCCCCTCCATCAGGCAGTTTCCCAGACATTACTCGATGGTCCG

CCCTTGTTGCCCAGGAACGGGTCACCAGGGGGACGATCCCAAGCTGTGTG

GAGAGGATGGCCGCCCCCACTGTATCTGAGCCCTGATCAAACTCTCTAAG

GAGGCCCAGTGGGGAATATTGTACACTGGGCGCAAGCCTGATGCACCCAT

GCCGCGTGTGTGAAAAAAGCCTTCGGGTTGTAAAGTACTTTTTGCGAGGA

GGAAAGGGATGTGGTTAATAATCGCATTCATTGTTTTTATTTTTCAGAAA

AAACGCGGGGGGGGGGGGGGTGCCACCAGCCCCGGTAATACGCAGGGGGG

TAAGGCGTTAATCGCAATTACTGGGGGGGGAAGCGCACGCAGGCGGTCTG

TCAAGTCTCATATGAATCCCCGGGCTCTACCTGTGAACTGCGTTCTAAAC

TGTCGGGCTATAGTCTTGTGTAGAGGGGTATAAATTCACGTGTATCGGTG

AAAATGCGTATAGATCTCCAGGAATACCGCTGCCGAAAAAGGGCCCCCCC

GCGACAAAAAACTGACGCTCAGATGCTGCAAACCGTGGGGAGCAAAACAG

GAGTAATATAACACTGGTGAGTCCACCCTGTAAACAATGTCGACATTGCA

GATGTTTCCCCTCGAGGAGGGGCTTCCCGAGCTAAACACGCT

>SYC40

GGCCGGGGGGTCTTTGGTTACAATTCCCCCCAAGTCATGAATCACACCGT

GGTAAACGCCCTCCCGAAGGTTAAACTAAAACGGCGGGGGCAACCCACTC

CCATGGGGTGACGGGCGGTGTGTACAAGGCCCGGGAACGTATTCACCGCA

ACATTCTGATTCGCGATTACTAGCGATTCCGACTTCACGGAGTCGAGTTG

CAGACTGCGATCCGGACTACGATCGGCTTTTTGGGATTAGCTCGCCCTCG

CGAGTTGGCATCCCTCTGTACCCACCATTGTAGCACGTGTGTAGCCCTGG

CCGTAAGGGCCATGATGACTTGACGTCATCCCCACCTTCCTCCGGTTTGT

CACCGGCAGTCTCCTTTGAGTGCCCAACATTACGTGCTGGTAACTAAGGA

CAAGGGTTGCGCTCGTTGCGGGACTTAACCCAACATCTCACGACACAATC

TGACGACAGCCATGCAGGACCTGTGTCATAATTCCCGAAGGCACTAATCC

ATCTCTGGAAAATTCTCTGCATGTCAAGGCCAGGTAAGGTTCTTCGCGTT

GCTTCGAATTAAACCACATGCTCCACCGCTTGTGCGGGCCCCCGTCAATT

CATTTGAGTTTTAACCTTGCGGCCGTACTCCCCCAGGCGGTCGATTTAAC

GCGTTTAGCTCCGCAAGTCAAATCTCAAGGACACCAACCTCCAAATTGAC

ATCGTTTACGGCGTGGACTACCAGGGTATCTAATCCTGTTTTGCTCCCCC

ACGCTTTCGCACCTGAGCGTCAGCTTTGTCCAGGGGGGGCCGCCTTCGCC

ACCGGTGTTCCTTCCGATATCTACGCATTCACCGCTACACATGAAATTCC

ACCCCCCCTCTACATACTCAAGCTTGGCCGT

>SYC41

AGCTATAGGTGTTTGGTTAGACTTCCCCCAGTCATGAATCACACCGTGGT

AAACGCCCTCCCGAAGGTTAAACTATAAAGGGTGGTGCAACCCACTCCCA

TGGTGTGACGGGCGGTGTGTACAAGGCCCGGGAACGTATTCACCGCAACA

TTCTGATTTGCGATTACTAGCGATTCCGACTTCACGGAGTCGAGTTGCAG

ACTCCGATCCGGACTACGATCCGCTTTGTGGGATTAGCTCACTCTCGCGA

GTTGGCAACCCTCTGTACCCGCCATTGTAGCACGTGTGTAGCCCTGGCCG

TAAGGGCCATGATGACTTGACGTCATCCCCACCTTCCTCCGGTTTATCAC

CGGCAGTCTCCTTTGAGTGCCCACCATTACGTGCTGGTAACTAAGGACAA

GGGTTGCGCTCGTTGCGGGACTTAACCCAACATCTCACGACACGAGCTGA

CGACAGCCATGCAGCAGCTGTGTCATAATTCCCGAAGGCACTCATCCATC

TCTAGAGAATTCTCTACATGTCAAGGCCAGGTAAGGTTCTTCGCGTTGCA

TCGAATTAAACCACATGCTCCACCGCTTGTGCGGGCCCCCGTCAATTCAT

TTGAGTTTTAACCTTGCGGCCGTACTCCCCAGGCGGTCAACTTAACGCGT

TAGCTGCGGAAGCCACATCTCAAGGACACCAACTCCTAATCGACATCGTT

TACGGCGTGGACTACCAGGGTATCTAATCCTGTTTGCTCCCCACGCTTTC

GCACCTGAGCGTCAGTCTTTGTCCAGGGGGCCGCCTTCGCCACCGGTGTT

CCTCCAGATCTCTACGCATTTCACCGCTACACCTGAAATTCTACCCCCCT

CTACAGTACTCTAGCTTGACAGTTTTAGATGCAATTCCCAGGGTTGAGCC

CGGGGCTTTCACATCTAACTTATCAAACCGCCTGCGTGCGCTTTACGCCC

AGTAATTCCGATTAACGCTTGCACCCTCTGTATTACCGCGCTGCTGGCAC

GGAGTTAGCCGTGCTTACTTCTGCCAGTAACGTCCACAACTGAAACGTTA

TTAGCTATCATGCCTTTTCTCTCACTGAAAGATGCTTACAATCCGGAAGG

CGTTCTTCAACACCGCGCATGGCTGATCAGGCTTTCGCCCGAATGTCAAT

ATTGCCCAACTGCCTGCTTCGTAGGAGCCTGGGACGGGGTCTTCAGTCCA

GTGTGGACTTGAT

>SYC42

GGGGGGGGGGCCCTGGGTTTCACTTTCCCCCAAGTCATGAATCACAAAGG

TGGTAAAGCGCCCTCCCGAAGGTTAAGCTAAAACAAAATTTTGCAAACCA

CTCCCATGGGGGGACGGGGGGGGTGTAAAAGGACCGGGAACGTATTCACC

GTAGCATTCTGATCTACGATTACTAGCGATTCCGACTTCATGGAGTCGAG

TTGCAGACTCCAATCCGGACTACGACGTACTTTATGAGGGCCGCTTGCTC

TCGCGAGTTCGCTTCTCTTTGTATACGCCATTGTAGCACGTGTGTAGCCC

TACTCGTAAGGGCCATGATGACTTGACGTCATCCCCACCTTCCTCCGGTT

TATCACCGGCAGTCTCCTTTGAGTTCCCGACCGAATCGCTGGCAACAAAG

GATAAGGGTTGCGCTCGTTGCGGGACTTAACCCAACATTTCACAACACGA

TCTGACGACAGCCATGCAGCACGTGTGTCAGAGTTCCCGAAGGCACCAAT

CCATCTCTGGAAAGTTCTCTGGATGTCAAGAGTAGGTAAGGTTCTTCGCG

TTGCATCGAATTAAACCACATGCTCCACCGCTTGTGCGGGCCCCCGTCAA

TTCATTTGAGTTTTAACCTTGCGGCCGTACTCCCCAGGCGGTCGATTTAA

CGCGTTAGCTCCGGAAGTCACGCCTCAAGGGCACAAACCTCCAAATCGAC

ATCGTTTACAGCGTGGACTACCAGGGTATCTAATCCTGTTTGCTCCCCCA

CGCTTTCGCACCTGAGCGTCAGTGTTTGTCCAGGGGGCCGCCTTCGCCAC

CGGTATTCCTCCACATCTCTACGCATTTCACCGCTACACCTGGAATTCTA

TCCCCCTCTACAAGACTCTAGCTTGCCAGTTTCAAATGCAGTTCCCACGT

TAAGCGCGGGGATTTCACATCTGACTTACCAAACCGCCTGCGTGCGCTTT

ACGCCCAGTAAATCCGATAACGCTTGCACCCTACGTATTACCGCGGCTGC

TGGCACGGAGGTAGCCGGTGGCTTACTTCTTGCGGTAACGGTCATGCAAT

GTGGCTATAACCCACCTAGCCTTTCCTCCTCGACTGAAAGCGCTTTAACA

ACCCTAAGGGCTTTTCATCCCAGACCGCGGCATGGCGTGGAATCAAGGCA

TGGGCCCCAATGGGACATAATTTCCCCCATGGGTGTGCACTCCCCGTTAA

GAGATCCTGGGAACGCTGTGT

>SYC43

GGGGGGGGTTGTTGGTTAGAACTTCCCCCAGTCATGATCACAAAGTGGGA

AGCGCCCTCCCGAAGGTTAAACTAAAAACATCATTTGAACCACTCCCATG

GTGTGACGGGGGCCGTGTAAAGGCCCGGGAACGTATTCACCGTAACATTC

TGATCTACGATTACTAGCGATTCCGACTTCATGGAGTCGAGTTGCAGACT

CCAATCCGGACTACGACGTACTTTATGAGGGCCGCTTGCTCTCGCGAGTT

GGCTTCCCTTTGTATACGCCATTGTAGCACGTGTGTAGCCCTACTCGTAA

GGGCCATGATGACTTGACGTCATCCCCACCTTCCTCCGGTTTATCACCGG

CAGTCTCCTTTGAGTTCCCGACCGAATCGGTGGGAACAAAGGAACAGGGG

TGCGCTCGTTGCGGGAATTTACCCCACATTTCCCATAATTTTTTTGAAGA

AAGCCCTTGCGCGCCGGGCTCAAAATTCCCGAAGGGCCCCATCCCTCTCT

GGAAAGGTCTCTGGATGTCAAAAGGATGGAAGGGTCTTCCCGTTTCATCC

AAATAAAACCAATGCTCCCCCGCTTGGGCGGGCCCCCGGCAATTTCTTTG

AATTTTAACCTTGGCGGCGTACTCCCCCGGGGGGCGAATTAAATGGTTAG

CTCGGGAAGCCACACCCTCAAGGGCACAACCTCCAAATCGAATCCGTTAC

AGCGTGGAATACCAGGGTATCCAATCCCGGTTGGTCCCCACGGCTTCCCA

ACTGAGCGGCAGTCATCGTCCAGGGGGCCGCCTTCGCACCGGTATTCCTC

TAGATCTCCACGCATTTCACCGCTAACCTGGAAATCCACCCCCCCTCCAC

ACGACTCTAGCTTGGCAGTTTCAGATGCAGTTCCACGGTAGCGCGGGGAT

TTCACATCTGACTTAACCAACCCCCTGCGTGCGCTTTACCCCCAGTAATC

CGAATATAAGCTTGCGACCCTCCGTGATTTACCGCGGGCTGGCGGGCACG

GAATTAGCCCGGGGGCTCATTCTGCGAGGAACATCAAATGAAAATGTGCT

ATAAAAGCACTATGCCTTCCTCCCTCCGCTGAAAGAGGGCTTTTAACACA

ACTCAAGGACTTTCTTCATACACACCCGCGGCGCAATGGGTTGCGTAAAG

GGGTGTGATTCGCAATGGGGCATAAATTTCCCCTCGTGGGTGGCCGCCCC

CACCGTAGGAAAGAATCTTGGA

>SYC44

GTACATTGCCAAGTCGAGCGGGCAGACAGGAGCTTGCGCTTGAGTATCGG

GGGAGGGTGGGGAAGAAGGCTCTCTGTTGGGAGTGGGGGAAAAGATTTAA

TGGAACGCTAATACAGATCACGTATTACGGGAAAAAGCAGGGGGGGGTCA

GTCCTTGCTCTATCCCATGAGCCTGGGTCGGATTAGCTTGTTGGTGGTGA

GTGATTATGCAATGCAACGATAATAACTGGTCTGGGAGGGAGATCAAGCA

CACTGCACTGATCAGAGACCGGACTCATACCTATATGAGGAGGGGCGAGA

TTAATCATTGGTCGGAAGCCTGACCCAGCCATGCCGCGCCGTGAATAAGG

TCATCGGATTGATATCACTTCAATTTGGGAGGAGGACAACGCGTTAATTC

CTATGCTCACCGACGTAAAATTTCAATGAGAATACGTCGGGTCCGTGCTA

CCACCGCGGTAATAAAAGTGGGCTGCGTAGCTTTAATTACTGGACGTAGG

CACCGTAGTGGCTCGTTATTTGTATGAGATGACACCCCCCTGAACCTGGT

AAGACATCCTATACTGGTCAGCTACTATACGTTTAGGGGGGGGACTGATT

CCGGAGCGCGAAGAGATTGATATAATAAGGAAAAACACTGG

>SYC45

GGGCTGCGGCCTTTGGTATCGACTTCCCCCCCCGTGCTGAATCCCAAAGG

GGGTAAGCGCCCTCCCGAAGGGAAATAAAAAGGGTAAGGGGGACACTTCA

TGGTGGAGGGGGGTCCTAGAAGGACCGGGAACGTATTACCGGGGATTCTG

ATCCCCGATTATTAGCGATTCCAAACTCACGCGTCTAGTTGAGACTCCGA

TCCGGACCACGACATACTTTAGAGGGGGGGGTGCTCTCCCGCGGGGGTTT

CTCTTTGTATGGCCATTGTATGAGGGGGTAGCCCTACCCGTAAGGGCCAT

GATGACTTGACGTCATCCCCCCCTTCCTCCAGTTTGTCACTGGCAGTCTC

TTTTGAGTTCCCGGCTGAACGGCTGGCAAAAAAAGAAAAGGGGTGCGCCC

GCTGCGGGATTTTTCTCAACATTTCGCCCCTATAAGAGAACACAGCCATG

CTGCACCTGTCCCAGAGTTCCCCAAGGCTCCAAAGAATCTCTGCTAACGT

CTCTGGATGTCAAGAGGTAGGAAGGGTTTTCGCGTTGCATCAAATTAAAC

CACATGCTCCACCGCTTGTGCGGGGCCCCGGGCATTCATTTGAGTTTTAA

CCTTGCGGCCGTACTCCCCAGGGGTCGACTTAACGCGTTAGCTCCGGAAC

CTCTCATCAGGGGAACA

>SYC46

AAGCGGGTTCTTGGTTAGAATTCCCCCCAGTCAGGAACCCACGGGGGGAA

GCGCCCTCCTTGCGGTTAGGTAAAAAGGGATGGGAGACCCGTTCCCAGGG

GGGGAGGGGGGGCGTGAACAAGACCCGGGAACGTATTCACCGCGGCATGC

TGATCCGCGATTACTAGCGATTCCGACTTCACGCAGTCGAGTTGCAGACT

GCGATCCGGACTACGACTGGTTTTATGGGATTAGCTCCCCCTCGCGGGTT

GGCAACCCTCTGTACCAGCCATTGTATGACGGGTGTAGCCCCACCTATAA

GGGCCATGAGGACTTGACGTCATCCCCACCTTCCTCCGGTTTGTCACCGG

CAGTCTCATTAGAGTGCCCAACTAAATGTAGCAACTAAAGACAAGGGGTG

CGCTCGTTGCGGGACTTAACCCAATATCTCACGACACGAGCTTTTAACAG

CCATGCACCACCTGTGTGCAGGATCTCTTTCGAGCACGAATCCATCTCTG

GAAACTTCCTGCCATGTCAAAAGTGGGTAAGGTTTTTCGCGTTGCATCGA

ATTAAACCACATGATCCACCGCTTGTGCGGGGCCCCGTCAATTCCTTTGA

GTTTCAACCTTGCGGCCGTACTCCCCAGGCGGTCAACTTCACGCGTTAGC

TTCGTTACTGAGAAAACTAATTCCCAACCACCAGTTGACCTCGTTTTAGG

GCGTGGGACTACCAGGGCATCTAATCCCTGGTTTGCTCCCCCACGCTTTC

GTGCATGAGCGTCAAGTACTAGGTCCAGGGGGATTGCCTTCGCCATCGGG

TGTTTCCTCCGACATATCTACGCATTTCACTGCTACACGTGGGAATTCCA

TCCCCCTCTACCGGTACTCTAGCCATGCCAGTCACAAATGCAGTTCCCAG

GGTTGAGCCCGGGGATTTCACAT

>SYC47

TGCCTGTAGGTCCTTGGGTTACGACTTCACCCCAGTCATGATCACACCGT

GGTAAACGCCCTCCCGAAGGTTAAACTATCTACTTCTGGTGCAACCCACT

CCCATGGTGTGACGGGCGGTGTGTACAAGGCCCGGGAACGTATTCACCGC

GACATTCTGATTTGCGATTACTAGCGATTCCGACTTCACGGAGTCGAGTT

GCAGACTGCGATCCGGACTACGATCGGTTTTGTGAGATTAGCTCCACCTC

GCGGCTTGGCAACCCTCTGTACCGACCATTGTAGCACGTGTGTAGCCCAG

GCCGTAAGGGCCATGATGACTTGACGTCATCCCCACCTTCCTCCGGTTTG

TCACCGGCAGTCTCCTTAGAGTGCCCACCATAACGTGCTGGTAACTAAGG

ACAAGGGTTGCGCTCGTTACGGGACTTAACCCAACATCTCACGACACGAG

CTGACGACAGCCATGCAGCACCTGTGTCCTAGTTCCCGAAGGCACCAATC

TATCTCTAGAAAGTTCTCTACATGTCAAGGCCTGGTAAGGTTCTTCGCGT

TGCTTCGAATTAAACCACATGCTCCACCGCTTGTGCGGGCCCCCGTCAAT

TCATTTGAGTTTTAACCTTGCGGCCGTACTCCCCAGGCGGTCAATTTAAT

GCGTTAGCTGCGGAACTAACATCTCAAGGATTCAAACGGCTAATCGACAT

CGTTTACGGCGTGGACTACCAGGGTATCTAATCCTGTTTGCTCCCCACGC

TTTCGCACCTGAGCGTCAGTATCAGTCCAGGGGGTCGCCTTCGCCACTGG

TGTTCCTTCCTATATCTACGCATTTCACCGCTACACCTGAAATTCCACCC

CCCTCTACCAGACTCTAGCTGGACAGTTTTAAATGCAGTTCCCAGGTTGA

GCCCGGGGCTTTCACATCTAACTTAACAAACCACCTACGCGCGCTTTACG

CCCAGTAATTCCGATTAACGCTTGCACCCCTCTGTATTACCGCGGCTGCT

GGCACAGAGTTAGCCGGTGCTTCTTTCTGTCGAGTAACGTCAAAAACTGA

TAACGTATTTAGCTTATCTAGCCTTTCCTCCTCAGCTGAAGGTGCTTACA

ATTCCGAAGACTTCTTCACACACGCCGGCATGCTGCATCAAGCTTCGCCA

ATTGGTCCATATTCCCCCTTGCCTGCCTCCGTTAGAGTCTGGAACGTTCT

CAGTCCAATGGTGGACTGATCATTCCATCCTAGAAACACCAACTCTACCG

AATTCGGTC

>SYC48

CGCGTGGCAACCTTGGTTCGACTTCACCCAGTCATGAATCACACCGTGGT

AACCGTCCTCCCGAAGGTTAGACTAGAACATCTGGTGCAACCCACTCCCA

TGGTGTGACGGGCGGTGTGTACAAGGCCCGGGAACGTATTCACCGCGACA

TTCTGATTCGCGATTACTAGCGATTCCGACTTCACGCAGTCGAGTTGCAG

ACTGCGATCCGGACTACGATCGGTTTTGTGAGATTAGCTCCACCTCGCGG

CTTGGCAACCCTCTGTACCGACCATTGTAGCACGTGTGTAGCCCAGGCCG

TAAGGGCCATGATGACTTGACGTCATCCCCACCTTCCTCCGGTTTGTCAC

CGGCAGTCTCCTTAGAGTGCCCACCATTACGTGCTGGTAACTAAGGACAA

GGGTTGCGCTCGTTACGGGACTTAACCCAACATCTCACGACACGAGCTGA

CGACAGCCATGCAGCACCTGTGTCAAAGTTCCCGAAGGCACCAATCCATC

TCTGGAAAGTTCATTGGATGTCAAGGCCTGGTAAGGTTCTTCGCGTTGCT

TCGAATTAAACCACATGCTCCACCGCTTGTGCGGGCCCCCGTCAATTCAT

TTGAGTTTTAACCTTGCGGCCGTACTCCCCAGGCGGTCAACTTAATGCGT

TAGCTGCGCCACTAAAATCTCAAGGATTCCAACGGCTAGTTGACATCGTT

TACGGCGTGGACTACCAGGGTATCTAATCCTGTTTGCTCCCCACGCTTTC

GCACCTCAGTGTCAGTATGAGCCCAGGGTGGTCGCCTTCGCCACTGGTGT

TCCTTCCTATATCTACGCATTTCACCGCTACACAGGAAATTCCACCACCC

TCTACCCTACTCTAGCTCGCCAGTTTTGGATGCAGTTCCCAGGTTGAGCC

CGGGGATTTCACATCCAACTTAACGAACCACCTACGCGCGCTTTACGCCC

AGTAATTCCGATTAACGCCTTGCACCCTCTGTAATAACCCGCGCTGCTGG

CCAGAGTTAGCCCGGTGCTTTATTCTGTCCGGTACGTCAAACACAGCAAA

GGTATTAGCCTTACTGGCCCTTCCTCCCAAGCTTAAAGTGCTTTAACAAT

CCGAAGACTTCTTTCCACAACACGCGAATGGCTTGGATCCAGGCTTCGCC

AATTGGTCAATATCCCCTACTGCTGCCTTCTCGGTAGGAGTCCTGGA

>SYC49

AGGGGCCGTGGTTGCCTTGTTTCGACTTCACCCCAGTCATGATCACAAAG

TGGTAAGCGCCCTCCCGAAGGTTAAGCTAGTACATCGTTTGCAACCCACT

CCCATGGTGTGACGGGCGGTGTGTACAAGGCCCGGGAACGTATTCACCGT

GGCATTCTGATCCACGATTACTAGCGATTCCGACTTCACGGAGTCGAGTT

GCAGACTCCGATCCGGACTACGACATACTTTATGAGGTCCGCTTGCTCTC

GCGAGGTCGCTTCTCTTTGTATATGCCATTGTAGCACGTGTGTAGCCCTA

CTCGTAAGGGCCATGATGACTTGACGTCATCCCCACCTTCCTCCAGTTTA

TCACTGGCAGTCTCCTTTGAGTTCCCGGCCGAATCGCTGGCAACAAAGGA

TAAGGGTTGCGCTCGTTGCGGGACTTAACCCAACATTTCACAACACGATC

TGACGACAGCCATGCAGCACCTGTCTCAGAGTTCCCGAAGGCACCAAAGC

ATCTCTGCTAAGTTCTCTGGATGTCAAGAGTAGGTAAGGTTCTTCGCGTT

GCATCGAATTAAACCACATGCTCCACCGCTTGTGCGGGCCCCCGTCAATT

CATTTGAGTTTTAACCTTGCGGCCGTACTCCCCAGGCGGTCGACTTAACG

CGTTAGCTCCGGAAGCCACTCCTCAAGGGAACAACCTCCAAGTCGACATC

GTTTACAGCGTGGACTACCAGGGTATCTAATCCTGTTTGCTCCCCACGCT

TTCGCACCTGAGCGTCAGTCTTTGTCCAGGGGGCCGCCTTCGCCACCGGT

ATTCCTCCAGATCTCTACGCATTTCACCGCTACACCTGGAATTCTACCCC

CCTCTACAGACTCTAGCCTGCCAGTTTCAATGCAGTTCCCAAGGTGGAGC

CCGGGGGATTTCACATCCGACTTGACAGACCGCCTGCGTGCCGCTTTACG

CCCAGTAATTTCCGATTAACGCTTGCACCCTCCGTATTACCGCGGCTGCT

GGCACGGAGTTAGCCGGGGCTTCTTCTGCGATTACGTCAATGGATGGCGG

TATTAACCCACATCCCTTTCCTCCCTCGCCTGAAGGTACTTACAACCTGA

AGCCTTCTTTCATAACACGCGGCATGGCTTGCAATCAGCTGGCGCCAATG

GGGTAAAATTCCCCACTGGCTGGCCTCCGTAAGGAGTCTGACCTGTTCTC

AGTTCGATTGTGGCTTGGTCAATCTCACTTCAGAACAGAGCCAGAGGAGA

ATCTGC

>SYC50

CGGGGGCCCGTTGTTACGACTTCACCCCAGTCACGAACCCCGCCGTGGTA

AGCGCCCTCCTTGCGGTTAGGCTACCTACTTCTGGCGAGACCCGCTCCCA

TGGTGTGACGGGCGGTGTGTACAAGACCCGGGAACGTATTCACCGTGACA

TTCTGATCCACGATTACTAGCGATTCCGACTTCACGCAGTCGAGTTGCAG

ACTGCGATCCGGACTACGACTGGCTTTATGGGATTAGCTCCCCCTCGCGG

GTTGGCAACCCTTTGTACCAGCCATTGTATGACGTGTGTAGCCCCACCTA

TAAGGGCCATGAGGACTTGACGTCATCCCCACCTTCCTCCGGTTTGTCAC

CGGCAGTCCCATTAGAGTGCTCAACTGAATGTAGCAACTAATGGCAAGGG

TTGCGCTCGTTGCGGGACTTAACCCAACATCTCACGACACGAGCTGACGA

CAGCCATGCAGCACCTGTGTGCAGGTTCTCTTTCAAGCACCAAACCATCT

CTGGTAAGTTCCTGCCATGTCAAAGGTGGGTAAGGGTTTTCGCGTTGCAT

CGAATTAAACCACATCATCCACCGCTTGTGCGGGTCCCCGTCAATTCCTT

TGAGTTTCAACCTTGCGGCCGTACTCCCCAGGCGGTCAACTTCACGCGTT

AGCTTCGTTACTGAGTCAGTTAAGACCCAACAACCAGTTGACATCGTTTA

GGGCGTGGGACTACCAGGGTATCTAATCCTGTGTTGCTCTCCCACGCTTT

CGTGCATGAGCGTCAGTGCAGGCCCAGGGGATTGCCTTCGCCATCGGTGT

TTCCTCCGCATATCTACGCATTTCACTGCTACACGCGGAATTCCATCCCC

CCTCTGCCGCACTCTAGCTTTGCAGTCACAATGGTCAGTTCCCAGGGTGA

GCCCGGGGATTTCACCACTGTCTTACAACAACCGCCTGCGCACGCTTTAC

GCCCAGTAATTTCCGAATAAACGCCTTGCACCCTACGTATTACCGCGGCC

TGCTGGCAACGTAGTTAGCCGGTGCTTATTTCTTACGATAACAGTCATGA

CCTGAGGGATAATTAGCCCAAGACTTTTCGTTCGTACTAAAGCAGCTTAC

AACCGGAGAGTCTTCATCTGGCACGCGCATGCTGATCAGGCTTCGCCCAT

TGTCGAATCCCACGTGCTGCTTCCGTAGAGTCTGACCGGTTTCAGACAGT

GGCTGATCGTCTCTTCGACAGTCTACGATCGCAAGCCTTGTA

>SYC51

GCCGGTGGCGGCCGGGGTTACGACTTTCCCCCAGTCATGAATCACACCGT

GGTAACCGCCCTCCCGAAGGTTAAACTAGAACATCTGGTGCAACCCACTC

CCATGGTGTGACGGGCGGTGTGTACAAGGCCCGGGAACGTATTCACCGCG

ACATTCTGATTTGCGATTACTAGCGATTCCGACTTCACGGAGTCGAGTTG

CAGACTGCGATCCGGACTACGATCGGCTTTGTGAGATTAGCTCCACCTCG

CGGCTTGGCAACCCTCTGTACCCACCATTGTAGCACGTGTGTAGCCCTGG

CCGTAAGGGCCATGATGACTTGACGTCATCCCCACCTTCCTCCGGTTTGT

CACCGGCAGTCTCCTTTGAGTGCCCACCATAACGTGCTGGTAACTAAGGA

CAAGGGTTGCGCTCGTTGCGGGACTTAACCCAACATCTCACGACACGAGC

TGACGACAGCCATGCAGCACCTGTGTTCTAATTCCCGAAGGCACCAATCC

ATCTCTGGAAAGTTCTCTACATGTCAAGGCCTGGTAAGGTTCTTCGCGTT

GCTTCGAATTAAACCACATGCTCCACCGCTTGTGCGGGCCCCCGTCAATT

CATTTGAGTTTTAACCTTGCGGCCGTACTCCCCAGGCGGTCAATTTAACG

CGTTAGCTGCGGAACTCACATCTCAAGGACACAAACGGCTAATTGACATC

GTTTACGGCGTGGACTACCAGGGTATCTAATCCTGTTTGCTCCCCACGCT

TTCGCACCTGAGCGTCAGTATCAGTCCAGGGGGTCGCCTTCGCCACTGGT

GTTCCTTCATATCTCTACGCATTTCACCGCTACACATGAAATTCTACCCC

CCTCTACCGGACTCTAGCTTGACAGTTTTAGATGCAGTTCCCAGGTTGAG

CCCGGGGCTTTCACATCTAACTTATCAAACCACCTACGCGCGCTTTACGC

CCAGTATTTCCGATTAACGCTTGCACCCTCTGTATTTACCGCGGGCTGCT

GGCACAGAAGTTAGCCCGGTGGCTTATTTCTGGCGAGTAACGTTCACAGC

TGCAAAGGGTATTAAGGTTATCAGCCTTTTCTCCCTACCTTGAAGGTGGC

TTTTACAACCCGGAAGGCCCTTCTTCCCCACACGCGGGCATGGCTGGCAT

TCAAGGGCTTTCCCCAATTGTGCCAATAATTCCCCACTTGCTGCCTTCCG

GTAGGAAGTCTGGAACGGTTCCAAGTCCAAGTTGTGACTTGATCATTCCT

CTCTAGGACCGAGCAACGGGATAGATGTCTGCGTCG

>SYC52

CAATGGCTCCGTTGCTACGACTTCACCCCAGTCACGAACCCCGCCGTGGT

AAGCGCCCTCCTTGCGGTTAGGCTACCTACTTCTGGCGAGACCCGCTCCC

ATGGTGTGACGGGCGGTGTGTACAAGACCCGGGAACGTATTCACCGTGAC

ATTCTGATCCACGATTACTAGCGATTCCGACTTCACGCAGTCGAGTTGCA

GACTGCGATCCGGACTACGACTGGCTTTATGGGATTAGCTCCCCCTCGCG

GGTTGGCAACCCTTTGTACCAGCCATTGTATGACGTGTGTAGCCCCACCT

ATAAGGGCCATGAGGACTTGACGTCATCCCCACCTTCCTCCGGTTTGTCA

CCGGCAGTCCCATTAGAGTGCTCAACTGAATGTAGCAACTAATGGCAAGG

GTTGCGCTCGTTGCGGGACTTAACCCAACATCTCACGACACGAGCTGACG

ACAGCCATGCAGCACCTGTGTGCAGGTTCTCTTTCGAGCACCAAACCATC

TCTGGTAAGTTCCTGCCATGTCAAAGGTGGGTAAGGTTTTTCGCGTTGCA

TCGAATTAAACCACATCATCCACCGCTTGTGCGGGTCCCCGTCAATTCCT

TTGAGTTTCAACCTTGCGGCCGTACTCCCCAGGCGGTCAACTTCACGCGT

TAGCTTCGTTACTGAGTCAGTTAAGACCCAACAACCAGTTGACATCGTTT

AGGGCGTGGACTACCAGGGTATCTAATCCTGTTTGCTCCCCACGCTTTCG

TGCATGAGCGTCAGTGCAGGCCCAGGGGATTGCCTTCGCCATCGGTGTTC

CTCCGCATATCTACGCATTTCACTGCTACACGCGGAATTCCATCCCCCTC

TGCCGCACTCTAGCTTTGCAGTCACAATGGCAGTTCCCAGGTTGAGCCCG

GGGATTTCACCACTGTCTTACAAAACGGCCTGCGCACGCTTTACGCCCAG

TAATTTCCGATTAACGCTTGCACCCTACGTATTACCGCGGCTGCTGGGCA

CGTAGTTAGCCGGTGCTTATTCTTACGTACAGTCATGACCTGAGGGTATT

AGCCCAGCCTTTCGTCGTACTAAAGCAGTTTACACCGAGGGCTCATCTGC

ACGCGCCATGCTGATCAGCCTTCGCCATTGTCAAATCCCACTGCTGCTCC

GTAGATCTGTACGGTTTCAGTTCCAGTGACTGATCGTCCTCTTCGAACAG

CTACGAAATCGCAAGG

>SYC53

GCGTTTCTGCTGTTTCCCGTGCGTGTCGAGTGGTAGCCTTGGTGAGCTTT

ACCTCAGTGCCGGCGGGGGAACAAGGACCGTAAAGTCCGGCAAAGTGCGG

GGTACAGCGGCATAAAAGTGGCGAACGAATGCTAAAACCGCAGACGGACT

GCGCGGGAAACCCGGGCCCCTTGCGACTTTACCGGATGTTAGTCGCCCAG

GTGGTATTCGCGGGAAAGGGTAGCAAGGGCTCCGCTCGGCGACGGTCCTG

AGAGGACCTGAAAGGATACTGGGACTGTGACATATTAACCACTAAAACAC

TTTAAAGCGGTGGGGAATTTTGGACAATGGGGGAAAGCCTGATCCAGCAA

TGCCCCGTGCAGGATGAAGGCCCTCGGGTTGTAAACTGCTTTTGTACGGA

ACGAAAAGCCTGGGGCTATTATCCCCGGGTCATGACGGTATCGTAAGAAT

AAGCACCGGGGGGGCTACGTGCCAGCACCCGCGGTAATACGTAGGGTGCA

AGCGTTAATCGGAATTACTCGGCGTTAAGCGTGCGCAAGCGGTTTTGTAA

GACAGTGGTGAAATCACCGGGCTCAACCTGGGAACTGCCATTGTGACTGC

AAAGCTAGAGTGCGGCAGAGGCGGGATGGAATTCCGCGTGTTCAGTGAAA

TGCGTAGATATGCGGAGGAACACCGATGGCGAAGGCAATCCCCTGGGCCT

GCACTGACGCTCATGCACGAAACGTGGGGAGCAACAGGATTATATACCCT

GGTAGTCCACGCCCTAAACGATGTCAACTGATTGTTGGTTCTTAACTGAC

TCAGTGACAAGCTAACGCGTGAAGTTGACCGCCTGGCGAGTACGGGCGCA

AGGCTGAAACTCGAGGAATTTGACGGGGACTCGCACGAGCCGGTGGATGA

TGTGGGTTAATTCTATGTACGCCAATAAACTTACCCACCTTTGACTTGGC

CGGAACTTACCTGAAATGGTTTGATGCTCGAAGAAGAACCTTGCCCACAG

GGGCCGCCATGGGCGGTCATCGACCTCGTGTCAGCGAGAGGTTTGGGTAA

GTTCCCCCAACGACCGCCACCCATGGCTATTCTACTGGCTTTCATTGTAG

CAGACCACTCTTATGGGGAACTTCAGTGAACAAATCGGGAGGAAGTGCCG

GATAACGCTAGGTCTCTCATGGACCTTATCAGTGGGCGCTTACCCCTTTA

TTACATGGCTGGGTCCAT

>SYC54

CTTGGCGGCTGGCTTTTAACATGCAGTCGAACGGTAACAGGTCTTCGGAT

GCTGACGAGTGGCGAACGGGTGAGTAATACATCGGAACGTGCCTAGTATT

GGGGGATAACTACTCGAAAGAGTAGCTAATACCGCATGAGATCTACGGAT

GAAAGCAGGGGACCTTCGGGCCTTGTGCTACTATAGCGGCTGATGGCAGA

TTAGGTAGTTGGTGGGGTAAAGGCTTACCAAGCCTGCGATCTGTAACTGG

TCTGAGAGGACGACCAGCCACACTGGGACTGAGACACGGCCCAAACTCCT

ACGGGAGGCAGCAGTGGGGAATTTTGGACAATGGGCGAAAGCCTGATCCA

GCAATGCCGCGTGCAGGATGAAGGCCCTCGGGTTGTAAACTGCTTTTGTA

CGGAACGAAAAGCCTGGGGCTAATATCCCCGGGTCATGACGGTACCGTAA

GAATAAGCACCGGCTAGCTACGTGCCAGCAGCCGCGGTAATACGTAGGGT

GCAAGCGTTAATCGGAATTACTGGGCGTAAAGCGTGCGCAGGCGGTTTTG

TAAGACAGTGGTGAAATCCCCGGGCTCAACCTGGGAACTGCCATTGTGAC

TGCAAAGCTAGAGTGCGGCAGAGGGGGATGGAATTCCGCGTGTAGCAGTG

AAATGCGTAGATATGCGGAGGAACACCGATGGCGAAGGCAATCCCCTGGG

CCTGCACTGACGCTCATGCACGAAAGCGTGCGGAGCAAACAGGATTAGAT

ACCCTGGTAGTCCACGCCCTACACGATGTCAACTGGTTGTTGGGTCTTAA

CTGACTCAGTAACGAAGCTAACGCGTGAAGTTGACCGCCTGGCGAGTACG

GCCGCAAGGTTGAAACTCAAAGGAATTGACGGGACTCGCACAAGCCGCGG

ATGATGTGGTTAAATTCGATGCAACTCGAAAACCCTTACCCACCTCTTGA

CATGGCCTGGACTTACCAGAGATGCTTTGTGCTCGAAGAAGACCCTGCAC

ACATGTGACTGCATGGCTGTCGTCAGCTCGTGTCGTGAGATGTTGGATAA

GTCCCGCACGAGCCGCACCCATTGCATACTTGCTACATGTCAGCTGAGCA

CTCCTATGGACTGCAGTGACAATCGCGGAGGAAGGTGCGATGAACGTCAG

TCTCATGCCCTATACGTGGGCTACAGCCGTGATACATGCTGTACAAGTGC

ACCCGCGTGGGAACTATTCCCATAAGGCACACTCCTCA

>SYC55

CTTGGCCGGCATGCCTTTCACATGCAGTCGAACGGTAACAGGTCTTCGGA

TGCTGACGAGTGGCGAACGGGTGAGTAACACATCGGAACGTGCCTAGTAG

TGGGGGATAACTACTCGAAAGAGTAGCTAATACCGCATGAGATCTACGGA

TGAAAGCAGGGGACCTTCGGGCCTTGTGCTACTAGAGCGGCTGATGGCAG

ATTAGGTAGTTGGTGGGGTAAAGGCTTACCAAGCCTGCGATCTGTAGCTG

GTCTGAGAGGACGACCAGCCACACTGGGACTGAGACACGGCCCAGACTCC

TACGGGAGGCAGCAGTGGGGAATTTTGGACAATGGGCGAAAGCCTGATCC

AGCAATGCCGCGTGCAGGATGAAGGCCCTCGGGTTGTAAACTGCTTTTGT

ACGGAACGAAAAGCCTGGGGCTAATACCCCCGGGTCATGACGGTACCGTA

AGAATAAGCACCGGCTAACTACGTGCCAGCAGCCGCGGTAATACGTAGGG

TGCAAGCGTTAATCGGAATTACTGGGCGTAAAGCGTGCGCAGGCGGTTTT

GTAAGACAGTGGTGAAATCCCCGGGCTCAACCTGGGAACTGCCATTGTGA

CTGCAAAGCTAGAGTGCGGCAGAGGGGGATGGAATTCCGCGTGTAGCAGT

GAAATGCGTAGATATGCGGAGGAACACCGATGGCGAAGGCAATCCCCTGG

GCCTGCACTGACGCTCATGCACGAAAGCGTGGGGGAGCAAACAGGATTAG

ATACCCTGGTAGTCCACGCCCTAAACGATGTCAACTGGTTGTTGGGTCTT

AACTGACTCAGTAACGAAGCTAACGCGTGAAGTTGACCGCCTGGGGAGTA

CGGCCGCAAGGTTGAAACTCAAAGGAATTGACGGGGACTCGCACAAGCGG

TGGATGATGTGGGTTTAATTCGATGCAACGCGAAAACCCTTACTCACCTT

TGACATGGCAGAACTTACCAGAGATGGTTTGGTGCTCGAAAGAGGACCTG

CACACAGTGCTGCATGCTGTCGTCAGCTCCGTGTCGTGAGATGTGGTAAG

TCCCGCACGAGCGCACCATGCATACTGCTACTTTCAGTGACACTCTATGG

GACTGCGGTGACAACCGGGAGAAGGTGGGATGACGTCAAGTCTCATGCCT

TAACGTGGGCTACAACGGTCTAACATGCTGTCAAGGTTGCCAACTCTCGG

AATGGGGACTATGTCCATGTAAAGGCAAGCTCCGTA

>SYC56

CGTTGCCGCCTGCTTTAACATGCAGTCGAACGGTAACAGGTCTTCGGATG

CTGACGAGTGGCGAACGGGTGAGTAATACATCGGAACGTGCCTAGTAGTG

GGGGATAACTACTCGAAAGAGTAGCTAATACCGCATGAGATCTACGGATG

AAAGCAGGGGACCTTCGGGCCTTGTGCTACTAGAGCGGCTGATGGCAGAT

TAGGTAGTTGGTGGGGTAAAGGCTTACCAAGCCTGCGATCTGTAGCTGGT

CTGAGAGGACGACCAGCCACACTGGGACTGAGACACGGCCCAGACTCCTA

CGGGAGGCAGCAGTGGGGAATTTTGGACAATGGGCGAAAGCCTGATCCAG

CAATGCCGCGTGCAGGATGAAGGCCCTCGGGTTGTAAACTGCTTTTGTAC

GGAACGAAAAGCCTGGGGCTAATACCCCCGGGTCATGACGGTACCGTAAG

AATAAGCACCGGCTAACTACGTGCCAGCAGCCGCGGTAATACGTAGGGTG

CAAGCGTTAATCGGAATTACTGGGCGTAAAGCGTGCGCAGGCGGTTTTGT

AAGACAGTGGTGAAATCCCCGGGCTCAACCTGGGAACTGCCATTGTGACT

GCAAAGCTAGAGTGCGGCAGAGGGGGATGGAATTCCGCGTGTAGCAGTGA

AATGCGTAGATATGCGGAGGAACACCGATGGCGAAGGCAATCCCCTGGGC

CTGCACTGACGCTCATGCACGAAAGCGTGGGGGAGCAAACAGGATTAGAT

ACCCTGGTAGTCCACGCCCTAAACGATGTCAACTGGTTGTTGGGTCTTAA

CTGACTCAGTAACGAAGCTAACGCGTGAAGTTGACCGCCTGGGGAGTACG

GCCGCAAGGTTGAAACTCAAAGGAATTGACGGGGACCCGCACAAGCGGTG

GATGATGTGGTTTAATTCGATGCAACGCGAAAACCTTTACCCAACCTTTG

ACATGGCAGGAACTTACCAGAGATGGTTTGGTGCTCGAAAGAGAACCTGC

ACACAGTGCTGCATGCTGTCGTCAGCTCGTGTCGTGAGATGTGGTTAAGT

CCCGCACGAGCGCATCATGCATAGTGCTACATTCAGTGACACTCTATGGA

CTGCGTGACATCGGGAGGAGTGGGATGACGTCAGTCTCATGTCTTACGGT

GGCTACACGTCATACATGCTGTCAGCTGCAACAGCTAGGGGACCTATGTC

CGTAAGGGACGAG

>SYC57

CCTTTGGGGTGCTCTCACGTGCGCTCGGATCGTAGGCTGGGTCGGATGCA

GACGAGTGGTGCACGGGCGAGTAACACATCGGAACGTGCCTAGAGGTGGG

GAGTAACTACGCGAAAGAGTGTAGATTACAGCATGAGATCTACGGATGAA

AGCAGGGGACCTTCAGGCCTTGAGCTACGTTAGCGGGTGATGCCCGATTG

GGCGCCTGGCGGGGGAAAGGCTTACCAAGCCTGCGATCTGTAGCTGGGCT

GAGAGGATGACCAGCCCCACTGGTCCTGACCCTGGTCCAACTCTTCAACG

GGAGGCAGCAGTGGGGAATTTTGGACAATGGGCGAAAGCCTGATCCAGCA

ATGCCCCGAACAGGATGAAGGCCCTCGGGTTGTAAACTGCTTTTGTACTG

AACGAAAAGCCTGGGGCTAATATCCCCGGGTCATGACGGTACCGTAAGAA

TAAGCACCGGCTAACTACGTGCCAGCAGCCGCGGTAATACGTAGGGTGCA

AGCGTTAATCGGAATTACTGGGCGTAAAGCGTGCGCAGGCGGTTTTGTAA

GACAGTGGTGAAATCCCCGGGCTCAACCTGGGAACTGCCATTGTGACTGC

AAAGCTAGAGTGCGGCAGAGGGGGGATGGAATTCCGCGTGTAGCAGTGAA

ATGCGTAGATATGCGGAGGAACACCGATGGCGAAGGCAATCCCCCTGGGC

CTGCACTGACGCTCATGCACGAAAGCGTGAGGAGCAAACAGGATTAGATA

CCCTGGTAGTCCACGCCCTAAACGATGTCAACTGGTTGTTGGGTCTTAAC

TGACTCAGTAACGAAGCTAACGCGTGAAGCTGACCGCCTGCGGAGTACGG

CCGCAAGGTTGAAACTCAAAGGAATTGACGGGGACCCGCGCACGCGCGGA

TGATGTGGTTTAAGTTCGATTGCATCGCGAAAAACCTTACCCTCTTTTGA

TATGGCAGATACCTTACCAAAGATGGATAGGTGCTCGAGAGAGAACCTGC

CACCCAGAAGCTGCGATGGCTGTCGTCTGCTCCGCGTCCTGGAGATGATG

AGGTTAGGTCCACGACAACGAGCCGCAAACCCCTTGCCCTTTACTTTGCT

TACGTTTCCAGTTGATCACCTCCTGAATGCGCACCTAGCCCGGATGACAA

CCGGAAAGACAGGGTGGGGGAAGTGCACGTCCAGATCCTCCTCATGGCCT

TTGTAGTGAGGGAGCGTGGCGACCTAACACGCGCGTTCAATACAGATTGT

CCTAGGGAACGAGAGTGATATTGCACAGCAGCATCGA

>SYC58

GCTTTGGGGGTGCTTTTAACATGCAGTCGACGGTAACAGGTCTTCGGATG

CTGACGAGTGGCGAACGGGTGAGTAAACATCGGAACGTGCCTAGTAGTGG

GGGATAACTACTCGAAAGAGTAGCTAATACCGCATGAGATCTACGGATGA

AAGCAGGGGACCTTCGGGCCTTGTGCTACTAGAGCGGCTGATGGCAGATT

AGGTAGTTGGTGGGGTAAAGGCTTACCAAGCCTGCGATCTGTAGCTGGTC

TGAGAGGACGACCAGCCACACTGGGACTGAGACACGGCCCAGACTCCTAC

GGGAGGCAGCAGTGGGGAATTTTGGACAATGGGCGAAAGCCTGATCCAGC

AATGCCGCGTGCAGGATGAAGGCCCTCGGGTTGTAAACTGCTTTTGTACG

GAACGAAAAGCCTGGGGCTAATATCCCCGGGTCATGACGGTATCGTAAGA

ATAAGCACCGGCTAACTACGTGCCAGCAGCCGCGGTAATACGTAGGGTGC

AAGCGTTAATCGGAATTACTGGGCGTAAAGCGTGCGCAGGCGGTTTTGTA

AGACAGTGGTGAAATCCCCGGGCTCAACCTGGGAACTGCCATTGTGACTG

CAAAGCTAGAGTGCGGCAGAGGGGGATGGAATTCCGCGTGTAGCAGTGAA

ATGCGTAGATATGCGGAGGAACACCGATGGCGAAGGCAATCCCCTGGGCC

TGCACTGACGCTCATGCACGAAAGCGTGGGGAGCAAACAGGATTAGATAC

CCTGGTAGTCCACGCCCTAAACGATGTCAACTGGTTGTTGGGTCTTAACT

GACTCAGTAACGAAGCTAACGCGTGAAGTTGACCGCCTGGGGAGTACGGC

CGCAAGGTTGAAACTCAAAGGAATTGACGGGGACCCGCACAAGCGGTGGA

TGATGTGGTTTAATTCGATGCAACGCGAAAAACCTTACCCACCTTTGACA

TGGCAGGAACTTACCAGAGATGGTTTGGTGCTCGAAAGAGAACCTGCACA

CAGGTGCTGCATGGCTGTCGTCAGCTCGTGTCGTGAGAATGTTGGGTTAA

GTCCCGCAACGAGCGCAACCCTTGGCCATTAGTTGCTACATTCAGTTGAG

CACTCTAATGGGACCTGCCGGTGACAACCGGGAGGAGGTGGGGATGACGT

CAGGTCCCTCATGACCTTAATAGTGGGGCTTAACACCACGTCGATCATGC

TGACAAGTTGCTACCCGCGCAGGGGAAGCCTTAATCTCCCCATAAAA

>SYC59

CTTTTGGGGAGGCTTTTCCATGCAAGTCGAACGGTTACAGGTCTTCGGAT

GCTGACGAGTGGCGAACGGGTGAGAAAAACATCGGAACGTGCCTAGGAGT

GGGGGATAACTACTCGCAAGAGTAGCTAATACCGCATGAGATCTACGGAT

GAAAGCAGGGGACCTTCGGGCCTTGTGCTACTAGAGCGGCTGATGGCAGA

TTAGGTAGTTGGTGGGGTAAAGGCTTACCAAGCCTGCGATCTGTAGCTGG

TCTGAGAGGACGACCAGCCACACTGGGACTGAGACACGGCCCAGACTCCT

ACGGGAGGCAGCAGTGGGGAATTTTGGACAATGGGCGAAAGCCTGATCCA

GCAATGCCGCGTGCAGGATGAAGGCCCTCGGGTTGTAAACTGCTTTTGTA

CGGAACGAAAAGCCTGGGGCTAATATCCCCGGGTCATGACGGTATTTTAA

GAATAAGCACCGGCTAACTACGTGCCAGCAGCCGCGGTAATACGTAGGGT

GCAAGCGTTAATCGGAATTACTGGGCGTAAAGCGTGCGCAGGCGGTTTTG

TAAGACAGTGGTGAAATCCCCGGGCTCAACCTGGGAACTGCCATTGTGAC

TGCAAAGCTAGAGTGCGGCAGAGGGGGATGGAATTCCGCGTGTAGCAGTG

AAATGCGTAGATATGCGGAGGAACACCGATGGCGAAGGCAATCCCCTGGG

CCTGCACTGACGCTCATGCACGAAAGCGTGGGGAGCAAACAGGATTAGAT

ACCCTGGTAGTCCACGCCCTAAACGATGTCAACTGGTTGTTGGGTCTTAA

CTGACTCAGTAACGAAGCTAACGCGTGAAGTTGACCGCCTGGGGAGTACG

GCCGCAAGGTTGAAACTCAAAGGAATTGACGGGACCCGCACAAGCGGTGG

ATGATGTGGTTTAATTCGATGCAACGCGAAAAACCTTACCCACCTTTGAC

ATGGCAGAACTTACCAGAGATGGTTTGGTGCTCGAAAGAGAACCTGCACA

CCAGTGCTGCATGGCTGTTCGTTCAGCTCGTGTCGTGAGATGTTGGTTAA

GTTCCCGCAAACAGCCGCAAACCCTTGCCATTAGTTGCTAACATTCAGTT

TGAGCACGTCTATGACTGCCCGTTGACCAAACCGGAGAGGTAGGGATGAC

GTCAGTCCTCATGCTTATAGTTGGGCCTACACACGTCATCATGTCTGTCA

GAGGGTTGCATCCCGCCAAGGGACTTATTCCCTATAG

>SYC60

TGGGAGGGTCCTGGGGTTCACTTCCCCCCATGTCATGATCACACCGTGGT

AAGCGCCCTCCTTAAGGTTAAGGTAAAAAGGGGGGGTGCAGACCAGTTCC

ATGGGGGGACGGGGGGCGCGTAAAAAAGACCCGGGAACGTATTCACCGTG

GCATTCTGATCCACGATTACTAGCGATTCCGACTTCACGGAGTCGAGTTG

CAGACTGCGATCCGGACTACGACTGGGTTTTTGGGATTGGGTCCCCCTCG

CGGGTTGGCAACCCTCTGTACCGACCATTGTATGACGTGTGTAGCCCCCC

CCATAAAGGGCCATGAGGACTTGACGTCATCCCCCACCTTCCTCCGGGTT

TTTCCCCCGGGCAGTCTCCTTAGAGTGCCCCGCCGAACGGAGGGAAAAAA

AGGGAAAGGGGGTGCGCCCGGTGCGGGGACTTTTTTCCTTCCTTTCCCAA

CATGTTTTTTCCACACACCTGCTGGGGGTGTGTGTTATTTCCCTAAAGCA

CCAAACCATCTCTGCTAAGTTCTCTGGATGTCGAAGGGAGGGGAGGTTCT

TCTCGTTGCATCTAATTAAAACACATCATCCACTGCTTGTGGGGGCCCCC

CCCCAATTCTTTTTTAGTTTTAACCTTGCGGGGCGTACTCCCCAGGCGGG

>SYC61

CCGGGCGGCGGCCCTACACATGCAAGTCGAGCGGCAGCGGGAAAGTAGCT

TGCTACTTTTGCCGGCGAGCGGCGGACGGGTGAGTAATGCCTGGGAAATT

GCCCAGTCGAGGGGGATAACAGTTGGAAACGACTGCTAATACCGCATACG

CCCTACGGGGGAAAGCAGGGGACCTTCGGGCCTTGCGCGATTGGATATGC

CCAGGTGGGATTAGCTTGTTGGTGAGGTAATGGCTCACCAAGGCGACGAT

CCCTAGCTGGTCTGAGAGGATGATCAGCCACACTGGAACTGAGACACGGT

CCAGACTCCTACGGGAGGCAGCAGTGGGGAATATTGCACAATGGGGGAAA

CCCTGATGCAGCCATGCCGCGTGTGTGAAGAAGGCCTTCGGGTTGTAAAG

CACTTTCAGCGAGGAGGAAAGGTTGATGCCTAATACGTATCAGCTGTGAC

GTTACTCGCAGAAGAAGCACCGGCTAACTCCGTGCCAGCAGCCGCGGTAA

TACGGAGGGTGCAAGCGTTAATCGGAATTACTGGGCGTAAAGCGCACGCA

GGCGGTTGGATAAGTTAGATGTGAAAGCCCCGGGCTCAACCTGGGAATTG

CATTTAAAACTGTCCAGCTAGAGTCTTGTAGAGGGGGGTAGAATTCCAGG

TGTAGCGGTGAAATGCGTAGAGATCTGGAGGAATACCGGTGGCGAAGGCG

GCCCCCTGGACAAAGACTGACGCTCAGGTGCGAAAGCGTGGGGAGCAAAC

AGGATTAGATACCCTGGTAGTCCACGCCGTAAACGATGTCGATTTGGAGG

CTGTGTCCTTGAGACGTGGCTTCCGGAGCTAACGCGTTAAATCGACCGCC

TGGGGAGTACGGCCGCAAGGTTAAAACTCAAATGAATTGACGGGGGCCCG

CACAAGCGGTGGAGCATGTGGTTTAATTCGATGCAACGCGAAGAACCTTA

CCTGGCCTTGACATGTCTGGAATCCTGTAGAGATGCGGGAGTGCCTTCGG

GAATCAGACACAGTGCTGCATGCTGTCGTCAGCTCGTGTCGTGAGATGTT

GGGTTAGTCCCGCACGAGCGCACCCTTGTCTTTGTTGCAGCACGTATGGT

GGACTCAAGGGAGACTGCCGGTGATAACTGAGAGTGGGATGACGTCAGTC

ATCATGGCTTACGGCAGGCTACACTGCTAATGCGCGTACAAAGGCTCAAG

CTATGCGTATGTGAGCGCAAT

>SYC62

CGTGGCGGCCTGCTTTAACATGCAAGTCGAACGGTAACAGGTCTTCGGAT

GCTGACGAGTGGCGAACGGGTGAGTAATACATCGGAACGTGCCTAGTAGT

GGGGGATAACTACTCGAAAGAGTAGCTAATACCGCATGAGATCTACGGAT

GAAAGCAGGGGACCTTCGGGCCTTGTGCTACTAGAGCGGCTGATGGCAGA

TTAGGTAGTTGGTGGGGTAAAGGCTTACCAAGCCTGCGATCTGTAGCTGG

TCTGAGAGGACGACCAGCCACACTGGGACTGAGACACGGCCCAAACTCCT

ACGGGAGGCAGCAGTGGGGAATTTTGGACAATGGGCGAAAGCCTGATCCA

GCAATGCCGCGTGCAGGATGAAGGCCCTCGGGTTGTAAACTGCTTTTGTA

CGGAACGAAAAGCCTGGGGCTAATACCCCCGGGTCATGACGGTACCGTAA

GAATAAGCACCGGCTAACTACGTGCCAGCAGCCGCGGTAATACGTAGGGT

GCAAGCGTTAATCGGAATTACTGGGCGTAAAGCGTGCGCAGGCGGTTTTG

TAAGACAGTGGTGAAATCCCCGGGCTCAACCTGGGAACTGCCATTGTGAC

TGCAAAGCTAGAGTGCGGCAGAGGGGGATGGAATTCCGCGTGTAGCAGTG

AAATGCGTAGATATGCGGAGGAACACCGATGGCGAAGGCAATCCCCTGGG

CCTGCACTGACGCTCATGCACGCAAAGCGTGGGGAGCAAACAGGATTAGA

TACCCTGGTAGTCCACGCCCTAAACGATGTCAACTGGTTGTTGGGTCTTA

ACTGACTCAGTAACGAAGCTAACGCGTGAAGTTGACCGCCTGGGGAGTAC

GGCCGCAAGGTTGAAACTCAAAGGAATTGACGGGGACTCGCACAAGCGGT

GGATGATGTGGTTTAATTCGATGCAACGCGAAAAACCTTACCCACCTTTG

ACATGGCCAGGAACTTACCAGAGATGGTTTGGTGCTCGAAAGAGACCTGC

ACACAGTGCTGCATGGCTGTCGTCAGCTCGTGTCGTGAGATGTTGGATTA

AGTCCCGCTACGACCGCAACCCTTTGCCATTAGTGGCTACATTCAGCTGG

AGCACTCTAGTGGACTGCAGTGACAAACCGGAGGGAAGTGGGATGGACGT

CCAGGTCCTCATGCCTTATCGTGGGCTTACCACGTCGATACATTGACTGG

TACAAAGGATGCCTAAACTACGCGAGGCGGAACTTATCACAATTAAAGCC

ACATCTCTTTAAAAT

>SYC63

CTTGGCGGATGCTTTAAAATGCAGTCGACGGTAACAGGTCTTCGGATGCT

GACGAGTGGCGAACGGGTGAGTAATACATCGGAACGTGCCTAGTAGTGGG

GGATAACTACTCGAAAGAGTAGCTAATACCGCATGAGATCTACGGATGAA

AGCAGGGGACCTTCGGGCCTTGTGCTACTAGAGCGGCTGATGGCAGATTA

GGTAGTTGGTGGGGTAAAGGCTTACCAAGCCTGCGATCTGTAGCTGGTCT

GAGAGGACGACCAGCCACACTGGGACTGAGACACGGCCCAGACTCCTACG

GGAGGCAGCAGTGGGGAATTTTGGACAATGGGCGAAAGCCTGATCCAGCA

ATGCCGCGTGCAGGATGAAGGCCCTCGGGTTGTAAACTGCTTTTGTACGG

AACGAAAAGCCTGGGGCTAATATCCCCGGGTCATGACGGTACCGTAAGAA

TAAGCACCGGCTAACTACGTGCCAGCAGCCGCGGTAATACGTAGGGTGCA

AGCGTTAATCGGAATTACTGGGCGTAAAGCGTGCGCAGGCGGTTTTGTAA

GACAGTGGTGAAATCCCCGGGCTCAACCTGGGAACTGCCATTGTGACTGC

AAAGCTAGAGTGCGGCAGAGGGGGATGGAATTCCGCGTGTAGCAGTGAAA

TGCGTAGATATGCGGAGGAACACCGATGGCGAAGGCAATCCCCTGGGCCT

GCACTGACGCTCATGCACGAAAGCGTGGGGAGCAAACAGGATTAGATACC

CTGGTAGTCCACGCCCTAAACGATGTCAACTGGTTGTTGGGTCTTAACTG

ACTCAGTAACGAAGCTAACGCGTGAAGTTGACCGCCTGGGGAGTACGGCC

GCAAGGTTGAAACTCAAAGGAATTTGACGGGGACTCGCACAAGCGGTGGA

TGATGTGGTTTAATTTCGATGCTACGCGAAAAACCTTACCCACCTTTTGA

CATGGCAGAACTTACCAGAGATGTTTGGTGCTCGAAAGAAGACTGCACAC

AGTGCTGCATGCTGTCGTCAGCTCGTGTCGTGAGATGTGGGTAAGTCCCG

CACGACGCACCCTGCATAGTGCTACTTCAGTGAGCATCTATGGACTGCGT

GACAACCGAGGAAGTGGGATGACGTCAGTCCTCCATGCCCTTAGTGGGCT

AACAGCTATACATGCTGTAAAGTTGCCACTCGGAGGGACATTATCCTATA

AG

>SYC64

CCTGGGGGGGGCCTTTCCCTTGCAGTCGAACGGTAACAGGTCTTCGGATG

CTGACGAGTGGCGAACGGGTGAGTAAAACATCGGAACGTGCCTAGTAGTG

GGGGATAACTACTCCAAAGAGTAGCTAATACCGCATGAGATCTACGGATG

AAAGCAGGGGACCTTCGGGCCTTGTGCTACTAGAGCGGCTGATGGCAGAT

TAGGTAGTTGGTGGGGTAAAGGCTTACCAAGCCTGCGATCTGTAGCTGGT

CTGAGAGGACGACCAGCCACACTGGGACTGAGACACGGCCCAGACTCCTA

CGGGAGGCAGCAGTGGGGAATTTTGGACAATGGGCGAAAGCCTGATCCAG

CAATGCCGCGTGCAGGATGAAGGCCCTCGGGTTGTAAACTGCTTTTGTAC

GGAACGAAAAGCCTGGGGCTAATATCCCCGGGTCATGACGGTACCTTAAG

AATAAGCACCGGCTAACTACGTGCCAGCAGCCGCGGTAATACGTAGGGTG

CAAGCGTTAATCGGAATTACTGGGCGTAAAGCGTGCGCAGGCGGTTTTGT

AAGACAGTGGTGAAATCCCCGGGCTCAACCTGGGAACTGCCATTGTGACT

GCAAAGCTAGAGTGCGGGCAGAGGGGGGATGGAATTCCGCGTGTAGCAGT

GAAATGCGTAGATATGCGGAGGAACACCGATGGCGAAGGCAATCCCCTGG

GCCTGCACTGACGCTCATGCACGAAAGCGTGGGGAGCAACAGGATTAGAT

ACCCTGGTAGTCTACGCCTAACGATGTCAACTGGTTGTGGGGTCTTAACT

GACTCATAACAAAGCTAACGCGTGAAGTTGACCGCCTGGGAGTACGGCCG

CAAGGTTGAAACTCAAAGAATTGACGGGACCGCACAGCGCGGATGATGTG

GTTTATTCATGCACGCTAATAACCTTACCACCTTTGACATGGCGGAACTA

ACCGAATGGTTGGTGGCTCAAAAAAAACCTGCACACAGAGGCTGCATGGC

TTGTCGTCAGCTTCTGGT

>SYC65

AATGATTGTTCCTTGGTTACGACTTCACCCCAGTCACGAACCCCGCCGTG

GTAAGCGCCCTCCTTGCGGTTAGGCTACCTACTTCTGGCGAGACCCGCTC

CCATGGTGTGACGGGCGGTGTGTACAAGACCCGGGAACGTATTCACCGTG

ACATTCTGATCCACGATTACTAGCGATTCCGACTTCACGCAGTCGAGTTG

CAGACTGCGATCCGGACTACGACTGGCTTTATGGGATTAGCTCCCCCTCG

CGGGTTGGCAACCCTTTGTACCAGCCATTGTATGACGTGTGTAGCCCCAC

CTATAAGGGCCATGAGGACTTGACGTCATCCCCACCTTCCTCCGGTTTGT

CACCGGCAGTCCCATTAGAGTGCTCAACTGAATGTAGCAACTAATGGCAA

GGGTTGCGCTCGTTGCGGGACTTAACCCAACATCTCACGACACGAGCTGA

CGACAGCCATGCAGCACGTGTGTGCAGGTTCTCTTTCGAGCACCAAACCA

TCTCTGGTAAGTTCCTGCCATGTCAAAGGTGGGTAAGGTTTTTCGCGTTG

CATCGAATTAAACCACATCATCCACCGCTTGTGCGGGTCCCCGTCAATTC

CTTTGAGTTTCAACCTTGCGGCCGTACTCCCCAGGCGGTCAACTTCACGC

GTTAGCTTCGTTACTGAGTCAGTTAAGACCCAACAACCAGTTGACATCGT

TTAGGGCGTGGACTACCAGGGTATCTAATCCTGTTTGCTCCCCACGCTTT

CGTGCATGAGCGTCAGTGCAGGCCCAGGGGATTGCCTTTCGCCATCGGTG

TTCCTCCGCATATCTACGCATTTCACTGCTACACGCGGAATTCCATCCCC

CTCTGCCGCACTCTAGCTTTGCAGTCACAATGGCAGTTCCCAGGTTGAGC

CCGGGGATTTCACCACTGTCTTACAAAACCGCCTGCGGCACGCTTTACGC

CAGTAATTCCGATTAACGCTTGCAACCCTACGTATTACCGCGGCTGCTGG

CAACGTAGTTAGCCGGTGCTTAATTCTTTACGGTAACGTCATGACCGGGG

GATAATTAAGCCCCATGCTTTTCGTTCGTACAAAAGCAGTTACAACCGAA

GGACCTTCATCCTGCCGCCGGGAATTGCTGGATCAGCCTTGCGCCCAATG

GTCAAAGTTCCAACTGCTGCCTCCGTAAGAGTTCTGGATCGGATTCAGTC

CAAGTGTGCTGAATCTGTCTTTCTGAAGAAACCAGCTAACGAAATATCGC

A

>SYC66

CATGCTAGTTTGTTACGACTTCACCCCAGTCACGAACCCCGCCGTGGTAA

GCGCCCTCCTTGCGGTTAGGCTACCTACTTCTGGCGAGACCCGCTCCCAT

GGTGTGACGGGCGGTGTGTACAAGACCCGGGAACGTATTCACCGTGACAT

TCTGATCCACGATTACTAGCGATTCCGACTTCACGCAGTCGAGTTGCAGA

CTGCGATCCGGACTACGACTGGCTTTATGGGATTAGCTCCCCCTCGCGGG

TTGGCAACCCTTTGTACCAGCCATTGTATGACGTGTGTAGCCCCACCTAT

AAGGGCCATGAGGACTTGACGTCATCCCCACCTTCCTCCGGTTTGTCACC

GGCAGTCCCATTAGAGTGCTCAACTGAATGTAGCAACTAATGGCAAGGGT

TGCGCTCGTTGCGGGACTTAACCCAACATCTCACGACACGAGCTGACGAC

AGCCATGCAGCACCTGTGTGCAGGTTCTCTTTCGAGCACCAAACCATCTC

TGGTAAGTTCCTGCCATGTCAAAGGTGGGTAAGGTTTTTCGCGTTGCATC

GAATTAAACCACATCATCCACCGCTTGTGCGGGTCCCCGTCAATTCCTTT

GAGTTTCAACCTTGCGGCCGTACTCCCCAGGCGGTCAACTTCACGCGTTA

GCTTCGTTACTGAGTCAGTTAAGACCCAACAACCAGTTGACATCGTTTAG

GGCGTGGACTACCAGGGTATCTAATCCTGTTTGCTCCCCACGCTTTCGTG

CATGAGCGTCAGTGCAGGCCCAGGGGATTGCCTTCGCCATCGGTGTTCCT

CCGCATATCTACGCATTTCACTGCTACACGCGGAATTCCATCCCCCTCTG

CCGCACTCTAGCTTTGCAGTCACAATGGCAGTTCCCAGGTTGAGCCCGGG

GATTTCACCACTGTCTTACAAAACCGCCTGCGCACGCTTTACGCCCAGTA

ATTCCGATTAACGCTTGCACCCTACGTATTACCGCGGCTGCTGGCACGTA

GTTAGCCGGTGCTTATTTCTTACGGTACGTCATGACCGAGATATTAGCCC

CAGCCTTTCGTCGTACAAAAGCAGTTACACCCGAGGTCTCATCTGCCGCG

CATGGCTGATCAGGCTTTCGCATGTCAAATCCCCATGCTGCTTCGTAGAG

TCTGGGCCAGTTTCAGTTCCAGTGGCTGATCGTCTCTCGAACAGGCTACG

GATCGCATGGCCTGA

>SYC67

GAGGGGTACTTGTTACGACTTCACCCCAGTCACGAACCCCGCCGTGGTAA

GCGCCCTCCTTGCGGTTAGGCTACCTACTTCTGGCGAGACCCGCTCCCAT

GGTGTGACGGGCGGTGTGTACAAGACCCGGGAACGTATTCACCGTGACAT

TCTGATCCACGATTACTAGCGATTCCGACTTCACGCAGTCGAGTTGCAGA

CTGCGATCCGGACTACGACTGGCTTTATGGGATTAGCTCCCCCTCGCGGG

TTGGCAACCCTTTGTACCAGCCATTGTATGACGTGTGTAGCCCCACCTAT

AAGGGCCATGAGGACTTGACGTCATCCCCACCTTCCTCCGGTTTGTCACC

GGCAGTCCCATTAGAGTGCTCAACTGAATGTAGCAACTAATGGCAAGGGT

TGCGCTCGTTGCGGGACTTAACCCAACATCTCACGACACGAGCTGACGAC

AGCCATGCAGCACCTGTGTGCAGGTTCTCTTTCGAGCACCAAACCATCTC

TGGTAAGTTCCTGCCATGTCAAAGGTGGGTAAGGTTTTTCGCGTTGCATC

GAATTAAACCACATCATCCACCGCTTGTGCGGGTCCCCGTCAATTCCTTT

GAGTTTCAACCTTGCGGCCGTACTCCCCAGGCGGTCAACTTCACGCGTTA

GCTTCGTTACTGAGTCAGTTAAGACCCAACAACCAGTTGACATCGTTTAG

GGCGTGGACTACCAGGGTATCTAATCCTGTTTGCTCCCCACGCTTTCGTG

CATGAGCGTCAGTGCAGGCCCAGGGGATTGCCTTCGCCATCGGTGTTCCT

CCGCATATCTACGCATTTCACTGCTACACGCGGAATTCCATCCCCCTCTG

CCGCACTCTAGCTTTGCAGTCACAATGGCAGTTCCCAGGTTGAGCCCGGG

GATTTCACCACTGTCTTACAAAACCGCCTGCGCACGCTTTACGCCCAGTA

ATTTCCGATTAACGCTTGCACCCTACGTATTACCGCGGCTGCTGGCACGT

AGTTAGCCGTGCTTATTCTTACGGTACGGTCATGACCGAGGTATTAGCCC

CAAGCCTTTTCGTTCGTACAAAAGCAGTTTAACAACCGAGGGCTCATCTG

CACGCGGCATGCTGATCAGCCTTCGCCATGGTCAAATCCCCACTGCTGCT

CCGTAGAGTCTGACGGTTTCAGTTCCAAGTGACTGAATCGTCTCTCGAAC

AGCATACGATCGCCAGGCCTTGG

>SYC68

TTGGGGGGGGCCTTCACTTGCAGGTCGAAGCGGTAGCAGAGAGAGGGTGG

CTTACTATGGCGAGGGGGGGGAGGTAAAGAATGAGAGTGAGCCTGCCTGA

GGGAGAAAAGACCTACGGGGAACGGTACCTAATAATATCTGAGGAGGAGA

ACGGAGGAGATGTACGCTTCTTGCGCTTGCAGATGAATATGGGCCAATGG

GACTATGGGGGGGGAGAAGGTTACGCTCACGAGGAGATGGAGCTCTGGTG

GGAGTGAGAGGATCCACACTGTCACTGAGACACAGACCCGATACCCACTC

CTGGGGGAGGTGGGGAAGTGTGTAATTTGTGCGAAAGCCTGATCCTGAAG

TGCCGCTTGCACGAGGATGGCCTACGGGTTGTAAACTGCTTTTTTTTTCA

ACGAAGAGGAATCTCCTAATGTTAAAAGCCCTTGACTTTTTTTTTACACT

CAGCAGAGGCTAACTCGGACTCCGTGGCCGCGCCACACTTATGCTGAGAG

CGTTAATCGGAATTTCAGGGCTCGGGGGTGCGCGTCGGGGTGTGTTACAA

ATTATGAATTCAAATCCTCCTCCTCGGAACTGCATTTGTGTATGCATGTG

TCTGTACGATTGTATGTGGATGGAGGTACACCCTGTGGAGTGGCGTGCTA

ATAATACAGAGCAGGACCAATAGCGAAGGCGATCACCTGCCCCTGTACTA

ACACTCATGCTCTGGAGCGCGGGGGGGAGAAAACAATCATAAAATCCTAT

GCTCGTCGCCCCCGCAGATGACGACTGCTACTTGGTGGTTACCTTTTTAT

TTTACGACGTCAGCACATGAAGTAGATCGACCGGCCGCGTGCGGACGGCA

GGCTTGAAGTTAAAACAATTAATTGGTTACCGGCCCCCCCCGTGGTGGAG

CGGGTTGTTTCTTTGTACCCACCACACCAACCCCACCTGTGCTTCGCTGA

AATTACATTATATAGAAGATTGGTTGCATAGACACTGACTACAAGAGCGC

GCTGCGGTGGTCTCATCTCGTCTGGTAGAGTTAGGTTAAGTTCACCCACC

AGGACCAACCCCATTCCTTATTTTTTTTTACTTTCCAGTTGTATCGGCTC

GCGAAAGCCTTCTGGGGAAAAGTTGCGGAGACCTGAATCCAAGGGCGAGG

GAATGGTGGCGTTGAAGTCTCCACATGGTCTCCTTGATGCCCTTGCGGGG

CCTTAGGTCGCTTCTCTACCAAGTTGCCTTAGGGTGCGGGATGTATATGG

ACCGGAATCGACACTAGACGCGCGCTGTCG

>SYC69

AGGGGGGGTTCTTTGTTACGACTTTACCCCAGTCATGATCACACCGTGGT

AACCGTCCTCCCGAAGGTTAGACTAGAAAAGAAAAGGGCAACCCACTCCC

ATGGTGTGACGGGCGGTGCGCACAAGGCCCGGGAACGTATTCACCGCGAC

ATTCTGATTCGCGATTACTAGCGATTCCGACTTCACGCAGTCGAGTTGCA

GACTGCGATCCGGACTACGATCGGTTTTGTGAGATTAGCTCCCCCTCGCG

GCTTGGCAACCCTCTGTACCGACCATTGTAGCACGTGTGTAGCCCAGGCC

GTAAGGGCCATGATGACTTGACGTCATCCCCACCTTCCTCCGGTTTGTCA

CCGGCAGTCTCCTTAGAGTGCCCACCATAACGTGCTGGTAACTAAGGAAA

AGGGTGGCGCTCGTTACGGGACTTAACCCAACTTCTCACGACACAAGTTG

ACAACAGCCTTGCACCACCGGTGTCAGAGTTCCCAAAGGCACCAATCCAT

CTCTGGAAAGTTCTCTGCATGTCAAGGCCTGGTAAGGTTCTTCGCGTTGC

TTCAAATTAAACCACATGCTCCACCGCTTGTGCGGGCCCCCGTCAATTCA

TTTGAGTTTTAACCTTGCGGCCGTACTCCCCAGGCGGTCAACTTAATGCG

TTAGCTGCGCCACTAAAATCTCAAGGATTCCAACGGCTAGTTGACATCGT

TTACGGCGTGAACTACCAGGGAATCTAATCCTGTTGCTCCCCACGCTTTC

CCACCTCAATGTCAGCATCGTCAGGTGGTCGCTTTCGCCCCCGGGAGTTC

CCTTCCAATATCTACGCATTTCACCGCTAACCAGGAAATCCCACCACCCT

TCTACCCGTACTCTAGCTTGCCAGTTTTAGGATGAAGTTCCCAGGGTAGA

CCCGGGGGCTTTCACATTCCAATTTAACAAACCCACCTACGCGCGGCTTA

ACGCCCAGTAATTCCGAATTAACGGTTTGCCCCCTTCTGATATTACCGCC

GGTCTGCTTGGCACAGAAGTTAGGCCGGGTGCTTTATTCTGGTCGGTAAC

GCTCAAAAACAAGAAGTGTATTACCCTTAACTGGCCCTTTCCCTCCCCAA

ATTAAAGTTGCCTTTAAAACTCCGAGAGAACCTTTCCTTTCACAACACGG

CGGACGAGGCCTGGGGAT

>SYC70

CCCTAAGGGATCCTAACCATGCGTGTCGAGTCGTCGCCTGAGAGTGCATT

ACCAGGAGCGTAGCTCATCCCACGTAGGCACATCTGACGGCCCGGGGCTT

TGGGACGACTGCGTTCTCCGGAGAGGGGTCTATAGAAAAAGCTGCGTAAC

AAAAAAAGTTTCCGACGGGCCAAGCTCGATACCTAGGACTTACCCCCTAG

TACCTACATTGGACCGAAGGAAAGCGCGGAAGCGGGGGACGATTACTATC

TGGACAGGAAGCACACTCTTCCTCACCCTGACCCAGGATCAACCCTTTTC

TATACGGGAGGCAGCGGGGTTGAATATTGCACAACGGGCGCAAGCCTGAT

GCACCCCTGCCGCGTGTATGAAGAAACCTTCGGGTTGTAAATTACTTTCA

GCGAGGAGGAAGGCGATTTTTTAATAACCGCTTTTTTTTTTTTTTTACTC

GCAGAAGAGGGGGGGGGCTATCTCCCTGCCCTTCAGCCGTGGTAATACGG

AGGGTGCAAGTGTTAATCGGAAGGGGTGGGCGTAAAGCGCACGCAGGCGG

TCTGTCAAGTCGGATGTGAAACCCCCCGGCTCAACCCTGGGAACTG

>SYC71

TGGATAGTTGTTAAAACTGCACTAGTCATCAGTCCACCTTAGGCGGCGGG

CTCCAAAAAGGTTACCCCACAGACTTCGGGTGTTACAAACTCTCGTGGTG

TGACGGGCGGTGTGTACAAGGCCCGGGAACGTATTCACCGCGGCATGCTG

ATCCGCGATTACTAGCGATTCCAGCTTCATGTAGGCAAGTTGCAGCCTAC

AATCCGAACTGAAAACGGTTTTATGAGATTAGCTCCACCTCGCGGTCTTG

CAGCTCTTTGTACCGTCCATTGTAGCACGTGTGTAGCCCAGGTCATAAGG

GGCATGATGATTTGACGTCATCCCCACCTTCCTCCGGTTTGTCACCGGCA

GTCACCTTAGAGTGCCCAACTTAATGATGGCAACTAAGATCAAGGGTTGC

GCTCGTTGCGGGACTTAACCCAACATCTCACGACACGAGCTGACGACAAC

CATGCACCACCTGTCACTCTGCTCCCGAAGGAGAAGCCCTATCTCTAGGG

TTTTCAGAGGATGTCAAGACCTGGTAAGGTTCTTCGCGTTGCTTCGAATT

AAACCACATGCTCCACCGCTTGTGCGGGCCCCCGTCAATTCCTTTGAGTT

TCAGCCTTGCGGCCGTACTCCCCAGGCGGAGTGCTTAATGCGTTAACTTC

AGCACTAAAAGGGCGGAAACCCTCTAACACTTAGCACTCATCGTTTACGG

CGTGGACTACCAGGGTATCTAATCCTGTTTGCTCCCCACGCTTTCGCGCC

TCAGTGTCAGTTACAGACCAGAGAGTCGCCTTCGCCACTGGTGTTCCTCC

ATATCTCTACGCATTTCACCGCTACACATGGAAATTCCACTTTCCTCTTC

TGCACTCAAGTCTGCCAGTTTCCAATGACCCTCCACGGTTGAGCCGTGGC

TTTCACATCAGACTGAAGAAACCACCTGCGCGCGCTTTACGCCCAATAAT

TCCGAGATAACGCTTGACACCTTACGTATTACGCCGCTGCTGCACGTAGT

TAGCCGTGCTTTTCTGGTTAGGTACCGTCAAGTGCAGCTTATTACACCTA

GGACTTGGTTCCTTCCCTAACAACGGAGTTTATACGACCGGAGAGCCTTT

CTTCGACTCCGCGCGATGGTTTGTGTCTCTCGGACTTCCGTCTATTGCGA

GATTCCCTTTCTGTCTGCTTCGTAAGGATCTTAGCCGTTTCCTCAAGTCG

AGTGTGACCGATTTCACCTCTCTATAACGCGTTCAGTGCTATGACGACTT

ACGAGATGCATCG

>SYC72

CGCCGGTGCCTTGGGTTACGAATTTCCCCCAGTCTGAATCACAAAAGTGG

TAAGCGCCCTCCCGAAGGTTAAACTAAAAACGTGAGGGTGCAACCCACTC

CCATGGTGTGACGGGCGGTGAGAACAAGGCCCGGGAACGTATTCACCGTA

ACATTCTGATCTACGATTACTAGCGATTCCGACTTCATGGAGTCGAGTTG

CAGACTCCAATCCGGACTACGACATACTTTATGAGGTCCGCTTGCTCTCG

CGAGTTGGCTTCTCTTTGTATATGCCATTGTAGCACGTGTGTAGCCCTAC

TCGTAAGGGCCATGATGACTTGACGTCATCCCCACCTTCCTCCGGTTTAT

CACCGGCAGTCTCCTTTGAGTTCCCACCATTACGTGCTGGCAACAAAGGA

TAAGGGTTGCGCTCGTTGCGGGACTTAACCCAACATTTCACAACTCGTTT

CTGACGACAGCCATGCAGCACCTGTCTCACGGTTCCCGAAGGCACTAAGC

CATCTCTGGCGAATTCCGTGGATGTCAAGAGTAGGTAAGGTTCTTCGCGT

TGCATCGAATTAAACCACATGCTCCACCGCTTGTGCGGGCCCCCGTCAAT

TCATTTGAGTTTTAACCTTGCGGCCGTACTCCCCAGGCGGTCGACTTAAC

GCGTTAGCTCCGGAAGCCACACCTCAAGGGCACAACCTCCTAGTCGACAT

CGTTTACAGCGTGGACTACCAGGGTATCTAATCCTGTTTGCTCCCCACGC

TTTCGCACCTTGAGCGTCAGTCTTTGTCCAGGGGGCCGCCTTCGCCACCG

GTATTCCTCCAGATCTCTACGCATTTCACCGCTACACCTGGAATTCTACC

CCCCTCTACAAGACTCTAGCTTTGCCAGTTTCAATGCAGTTCCCACGTTA

AGCGCGGGGATTTCCATCTGACTTACAACCCCCTGCGTGCGCTTTACGCC

CAGTAATTCGATTAACGCTTGCACCCCTCCGTATTACTGCGGCTGCTGGG

ACGGAGTTAAGCGGTGCCTTCTTCTGGCGAGGCAGGTCAATCACCACAAC

GTAATAAGGATGGACAGCCATCCCCCCTCGCTGAAGGTGCTTTACAACCC

GTAAGGACCTTCTCTCACAAACACGCGGCATGGCGTGCATTACAGGGC

>SYC73

TGGCGAGGGGTGACTGGTTACAACTTCACCCCAGTTCATGATCACAAAGT

GGTAAGCGCCCTCCCGAAGGTTAAGCTACAACAAGATTTGCAACCCACTC

CCATGGTGTGACGGGCGGTGTGTACAAGGCCCGGGAACGTATTCACCGTA

GCATTCTGATCTACGATTACTAGCGATTCCGACTTCATGGAGTCGAGTTG

CAGACTCCAATCCGGACTACGACATACTTTATGAGGTCCGCTTGCTCTCG

CGAGTTCGCTTCTCTTTGTATATGCCATTGTAGCACGTGTGTAGCCCTAC

TCGTAAGGGCCATGATGACTTGACGTCATCCCCACCTTCCTCCGGTTTAT

CACCGGCAGTCTCCTTTGAGTTCCCACCATTACGTGCTGGCAACAAAGGA

TAAGGGTTGCGCTCGTTGCGGGACTTAACCCAACATTTCACAATACGAGC

TGACGACAGCCATGCAGCACCTGTCTCACGGTTCCCGAAGGCACTAAGCC

ATCTCTGGCGAATTCCGTGGATGTCAAGAGTAGGTAAGGTTCTTCGCGTT

GCATCGAATTAAACCACATGCTCCACCGCTTGTGCGGGCCCCCGTCAATT

CATTTGAGTTTTAACCTTGCGGCCGTACTCCCCAGGCGGTCGACTTAACG

CGTTAGCTCCGGAAGCCACGCCTCAAGGGCACAACCTCCAAGTCGACATC

GTTTACAGCGTGGACTACCAGGGTATCTAATCCTGTTTGCTCCCCACGCT

TTCGCACCTGAGCGTCAGTCTTTGTCCAGGGGGGCCGCCTTCGCCACCGG

TATTCCTCCAGATCTCTACGCATTTCACCGCTACACCTGGAATTCTACCC

CCCTCTACAAGACTCTAGCTTGCCAGTTTCAAATGCAGTTCCCACGTTAA

GCGCGGGGATTTCACATCTGACTTAACAACCGCCTGCGTGCGCTTTACGC

CCAGTAATTCCGATTAACGCTTGCACCCTCCGTATTACCGCGCTGCTGGC

ACGGAGTTAGGCCGGTGCTTCTTCTGGCGAGTAACGTCCATCACCACACG

TATAAAGGGTGGATGCCTTTCCTCCTTCGCTGAAAGTGCTTTACAACCCT

AAGGCCTTCTCCCACACACGCGGCATGGCTGGCATCAGGCTTTGCGCCCA

TTGGGCAAATATTCCCAATGCTGCCTCCGTAGGAGTCTGGACGGGCCTCA

GTTCCAGGTGGCTGGCCTCCTCTCGAACGGCCTAGGTAATCGCTCGCCTA

GGGTGTGAACCCATTAATCGTCA

>SYC74

CTATGGGGGGGGCCTAAACATGCAAGTCGAGCGATGGATTGAGAGCTTGC

TCTCAAGAAGTTAGCGGCGGACGGGAGAGTAACACGTGGGTAACCTGCCC

ATAAGACTGGGATAACTCCGGGAAACCGGGGCTAATACCGGATAACATTT

TGAACTGCATGGTTCGAAATTGAAAGGCGGCTTCGGCTGTCACTTATGGA

TGGACCCGCGTCGCATTAGCTAGTTGGTGAGGTAACGGCTCACCAAGGCA

ACGATGCGTAGCCGACCTGAGAGGGTGATCGGCCACACTGGGACTGAGAC

ACGGCCCAGACTCCTACGGGAGGCAGCAGTAGGGAATCTTCCGCAATGGA

CGAAAGTCTGACGGAGCAACGCCGCGTGAGTGATGAAGGCTTTCGGGTCG

TAAAACTCTGTTGTTAGGGAAGAACAAGTGCTAGTTGAATAAGCTGGCAC

CTTGACGGTACCTAACCAGAAAGCCACGGCTAACTACGTGCCAGCAGCCG

CGGTAATACGTAGGTGGCAAGCGTTATCCGGAATTATTGGGCGTAAAGCG

CGCGCAGGTGGTTTCTTAAGTCTGATGTGAAAGCCCACGGCTCAACCGTG

GAGGGTCATTGGAAACTGGGAGACTTGAGTGCATAAGAGGAAAGTGGAAT

TCCATGTGTAGCGGTGAAATGCGTACAGATATGGAGGAACACCAGTGGCG

AAGGCGACTTTCTGGTCTGTAACTGACACTGAGGCGCGAAAGCGTGGGGA

GCAAACAGGATTAGATACCCTGGTAGTCCACGCCGTAAACGATGAGTGCT

AAGTGTTAGAGGGTTTCCGCCCTTTAGTGCTGAACGTCAACGCATTAAGC

ACTCCGCCTGGGGAGTACGGCCGCAAGGCTGAACTCAAGGAATTGACGGG

GGCCCGCACAAGCGGTGGAGCATGTGGTTTAATTCGAAGCCACCGCGAAA

ACCTTACCAGGTACTTGACATCCTCTGAAAACCCTAGAGATAGGGCTTCC

TCCTTTCGGGAGCAGAGTGGAAGGTGGTGCATGGTTGGTCGACAGCTCGT

GTCGTGAGATGTTGGGCTCAGTCCCGCCACTAACCCTATCCCTTTGATCT

TAGTTGCCATCATTTAGTTTGGGGACCTCCTAAAGCTGGAACTGCCGGGT

GAGCATGCGGAGGAGCGTGGGATTGACTTTCAGTTCAGTCCATGCCCTTT

ATGACTTGGGCCTACCACTCTGTGCTGACATTGGACCGTGTACACAAAGG

CAGCGTTTGACTCAGTAACAGCG

>SYC75

ACCCCTTGGCCAGGTCGGAGCGGCAGCGGAAGTAGCTTGCTACTTTGCCG

GCGAGCGGCGGAAGGAAGAGTAATGTCTGGGAAACTGCCTGATGGAGGGG

GATAACTACTGGAAACGGTAGCTAATACCGCATGACCTCGAAAGAGCAAA

GTGGGGGATCTTCGGACCTCACGCCATCGGATGTGCCCAGATGGGATTAG

CTAGTAGGTGAGGTAATGGCTCACCTAGGCGACGATCCCTAGCTGGTCTG

AGAGGATGACCAGCCACACTGGAACTGAGACACGGTCCAGACTCCTACGG

GAGGCAGCAGTGGGGAATATTGCACAATGGGCGCAAGCCTGATGCAGCCA

TGCCGCGTGTGTGAAGAAGGCCTTAGGGTTGTAAAGCACTTTCAGCGAGG

AGGAAGGCATCACACTTAATACGTGTGGTGATTGACGTTACTCGCAGAAG

AAGCACCGGCTAACTCCGTGCCAGCAGCCGCGGTAATACGGAGGGTGCAA

GCGTTAATCGGAATTACTGGGCGTAAAGCGCACGCAGGCGGTTTGTTAAG

TCAGATGTGAAATCCCCGCGCTTAACGTGGGAACTGCATTTGAAACTGGC

AAGCTAGAGTCTTGTAGAGGGGGGTAGAATTCCAGGTGTAGCGGTGAAAT

GCGTAGAGATCTGGAGGAATACCGGTGGCGAAGGCGGCCCCCTGGACAAA

GACTGACGCTCAGGTGCGAAAGCGTGGGGAGCAAACAGGATTAGATACCC

TGGTAGTCCACGCTGTAAACGATGTCGACTTGGAGGTTGTGCCCTTGATG

CGTGGCTTTCCGGAGCTAACGCGTTAAGTCGACCGCCTGGGGAGTACCGC

CGCAAGGTTAAAACTCAAATGAATTGACGGCGCCCGCCACAAGCGGTGGA

GCATGTGGTTTAATTCGATGCACGCGAAGTACCTTACCTACTCTTGACAT

CCACTGAATTCGCCAGAAATGGCTTATTGCCTTCGTAACCGTGAGACGGT

GCTGCATTGCCTGTCGTCAGCTCGTGTTAGTGAAATGGTTGGGTTAACGT

TCCCGCAAACGAGCGCTAGCCCTTTATCCTTTCGTTGCCAGCTCGTAAAC

GGTGGGTACCTCAAAGGGACACTGCCCGGAGAAATAAACCGGAGGGATGT

GTGGTGATTAACGTTCAAGTCTCGTCATGGCCCCTTTACTCAGCTAAGGG

CCTACCAT

>SYC76

CCGGTGCGAAGGCCTAGACATGCAAGTCGAGCGGCAGCGGAGAGTAGCTT

GCTACTTTGCCGGCGAGAGGCGGAAGGGTGAGGAATGTCTGGGAAACTGC

CTGATGGAGGGGGATAATTACTGGAAACGGTAGCTAATACCGCATGACCT

CGAAAGAGCAAAGTGGGGGATCTTCGGACCTCACGCCATCGGATGTGCCC

AGATGGGATTAGCTAGTAGGTGAGGTAATGGCTCACCTAGGCGACGATCC

CTAGCTGGTCTGAGAGGATGACCAGCCACACTGGAACTGAGACACGGTCC

AGACTCCTACGGGAGGCAGCAGTGGGGAATATTGCACAATGGGCGCAAGC

CTGATGCAGCCATGCCGCGTGTGTGAAGAAGGCCTTAGGGTTGTAAAGCA

CTTTCAGCGAGGAGGAAGGCATCACACTTAATACGTGTGGTGATTGACGT

TACTCGCAGAAGAAGCACCGGCTAACTCCGTGCCAGCAGCCGCGGTAATA

CGGAGGGTGCAAGCGTTAATCGGAATTACTGGGCGTAAAGCGCACGCAGG

CGGTTTGTTAAGTCAGATGTGAAATCCCCGCGCTTAACGTGGGAACTGCA

TTTGAAACTGGCAAGCTAGAGTCTTGTAGAGGGGGGTAGAATTCCAGGTG

TAGCGGTGAAATGCGTAGAGATCTGGAGGAATACCGGTGGCGAAGGCGGC

CCCCTGGACAAAGACTGACGCTCAGGTGCGAAAGCGTGGGGAGCAAACAG

GATTAGATACCCTGGTAGTCCACGCTGTAAACGATGTCGACTTGGAGGTT

GTGCCTTTGAGGCGTGGCTTCCGGAGCTAACGCGTTAAGTCGACCGGCCT

GGGAGTACGGCCGCAAGGTTAAACTCAAATGATTGACGGGGGCCCGCACA

AGCGGTGGAGCATGTGGTTTAATTCGATGCAACGCGAGACCCTTACCTAC

TCTTGACATCCACGGAATTCGCCAGAGATGGCTTATTGCCTTCGGGAACG

TGAGACAGGTGCTGGCATGGCTGTCGTCAG

>SYC77

GCAGTTAAACGGCCTCGCTGCAGCTCGAGCGGCAGTCGAAGAGGTGGTCT

CGGGTGAGAGTGGGGGGCGGGGGAGACGTGTCTGGGAAATGCGTGAGGGA

GGGGGGTATCTCTAGGGAACGGGGGCTAATACCGCGTATGGTCGCAAGAG

CAAAGAGGGGGACCTTCTGGGCTCTTGCCCTCTCATGTGCCCAGATGGGA

TTAGCTATTAGGTGGGGTAATGGGTCACCTATGCGACAATCCCTAGCTGG

GCTGAGAGGATGACCAGCCACACTGGAACTGAGACACGGTCCAGACTCCT

ACGGGAGGCAGCACTGGGGAATATTGTGCAATGGGCGCAAGCCTGATGCA

CCCATGCCCCGTGTATGAAAAAAGCCTTCTGGTTGTAAAGTACTTTCTCC

GAGGAGGAAAGGGCTGAGGTTAATAACCTTATGGATTGTCGTTTCTCGCA

CAAAAAACACCGGGTAACTCCGTGCCAGCACCCGCGGTAATACAGAGGGT

GCAAGCGTTAATCTCAATTACTGGGCGTAAAGCGCACGCACGCGGTCTGT

CAAGTCTGATATGAAATCCCCGGGCTCTACCTGGGAACTGCGTTCTAAAC

TGTGAGGCTATAGTCTTGTGTAGGGGGGTAGAATTCCCCGTGTATCGGTG

AGATGCGTATAGATCTGGAGGAATACCGGTGGCGAAAGCGGCCCCCTGTA

CAAAGACTGACACTCACGTGCGAAAGCGTGTGGAGCACACAGGATTATAT

ACCCTGTGAGTCCACGCCCTATACGATGTCTACTTGTAGGTTGTTCCCTT

GAGGAGTGGCTTCCGCAGCTAACACGTTATGTCTACAGCCTGTGGAGTAC

AGCCGCAAGGTTAAAACTCTAATGAATTGTCGGGGGCCCGCACAAGCGGT

GGAGCATGTGGTTTATTTCGATGTACGCGAAGAACATTACCTACTCTCGA

CGTCTCGGGATTTACACAGATGCTTTTATGTCTTCCTGGAACCGTGAGAC

AGTGCTGCTATGGCTGTCGTCAGCTCTCGTTGTGAAAAGTTGGGTTAAAT

CCCCACCAAGCGCAACCTTATTCCCTCTGTTTGCCGCGGATCTGACTGGG

GAACTACATAGGGAAGAAATGACTCGTTGATAGACTGGCAGGAAGGGGGG

GATGACTGTCAGTCATCATGGGCCCTCACGACTAGGTTACATCTGCTTCA

ATGACGTTATTCAACGAGAGACGAGCCCTACTCTCGGAGAGACCAAGCCG

TGA

>SYC78

ATTTGCGGGGGCCTACCATGCAGTCGAGCGGTAGCACAGAGAGCTTGCTC

TCGGGTGACGAGCGGCGGACGGGGGAGTAATGTCTGGGAAACTGCCTGAG

GGAGGGGGATAACTACTGGAAACGGTAGCTAATACCGCATAACGTCGCAA

GACCAAAGAGGGGGACCTTCGGGCCTCTTGCCATCAGATGTGCCCAGATG

GGATTAGCTAGTAGGTGGGGTAATGGCTCACCTAGGCGACGATCCCTAGC

TGGTCTGAGAGGATGACCAGCCACACTGGAACTGAGACACGGTCCACACT

CCTACGGGAGGCAGCAGTGGGGAATATTGCACAATGGGCGCAAGCCTGAT

GCAGCCATGCCGCGTGTATGAAGAAGGCCTTCGGGTTGTAAAGTACTTTC

AGCGAGGAGGAAGGCGTTGAGGTTAATAACCTTATTGATTGACGTTACTC

GCAGAAGAAGCACCGGCTAACTCCGTGCCAGCAGCCGCGGTAATACGGAG

GGTGCAAGCGTTAATCGGAATTACTGGGCGTAAAGCGCACGCAGGCGGTC

TGTCAAGTCGGATGTGAAATCCCCGGGCTCAACCTGGGAACTGCATTCGA

AACTGGCAGGCTAGAGTCTTGTAGAGGGGGGTAGAATTCCAGGTGTAGCG

GTGAAATGCGTAGAGATCTGGAGGAATACCGGTGGCGAAGGCGGCCCCCT

GGACAAAGACTGACGCTCAGGTGCGAAAGCGTGGGGAGCAAACATGATTA

TATACCCTGGTAGTCCACGCCGTATACGATGTCTATTTGTAGGTTGTTCC

CTTGAGGAGTGGGTTCCGGAGCTAACGCGTTAAGTCTACCGCCTGGGGAG

TACGCCGCAAGGTTAAAACTCACATGAATTGACGGGGGCCCGCACAAGCG

GTGGAGCATGTG

>SYC79

GGGCGGCGTCTTTCTTTGGTTACGACTTCACCCCAGTCATGATCACAAAA

GTGGTAAGCGCCCTCCCGAAGGTTAAGCTAAAACAAACTTTTGCAACCCA

CTCCCATGGTGTGACGGGCGGTGTGTACAAGGCCCGGGAACGTATTCACC

GTAGCATTCTGATCTACGATTACTAGCGATTCCGACTTCATGGAGTCGAG

TTGCAGACTCCAATCCGGACTACGACATACTTTATGAGGTCCGCTTGCTC

TCGCGAGTTCGCTTCTCTTTGTATATGCCATTGTAGCACGTGTGTAGCCC

TACTCGTAAGGGCCATGATGACTTGACGTCATCCCCACCTTCCTCCGGTT

TATCACCGGCAGTCTCCTTTGAGTTCCCACCATTACGTGCTGGCAACAAA

GGATAAGGGTTGCGCTCGTTGCGGGACTTAACCCAACATTTCACAACACT

AGCTGACGACAGCCATGCGGCACCTGTCTCACGGTTCCCGAAGGCACTAA

GCCATCTCTGGCGAATTCCGTGGATGTCAAGAGTAGGTAAGGTTCTTCGC

GTTGCATCGAATTAAACCACATGCTCCACCGCTTGTGCGGGCCCCCGTCA

ATTCATTTGAGTTTTAACCTTGCGGCCGTACTCCCCAGGCGGTCGACTTA

ACGCGTTAGCTCCGGAAGCCACGCCTCAAGGGCACAACCTCCAAGTCGAC

ATCGTTTACAGCGTGGACTACCAGGGTATCTAATCCTGTTTGCTCCCCAC

GCTTTCGCACCTGAGCGTCAGTCTTTGTCCAGGGGGCCGCCTTCGCCACC

GGTATTCCTCCAGATCTCTACGCATTTCACCGCTACACCTGGAATTCTAC

CCCCCTCTACAAGACTCTAGCTTGCCAGTTTCAAATGCAGTTCCCACGTT

AGGCGCGGGGATTTCACATCTGACTTAACAAACCGCCTGCGTGCGGCTTT

ACGCCCAGTAATTCCGATTAACGCTTGCACCCTCGTATTACCGCGGCTGC

TGGGCACGGAGTTAGCCGGGTGCTTCTTCTGCAGTAACGTCATCACCAAC

AACGTATTAAGTGTGAGATGCCTTTCCTCCTCGCTGAAAGGTGCTTTTAA

AAGCTCTTAAAGACCTTCTCCAACACACCGCGCAAGGCTGCATACAGGCT

TGGCGCCCATTGGGCCAAAATTCCCCCCGTGGCTTGCCTCCCTGAGGGCT

CTGGAACCGGTCTCATTTCCAAGGGTGCCGTTGTTATCCCCTCTCTCAGA

ACCGACCTATGGATGGATCGGCTCGGCCCTAAGAGT

>SYC80

TGGGCCCTAAGGGACTTTTTTACGACTTCACCCCAGTCATGAATCACAAA

GTGGTAAGCGCCCTCCCGAAGGTTAAGCTACAACTTCTTTTGCAACCCAC

TCCCATGGTGTGACGGGCGGTGTGTACAAGGCCCGGGAACGTATTCACCG

TAGCATTCTGATCTACGATTACTAGCGATTCCGACTTCATGGAGTCGAGT

TGCAGACTCCAATCCGGACTACGACGTACTTTATGAGGTCCGCTTGCTCT

CGCGAGTTCGCTTCTCTTTGTATACGCCATTGTAGCACGTGTGTAGCCCT

ACTCGTAAGGGCCATGATGACTTGACGTCATCCCCACCTTCCTCCGGTTT

ATCACCGGCAGTCTCCTTTGAGTTCCCGACCGAATCGCTGGCAACAAAGG

ATAAGGGTTGCGCTCGTTGCGGGACTTAACCCAACATTTCACAACACGAG

CTGACGACAGCCATGCAGCGCCTGTCTCAGAGTTCCCGAAGGCACTAAGC

TATCTCTAGCGAATTCTCTGGATGTCAAGAGTAGGTAAGGTTCTTCGCGT

TGCATCGAATTAAACCACATGCTCCACCGCTTGTGCGGGCCCCCGTCAAT

TCATTTGAGTTTTAACCTTGCGGCCGTACTCCCCAGGCGGTCGATTTAAC

GCGTTAGCTCCGGAAGCCACGCCTCAAGGGCACAACCTCCAAATCGACAT

CGTTTACAGCGTGGACTACCAGGGGTATCTAATCCTGTTTGCTCCCCACG

CTTTCGCACCTGAGCGTCAGTCTTTGTCCAGGGGGCCGCCTTCGCCACCG

GTATTCCTCCAGATCTCTACGCATTTCACCGCTACACCTGGGAATTCTAC

CCCCCTCTACAAGACTCTAGCTTGCCAGTTTCAAATGCAGTTCCCACGTT

AAGCGCGGGGATTCCACATCTGACTAAACAAACCGCCTGCGTGCGCCTTA

CGCCCAGTAATTCCGATTAACGGCTTGCACCCTCCGGTATTACCGCCGGC

TGCTGGCACGGAGTTTAGCCGGTGGCTCTTCTGCGAGTAACGTCCATGGA

CCAGTGGCTTATAACACTGAAACCTTTCCTCCTCGCTGAAAGTTGCTTAC

AACCCTAAGGCCTTTTCCCACACGCGGCATGCCTGCATTCGGGCTGCGCC

CATGTGCAATGTCCCCCCACTTGCCGTGCTCCCGTTGGAGTCTGGAACCG

GTACTGCTAAGAGTTGTCTCCAACAAGTGTGGCTTGGCACATG

>SYC81

TCCGTGTGGTACCTTGGGTTACGACTTCCCCCAGTCATGAATCACAAAGT

GGTAAGCGCCCTCCCGAAGGTTAAGCTAGAACATCTTTTGCAACCCACTC

CCATGGTGTGACGGGCGGTGTGTACAAGGCCCGGGAACGTATTCACCGTA

GCATTCTGATCTACGATTACTAGCGATTCCGACTTCACGGAGTCGAGTTG

CAGACTCCGATCCGGACTACGACGCACTTTATGAGGTCCGCTTGCTCTCG

CGAGGTCGCTTCTCTTTGTATGCGCCATTGTAGCACGTGTGTAGCCCTGG

CCGTAAGGGCCATGATGACTTGACGTCATCCCCACCTTCCTCCGGTTTAT

CACCGGCAGTCTCCTTTGAGTTCCCGACATTACCCGCTGGAAACAAAGGA

AAAGGGTTGCGCTCGTTGCGGGACTTAACCCAACATTTCACAACACAAGT

TGACAACAGCCTTGCAGCACCGGTCTCAAGGTTCCCAAAGGCACTAACCC

ATCTCTGGAAAATTCGTTGAATGTCAAGGCCGGGTAAGGTTCTTCGCGTT

GCTTCAAATTAAACCACATGCTCCACCGCTTGTGCGGGCCCCCGTCAATT

CATTTGAGTTTTAACCTTGCGGCCGTACTCCCCAGGCGGTCAACTTAATG

CGTTAGCTCCGAAACCAACACCTCAAGGCCACAACCTCCAAGTCGACATC

GTTTACGGCGGGGACTACCAGGGTATCTAATCCGGTTTGCTCCCCACGCT

TTCGCACCTAAGCGTCAGTATCAGTCCGGGGGGCCGCCTTCGCCACGGGT

GTTCCTCCAATCTCTACGCTTTTCACCGCTACACTGGGAAATTCTACCCC

CTCTCTACAAAACTCTAGCTTGCCAGTTTAGAATGCAGTTCCCAGGGTTG

AGCCCGGGGGATTTCACATCGAACTTGACAACCGCCTGCGCGCGCTTTAC

GCCCAGAAATTCCAATTAAGCGCTTGCCCCCTCTGTATTACCGCGGCTGG

TGGCACGGATTACGCGGGGGCTTTTTTTCGGGGGTAACGTCATCGATAGA

GAGATTATAACCTTATCTGCCTTTCTCCCCACTGAAGGACTTACAACCCG

GAGGACCTTTCTTTCAACGCGCGCATGGCTGATAGGCTTGGGCCATTGGG

CCAAATTTCCCATTCTGGTGCCTCGGTAGAGATCTGGACCGTGTTTCAAG

TTCAGTGGAGCTGATACACACTCTCCTTCTCTCGAGAGCGGTTAGCGGGA

ATCTGCTCGCCCTATGTTGTGAGC

>SYC82

TCGTAACTTTTCCCCCCCACTTCCTGGAATCACAAAGTGGTAAGCGCCCT

CCCGAAGGTTAAGCTAGAAAAGGGGAGGTGCAACCCATTCCCATGGTGTG

ACGGGCGGTGTGAACAAGGCCCGGGAACGTATTCACCGTAGCATTCTGAT

CTACGATTACTAGCGATTCCGACTTCATGGAGTCGAGTTGCAGACTCCAA

TCCGGACTACGACATACTTTATGAGGTCCGCTTGCTCTCGCGAGTTCGCT

TCTCTTTGTATATGCCATTGTAGCACGTGTGTAGCCCTACTCGTAAGGGC

CATGATGACTTGACGTCATCCCCACCTTCCTCCGGTTTATCACCGGCAGT

CTCCTTTGAGTTCCCACCATTACGTGCTGGCAACAAAGGATAAGGGTTGC

GCTCGTTGCGGGACTTAACCCAACATTTCACAACACGAGCTGACGACAGC

CATGCAGCACCTGTCTCACGGTTCCCGAAGGCACTAAGCCATCTCTGGCG

AATTCCGTGGATGTCAAGAGTAGGTAAGGTTCTTCGCGTTGCATCGAATT

AAACCACATGCTCCACCGCTTGTGCGGGCCCCCGTCAATTCATTTGAGTT

TTAACCTTGCGGCCGTACTCCCCAGGCGGTCGACTTAACGCGGTTAGCTC

CGGGAAGCCACGCCTCAAGGGCACAACCTCCAAGTCGACATCGTTTACAG

CGTGGACTACCAGGGTATCTAAATCCTGTTTGCTCCCCACGCTTTCGCAC

CTGAAACGTCAGTCTTTGTCCAGGGGGCCGCCTTCGCCACCGGTATTCCT

CCAGATCTCTACGCATTTCACCGCTACACCTGGAATTCTACCCCCCTCTA

CAAGAATCCTAGCTTGCCAGTTTCCAATTGCAGTTTCCCACGTTAAGCGC

CGGGATTTCACTTCTGACTTAAACAAACCGCCCTGCGTGCGCCTTACCGC

CCAAGTAAATTCCGATTAACGGCTTGCACCCTTCCGTAATTACCGCGGCT

GCCTGGGCAAGGAAGTTAAGCCGGTGCTTTCTTCTGCCAAGTAACGTCAA

ATCAACACGACGTTATTAAGTGTGAATGCCCTCCCTCCTCACCTAAAAGT

GGCTTTACAATCCTAGGCCTGTCTTCCAGCAGCCGGAATGACTGCATCAG

CTTGCCGCAATGGGCAATAATTCCGACGGCGTGCTCCCGTAAGAAGTTCT

GGACACTGTGTCCTTAAGTTTCAAGTGGCCATGGTTCATCTC

>SYC83

GAAACATTGGCAAAGGTCCGAGGCGGCAGCGGAAAGTATGTTGCTACTTT

GCCGGGGAGCGGCGGAAGGGAGGGAAATGTCTGGGAAACTGCCTGATGGA

GGGGGATAACTACTGGAAACGGTAGCTAATACCGCATGACCTCGAAAGAG

CAGAGTGGGGGATCTTCGGACCTCACGCCATCGGATGTGCCCAGATGGGA

TTAGCTAGTAGGTGAGGTAATGGCTCACCTAGGCGACGATCCCTAGCTGG

TCTGAGAGGATGACCAGCCACACTGGAACTGAGACACGGTCCAGACTCCT

ACGGGAGGCAGCAGTGGGGAATATTGCACAATGGGCGCAAGCCTGATGCA

GCCATGCCGCGTGTGTGAAGAAGGCCTTAGGGTTGTAAAGCACTTTCAGC

GAGGAGGAAGGCATCACACTTAATACGTGTGGTGATTGACTTTACTCGCA

GAAGAAGCACCGGCTAACTCCGTGCCAGCAGCCGCGGTAATACGGAGGGT

GCAAGCGTTAATCGGAATTACTGGGCGTAAAGCGCACGCAGGCGGTTTGT

TAAGTCAGATGTGAAATCCCCGCGCTTAACGTGTGAACTGCATTTGAAAC

TGGCAAGCTAGAGTCTTGTAGAGGGGGGGTAGAATTCCAGGTGTAGCGGT

GAAATGCGTAGAGATCTGGAGGAATACCGGTGGCGAAGGCGGCCCCCTGG

ACAAAGACTGACGCTCAGGTGCGAAAGCGTGCGGAGCAAACAGGATTAGA

TACCCTGGTAGTCCACGCTGTAACGATGTCGACTTGGAGGTTGTGCCCTT

GATGCGTGGCTTCCCGAGCTAACGCGTTAACTCGACCGCCTGGGGAGTAC

CGGCGCAGGTTAAAACTCAATGAATTGACCGCGGGCCCGCACAGCGGTGG

AGCATGTGAGTTTAATTCGATGCAACGCGAAGTAACCTTGCCTAGTCTTG

ACATCCACTGTAATTCGCCAGAAATGGCTTCATTGCCTTACGGTAACCAT

GAGACAGGTGCTGGCTTGGCTGTCGTCAAGCTCGGGTTGGTGAATGTTTG

GGTTCAGTCCCCGCAACCAGCCGCAACCCTTATCCTTCGTTGCCAGCACG

GTAAGGGTGGCACTTCAAAGGGAGAACTGTGCAGGTAATAACGGAAAGGA

AGGTGGGATGACGTCAAGCTTATCTGGCCTTCCGAGTAGGCTACGCACGT

CAGCATTGCCATTAGCAATAGTACAAGCCGACCATCTCTGGCCGA

>SYC84

AGGTGGGGGGGGCCTAAAATGCAAGTCGAGCGGCAGCGGAAAGTAGGTTG

CTACTTTGCCGGCGAGCGGCGGAAGGGAGAGTAATGTCTGGGAAACTGCC

TGATGGAGGGGGATAACTACTGGAAACGGTAGCTAATACCGCATGACCTC

GAAAGAGCAGAGTGGGGGATCTTCGGACCTCACGCCATCGGATGTGCCCA

GATGGGATTAGCTAGTAGGTGAGGTAATGGCTCGCCTAGGCGACGATCCC

TAGCTGGTCTGAGAGGATGACCAGCCACACTGGAACTGAGACACGGTCCA

GACTCCTACGGGAGGCAGCAGTGGGGAATATTGGACAATGGGCGCAAGCC

TGATGCAGCCATGCCGCGTGTGTGAAGAAGGCCTTAGGGTTGTAAAGCAC

TTTCAGCGAGGAGGAAGGCATCACACTTAATACGTGTGGTGATTGACGTT

ACTCGCAGAAGAAGCACCGGCTAACTCCGTGCCAGCAGCCGCGGTAATAC

GGAGGGTGCAAGCGTTAATCGGAATTACTGGGCGTAAAGCGCACGCAGGC

GGTTTGTTAAGTCAGATGTGAAATCCCCGCGCTTAACGTGGGAACTGCAT

TTGAAACTGGCAAGCTAGAGTCTTGTAGAGGGGGGTAGAATTCCAGGTGT

AGCGGTGAAATGCGTAGAGATCTGGAGGAATACCGGTGGCGAAGGCGGCC

CCCTGGACAAAGACTGACGCTCAGGTGCGAAAGCGTGGGGAGCAAACAGG

ATTAGATACCCTGGTAGTCCACGCTGTAAACGATGTCGACTTGGAGGTTG

TGCCCTTGAGGCGTGGCTTCCGGAGCTAACGCGTTAAGTCGACCGCCTGG

GGAGTACGGCCGCAGGTTAAAACTCAAATGAATTGACCGGGGCCCGCACA

AGCGGTGGAGCATGTGGTTTAATTCGATGCAACGCGAATAACCTTACCTA

CTCTTGACATCCACGTATTCGCAGAAATGGCTTAGTGCCTTACGTAACCG

TGAGACAGGTGCTGCATTGGCTGTCGTCAGCTCGTGTGTGAAATGTTGGG

TTAAGTCCCGCACCGAGCGCCTATCCCTTATCCTTTTGTTGCCAGCACGG

TAATGGTGGGTACTCAAAGGACACTGTCACGGTGATAAACCGGAGGGAGG

GTGGAATGAACGTCAAGTCATCCCTGGACCGTTACGAAGTTAGGCCTACA

CTACATGCCTACCATTGCCATATAGCGAATGGACAAGCCGAAGCCTCTCG

CTCGAGAAGGCCTAGGCTGCGAACCCTCTCACTCAG

>SS1

ACGTTTGGTAACTCTTACCATGCAAGTCGAGCCGTCGCCTTGAGGAGCAT

GATACTTCTGCCAAGCTAATGGGAAAAAGGGGTGTCTGTCTGGCAAGGTG

CGAAGGCCAGCTGTTTAACAGCGGAGAACGAATGCGAAAACCGCATACGC

CCTACGCTGGTATCCCCCGCAACTTGGGACCTTGCGCGACTTTACACGCC

CGGGCGCCGTTCGCTTGTTAAAGAAGCAAGGGGTCTCCCAGGCGACGCTC

CCTATGTATGCTGAAAGGATGCCCAGCGACTTCGAAACTAACCCTTGTCC

AAATCTTGAAAGCCGTGGGGAATATTGGACTATGGGCCAAAGCCTGATCC

CGCCATGCCGCGAGGGTGCAGAAGGTCTTCGGATCGTCAAGCACTTTAAT

TTGTTAGGAATGGCATTTTGCTTATTTCAATGCTGCCTTTACTTTTTTTT

TCCCAATAAGCATCGTGGTGGCATCTGTGCCACCAGCCCCCGTAATACAG

TTGGTGGCAGCGTTAAATCGG

>SS2

AGGGGCCTCGGACCAAAAAGAAGAGGGGGCCAATCTAGACTGGCAGAGGG

GGACAACGTTCCTTTAGGGAACGCTAATACCGCACACGTCCTACCGCAGA

ACCCAAGGGACCTTCGGGCCGTGCGCTAGAAATGAACCTACGCCGAATGA

ACTAGCTGGTGGGTGAGGGGCTCACCCAGCGGAGGAACCATACCTGGCCT

GACAGGAAGATCGATCACCCTGGAACTGAGACACGGTCCAGACTCATTCC

GTTGGCAGGCATGGTTAATAAAGAATAAAGGACGAAAGCCTGACCCATGC

ATGCCTCCTGCGTGAATAAGGACTTCCGATTGTAAAGCAATTAACGTTGG

GAAGAAGGGAAGTAAGAGAATACCTTGCTGTCTGACCTTACTTTTATAAT

AAACACTGGTTAACTCTGCCCTGCAGCCGCGCTAATACAGAGGGAGGGTG

CGTTAATCAGAAGTAATGCGCGGAAATCGCGCGTACGCAGGTTTGTTGTT

TGAATGATAAAGACCCGGGCTCCTCTGGTGAGGCATTCATTACTAGCGCC

TAGACAAGGAACG

>SS3

CGGATGGGGCAATATCTCGTGCGTGTCGAGCGGTAGCCTAAGAGAGCCAT

TACCGCACCTCGTAGGGCGTCCGACCTGGTCAATCTGATAACCTGAGGCC

CGAAGGTCCGATTCTTTGGGAAACGAACGATGATCGGAATCTGCGACCGT

TTCCAGAAGAGATCCCCCTTCATCAGCTCTTGCCCCAGACATTACTCACC

CGTCCGCCGCACGACACGGAGAGCAAGCTCACCTGTGCGAACGATCCCTA

GCTGGTCGGAAAGCATGGCCGGCCGCGCTGGAACTGAGCCATGATCAATC

TCTG

>SS4

ACCTTTTGATATCCTCTAGTGCGTGTCGAGCGGTAGCCTTGGACTTGATT

ACGTCGGAGCTAGCTAATCCGACCTATAATGATCTAAAATCTGCCTAGCC

GAAGGTCCTAACCTTTCTCAAGGAGCGCGAATACCGCATACGTCCTAGTT

TCCGGACGTTATCATCTTCGAACCTCACGCTATTAAATGATTACTCACCG

GATTACCTAGTTGGTGAGGTAATGGCTCCCCGAGGCGACGATCCGTAACT

GTGCTGAAAGGATGATCAGTCACACTGATCCTGACCCAGGATCAACTATT

GTCGCTCAGGTTGCATGGGGGAATATTGCACAATGCGCTAAATCCTGACC

CTGATCCAACTATTATGTCGTTCTTTTTG

>SS5

TGGTTTGTGGGGGCCTAACCATGCAATTTGAGCGGTAGCCAGGGGGCGTG

CTCTCGGATGAAGAGGGGAGGACGGGAGAGGGATGTCTGGGAAACTGCCT

GAGGGGGGGGGATAATTACTGGAAACGGTAGCTTATACCGCATAACGTCG

TAGACCAAAGAGGGGGACCTTCGGGCCTCTTGCCATCAGATGTGCCCAGA

TGGGATTAGCTAGTAGGTGGATAATGGCTCACCTAGGCGACGATCCCTAG

CTGGTCTGAAAGGATGACCAGCCACACTGGAACTGACCATGATCCAAACT

CTACGGGAGGCAGCAGTGGGGAATATTGCACAATGGGCGCAAGCCTGATG

CAGGCATGCCGCGTGTATGAAGAAGGCCTTCTGGTTGTAAAGTACTTTCA

GCGAGGAGGAAGGATTGTGGTTAATAACCGCAGTGATTGACGTTACTCGC

AGAAGAAGCACCGGCTAACTCCGTGCCAGCAGCCGCGGTAATACGGAGGG

TGCAAGCGTTAATCGTATTACTGGCGTAGCCGCACGCAGGCGGTCTGTCA

GTCGGATGTGAAATCCCCGGGCTCATCCTGGGAGCTGCATTCGAACTGGA

GGCAAGAGCTTGTAGAGGGGGTAGAATTCCTGGTGTAGCGATGAACTGCG

TAGAGATCTGGAGAATACCGGGGCGAAGGCGGCCCCCTGTAAAAGACTGA

CGCTCAGTGCAAATGTGAGAGCAAACAGATTAATACCTGGTATCCACGCC

TAACGTGCTATTGGAGGTGTTCCTTGAGATGGTTCCGAACTAACGCGTAA

GGCGACCGCTGGGGAGACGCCGCAGTTAAACTCAAGTATTGCGGGGCCCG

CACAGCGGTGAGTGTTTTATTCACGCACGAAAAACCTTACCACTCTGACT

CTAAAATTACAAATTTTTGGGCTCAGAACTCAGAAAAGGGCGATGTCGCG

CCGTCGGGTGGAATGGTGGATACTCCCGCAGAGCTCTCTCTTATCATTGT

TGCACGGTCGTCGGAATCAGAAACTGCCGGATAACCTGAGAAGGGGATGA

CTAGATTA

>SS6

AAACCCCCAAAAAAAGAAGAGCAAGCCGGCAGGGGATTCCATGGTGGGAC

GGGCGCCCCCGAACAAGGCCCGGGAACGTATTCACCGTGGATTCTGATCC

ACGATTTCTAGCGATTCCGACTTCACGGAGTCGAGTTGCAGACTCCGATC

CGGACTACCACATACTTTGTGAGGTGCGGTGGGTCTCGCGAGGTCGCTTC

TCTTTGTATATGCCATTGTAGCACGTGTGTAGCCCTACTCGTAAGGGCCA

TGATGACTTGGCGTCATCCCCCCCTTCCTCCGGTTATCACTGGGATTCTC

CTTTGAGTTCCCGGCCGAATCTTTGGTTACAAGGGTACGGGTTGGGCTTG

TTG

>SS7

CCTTTGGGTACCTTTGTTAGGCCTTTCACCCCAGTCATGAATCACAAAGT

GGTAAGCGCCCTCCCGAAGGTTAAGCTAGAACATCTTTTGCACCCACTCC

CATGGTGTGACGGGCGGTGTGTACAAGGCCCGGGAACGTATTCACCGTAG

CATTCTGATCTACGATTACTAGCGATTCCGACTTCACGGAGTCGAGTTGC

AGACTCCGATCCGGACTACGACATACTTTGTGAGGTCCGCTTGCTCTCGC

GAGGTCGCTTCTCTTTGTATATGCCATTGTAGCACGTGTGTAGCCCTACT

CGTAAGGGCCATGATGACTTGACGTCATCCCCACCTTCCTCCAGTTTATC

ACTGGCAGTCTCCTTTGAGTTCCCGGCCGAACCGCTGGCAACAAAGGATA

AGGGTTGCGCTCGTTGCGGGACTTAACCCAACATTTCACAACACGAGCTG

ACGACAGCCATGCAGCACCTGTCTCAAAGTTCCCGAAGGCACCAAAGCAT

CTCTGCTAAGTTCTCTGGATGTCAAGAGTAGGTAAGGTTCTTCGCGTTGC

ATCCAATTAAACCACATGGCTCCACCGCTTGTGCGGGCCCCCGTCAATTC

ATTTGAGTTTTAACCTTGCGGCCGTACTCCCCAGGCGGGCGACTTAACGC

GTTAGCTCCGGAAGCCACTCCTCCAGGGAACAACCTCCAAGTCGACATCG

TTTACGGCGTGGACTACCAGGGTATCTAATCCTGGTTGCTCCCCCACGCT

TTCGCACCTGAGCGTCAGTCTTTGTCCAGGGGGCGCCTTCGCCACCGGTA

GTCCTCCCAGATCTCTACGCATTTCACCGCTACACCTGGGATTCTACCCC

CCTCTACCAGACTCTAGCCTGCCAGTTTCTGATGCAGTTCCCAGGTTGGA

GCCCGGGGATTTCACATACCGACTTGACAGACCGCCTGCGTGCGGCTTTA

CGCCCCAGTAATTTCCGATTAAACGGCTTGCACCCCTCCGGTATTACCGC

CGGCTGCTGGGCACGGAAGTTAGGCCGGTGGCCTTCTTCTGCGGAGTAAC

CGTCAATCACCTAGTCGATTATTACGCACATGCCCTTCCTCCTCGCTTGA

AAAGTACTTTACCAACCCGGAAGGGCCTTCTTCATACTCGCGGAATGGCT

GCATCATGCCTTGCCCCAATGTGCCAAAATTCCCACCTGGCTTGCTCTCC

>SS8

CCTTGGTACGTTGGTTAGAATTTCCCCAGTCATGAATCGCAAAGGGGAAG

CGGCCTCCCGAAGGTTAAGATAGAAGGGGGAGGGGAACCCACTCCCATGG

GGGGAGGGGGCCGTAAGAGGCCCGGGAACGTATTCACCGGGCATTCTGAT

CTACTATTACTAGCGATTCCGACTTCACGGAGTCGAGTTGCAGACTGCGA

TCCGGACTACGACATACTTTGTGAGGTCCGCTTGCTCTCGCGAGGTGGCT

TCCCTTTGTATATGCCATTGTAGCACGTGTGTAGCCCTACTCGTAAGGGC

CATGATGACTTGACGTCATCCCCACCTTCCTCCGGTTTATCACTGGCAGT

CTCCTTTGAGTGCCCGGCCGAACCGGTGGGAAAGAAGGATAAGGGTTGCG

CTCCGTGCGGGACTTAACCCAACGTTTCAAAACACCAGCTTAACAAAGCC

CTGCAACAACTGGCTCAAAATTCCCGAAGGGACCCAATCATCTCCGCGAA

ATTCTCCGGATGGCAAAAGTAGGGAAGGTTCTTCGCGTTTCGTCGAAATA

AACCACATGCTCCACCGCTTGGGCGGGCCCCCGTCCATTTATTTGAATTT

TAACCTTGGCGCCGTACTCCCCCAGGGGGCGACTTACGGGTTAACTTCGG

AAACTACTCCTCAAGGGAACAACCTCCATATCTACCTTCGTTACGGGGTG

GAATAACCAGGGGAACTTAATCCTGGTTGCTCCCCAAGCTTTCACACCTG

AGTGGTATTCTTATCCAGGGGGCGCCCTCCCCCCCCGGGATTCCCTCTCA

TCTCGACGCATTTCTCCGCTAACACTGGAATTCTATCCGCCCTCAACAGA

ATCTCAACCTGCCCGGTTCTAGGTGGACGTCCCCGGTTAAGCCCGGGGAT

TTTCACATCTCGAATTGGACAAACCGCCCTGACTAGCGCATTAAGCGCCC

AGTAAATTCTGATTAAAGGCTTGCACCCTCCTGTATTACGCGGGCTCGCT

GGATCCAGAAATTTGGCCGGTGGCT

>SS9

GCGTGGCGGGACGCCTTCACGTGCGAGTCGAGCGGTAGACTAGAGAGCCT

TTACCCCACCTACTAACCAACGGACGGGGACTAACGGAGGCGTCAGGCTG

GTGGAGGCGACATTGCTGGATCGGTCGTGCGACCGATGACGTCTTCCAGG

AGAGTCCGGACCTTCGGGCCTCTCCCCAGCCAATACGCCCCCATGGGATT

ATCGAGAACGAGAGAGAAAGCCTCCCCGAGCTGACGATCGATTTGTAGGA

TGAAAGCATGCCACGCCCCTCTGAAACTAACCCTGGTCCAACTCTTGG

>SS10

AAAGCCGCGGGCCTTACAATGCAAGTCCGAGCGGTAGAGAGAAGCTTGCT

TCTCTTGAGAGCGGCGGACGGGTGAGAAAAGGCTAGGAATCTGCCTGGCA

GTGGGGGATAACGCTCGTTAACGGACGCTAATACCGCATACGTCCTACGG

GAGAAAGCAGGGGACCTTCGGGCCTTGCGCTATCAGATGAGCCTAGGTCG

GATTAGCTAGTTGGTGAGGTAATGGCTCACCAAGGCGACGATCCGTAACT

GGTCTGAGAGGATGATCAGTCACACTGGAACTGAGACACGGTCCAGACTC

CTACAGGAGGCAGCAGTGGGGAATATTGGACAATGGGCGAAAGCCTGATC

CAGCCATGCCGCGTGTGTGAAGAAGGTCTTCGGATTGTAAAGCACTTTAA

GTTGGGAGGAAGGGTTGAAACCTAATACGTTGCAATCTTGACGTTTTTTA

TAGAATAAGCACCGGCTAACTCTGTGCCAGCAGCCGCGGTAATACAGAGG

GTGCAAGCGTTAATCGGAATTACTGGGCGTAAAGCGCGCGTAGGTGGTTC

GTTAAGTTGGT

>SS11

CCCAATTTGGAAAGGAAAAAGCGGTAACAGGGCTTAGGTGCTGACTAGTG

GCGACGGGTGAGTAACAAAACGGACCCTGCCTAAAGTGGGGGATACTACT

CCCTATAGAGCTAATACCGCATGAGATCTATGGATGAAAGCAGGGGACCT

TCGGGCCTTGTGCTACTACACCGGCTGATGGCACATTATGTAGTTGGTGG

GGTAAAGGGTTACCGAGCCTGCGATCTGTCGATGGTCTGAGAGGACGACC

AGCCACACTGGGACAGAGACACGGACCCAACTCCTACAGGAGGCAACAAT

GGGGAATTTTGGACAATGGGCGAAAGCCTGATCCCCCAATGCCCCCTGCA

GGATGAAGGCCCTCGGGTCGTAAGCTGCTTTTGTACTGTCCGAAAAGCGT

GGGGCTAATACCCCCGGGTCATGACGGAAATATAATAATAACCACCGACT

AACTACGTGTCACCATCCGCGCTAATACCTAGGGTGCGAGCGTGAATCCG

AATTACTGGGCGTAAAGCCTGTGCCGCGGTTTTGTAACACTTGGTGAAAT

CCCCGCTCCCCACTGTGATCTGCCATTGTGATGGAAACTAAAGAGCAGAC

ACGGGGATAGGATTCCACGTGTACCGTGTACTGCGTATATGTGCACAGAA

ACACCGATGGCC

>SS12

TGGGAGGAGGGGCGGGGCTTCGGCCGGAGACATTCCCATGGGGGGGACGG

TCGGCCAAAAAAGGGACCCGGGAACGTTTTCCCCCGCGACTTCCGGATCC

CTTTTTTCTAGCGATTCCGACTTTCTGGATTTTAAGTTGCAGACTCCTAC

CCGACTTCGACATGGCTTTATGGGGGGTGTGTGTTGTGCTTCCGAGGTTC

GCTTCTCTTTTGTATCTGCCCTTGGTATGACGTGGGTACCCCCACTCTTA

AGGGGCATGAGGACTTGACGTCATCCCCACCTTCCTCCGGTTTATCACCG

GCAGTCCCCTTTGAGTTCTCACCTTTTTTGTGCTGGCTACTGGCAATGAA

GGCTGCCGTCGCGGGACGTATTTTCCCA

>SS13

ACCGAGAATTTGATTCGACTTCACCCAGTCATGATCACAAAAGTGGTAAG

CGCCCTCCCGAAGGTTAAGCTACAAACAATGGGGTTGCAACCCACTCCCA

TGGTGTGACGGGCGGCGTGTACAAGGCCCGGGAACGTATTCACCGTAGCA

TTCTGATCTACGATTACTAGCGATTCCGACTTCATGGAGTCGAGTTGCAG

ACTCCAATCCGGACTACGACATACTTTATGAGGTCCGCTTGGTCTCGCGA

GTTCGCTTCTCTTTGTATATGCCATTGTAGCACGTGTGTAGCCCTACTCG

TAAGGGCCATGATGACTTGACGTCATCCCCACCTTCCTCCGGTTTATCAC

CGGCAGTCTCCTTTGAATTCCCACCATTACGTGCTGGCAACAAAGGATAA

GGGTTGGGCTCGTTGCGGGACTTAACCCAACATTTCACAACACGAGCTGA

CGACAGCCATGCAGCACCTGTCTCACGGTTCCCGAAGGCACTAAGCCATC

TCTGGCGAATTCCGTGGATGTCAAGAATAGGTAAGGTTCTTCGCGTTGCA

TCGAATTAAACCACATGCTCCACCGCTTGGGCGGGCCCCCGTCAATTCAT

TTGAGTTTTAACCTTGCGGCCGTACTCCCCAGGCGGTCGACTTAACGCGT

TAGCTCCGGAAGCCACGCCTCAAGGGCACAACCTCCAAGTCGACATCGTT

TACAGCGTGGACTACCACGGTATCTAATCCTGTGTGCTCCCCACGCTTTC

GCACCTGAGCGTCAGTCTTTGTCCAGGGGGCCGCCTTCTCCACCGGTATT

CCTCACATCTCTACGCATTTCACCGCTACACCTGGAATTCTACCCCCCTC

TACAGACTCTAGCTTGCCAGTTTCAAATGCAGTTCCCACGTTAAGCGCGC

GGAGTTTCACATCTGACTTATCAACCGCCTGCGATGCGCTTTACGCCCAG

TAAATACTGATTAACGCTTGCACCCCTCCTGTATATCCGCCGGCTGCTGG

CCACGCAGTTAGTCACGGTGCTTCTTTCTGCCAAGTACGTCAATCCACAC

ACGTATTAAGTATCGATTGCTTCCTCCCTCCCTGAAAATGGCTTTTACTA

CCCTAGACGTTCTTTCACACACCGCGGCAATGGCTGCATCCAGCCTGGCG

TGCCCACTGTGTGCCAAATTCCCCCATTGCTTGCCTTCCCGTAGATTTGA

CTAGGCTTCCAATTCCAAGGTGTGGCTAATCTCTCTTG

>SS14

ACCTGGCTTCTGTCCTATCGACCGCTCGAGCGGTCGGGAGGTGAGCTGTA

CTTTGCTGCCGAGCGATCCCCGGGGGAGTATCCGATGGAAACAGGCCGGA

GGAGGGGGACTTTACTGGAAACGGAGCTAATACCGCATGACCTCGAAAGA

GCAAAGAGGGGGATCTTCGGACCTCACGCCATCGGATGTGCCCAGATGGG

ATTAGCTAGTAGGTGAGGTAATGGCTCACCTAGGCGACGATCCCTAGCTG

GGCTGAGAGGATGACCGGCCACACTGGAACTGACCTGATCAACTCTCAAC

GGGAGGCAGCAGTGGGGAATATTGCACATGGGCGCAAGCCTGATGCACCC

ATGCCGCGTGTGTGAAGAAAGCCTTATGGTTGTAAAGCACTTTCAGCGAG

GAGGAAGGCATCATACTTAATACGTGTGGTGATTGATTTTACTCGCACAA

GATGCACCGGGTAACTCCGTGCCAGCAGCCGCGGTAATACGGAGGGTGCA

AGCGTTAATCGGAATTACTGGGCGTAAAGCGCACGCAGGCGGTTTGTTAA

GTCAGATGTGAAATCCCCGCGCTTAACGTGGGAACTGCATTTGAAACTGT

CAAGCTAGAGTCTTGTAGAGGGGGGGTAGAATTCCAGGTGTAGCGGCGAA

CTGCGTATAGATCTGGAGGAATACCGGTGGCGAAGGCGCCCCCTGGACAA

AGACTGACGCT

>SS15

TTGGGGGGGCCTAACCATGGCAAGTCGAGCGGTAGCACAAAGAGGGTTGC

TTCCTGAAGCGAGGGACGGGTGCGTAAAGAATAGGAGTCTGACTGGGAGT

GGGGGAGGGGGATCCCTTTTGAACGCTAGTACCGAATACGTATTACGGCA

GAAAACAAGGGGGGGTCCTTCTGGGCCCTAGCGCATGAATATGCGCCGAT

ATGGTTAGATCTTGAGGGAGAGGCTCACTCACGCGACGGACAATATCTGG

CCGGGGAGAGAGATCATGCACACTCACTCTGACTCAGAGACAGGACCCAT

ACCCCTAGGAGGGGTGGGGAATATTGATTGATCACTGTGGGCCTGATCCT

GACATGCCGCTCGCGTGTATAAGGACATCGGATTGTATATGACTTACAGT

TTCCGAGGAGGAAAGCGACCTAGTATAAAACACCATCGACTTAGGTTATT

CTCACAAAACAGGCGGGTATGTCCGCGTCACCCGCGGCGCACAAAGGGGG

GGGGCGTTAATCTTCTCACTTTACTGAGTAAAACGTCGCTGGTTTGCTGT

GTTGTATGAGGTGACATCCCCCGCCTCTCCCTGACTGCACTCCACACTGA

CTGACTACACTATAGTCGAGGATGGGGGAATTTCCTGTGGGGTGGAGCAA

TGATTCGCTTAGAGATGGAGCACTATCGCTGTAGAAAACCCCCTCCCCTA

CAACTAATCATGCTCTCCTGCGACAAGGCGAGCAGACAACACTAGATTAC

ATGCTGGGACACTCCGCTGTATATGATATCTACCCGTTGGAAGCGCTCAT

CTTGAGAGGGTGCTGCAGATCACATGAGTTGACCCCCCCCGCGGAGGACA

CCCGCCGCGCTAGTATTACTCTGATTTGATTGGGGGCGCGCCCCACACGC

GGGGGAGGGTGGTGTATTTCTATTGCACGCGAAGAAAATTACCTTGCTCC

TTCGTTTTCCTATGCAAAATTCTTTAACAAAATATTGTTTGCGTCCCTTG

AAAGAATACTGACAGAGGCTGGCTTCGACTGGTCGTTCACATCCGCTGCC

GTG

>SS16

ACTTGGGGGGGGGCTTCCCATGCGGTCGAGCGGTCGACAGAGAGAGCGTC

TCCTGACACGAAGGTCGGCCGCCAAGGCATATCTGACTGCCTGGGAGAGG

GGGAGGCGACTTTGGCGAACGCGGTACCGCGTAATTCCCACCGCAAAACG

CAAGTGAGCTTCCTTCCTTGCCCTAGCAAATGAGCCTGGGTACCATTCCG

CCATTGGTGAGGGAGGGGCTCACCCAGGCGGGATCCGGATCTGGTCGGAG

AGGAAGATCAGTCACACTGCTCTGATGAGAGTCCAGACTAATCTGGAAGG

CAGCAGTGGGGAATATTGGACAATGGGCGAAAGCCTGATCCAGCCATGCC

GCGTGTGTGAAGAAGGTCTTCGGATTGTAAAGCACTTTAAGTTGGGAGGA

AGGGCAGTTACCTAATACATGATTGTTTTGACGTTATTAACAGAATAAGC

ACCGGCTAACTCTGTGCCAGCAGCCGCGGCATACAGAGGGTGCAAGCGTT

AATCGGAATTACTGGGCGGAGAGCGCGCGTAGGTGGTTTGTTAACTTGGA

TGTGAAATCCCCGGGCTCAACCCTGGGAACTGCTTTCTAAACTGACTGAC

TAGAATATGGTAGAGGGAGGGGGGAATTTTCCTGTGTAGCGGTGAGTGCG

TAGATATAGGAAGGAACACCACTGGCGGAAGGAGACCACCCGGACTACTA

A

>SS17

TTTTTGCAATTTCCAAACGCGAAGAACAATAGTTTGCTCTTCCCTTCTGC

GGCGGGCGGGTGAGAAAAAAAAGGCTCTGTCTGGTAGTGGGAGACACGTT

CAAAAGGAACGGTAACCCCGTATACTTTTTACGGGAAAACGGGGGGATCT

TCGGGCTTTGCTCTATCAGATGATCCTAGGTCGTATTGTCTACCTGGTGG

GGTAAGGGCTCCCCCCCGGGGGGGAGGCGTCTCTGGTCTGAGAGGAAGAA

CACTCACTCTGGCTCTGACACACCCTCCAGACTCCTACGGGAGGCAGCTG

GGGGAATTTTTGCACTGTGGGTGAAAGCCTGCCCCCCCCGTCCCGCGCGC

GTGAAAAACCCCTCCCGATTGTACAGCACTTTTACTTGGGAGGAAGGCTG

GTGCTCTCATACTTTTTTGTTGTCACGTTTTATTTTAATATACGCAGCGG

CTGTCTCTGTGCCCCCACCGCTGCATACAGATTGTGTTTCGTTTTTTATC

TTTTTTTATTGCG

>SS18

GGGTGGGGGACGTTGGTTCGAATTTCCCTCAGTCATGAATCAAAAGTGGT

AAGCGCCCTCCCGAAGGTTAAGCTAAAACAGCTTTTGCAACCCACTCCCA

TGGTGTGACGGGCGGTGTGTACAAGGCCCGGGAACGTATTCACCGTAGCA

TTCTGATCTACGATTACTAGCGATTCCGACTTCACGGAGTCGAGTTGCAG

ACTCCGATCCGGACTACGACATACTTTGTGAGGTCCGCTTGCTCTCGCGA

GGTCGCTTCTCTTTGTATATGCCATTGTAGCACGTGTGTAGCCCTACTCG

TAAGGGCCATGATGACTTGACGTCATCCCCACCTTCCTCCAGTTTATCAC

TGGCAGTCTCCTTTGAGTTCCCGGCCGAACCGCTGGCAACAAAGGATAAG

GGTTGCGCTCGTTGCGGGACTTAACCCAACATTTCACAACACTATCTGAC

GACAGCCATGCAGCACCTGTCTCAGAGTTCCCGAAGGCACCAAAGCATCT

CTGCTAAATTCTCTGGATGTCAAGAGTAGGTAAGGTTCTTCGCGTTGCAT

CGAATTAAACCACATGCTCCACCGCTTGTGCGGGCCCCCGTCAATTCATT

TGAGTTTTAACCTTGCGGCCGTACTCCCCAGGCGGTCGACTTAACGCGTT

AGCTCCGGAAGCCACTCCTCAAGGGAACAACCTCCAAGTCGACATCGTTT

ACGGCGTGGACTACCAGGGTATCTAATCCTGTTTGCTCCCCACGCTTTCG

CACCTGAGCGTCAGTCTTTGTCCAGGGGGGCGCCTTCGCCACCGGTATTC

CTCCAGATCTCTACGCATTTCACCGCTACACCTGGGAATTCTACCCCCCT

CTACAAGACTCTAGCCTGCCAGTTTCGAATGCAGTTCCCAGGTTGAGCCC

CGGGGATTTCACATCTCGACTTGACAGACCCGCCCTGCGTGGCGCTTTAC

GCCCAAGTAATTTCCCGATAACGCTTTGGACCCTCCGTTATTACCGCGGC

CTGCTGGCACCGGAGTTAGCCGGTGCTTCTTTCTGCGAGTAACGTCAATC

ACTGGGGTTATTAACCACAAATGCCCTTCCTCCTCGCTGAAAGGTACTTA

CAGCCGGAAGGCCTCTTCATAACCCGCGGCATGGCTTGCATCAGGCTGCG

CCCATTGTGCAATATTCCCATTGGCGTGCCTCCGTAGAGATTTGGAACCG

TGTCTCTCAGTTCCAGTGGTGGGCGCGTGGTACAT

>SS19

TCCCTGAAAATCCCCCGGCCCGGGGGGAAGCGCCCTCCTTTGAGGTTAGG

TTAGAAACGGGCGGGGGGAGACCCGTTCCATGGTGTGACGGGCGCTGCCG

TAAAAGGACCCGGGAACGTATTCACCGTGACATTCTGATCCCGATTACTA

GCGATTCCGACTTCACGCAGTCGAGTTGCAGACTGCGATCCGGACTACGA

CTGGTTTTATGGGATTAGCTCCCCCTCGCGGGTCGGCAACCCTTTGTACC

AGCCATTGTATGACGTGTGTAGCCCCACCTATAAGGGCCATGAGGACTTG

ACGTCATCCCCACCTTCCTCCGGTTTGTCACCGGCAGTCCCATTAGAGTG

CTCAACTGAATGTATGAACTAATGGGAAGGGTTGCGCTCTTTGTGGGACT

TATCCCAACATCTCACGACACGATTAGATAACAGCCATGCGGCACCGGTG

TGCAGGTTCTCTTTAAAGCACCAAACCATCTCTGGTAAGTTCCTGCCATG

TCAAAAGTGGGTAAGGTTTTTCGCGTTGCATCCAATTAAACCACATCATC

CACCGCTTGTGCGGGTCCCCGTCAATTCCTTTGAGGTTCAATCTTGGGGC

CGTACTCCTCAGGCGGGCAACTTCACTCTTTAGCTTCTTTACTGACTCAT

TTAAGACTCAACAACCAATTGACATCTT

>SS20

CGGCATTGGGGACTTGGTTTGGACTTCCCCCAGTCACGAATCCCGCCGTG

GTAAGCGCCCTCCTTGAGGTTAGGCTAAAAAAAGGGCGAGACCCGCTCCC

AGGGGGGGACGGGGGGTGTGTACAAGACCCGGGAACGTATTCACCGTGAC

ATTCTGATCCACGATTACTAGCGATTCCGACTTCACGCAGTCGAGTTGCA

GACTGCGATCCGGACTACGACTGGCTTTATGGGATTAGCTCCCCCTCGCG

GGTTGGCAACCCTTTGTACCAGCCATTGTATGACGTGTGTAGCCCCACCT

ATAAGGGCCATGAGGACTTGACGTCATCCCCACCTTCCTCCGGTTTGTCA

CCGGCAGTCCCATTAGAGTGCTCAGCTGAATGTAGCAACTAATGGGAAGG

GTTGCGCTCGTTGCGGGACTTAACCCAACATCTCACGACACGAGCTGACA

ACAGCCATGCAGCACCTGTGGGGAGGTTCTCTTTCAAGCACCAAACCATC

TCTGGTAAGTTCCTGCCATGTCAAAGGTGGGTAAGGTTTTTCGCGTTGCA

TCGAATTAAACCACATCATCCACCGCTTGTGCGGGTCCCCGTCAATTCCT

TTGAGTTTCAACCTTGCGGCCGTACTCCCCAGGCGGGTCAACTTCACGCG

TTAGCTTCGTTACTGAGTCATTTAAGAACCAACAACCAGTTGACATCGTT

TTAGGGCGTGGACTACCAGGGTATCTAATCCTGTTTGCTCCCCACGCTTT

CGTGCATGAACGTCAGTGCAGGCCCAGGGGATTGCCTTCGCCATCGGGGG

TTCCTCCGCATATCTACGCATTTCACTGCTACACCGCGGAATTCCATCCC

CCTCTGCCGGACTCTAGCTTTGCAGTCACAGTGGCAGTTCCCAGGTTGAG

CCCGGGGATTTCACCACTGTCTTACAAAAACGGCCTGCGCACGGCTTTAC

GCCCAGT

>SS21

GGCCCTGTGGTTCCTTTGTTTAGACTTCACCCAGTCATGAATCACACCGT

GGTAACCGTCCTCCCGAAGGTTAGACTAGCTACAAACTGGTGCAACCCAC

TCCCATGGTGTGACGGGCGGTGTGTACAAGGCCCGGGAACGTATTCACCG

CGACATTCTGATTCGCGATTACTAGCGATTCCGACTTCACGCAGTCGAGT

TGCAGACTGCGATCCGGACTACGATCGGTTTTATGGGATTAGCTCCACCT

CGCGGCTTGGCAACCCTCTGTACCGACCATTGTAGCACGTGTGTAGCCCA

GGCCGTAAGGGCCATGATGACTTGACGTCATCCCCACCTTCCTCCGGTTT

GTCACCGGCAGTCTCCTTAGAGTGCCCACCATTACGTGCTGGTAACTAAG

GACAAGGGTTGCGCTCGTTACGGGACTTAACCCAACATCTCACGACACGA

GTTGACAACAGCCATGCAGCACCTGTCTCAATGTTCCCAAAGGCACCAAT

CTATCTCTAGAAAGTTCATTGGATGTCAAGGCCTGGTAAGGTTCTTCGCG

TTGCTTCAAATTAAACCACATGCTCCACCGCTTGGGCGGGCCCCCGTCAA

TTCATTTGAGTTTTAACCTTGCGGCCGTACTCCCCAGGCGGTCAACTTAA

TGCGTTAGCTGCGCCACTAAAAGCTCAAGGCTTCCAACGGCTAGTTGACA

TCGTTTACGGCGTGAACTACCAGGGTATCTAATCCTGTTTGCTCCCCACG

CTTTCGCACCTCAGTGTCAGTATTAGTCCAGGTGGTCCCCTTCGCCACTG

GTGTTCCTTCCTATATCTACGCATTTCACCGCTACACAGGAAATTCCACC

ACCCCTCTACCATACTCTAGCTCAGTCATTTTTGAATGCAGTTCCCAGGT

TAGACCCCGGGGTATTTCACATCCAATTTAACAAACCACCTGACGCGCGG

CTTTACGCCCAGAAATTCCGAATTAACCGCTTGCACCCTCTCTGTATTAC

CGGCGGCCTGCTGGACACAGATTAGCGCGGTGTTTATTTCTTGTCGGAAC

GTCAAAACAATCGACGTAATAACGTAACTTGCCCCTTTCTCTCCCACTTA

AAGATGCTTACAACTCGAAAAGAACCTTCTTCCACACCCCGGCCATGGCC

TCGAAATCAGGGCTTCGCCAATTGCATTATTCCCACATTGCTGCCTCCGG

TAAGAAGTTCGGAACCGGTCCCAAGTTCCAGTGTGACGTGAACATCCCTC

>SS22

GGGGTCCACTTTGGTTAGAATTTTCCCCCCAGTCATGAATCACACCGTGG

TAAGCGCCCTCCCGAAGGTTAGACTAGCAAAACGGGCGCGACCCACTCCA

TGGGGGGACGGGCGGTGTGTACAAGGCCCGGGAACGTATTCACCGCGACA

TTCTGATTCGCGATTACTAGCGATTCCGACTTCACGCAGTCGAGTTGCAG

ACTGCGATCCGGACTACGATCGGTTTTGTGAGATTAGGTCCGCCTCGCGG

CTTGGCAACCCTCTGTACCAACCATTGTATCACGTGTGTAGCCCCAGCCA

TAAGGGCCATGATGACTTGACGTCATCCCCACCTTCCTCCGGTTTGTCAC

CGGCAGTCTCCTTAGAGTGCCCAACTGAATGTGCTGGCAACTAGGGACGG

GGGTGGTCCTCGTTAGGGTAATTACCCCATCATCTCACGACATTAGTTAA

TAACAGCCCTGCAGCCCGTGGGTCGAAGTTCCCGAAGGCACCAACCCTCT

TTTAGAAATTTCTGCGCAGGTAAAGGTGTGGAAGGGTTCTTCCCGTTGCT

TCAAATTAAACACCATGCCCCACCCCTGGGGGGGTCCCCCGCAATTCCAT

TTAAGTTAAAACTTGCCGGCGGAACCCCCAAGGGGTCACTTCAATGGGTT

ACTTGCGAACGACTCAGTTAGGATCCACCCGATATGTGACCGTTTAAGGG

CGGGAACCCGGAATCATTCTCTGTGCTTCCCCGCTCTCCCCCCTGCC

>SS23

ACGGGGGTCCTTGGTTACGATTTCCCCCGTCATGAATCAACAGTGGTGGC

GCCTCCCGAAGGTGAGACTAAAAAGGGCTCTTGCACCCTTCCCATGGGTG

AGGGCGGCTCGAAAAAGGCCCGGGAACGTATTCACCGGAGCATTCTGATC

TACGATTTCTAGCGATTCCGACTTCACGGAGTCGAGTTGCGGACTCCGAT

CCGGACTACGACCTACTTTGTGAGGTCCGGGTGCTCTCGCGAGGTCTCTT

CTCTCTGTATATGCCATTGTAGCAGGTGTGTAGCCCTACTCGTAAGGGCC

ATGATGACTTGACGTCATCCCCACCTTCCTCCAGTTTATCACCGGCAGTC

TCCTTTGAGTCCCCGGCCTAACCGCTGGGGAAAAAAGAAAAGGGGTGCCG

TCGGTGGGGGCTTTTTTTTTTTTTGTTTCAAG

>SS24

TGGGGGTACCTTGGGTACCAACTTCCCCCCAGTCTGAATCACAAAGGGTA

AGCGCCCTCCCGAAGGTTAAAAAGAACGGGAAGGTGGGGACCCATTCCAT

GGTGTGACGGGGGCGCGAACAAGGCCCGGGAACGTATTCACCGGAACATT

CTGATCTACTATTACTAGCGATTCCGACTTCACGGAGTCGAGTTGCAGAC

TCCGATCCGGACTACGACATACTTTATGAGGGGCGGTTGCTCTCGCGAGT

TGGCTTCTCTTTGTATATGCCATTGTAGCACGTGTGTAGCCCTACTCGTA

AGGGCCATGATGACTTGACGTCATCCCCACCTTCCTCCGGTTTATCACCG

GCAGTCTCCTTTGAGTTCCCACCATGACGTGCTGGCAACAAAGGATAAGG

GTTGCGCTCGTTGCGGGACTTAACCCAACATTTCACAACACGAGCTGACG

ACAGCCATGCAGCACCTGTCTCACAGTTCCCGAAGGCACCAATCCATCTC

TGGAAAGTTCTGTGCATGTCAAGAGTAGGGAAGGTTCTTCGCGTTGCATC

GAATTAAACCACATGCTCCACCGCTTGTGCGGGCCCCCGTCAATTCATTT

GAGTTTTAACCTTGCGGCCGTACTCCCCAGGCGGTCGACTTAATGCGTTA

GCTCCGGAAAGCCACGCCCTCAAGGGGCACAACCTCCAAAGTCGACATCG

TTTACGGCGTGGACTAGCAGGGTTATCTAAATCCTGTTTGCTCCCCACGC

TTTCGCACCTGAGCGGTCAGTCTATTGTCCAGGGGGGGCCGCCTTCGCCA

CTG

>SS25

CCCGGGTGGGTCCTTGTTTCAACATTCACCCCAGTCATGAATCACAAAGT

GGTAAGCGCCCTCCCGAAGGTTAAGCTACTACAACTTTTGCAACCCACTC

CCATGGTGTGACGGGCGGTGTGTACAAGGCCCGGGAACGTATTCACCGTA

GCATTCTGATCTACGATTACTAGCGATTCCGACTTCACGGAGTCGAGTTG

CAGACTCCGATCCGGACTACGACATACTTTGTGAGGTCCGCTTGCTCTCG

CGAGGTCGCTTCTCTTTGTATATGCCATTGTAGCACGTGTGTAGCCCTAC

TCGTAAGGGCCATGATGACTTGACGTCATCCCCACCTTCCTCCAGTTTAT

CACTGGCAGTCTCCTTTGAGTTCCCGGCCGAACCGCTGGCAACAAAGGAT

AAGGGTTGCGCTCGTTGCGGGACTTAACCTTACATTTCACAACACGAGTT

GACGACAGCCATGCAGCACCTGTCTCAGAGTTCCCGAAGGCACTAAAGCA

TCTCTGCTAAATTCTCTGGATGTCAAGAGTAGGTAAGGTTCTTCGCGTTG

CATCGAATTAAACCACATGCTCCACCGCTTGTGCGGGCCCCCGTCAATTC

ATTTGAGTTTTAACCTTGCGGCCGTACTCCCCAGGCGGTCGACTTAACGC

GTTAGCTCCGGAAGCCACTCCTCAAGGGAACAACCTCCAAGTCGACATCG

TTTACGGCGTGGACTACCAGGGTATCTAATCCTGTTTGCTCCCCACGCTT

TCGCACCTGAGCGTCAGTCTTTGTCCAGGGGGCCGCCTTCGCCACCGGTA

TTCCTCCAGATCTCTACGCATTTCACCGCTACACCTGGAATTCTACCCCC

CTCTACAAGACTCTAGCCTGCCAGTTTCGAATGCAGTTCCCACGTTGAGC

CCGGGGATTTCACATCCGACTTGACAGACCGCCTGCGTGCCGCTTTACGC

CCAGTAATTTCCGATTAACGCTTGCACCCTCCGTATTACCGCGGCTGCTG

GCACGGAAGTTAGCCCGGTGCTTCTTCTGGGAGTAACGTCAAATCACTTG

GTGGGTTATTAACCCACCAATGCCCTTCCCTCCTCGCTGAAAGGTACCTT

TACAACCTCGGAAGGCCCTTTCTTTCATTAAACGCGGGCAATGGCTGGAA

TCCAGCTTTGCGCCCCATTTGAGCAAATATTTCCCCACTGCCTGGCTCCC

GTTAGGAGATCTTGCACGGTCTTCAGTTCAGTGTGGCGTTGGACTATCCT

CTTCTCCAAGAACCAGCTTATGCGGAGAATCCGTGC

>SS26

ACCAAGGGTAGCGGTTTTCAACTTACCCCAGTCATGAATCACAACCGTGG

TAACCGTCCCCCCGAAGGTTAGACTAGCAACATCTGGTGCAACCCACTCC

CATGGTGTGACGGGCGGTGTGTACAAGGCCCGGGAACGTATTCACCGCGA

CATTCTGATTCGCGATTACTAGCGATTCCGACTTCACGCAGTCGAGTTGC

AGACTGCGATCCGGACTACGATCGGTTTTATGGGATTAGCTCCACCTCGC

GGCTTGGCAACCCTTTGTACCGACCATTGTAGCACGTGTGTAGCCCAGGC

CGTAAGGGCCATGATGACTTGACGTCATCCCCACCTTCCTCCGGTTTGTC

ACCGGCAGTCTCCTTAGAGTGCCCACCATTACGTGCTGGTAACTAAGGAC

AAGGGTTGCGCTCGTTACGGGACTTAACCCAACATCTCACGACACGAGCT

GACGACAGCCATGCAGCACCTGTCTCAATGCTCCCGAAGGCACCAATCTA

TCTCTAGAAAGTTCATTGGATGTCAAGGCCTGGTAAGGTTCTTCGCGTTG

CTTCGAATTAAACCACATGCTCCACCGCTTGTGCGGGCCCCCGTCAATTC

ATTTGAGTTTTAACCTTGCGGCCGTACTCCCCAGGCGGTCAACTTAATGC

GTTAGCTGCGCCACTAAGAGCTCAAGGCTCCCAACGGCTAGTTGACATCG

TTTACGGCGTGGACTACCAGGGTATCTAATCCTGTTTTGCTCCCCACGCT

TTCGCACCTCAGTGTCAGTATCAGTCCAGGTGGTCGCCTTTCGCCACTGG

TGTTCCTTCCTATATCTACGCATTTCACCGCTACACAGGAAATTCCACCA

CCCTCTACCATACTCTAGCTTGCCAGTTTTGGATGCAGTTTCCCAGGTTG

AGCCCGGGGATTTTCACATTTCAACTTAAACAACCACCTACGCGCGCTTT

ACGCCCAGTAATTCCGAATAACGGCTTGCACCCTCTGTATTACCGCGGCT

GCTTGCACAGAGTTAGCCGGGGGCTTATTTCTGTCGTAACGTCCAAAACC

AATTTACGTAATTAAGGTAACTGCCCTTTCCTCCCAACTTTAAGGTTGCT

TTTACAAATCCGGAGAACTTTCTTTCCACAAACGCGGGCATGGGCTGGGA

TACGGCTTTTCGACCCAATTGGTACCAATATTTCCCCACTGGCTGCTTCC

GTAAGAGACTGTGGAACCGGGTCTCAAGCTTCCAGTGTGTGACCTGAAAC

TACTTCCTCTTTCGAGACCAGCTATACGGGAATTGAGATGGGCGTTTTGT

TGA

>SS27

TAGGAAGAGGGAAGGGGGGCTCTCGCGGGGGCTATGCCAGGGGGGGACGG

GTGACAAAAAGAAGGCCGGGAACGTTTCACCGCGCATTCTGATCTACTTT

TACTAGCGATTCCGACTTCCGGTTTCGAGTTGCAGACTGCGATCCGGACT

ACGATCTACTTTATGAGGGTGGCGCGCCCTCGCGAGTTCGCTACTCTTTG

TATATGCCATTGTAGTCGTGTGTAGCCCTAGTCGTAAGGGCCATGATGAC

TTGACGTCATCCCCACCTTCCTCCGGTTTGTCACCGGCAGTCTCCTTAGA

GTGCCCACTATGACGTGCTGGAACAAGGATAAGGGTTGCGCTCGTTGCGG

TTTTTAACCCAACATTTCACAACACTTGCTGACGACACCCATGCGGCACC

TGTCTCAGAGTTCCCGAAGGCACCGATCTATCTCTGGAAAGTTCTGTGGA

TGTCAAGAGTAGGTA

>SS28

CTGGTGGTTAACGAACTTCCCCCCAGTTCATGAATCACAAAGTGGGGTAA

GCGCCCTCCCGAAGGTAAGCTAGCAAAAGGAGGGGCCCCCCCTCCCATGG

TGTGACGGGCGGCCCGCTAAAGACCGGGAACGTATTCGCCGTGGCATTCT

GATCCACGATTACTAGCGATTCCGACTTCACGGAGTCGAGTTGCAGACTC

CGATCCGGACTACGACATACTTTATGAGGTCCGGTTGGTCTCGCGAGGTC

GCTTCTCTTTGTATATGCCATTGTAGCACGTGTGTAGCCCTACTCGTAAG

GGCCATGATGACTTGACGTCATCCCCCCCTTCCTCCTGTTTATCTCTGGC

AGTCTCCTTTGAGTTCCCGGCCGAACCGGTGGGAATTAGGAAAAGGGT

>SS29

ACGGCATGGGGTCTTGGTTTCGACTTCCCCCAGTCATGAATCACAAAGTG

GTAAGCGCCCTCCCGAAGGTTAAGCTAGGAAACTGTTGCAACCCACTCCA

TGGTGTGACGGGCGGTGTGCACAAGGCCCGGGAACGTATTCACCGTGGCA

TTCTGATCCACGATTACTAGCGATTCCGACTTCACGGAGTCGAGTTGCAG

ACTGCGATCCGGACTACGACATACTTTATGAGGTCCGCTTGCTCTCGCGA

GGTGGCTTCCCTTTGTATATGCCATTGTAGCACGTGTGTAGCCCTACTCG

TAAGGGCCATGATGACTTGACGTCATCCCCACCTTCCTCCAGTTTATCAC

TGGCAGTCTCCTTTGAGTTCCCGGCCTAACCGCTGGCAACAAAAGAAAAG

GGTTGGCCTCGTTGCGGGAATTAACCCAACCTTTCCCAACACGAACTTAA

TAACGCCCTGGAGCACCTGGCTCAGAGTTCCCGAAAGCACCAAATCATCT

CTGCTAAAGTTCCTGGATGTCAAGAGTAGGTAAGGGTCTTCGCGTTGGCT

CCAATTAAACCACATGGTCCACCGCTTGGGGCGGCCCCCGGCAATTCATT

TGAGTTTTAACCTTGCGGCCGTACTCCCCAAGGGGGCGACTTAACGCGTT

AACTCGCGAAGCCACTCCTCCAAGGAATAACCTCCAAGTCGACATCGTTT

ACAGCGTGGACTACCAGGGTATCTAATCCTGGTTGCTCCCCAAGCTTTCC

ACCTGAGCGTCAGTCATTGTCCAGGGTGCCCCCCTCCCCCCCGGGATGTC

CTCTACAACACTACGCATTTCAACCGCTACACCTGGAAATTCTACCCCCC

TCCACAAGACTCTAACCTGCCAGTTTCGGATGGAGATTCCAGGGTGAACC

CGGGGATTTTCAATTCGACCTGACAGACCGACTGCGTGCGGCCTTACCGC

CCATAATTTCCGAATTAACGCCTTGGCACCCTTCGTATTACCGGGGCTGG

TTGGACGAATTAGCCGGGGGTCATCTGCGAGTAACGCCATCACAAGGGTA

TTTAACCTTATCGGCCTTCTCTCTCACTGAAAGGAGTTACCACCGAAGGA

CCTTCTTCCAACCACCGGCATGGCTGCGAACAGGCGTTGGCGCCAATTTG

TGCCATAATTCCCCCTTGGGGCCTTCCGGAAGGATCTCGGAACGGGGCTC

AATTTCCGGGGTGTGGATGGGGTAACCAT

>SS30

TCGGGGATACCTGGTTTCCGACTTCCCCCAGGTCATGAATCACACCGTGG

GTACCGTCCCCCCGAAGGTTAGAGTAGAGACGGCAGGGGCGACCCACTCC

CATGGTGTGACGGGCGGCGAGTACAAGGCCCGGGAACGTATTCACCGCGA

CATTCTGATTCGCGATTACTAGCGATTCCGACTTCACGCAGTCCAGTTGC

AGACTGCGATCCGGACTACGATCGGTTTTGTGGGATTAGCTCCACCTCGC

GGCTTGGCAACCCTTTGTACCGACCATTGTAGCACGTGTGTAGCCCAGGC

CGTAAGGGCCATGATGACTTGACGTCATCCCCACCTTCCTCCGGTTTGTC

ACCGGCAGTCTCCTTAGAGTGCCCACCATAACGTGCTGGTAACTAAGGAC

AAGGGTTGCGCTCGTTACGGGACTTTACCCTACATCTCACGACACGAGCT

GACGACAGCCATGCAGCACCTGTGTCAAAGTTCCCGAAGGCACCAATCTA

TCTCTAGAAAGTTCTTTGCATGTCAAGGCCTGGTAAGGTTCTTCGCGTTG

CTTCGAATTAAACCACATGCTCCACCGCTTGTGCGGGCCCCCGTCAATTC

ATTTGAGTTTTAACCTTGCGGGCGTACTCCCCAGGCGGTCACTTAATGCG

TTAGCTGCGCCACTAATAGCTCAAGGATTCCCAGGGCTAATTGACATCGT

TTACGGCGTGGACTACCAGGGTATCTAATCCTGTTTGGCTCCCCACGCTT

TCACACC

>SS31

AATTATGAGGAGAGAAGCTTGGGCCGAGAGTTCACATGGGGGTGGATGGG

TGGAAAAGGGGAGGTCGGGAACGTTATTCCCGCGAATTCTGATCTACGTT

ACTAGCGATTCCGACTTCCGGAGTCCAGTTGCAGACTCCGATCCGGACTA

CTATATACTTTGTGGGGTTGGGTTGCTCTCGCGGGGTTTCTTCCCTTTGT

ATCGACCATTGTAGCACTTGTGTACCCCTAGTCGTAAGGGCCATGATGAC

TTGACGTCATCCCCACCTTCCGCCAGTTTATCACTGGCAGTCTCCTTTGA

GTTCCCGGCCTAACCGCCTGTAACCTAAGAACAAGGTTGGGCTTCGTT

>SS32

GATGGGGAAAAAAGGCCGGGAAGTGGCTCGGTACTCCCTGGGTGACGGGT

GGTGAGAGGGGGGCCCGGGAACGTATTCCCGTAGCATTCTGATCTACGAT

TATTAGCGATTCCGACTTCTGGAGTCCCCTTGCATACTCCTATCCGGACT

ACGACATACTTTATGAGGTGGGGGTTGGGCTCGCGAGTTCGCTTCTCTTT

GTATATGCCATTGTAGCACGTGTGTAGCCCTACTCGTAAGGGCATGATGA

CTTGACGTCATCCCCACCTTCCTCCGGTTTATCACCGGGAGTCTCCTTTG

AGTTCCC

>SS33

CGTAGGGTTCCTTTGGTACGACTTCACCCCAGTCATGAATCACAAAGTGG

GTAAGCGCCCTCCCGAAGGTTAAGCTAGGAAAGCGGGTGGGCAAGCCACT

CCCATGGGGGGACGGGGGGTGTGCACAAAGGCCCGGGAACGTATTCACCG

TATCATTCTGATCTACGATTACTAGCGATTCCGACTTCACGGAGTCGAGT

TGCAGACTCCGATCCGGACTACGACATACTTTGTGAGGTCCGCTTGCTCT

CGCGAGGTCGCTTCTCTTTGTATATGCCATTGTAGCACGTGTGTAGCCCT

ACTCGTAAGGGCCATGATGACTTGACGTCATCCCCACCTTCCTCCAGTTT

ATCACTGGCAGTCTCCTTTGAGTTCCCGGCCGAACCGCTGGCAACAAAGG

ATAAGGGTTGCGCTCGTTGCGGGACTTAACCCTACATTTCACAACATGAG

TTTACGACAGCCATGCAGCACCTGTCTCAGAGTTCCCGAAGGCACCAAAG

CATCTCTGCTAAGTTCCCTGGATGTCAAGAGTAGGGAAGGGTCTTCGCGT

TGCATCGAATTAAACCAAATGCTCCACCGCTTGTGCGGGCCCCCGTCAAT

TCATTTGAGTTTTAACCTTGCGGCCGTACTCCCCAGGCGGTCGACTTAAC

GCGTTACTCCGGAAGCCACTCCTCAAGGGAACAACCTCCAAGTCGACATC

GTTTACCGCGTGGACTACCAGGGTATCTAATCCTGTTTGCTCCCCACGCT

TTCGCACCTGAGCGTCAGTCTTTGTCCAGGGGGGCGCCTTCGCCACCGGG

ATTCCTCCGATCTCTACGCATTTCACCGCTACACCTGGAAATCTACCCCC

CTTCTAAAAGACTCTAGCCTGGCAGTTTCGAATTCAGTTCCCAGGTTGAG

CCCCGGGGATTTCACATCCGACTTGATAGACCGCCTGCGGCCGCTTTACG

CCCCATAATTCCGATTTAACGCTTGCACCCCTCCGATTATCCCCGGGCTG

CCTGGCACGAATTAGCCGGGGCCTTCTTCCTGC

>SS34

CATGGTGTGACGGGCGGTGTGTACAAGGCCCGGGAACGTATTCACCGTAA

CATTCTGATCTACGATTACTAGCGATTCCGACTTCATGGAGTCGAGTTGC

AGACTGCGATCCGGACTACGACGTACTTTATGAGGTTAGCTTGCTCTCGC

GAGTTGGCTTCTCTTTGTATACGCCATTGTAGCACGTGTGTAGCCCTACT

CGTAAGGGCCATGATGACTTGACGTCATCCCCACCTTCCTCCGGTTTATC

ACCGGCAGTCTCCTTTGAGTGCCCGACCTAATCTGTGGGTAACAAAGAAA

AAGGTTGGGCTCCGTGAGGGAATTAACCCAACATTTCCCAAAACGAATTT

TAGAAAGCCATTGAAGGCCTTGTTCAAAGGTTCCGAAAGGACCCATCCTT

CTCTGGAAAGTTCTCTGGATGTCAAAAGTAGGTAAGGGTCTTCCCGTTGG

CTTCAAATAAACCACATGGCCCACCGGTTGGGCGGGCCCCCGTCCATTCA

TTTGGAGTTTAAACTTGGCGGCGGTATCCCCAAGGCGGCGATCTAAAGCG

TTAGCTCCCGAAGCCACGCCTCCAAGGCACACCATCGCTATCGAACTCCT

TTACAGGCTGGAATAACAAGGTAACTAAACCTGGTTGGTCCCCACGGTTT

CCAACTGAGCGGCAGTCATTGTCCAAGGTGGCGCCTTCCCCACCTGGAGT

CCTCCCCATCTCTACGCATTTCCCCGCTACACCAGGAATTCTACCCCCCC

CCTACACGACTCTAGCTTGGTCATTTCTAAGCAGGTTCCCGTTAGAGCCG

GGGATTTCTCATCTGAATTAACCAACCCACTGTGTGCGCGTTTAGCCCAA

AGAATTCCCATAAAGCTTGACCCTCTCGTATAACCCGGGCTGCTGGACGA

GTTAGACGGGGCTTCTTTCTGCGAGTAAGCTATGACAAGTGCTATTAAAG

CAAATGCCTTTCTCCTCCCACGAAGGTGCTTTTACAAACCTCAAAGACTT

TCTTCACACACGGCCGGAGTGGGCT

>SS35

GTAAGCGCCCTCCCGAAGGTTAAGCTAATAAAAAATTTTGCAACCCACTC

CCATGGTGTGACGGGCGGTGTGTACAAGGCCCGGGAACGTATTCACCGTA

GCATTCTGATCTACGATTACTAGCGATTCCGACTTCATGGAGTCGAGTTG

CAGACTCCAATCCGGACTACGACGTACTTTATGAGGTCCGCTTGCTCTCG

CGAGTTCGCTTCTCTTTGTATACGCCATTGTAGCACGTGTGTAGCCCTAC

TCGTAAGGGCCATGATGACTTGACGTCATCCCCACCTTCCTCCGGTTTAT

CACCGGCAGTCTCCTTTGAGTTCCCGACCGAATCGCTGGCAACAAAGGAT

AAGGGTTGCGCTCGTTGCGGGACTTAACCCAACATTTCACAACACGAGCT

GACGACAGCCATGCAGCACCTGTCTCAGAGTTCCCGAAGGCACCAATCCA

TCTCTGGAAAGTTCTCTGGATGTCAAGAGTAGGTAAGGTTCTTCGCGTTG

CATCGAATTAAACCACATGCTCCACCGCTTGTGCGGGCCCCCGTCAATTC

ATTTGAGTTTTAACCTTGCGGCCGTACTCCCCAGGCGGTCGATTTAACGC

GTTAGCTCCGGAAGCCACGCCTCAAGGGCACAACCTCCAAATCGACATCG

TTTACAGCGTGGACTACCAGGGTATCTAATCCTGTTTGCTCCCCACGCTT

TCGCACCTGAGCGTCAGTCTTTGTCCAGGGGGCCGCCTTCGCCACCGGTA

TTCCTCCAGATCTCTACGCATTTCACCGCTACACCTGGAATTCTACCCCC

CTCTACAAGACTCTAGCTTGCCAGTTTCAAATGCAGTTCCACGGTAAGCG

CGGGATTTCACATCTGACTTAACAAACCGCCTGCGTGCGCTTTACGCCAG

TAATTTCCGATTAACCGCTTGCCCCCTCCGTATTACGGCGGCTGCTGGCA

CGGAGTTAGCCGGTGCTACTTCTGCCGAGTACCGTCAATGCAATGGGGCT

ATTAGCACCACTACCCTTTCGTCTTGGCTGAAAGTCTTAACATCCTGAAG

>SS36

TCCCAGGGGGGGAGGGGGGGGGTGTACAAGACCCGGGAACGTATTCCCCG

TGAATTCTGACTACGATTACTGCGATTCCGACTTCTGGAGTCGAGTTGCA

GACTGCGATCCGACTACGACTGGTTTTATGGGATTACGTGGTGTCGCGGT

TGGTTCCCTTTGTTAACCATTGTATGACGTGTGTAGCCCCACCTATAAGG

GCCATGATGACTTGACGTCATCCCCACCTTCCTCCGGTTTGTCACCGGCA

GTCCCCTTTGAGTGCTCAACTGAATGGATGAACTAATGGGAAAGGGTGCG

CTCTCTGTGGGACATAAACCCCCATCTTTCAACACAAGATGAGAACACCC

CTGTGCGACGTGTGTGTCAGATCTCTTTAAAGCACCAAACCATCTCTGGA

AAGTTCCTGCCATGTCAAAAGTAGGTAAGGTTCTTCGCGTTGCATCGAAT

TAAACCACATCATCCACCGCTTGTGCGGGTCCCCGTCAATTCCTTTGAGT

TTTAACCTTGCGGCCGTACTCCCCAGGCGGTCAACTTCACGCGTTAGCTT

CGTAACTGAGTCCGCTAAGACACAACCACCAGATCACATCGTTTACGGCG

TGGACTACCAGGGTATCTAATCCTGTTTGCTCCCCACGCTTTCGTGCATG

AGCGTCAGTGTTTGCCCAGGGGATCGCCTTCGCCATCGGTGTTCCTCCGC

ATATCTACGCATTTCACTGCTACACGCGGAATTCCATCCCCCTCTGCCAC

ACTCTAGCTTTGCAGTCACAAAGGCAGTTCCCACGTTGAGCGCGGGGATT

TCACCTCTGACTTACAAACCGCCTGCGCGCGCTTTACGCCCAGTAATTCC

GATTAACGCTTGCACCCTACGTATTACCGCGGCTGCTGGCACGTAGTTAG

GCGGTGCTTCATTTCTTGACAGTTACCGTCATGACACTGGAGCTATTAGC

CACCTTAGCTTTTCCTCTCTCCGCTCGAAGTGCATTTAAACCTGAGGCAT

CTATCATGCACGCGCATTGCTGCGATTCAGGCCTTGTCGCGCTATTGCAT

ATTCCCCACTGGCTGGCTTCCGTAGAGTCTGACGGGTTCTCAGTCAGTGG

>SS37

GGGGGGGGGGCCCTGGGTTTCACTTTCCCCCAAGTCATGAATCACAAAGG

TGGTAAAGCGCCCTCCCGAAGGTTAAGCTAAAACAAAATTTTGCAAACCA

CTCCCATGGGGGGACGGGGGGGGTGTAAAAGGACCGGGAACGTATTCACC

GTAGCATTCTGATCTACGATTACTAGCGATTCCGACTTCATGGAGTCGAG

TTGCAGACTCCAATCCGGACTACGACGTACTTTATGAGGGCCGCTTGCTC

TCGCGAGTTCGCTTCTCTTTGTATACGCCATTGTAGCACGTGTGTAGCCC

TACTCGTAAGGGCCATGATGACTTGACGTCATCCCCACCTTCCTCCGGTT

TATCACCGGCAGTCTCCTTTGAGTTCCCGACCGAATCGCTGGCAACAAAG

GATAAGGGTTGCGCTCGTTGCGGGACTTAACCCAACATTTCACAACACGA

TCTGACGACAGCCATGCAGCACGTGTGTCAGAGTTCCCGAAGGCACCAAT

CCATCTCTGGAAAGTTCTCTGGATGTCAAGAGTAGGTAAGGTTCTTCGCG

TTGCATCGAATTAAACCACATGCTCCACCGCTTGTGCGGGCCCCCGTCAA

TTCATTTGAGTTTTAACCTTGCGGCCGTACTCCCCAGGCGGTCGATTTAA

CGCGTTAGCTCCGGAAGTCACGCCTCAAGGGCACAAACCTCCAAATCGAC

ATCGTTTACAGCGTGGACTACCAGGGTATCTAATCCTGTTTGCTCCCCCA

CGCTTTCGCACCTGAGCGTCAGTGTTTGTCCAGGGGGCCGCCTTCGCCAC

CGGTATTCCTCCACATCTCTACGCATTTCACCGCTACACCTGGAATTCTA

TCCCCCTCTACAAGACTCTAGCTTGCCAGTTTCAAATGCAGTTCCCACGT

TAAGCGCGGGGATTTCACATCTGACTTACCAAACCGCCTGCGTGCGCTTT

ACGCCCAGTAAATCCGATAACGCTTGCACCCTACGTATTACCGCGGCTGC

TGGCACGGAGGTAGCCGGTGGCTTACTTCTTGCGGTAACGGTCATGCAAT

GTGGCTATAACCCACCTAGCCTTTCCTCCTCGACTGAAAGCGCTTTAACA

ACCCTAAGGGCTTTTCATCCCAGAC

>SS38

GAGGAATGAGGAAAAAGGGGGGAAGCGCGTGTATCCCATGGGGTGACGGG

CGGAGAGAAGGAAAGCCGGGAACGTATTCACCGTAACATTCTGATCTACG

ATTACTTAGCGATTCCGACTTCTGGAGTCTAGTTGCAGACTGCAATCCGG

ACTACGACATACTTTATGAGGGCCGCTGGGTCTCGCGAGTTCGCTTCTCT

TTGTATATGCCATTGTAGCACGTGTGTAGCCCTACTCGTAAGGGCCATGA

TGACTTGACGTCATCCCCACCTTCCTCCGGTTTATCACCGGCAGTCTCCT

TTGAGTTCCCACCATTACGTGCTGGCAACAAAGGATAAGGGTTGCGCTCG

TTGCGGGACTTAACCCATCATTTCACAACACGATCTGACGACAGCCATGC

AGCACCGGTCTGAGGGTTCCCGAAGGCACTAATCCATCTCTGGAGAATTC

CGTGGATGTCAAGAGAAGGTAAGGGTCTTCGCGTTGCATCGAATTAAACC

ACATGCTCCACCG

>SS39

AGCTATAGGTGTTTGGTTAGACTTCCCCCAGTCATGAATCACACCGTGGT

AAACGCCCTCCCGAAGGTTAAACTATAAAGGGTGGTGCAACCCACTCCCA

TGGTGTGACGGGCGGTGTGTACAAGGCCCGGGAACGTATTCACCGCAACA

TTCTGATTTGCGATTACTAGCGATTCCGACTTCACGGAGTCGAGTTGCAG

ACTCCGATCCGGACTACGATCCGCTTTGTGGGATTAGCTCACTCTCGCGA

GTTGGCAACCCTCTGTACCCGCCATTGTAGCACGTGTGTAGCCCTGGCCG

TAAGGGCCATGATGACTTGACGTCATCCCCACCTTCCTCCGGTTTATCAC

CGGCAGTCTCCTTTGAGTGCCCACCATTACGTGCTGGTAACTAAGGACAA

GGGTTGCGCTCGTTGCGGGACTTAACCCAACATCTCACGACACGAGCTGA

CGACAGCCATGCAGCAGCTGTGTCATAATTCCCGAAGGCACTCATCCATC

TCTAGAGAATTCTCTACATGTCAAGGCCAGGTAAGGTTCTTCGCGTTGCA

TCGAATTAAACCACATGCTCCACCGCTTGTGCGGGCCCCCGTCAATTCAT

TTGAGTTTTAACCTTGCGGCCGTACTCCCCAGGCGGTCAACTTAACGCGT

TAGCTGCGGAAGCCACATCTCAAGGACACCAACTCCTAATCGACATCGTT

TACGGCGTGGACTACCAGGGTATCTAATCCTGTTTGCTCCCCACGCTTTC

GCACCTGAGCGTCAGTCTTTGTCCAGGGGGCCGCCTTCGCCACCGGTGTT

CCTCCAGATCTCTACGCATTTCACCGCTACACCTGAAATTCTACCCCCCT

CTACAGTACTCTAGCTTGACAGTTTTAGATGCAATTCCCAGGGTTGAGCC

CGGGGCTTTCACATCTAACTTATCAAACCGCCTGCGTGCGCTTTACGCCC

AGTAATTCCGATTAACGCTTGCACCCTCTGTATTACCGCGCTGCTGGCAC

GGAGTTAGCCGTGCTTACTTCTGCCAGTAACGTCCACAACTGAAACGTTA

TTAGCTATCATGCCTTTTCTCTCACTGAAAGATGCTTACAATCCGGAAGG

CGTTCTTCAACACCGCGCATGGCTGATCAGGCTTTCGCCCGAATGTCAAT

ATTGCCCAACTGCCTGCTTCGTAGGAGCCTGGGACGGGGTCTTCAGTCCA

GTGTGGACTTGAT

>SS40

GGGGGGGGTTGTTGGTTAGAACTTCCCCCAGTCATGATCACAAAGTGGGA

AGCGCCCTCCCGAAGGTTAAACTAAAAACATCATTTGAACCACTCCCATG

GTGTGACGGGGGCCGTGTAAAGGCCCGGGAACGTATTCACCGTAACATTC

TGATCTACGATTACTAGCGATTCCGACTTCATGGAGTCGAGTTGCAGACT

CCAATCCGGACTACGACGTACTTTATGAGGGCCGCTTGCTCTCGCGAGTT

GGCTTCCCTTTGTATACGCCATTGTAGCACGTGTGTAGCCCTACTCGTAA

GGGCCATGATGACTTGACGTCATCCCCACCTTCCTCCGGTTTATCACCGG

CAGTCTCCTTTGAGTTCCCGACCGAATCGGTGGGAACAAAGGAACAGGGG

TGCGCTCGTTGCGGGAATTTACCCCACATTTCCCATAATTTTTTTGAAGA

AAGCCCTTGCGCGCCGGGCTCAAAATTCCCGAAGGGCCCCATCCCTCTCT

GGAAAGGTCTCTGGATGTCAAAAGGATGGAAGGGTCTTCCCGTTTCATCC

AAATAAAACCAATGCTCCCCCGCTTGGGCGGGCCCCCGGCAATTTCTTTG

AATTTTAACCTTGGCGGCGTACTCCCCCGGGGGGCGAATTAAATGGTTAG

CTCGGGAAGCCACACCCTCAAGGGCACAACCTCCAAATCGAATCCGTTAC

AGCGTGGAATACCAGGGTATCCAATCCCGGTTGGTCCCCACGGCTTCCCA

ACTGAGCGGCAGTCATCGTCCAGGGGGCCGCCTTCGCACCGGTATTCCTC

TAGATCTCCACGCATTTCACCGCTAACCTGGAAATCCACCCCCCCTCCAC

ACGACTCTAGCTTGGCAGTTTCAGATGCAGTTCCACGGTAGCGCGGGGAT

TTCACATCTGACTTAACCAACCCCCTGCGTGCGCTTTACCCCCAGTAATC

CGAATATAAGCTTGCGACCCTCCGTGATTTACCGCGGGCTGGCGGGCACG

GAATTAGCCCGGGGGCTCATTCTGCGAGGAACATCAAATGAAAATGTGCT

ATAAAAGCACTATGCCTTCCTCCCTCCGCTGAAAGAGGGCTTTTAACACA

ACTCAAGGACTTTCTTCATACACACCCGCGGCGCAATGGGTTGCGTAAAG

GGGTGTGATTCGCAATGGGGCATAAATTTCCCCTCGTGGGTGGCCGCCCC

CACCGTAGGAAAGAATCTTGGA

>SS41

CGCGTGGCAACCTTGGTTCGACTTCACCCAGTCATGAATCACACCGTGGT

AACCGTCCTCCCGAAGGTTAGACTAGAACATCTGGTGCAACCCACTCCCA

TGGTGTGACGGGCGGTGTGTACAAGGCCCGGGAACGTATTCACCGCGACA

TTCTGATTCGCGATTACTAGCGATTCCGACTTCACGCAGTCGAGTTGCAG

ACTGCGATCCGGACTACGATCGGTTTTGTGAGATTAGCTCCACCTCGCGG

CTTGGCAACCCTCTGTACCGACCATTGTAGCACGTGTGTAGCCCAGGCCG

TAAGGGCCATGATGACTTGACGTCATCCCCACCTTCCTCCGGTTTGTCAC

CGGCAGTCTCCTTAGAGTGCCCACCATTACGTGCTGGTAACTAAGGACAA

GGGTTGCGCTCGTTACGGGACTTAACCCAACATCTCACGACACGAGCTGA

CGACAGCCATGCAGCACCTGTGTCAAAGTTCCCGAAGGCACCAATCCATC

TCTGGAAAGTTCATTGGATGTCAAGGCCTGGTAAGGTTCTTCGCGTTGCT

TCGAATTAAACCACATGCTCCACCGCTTGTGCGGGCCCCCGTCAATTCAT

TTGAGTTTTAACCTTGCGGCCGTACTCCCCAGGCGGTCAACTTAATGCGT

TAGCTGCGCCACTAAAATCTCAAGGATTCCAACGGCTAGTTGACATCGTT

TACGGCGTGGACTACCAGGGTATCTAATCCTGTTTGCTCCCCACGCTTTC

GCACCTCAGTGTCAGTATGAGCCCAGGGTGGTCGCCTTCGCCACTGGTGT

TCCTTCCTATATCTACGCATTTCACCGCTACACAGGAAATTCCACCACCC

TCTACCCTACTCTAGCTCGCCAGTTTTGGATGCAGTTCCCAGGTTGAGCC

CGGGGATTTCACATCCAACTTAACGAACCACCTACGCGCGCTTTACGCCC

AGTAATTCCGATTAACGCCTTGCACCCTCTGTAATAACCCGCGCTGCTGG

CCAGAGTTAGCCCGGTGCTTTATTCTGTCCGGTACGTCAAACACAGCAAA

GGTATTAGCCTTACTGGCCCTTCCTCCCAAGCTTAAAGTGCTTTAACAAT

CCGAAGACTTCTTTCCACAACACGCGAATGGCTTGGATCCAGGCTTCGCC

AATTGGTCAATATCCCCTACTGCTGCCTTCTCGGTAGGAGTCCTGGA

>SS42

CCTGGTGGCTGCCTTGGTTCGACTTCACCCCAGTCATGAATCACACCGTG

GTAAAACGCCCTCCCGAAGGTTAAACTAAAACATCTTAGTGCAACCCACT

CCCATGGTGTGACGGGCGGTGTGTACAAGGCCCGGGAACGTATTCACCGT

AACATTCTGATCTACGATTACTAGCGATTCCGACTTCACGGAGTCGAGTT

GCAGACTCCGATCCGGACTACGACATACTTTATGAGGGTAGCTTGCTCTC

GCGAGTTGGCTTCCCTCTGTATATGCCATTGTAGCACGTGTGTAGCCCTA

CTCGTAAGGGCCATGATGACTTGACGTCATCCCCACCTTCCTCCGGTTTA

TCACCGGCAGTCTCCTTTGAGTTCCCGACATTACGTGCTGGCAACAAAGG

AAAAGGGTTGCGCTCGTTGCGGGACTTAACCCAACATTTCACAACACAAT

TTGACGACAGCCATGCAGCACCTGTGTCAAAATTCCCGAAGGCACTAACC

TATCTCTAGAAAATTCTCTGGATGTCAAAGCCAGGTAAGGTTCTTCGCGT

TGCTTCGAATTAAACCACATGCTCCACCGCTTGTGCGGGCCCCCGTCAAT

TCATTTGAGTTTTAACCTTGCGGCCGTACTCCCCAGGCGGCCAACTTAAC

GCGTTAGCTCCGGAAGCAACGTCTCAAGGATACACCCTCCAAGTTCGACA

TCGTTTACGGCGTGAACTACCAGGGTATCTAATCCTGTTTGCTCCCCACG

CTTTCGCACCTGAGCGTCAGTATTTTGTCCAGGGGGGCCGCCTTCCCCAC

CGGTATTCCCTCCCAGATCTCTACGCATTT

>SS43

CCCATGGTGTGACGGGCGGTGTGTACAAGGCCCGGGAACGTATTCACCGT

GGCATTCTGATCCACGATTACTAGCGATTCCGACTTCACGGAGTCGAGTT

GCAGACTCCGATCCGGACTACGACATACTTTATGAGGTCCGCTTGCTCTC

GCGAGGTCGCTTCTCTTTGTATATGCCATTGTAGCACGTGTGTAGCCCTA

CTCGTAAGGGCCATGATGACTTGACGTCATCCCCACCTTCCTCCAGTTTA

TCACTGGCAGTCTCCTTTGAGTTCCCGGCCGAATCGCTGGCAACAAAGGA

TAAGGGTTGCGCTCGTTGCGGGACTTAACCCAACATTTCACAACACGATC

TGACGACAGCCATGCAGCACCTGTCTCAGAGTTCCCGAAGGCACCAAAGC

ATCTCTGCTAAGTTCTCTGGATGTCAAGAGTAGGTAAGGTTCTTCGCGTT

GCATCGAATTAAACCACATGCTCCACCGCTTGTGCGGGCCCCCGTCAATT

CATTTGAGTTTTAACCTTGCGGCCGTACTCCCCAGGCGGTCGACTTAACG

CGTTAGCTCCGGAAGCCACTCCTCAAGGGAACAACCTCCAAGTCGACATC

GTTTACAGCGTGGACTACCAGGGTATCTAATCCTGTTTGCTCCCCACGCT

TTCGCACCTGAGCGTCAGTCTTTGTCCAGGGGGCCGCCTTCGCCACCGGT

ATTCCTCCAGATCTCTACGCATTTCACCGCTACACCTGGAATTCTACCCC

CCTCTACAGACTCTAGCCTGCCAGTTTCAATGCAGTTCCCAAGGTGGAGC

CCGGGGGATTTCACATCCGACTTGACAGACCGCCTGCGTGCCGCTTTACG

CCCAGTAATTTCCGATTAACGCTTGCACCCTCCGTATTACCGCGGCTGCT

GGCACGGAGTTAGCCGGGGCTTCTTCTGCGATTACGTCAATGGATGGCGG

TATTAACCCACATCCCTTTCCTCCCTCGCCTGAAGGTACTTACAACCTGA

AGCCTTCTTTCATAACACGCGGCATGGCTTGCAATCAGCTGGCGCCAATG

GGGTAAAATTCCCCACTGGCTGGCCTCCGTAAGATCTGC

>SS44

GCCGGTGGCGGCCGGGGTTACGACTTTCCCCCAGTCATGAATCACACCGT

GGTAACCGCCCTCCCGAAGGTTAAACTAGAACATCTGGTGCAACCCACTC

CCATGGTGTGACGGGCGGTGTGTACAAGGCCCGGGAACGTATTCACCGCG

ACATTCTGATTTGCGATTACTAGCGATTCCGACTTCACGGAGTCGAGTTG

CAGACTGCGATCCGGACTACGATCGGCTTTGTGAGATTAGCTCCACCTCG

CGGCTTGGCAACCCTCTGTACCCACCATTGTAGCACGTGTGTAGCCCTGG

CCGTAAGGGCCATGATGACTTGACGTCATCCCCACCTTCCTCCGGTTTGT

CACCGGCAGTCTCCTTTGAGTGCCCACCATAACGTGCTGGTAACTAAGGA

CAAGGGTTGCGCTCGTTGCGGGACTTAACCCAACATCTCACGACACGAGC

TGACGACAGCCATGCAGCACCTGTGTTCTAATTCCCGAAGGCACCAATCC

ATCTCTGGAAAGTTCTCTACATGTCAAGGCCTGGTAAGGTTCTTCGCGTT

GCTTCGAATTAAACCACATGCTCCACCGCTTGTGCGGGCCCCCGTCAATT

CATTTGAGTTTTAACCTTGCGGCCGTACTCCCCAGGCGGTCAATTTAACG

CGTTAGCTGCGGAACTCACATCTCAAGGACACAAACGGCTAATTGACATC

GTTTACGGCGTGGACTACCAGGGTATCTAATCCTGTTTGCTCCCCACGCT

TTCGCACCTGAGCGTCAGTATCAGTCCAGGGGGTCGCCTTCGCCACTGGT

GTTCCTTCATATCTCTACGCATTTCACCGCTACACATGAAATTCTACCCC

CCTCTACCGGACTCTAGCTTGACAGTTTTAGATGCAGTTCCCAGGTTGAG

CCCGGGGCTTTCACATCTAACTTATCAAACCACCTACGCGCGCTTTACGC

CCAGTATTTCCGATTAACGCTTGCACCCTCTGTATTTACCGCGGGCTGCT

GGCACAGAAGTTAGCCCGGTGGCTTATTTCTGGCGAGTAACGTTCACAGC

TGCAAAGGGTATTAAGGTTATCAGCCTTTTCTCCCTACCTTGAAGGTGGC

TTTTACAACCCGGAAGGCCCTTCTTCCCCACACGCGGGCATGGCTGGCAT

TCAAGGGCTTTCCCCAATTGTGCCAATAATTCCCCACTTGCTGCCTTCCG

GTAGGAAGTCTGGAACGGTTCCAAGTCCAAGTTGTGACTTGATCATTCCT

CTCTAGGACCGAGCAACGGGATAGATGTCTGCGTCG

>SS45

AATTTAGTGAGGGAAGGCGGCTTCGCTTAATCCATGGGGTGGACGGGGGG

GAAAAGGGGGGGGCCGGGAACTTATTCACCGCAACATTCTGATCTACGTT

TTCTAGCGATTCCGACTTCACGGAGTCGAGTTGCAGACTGCAATCCGGAC

TACAATCGGTTTTGTGAGATGGGTGTGCTCTCGCGAGTTGGCTTCCCTTT

GTATATGCCAATGTAGCACGTGTGTAGCCCTAGCCGTAAGGGCCATGATG

ACTTGACGTCATCCCCACCTTCCTCCGGTTTATCACTGGCAGTCTCCTTA

GAGTGCCCAGCCGAACCTGTGGGAAACTAAGGACAGGGGTTGCCCTCTTG

ACGGATCTAACCTCTTCATTTTCCATAACCAATTTAACAAAGCCCTTGCA

CTACTGGGGGCAAAGTTCCCAAAGGCCCCAATCTTTCCTTGTAAAGTCTC

TGGATGCAAAGGCATGGAA

>SS46

AGGAAGTAAAGAAAGGGGGGGTGAGCGCAACCCACTCCCATGGTGTGACG

GGCGCCCGCGTAAAAAGGCCCGGGAACGTATTCACCGCGACATTCTGATT

CGCGATTACTAGCGATTCCGACTTCACGCAGTCGAGTTGCAGACTGCGAT

CCGGACTACGATCGGTTTTGTGAGATTAGCTCCACCTCGCGGCTTGGCAA

CCCTCTGTACCGACCATTGTAGCACGTGTGTAGCCCAGGCCGTAAGGGCC

ATGATGACTTGACGTCATCCCCACCTTCCTCCGGTTTGTCACCGGCAGTC

TCCTTAGAGTGCCCACCATAACGTGCTGGTAACTAAGGACAAGGGTTGCG

CTCGTTACGGGACTTATCCCAACATCTCACGACACGAGCTGACGACAGCC

ATGCAGCACCTGTGTCAGAGTTCCCGAAGGCACCAATCTATCTCTGGAAA

GTTCTCTGCATGTCAAGGCCTGGTAAGGTTCTTCGCGTTGCTTCGAATTA

AACCACATGCTCCACCGCTTGTGCGGGCCCCCGTCAATTCATTTGAGTTT

TAACCTTGCGGCCGTACTCCCCAGGCGGTCAACTTAATGCGTTAGCTGCG

CCCACTAAAATCTCAAGGATTCCCAACGGCTAGT

>SS47

GTTTACTCCTGAGGTGCTGAGCGTTGCCACGGGTGAGTAACGCATCGGGA

AATGCCCAATCGAGCGCGATAACAGTTGGAAACGACTGCTAATACCGCAT

ACGCCCTACGGGGGAAAGGAGGGCACCTTCGGGCCTTCCGCTATTGGATG

AACCTAGGTGGGCTTAGCTAGTTGGGGAGGTAATGCCTCACCGAGGCGAC

GATCCCTAGCTGTTCTGAAAGGATGATCACCCACACTGATACTGACAGTG

ATCCCAAACTCCCGCGTGGGCCACAGAGGGGGGGATATTGCCGGATGGGG

CTACCCTAGATTCCCCCATCCCGCGTGGGTGGAGAAGGGCTTCCTATTGG

ACTGCACTTTTCGAGATAGAGGACTGGCAGTAAACTTACTCCGTTATATC

TGCGGCGTTGCCTAAAGAAAGCGGAGCCGCTGACTTCGTTCCATAAAGCT

CGGAAAAACCGAAGGGTCCAGTCTTAATCGAAATTCCTGCACACTGTTCT

TGAACGAGACGGTTGATAGGCGAAGAAGTAGAACCCCGGGAGCTCATTTA

ATGAAAATACGTTTAGAAACTACTCACATATACTCT

>SS48

GAGTAGCTAGTTGGTGAGGTAATGGCTCACCAAGGCTACGATCCGTAACT

GGGCTGAGAGGATGATCAGTCACACTGGAACTGACCCAGGTTCAACTCTT

CTACGGGAGGCAGCAGTGGGGAATATTGGACAATGGGCGAAAGCCTGATC

CAGCCATGCCGCGTGTGTGAAGAAGGTCTTCGGATTGTAAAGCACTTTAA

GTTGGGAGGAAGGGCAGTTACCTAATACGTGACTGTCTTGACGTTACCGA

CAGAATAAGCACCGGCTAACTCTGTGCCAGCAGCCGCGGTAATACAGAGG

GTGCAAGCGTTAATCGGAATTACTGGGCGTAAAGCGCGCGTAGGTGGTTT

GTTAAGTTGAATGTGAAATCCCCGGGCTCAACCTGGGAACTGCATCCAAA

ACTGGCAAGCTAGAGTATGGTAGAGGGTAGTGGAATTTCCTGTGTAGCGG

TGAAATGCGTAGATATAGGAAGGAACACCAGTGGCGAAGGCGACTACCTG

GACTGATACTGACACTGAGGTGCGAAAGCGTGGGGAGCAAACAGGATTAG

ATACCCTGGTAGTCCACGCCGTAAACGATGTCAACTAGCCGTTGGGAGTC

CTTGAACTCTTAGTGGCGCAGCTAACGCATTAAGTTGACCGCCTGGGGAG

TACGGCCGCAAGGTTAAAACTCAATTGAATTGACCGGGGGCCCGCACAGC

CGTGGAAGCCATGTGGCTTAATTCGAAGCACGCCGAAGAACCTTTACCAG

CCCTTGACATCCCAATGAAACTTTTCAAAGAATAGAATTGGTGCTTTCGG

AACATTGGAAACAGGTGCCTTGCCATGCCTTGTTCGTCAAGCTT

>SS49

GGAAGAATATGACGCCTCTGTGCTACTCCACTTCGCGCCAAGTCGTGGCA

ATGAGTCCGGTGGCAAGGCAGGGAGGACGAAAAATGGGGCAGTCTGAAGA

GCCGCGCAGCGCGCGCTAAAAATTTTGAAACAGATACCGATGCGGCTGCG

CAGAATCGCGGTAAGTTGGCCGTAAAAAAAATTTATTGGCGACCAGCTTA

CCGGGCATCTTGGCGGGCGTGGCGTTAACTGTTTGGATTGTGAAGCCCAG

GGTGAACAACTCATCATACGGGCGGCAAATTTTGCCCTCACAGGTATGCC

CAGCGAACTGGTTGACATTGAATTAGGGATAAGTCAGATAGAACATTGGC

GAATTACCTCTGCCAATCTTGAGGCCGAGATCCAACTCTTATAGAACCGC

ACTCTAAGACTGCGCCAGCCGGCAGCAACGTACCTTATTATATATGCGAA

TTGCCAACAAAATACGGAGCTGATCACTCAGAGTCCATAGACCCCGCCCA

TACCCCACAGATTCCAATCGTATTCCGGAATAGCCTGGGACACCTGAGCT

CGAACCGAGTCTGGATTGTCACGGGCAAAATGCGAAGACCCCGTGAGCAG

CGAATTTAATTAACTACCG

>SS50

TCTTGAGAGCGGCGCTCGTCCGACCTAGGCCCATCTGTCTGCCTGGTAGT

GGGGGATAACGGTCGGATCGGACGCTCGTACCGCATACGTCCTACGCGTG

AAAGCAGGGGACCTTCGGGCCTTGCGCTATCAGATGAGCCTACGTCGGAG

TAGCTAGTTGGCGAGATAATGGCGCACCACGGCTACGATCCGTATCTGGG

CTGAGAGGATGATCAGTCACACTGGAACTGACCCTGGACCAACTCTTCTA

CGGGAGGCAGCAGTGGGGAATATTGGACAATGGGCGAAAGCCTGATCCAG

CCATGCCGCGTGTGTGAAGAAGGTCTTCGGATTGTAAAGCACTTTAAGTT

GGGAGGAAGGGCAGTTACCTAATACGTGACTGTCTTGACGTTACCGACAG

AATAAGCACCGGCTAACTCTGTGCCAGCAGCCGCGGTAATACAGAGGGTG

CAAGCGTTAATCGGAATTACTGGGCGTAAAGCGCGCGTAGGTGGTTTGTT

AAGATGAATGTGAAATCCCCCGGGCTCAACCTGGGAACTGCATCCAAAAC

TGGCAAGCTAGAGTATGGTAGAGGGTAGTGGACTTTCTTGTGTACCGGTG

AAATGCGTAGATATAGGAAGGAACACCAGTGTCGAAGGCGACTACCTGGA

CTGATACTGACACTGAAGTGCCAAAAGCGTGGGGAAGCAACAGGATTAGA

TACCCTGGTAAGTCCACGCCATAAACGATGTC

>SS51

CCTTGCCGTGGTCGCGGTCGTGCGTGCCGGGGGCTCACATGGTCAGCGCG

CCGTCGAGCCGAGATCGCACTGCCCCTCCCGGCGTACTACCTGCCCTTGC

GGATCCCACCAGCCAATCCTGCAAGATCGGGTACTTCGCGGGGCGCTGCA

CCGCGATCCCTTCGAGCAAATCCCGGCTCTGTTGACCGCCCGGCACCCCG

ACGCGGTGCCTGAGGACCCCGATGCTTAACCCTGGTCCAACTTTTAGACG

ATCCCTATCTGTGCTGAGAGGCTGATCACCCAAACTGGTCCTGACCCTGG

GTCAACTCTTCTGCGGCAGGTGGCTTTGGGCAATCCTGCGCTTTGGGAAA

GACCTGGTTCGCCATGCCGCGAGTGTGAAGAAGGCCTTCGCGTTGTAAAC

CACTTTCCGCGACGAGGAAGGAATGAGACCTAATATGTATCTCCCAGTGA

CGTTACTCGCAGATGAAGCGACGGCTACCTCCGTGCCTGCCGTGCGCCGT

>SS52

GAGGGCAAGGCCTGGATGAGCGGGGAGTTCGCGGGGCTCTGCAGCCCGAT

CCCTTCGAGCCAATCCCGACTCTGCTGACTCCCCGGCTTCCCGCCGCGGT

GCCGGAGGACCCCGGTGACTGGGCCCGTTCAACCTCTTCGTCGCTGGCCT

GATAGGCTGATCAGTCGCACTGGAACTGAGACACGGTCCCCTCTTCTACT

CTAAGCTGCGAGGGGGAATTGTGGACTATGGCCGAAAGCCTGAGCCCCCG

GTGCCGCCTAGGTGCGCGTGTGCTTCAGAATGTCAAGCACTTTAAGTTTT

TACGCAAGGACGTAAGTTTTTACCTTGCTGTTTTGACGTTGTCGAGTTAC

TAAGCAACGAATAACTCTGTACCTGCATGCCCGCTTACACGGAGATAGCG

AGCGTGCATCCGATATTCTGAGTTACTGGCGCACGATCGTCTCTTGTTTC

TTGGATAGTGTAAATGCCAAGGCTCACGCTGTCAACTGCATCCTTGACTG

AAAACTGTCCATACTGTAGACGTAGATAGGAATTTTCATGATGTACCGTG

AAATGCGTAAATGTAGAGAGATCTGCAAGATGTCCAGGGGGCTACACGCT

GGACCCATGCTGACACTGAGACTGCCCAAGCGGTGATATCGCGGAGAGCA

TTACGATGACTCTGATACTCCGACTACTGCTACGCGCATGATCAAGCTAG

TCCGTTTGGAAATCGCTTGGAGTCTCTTGAGTGCTTGCAGCTT

>SS53

TACTCCTGAGGTGGCGAGCGGCGGACGGGTGAGTAATGCCTAGGGATCTG

CCCAGTCGAGGGGGATAACAGTTGGAAACGACTGCTAATACCGCATACGC

CCTACGGGGGAAAGGAGGGGACCTTCGGGCCTTCCGCGATTGGATGAACC

TAGGTGGGATTAGCTAGTTGGTGAGGTAATGGCTCACCAAGGCGACGATC

CCTAGCTGTTCTGAGAGGATGATCAGCCACACTGGGACTGAGACACGGCC

CAAACTCCTACGGGAGGCAGCAGTGGGGAATATTGCACAATGGGGGAAAC

CCTGATGCAGCCATGCCGCGTGTGTGAAGAAGGCCTTCGGGTTGTAAAGC

ACTTTCAGTAGGGAGGAAAGGGTGTAACTTAATACGTTATATCTGTGACG

TTACCTACAGAAGAAGGACCGGCTAACTCCGTGCCAGCAGCCGCGGTAAT

ACGGAGGGTCCGAGCGTTAATCGGAATTACTGGGCGTAAAGCGTGCGCAG

GCGGTTTGTTAAGCGAGATGTGAAAGCCCTGGGCTCAACCTAGGAATAGC

ATTTCGAACTGGCGAACTAGAGTCTTGTAGAGGGGGGTAGAATTCCAGGT

GTAGCGGTGAAATGCGTAGAGATCTGGAGGAATACCGGTGGCGAAGGCGG

CCCCCTGGACAAAGACTGACGCTCATGCACGAAAGCGTGGGGAGCAAACA

GGATTAGATACCCTGGTAGTCCACGCCGTAACGATGTCTACTCGGAGTTT

GGTGTCTTGAACACTGAGCTCTCAAGCTAACGCATTAAGTAGACCGCCTG

GGGAGTACGGCCGCAAGGTTAAAACTTCAAATGAATTGACGGGGGCCCGC

ACAAGCGGTGGAGCATGTGTTATTTCGATGCAACGCGAAGAACCTTAACC

TACTCTTGACATCCAGAAGAATCGCTAGAGATAGCTTAGTGCCTT

>SS54

CTGGTAGTGGGGGACAACGTTTCGAAAGGAACGCTAATACCGCATACGTC

CTACGGGAGAAAGCAGGGGACCTTCGGGCCTTGCGCTATCAGATGAGCCT

AGGTCGGATTAGCTAGTTGGTGGGGTAATGGCTCACCAAGGCGACGATCC

GTAACTGGTCTGAGAGGATGATCAGTCACACTGGAACTGAGACACGGTCC

AGACTCCTACGGGAGGCAGCAGTGGGGAATATTGGACAATGGGCGAAAGC

CTGATCCAGCCATGCCGCGTGTGTGAAGAAGGTCTTCGGATTGTAAAGCA

CTTTAAGTTGGGAGGAAGGGCAGTAAGCGAATACCTTGCTGTTTTGACGT

TACCGACAGAATAAGCACCGGCTAACTCTGTGCCAGCAGCCGCGGTAATA

CAGAGGGTGCAAGCGTTAATCGGAATTACTGGGCGTAAAGCGCGCGTAGG

TGGTTTGTTAAGTTGAATGTGAAAGCCCCGGGCTCAACCTGGGAACTGCA

TCCAAAACTGGCAAGCTAGAGTACAGTAGAGGGTGGTGGAATTTCCTGTG

TAGCGGTGAAAATGCGTAGATATAGGAAGGAACACCAGTGGCGAAGGCGA

CCACCTGGACTGATACTGACACTGAGGTGCGAAAGCGTGGGGAGCAAACA

GGATTAGATACCCTGGTAGTCCACGCCGTAAACGATGTCAACTAGCCGTT

GGAATCCTTGAGATTTTAGTGGCGCAGCTAACGCATTAAGTTGACCGCCT

GGGGAGTACGGCCGCAAGGTTAAACTCAAATGAATTGACGGGGCCCGCAC

AAGCGGTGGAGCATGTGTTACTTCGAAGCAACGCGGAAGACCCTTACTAG

GTCTTTGACATGCCAGAAGAACTTTCCAGAGATGGGAATGGCTGCCTTCG

GAACCTTCTGGAACACAGGTGCTGCATGCCTGTCGTCAGCTCTGTTCTGA

GAAATGTTGGATTAGTTTCCGTTAACGAGCCAACTGGTCCTAGTACAGCC

GTTAATGTTGGACAACCCTCCTAG

>SS55

CGCCGGGGTCCCTTGGGTACGATTTCCCCCAGGTCATGAATCCCGCCGTG

GTAAGCGCCCTCCTTAAGGTAGGGAGAGGGGTAAGGTGAGACTATTCCAG

GGGGGGACGGGGCCCGAGAGGAGGACCCGGGAACGATTCACCGCGACATT

CTGATCCACTATTACTAGCGATTCCGACTTTCACGCAGTCGAGTTGCAGA

CTGCGATCCGGACTACGACTGGTTTTATGGGATGGGGTCCCCCTCGCGGG

GTTGCTTCCCTTTGTACCAACCTTTGTATGACGTGTGTAGCCCCCCCCAT

AAGGGCCATGATGACTTGACGTCATCCCCACCTTCCTCCGGTTTGTCACC

GGCAGTCCCCTTAGAGTGCTCAACTGTACTCGCTAGCAAATGAGGAGGAG

GGTGCTCGTTGCTGGACTTCTTAAAACATCTCTTCACATTTTTTTTTGAC

GACCGCGGTGCAGCAGCTGGCTGATACTTCTTCAAGGACCCAACCATCTC

TGGGAAAGTCCTTTGATGG

>SS56

CTTTTGGTTCGTTGTACGACTTCACCCAGTCACGAACCCCGCCGTGGTAA

GCGCCCTCCTTGCGGTTAGGCTACCTACTTCTGGCGAGACCCGCTCCCAT

GGTGTGACGGGCGGTGTGTACAAGACCCGGGAACGTATTCACCGTGACAT

TCTGATCCACGATTACTAGCGATTCCGACTTCACGCAGTCGAGTTGCAGA

CTGCGATCCGGACTACGACTGGCTTTATGGGATTAGCTCCCCCTCGCGGG

TTGGCAACCCTTTGTACCAGCCATTGTATGACGTGTGTAGCCCCACCTAT

AAGGGCCATGAGGACTTGACGTCATCCCCACCTTCCTCCGGTTTGTCACC

GGCAGTCCCATTAGAGTGCTCAACTGAATGTAGCAACTAATGGCAAGGGT

TGCGCTCGTTGCGGGACTTAACCCAACATCTCACGACACGAGCTGACGAC

AGCCATGCAGCACCTGTGTGCAGGTTCTCTTTCGAGCACCAAACCATCTC

TGGTAAGTTCCTGCCATGTCAAAGGTGGGTAAGGTTTTTCGCGTTGCATC

GAATTAAACCACATCATCCACCGCTTGTGCGGGTCCCCGTCAATTCCTTT

GAGTTTCAACCTTGCGGCCGTACTCCCCAGGCGGTCAACTTCACGCGTTA

GCTTCGTTACTGAGTCAGTTAAGACCCAACAACCAGTTGACATCGTTTAG

GGCGTGGACTACCAGGGTATCTAATCCTGTTTGCTCCCCACGCTTTCGTG

CATGAGCGTCAGTGCAGGCCCAGGGGATTGCCTTCGCCATCGGTGTTCCT

CCGCATATCTACGCATTTCACTGCTACACGCGGAATTCCATCCCCCTCTG

CCGCACTCTAGCTTTGCAGTCACAATGGCAGTTCCCAGGTTGAGCCCGGG

GATTTCACCACTGTCTTACAAAACCGCCTGCGCACGCTTTACGCCCAGTA

ATTCCGATTAACGCTTGCACCCTACGTATTACCGCGGCTGCTGGCACGTA

GTTAGCCGGTGCTTATTCTTACGGTACGTCATGACCCGAGGGTATTAGCC

CAGCCTTTTCGTCGTACAAAAGCAGTTACAACCGAGGTCTCATCTGCACG

CGCATGGCTTGATCAGGCTTTCGCAATGGTCAAATCCCACTGCTGCTTCG

TAGGATCTGTCCGTTCCAGTCCAGGCTGACTTCTCTCGAACAGCTACGGA

ATCGCAGG

>SS57

AGTAGTCCTTTGATAGACTTCACCCAGTCATGAATCACAACCGTGGTAAG

CGCCCTCCTTGAGGTTAGGCTATAACATCTGGCGAGACCAGTTCCAAGGG

GGGGAGGGGGGGGGTGTACAAGACCCGGGAACGTATTCACCGTGACATTC

TGATCCACGATTACTAGCGATTCCGACTTCACGGAGTCGAGTTGCAGACT

GCGATCCGGACTACGACTGGTTTTATGGGATTAGCTCCCCCTCGCGGGTT

GGCAACCCTTTGTACCAGCCCATTGTATGACGTGTGTAGCCCTACCTATA

AGGGCCATGAGGACTTGACGTCATCCCCACCTTCCTCCGGTTTGTCACCG

GCAGTCTCATTAGAGTGCTCAACTGAATGTATGAACTAATGGGAAGGGGT

GCGCTCGTTGCGGGACTTATACCACCATCTTTCGACACGAGCTGACAACA

GCCATGCAGCACCTGTGTGCAGGATCTCTTTCGAGCACCAAACCATCTCT

GGTAAGTTCCTGCCATGTCAAAAGTGGGTAAGGTTTTTCGCGTTGCATCG

AATTAAACCACATGCATCCACCGCTTGTGCGGGTCCCCGTCAATTCATTT

GAGTTTCAACCTTGCGGCCGTACTCCCCAGGCGGTCGACTTCACGCGTTA

GCTTCGGTACTGAGTCCTCTAAGACCCAACAACCAATTGACATCGTTTTA

GGGCGTGGGACTACCAGGGTATCTAATCCTGTGTTGCTCCCCACGCTTTC

GTGCATGAGCGTCAGTGCTTTGCCCAGGGGATTGCCTTCGCCATCGGTGT

TCCTCCGCATATCTACGCATTTCACTGCTACACGCGGGAATTCTATCCCC

CTCTGCCACACTCTAGCTTGGCAGTCACAGTGGGCAGTTCCCAGGGTTGA

GCCCGGGGATTTCACCATCTGTCTTACCAAAACCGCCTGCGCACGGCTTT

ACGCCCAGTAATTTCCGATTAACGCTTGCACCCTACGTATTACCGCGGCT

GCTGCACGAGTTAGCCGGTGCTTAATTCTTACGAGTAACGTCATGTAGCT

GAGGATAATAGCCTCAAGGCCTTTCGTCCTCGGACGAAAGCAGTTACACC

CGGAAGGGCTTCCTCATAGCACGCGGCATTGCCTGAATCAGCTTGCGCCC

AATGGTCCAAAATTTCCCCTGCTGCTTCGTTAAGGATCTGGACGTTCAGT

CCAGGGGCTGCATCCTCTCGACGCTTAGCGAATCGCCTAG

>SS58

AGGGGGAGAAGGGCGGGGCGGGGAGACGTGACCCATGGGGTGACGGGGGA

GGGGGGAGGACCCGGGAACGTATTCACCGTGACATTCTGATCCTTTATAC

TAGCGATTCCGACTTCACGCATTCCAGTTGCAGACTGCGATCCGGACTAC

GACTGGCTTTATGGGGTTAGGTCGCCCTCGCGGGTTGGCAACCTTTTGTA

CCAGCCATTGTATGACGTGTGTAGCCCCACCTATAAGGGCCATGAGGACT

TGACTTCATCCCCACCTTCCTCCGGTTTGTCACCGGCAGTCCCATTAAAG

TGCTCAACTGAATGTAGCAACTAATGGCAAGGGCTGCGCTCGGTGCGGGA

CGCACCCTACATCTCACGACACGATTTGTCGACAGCCATGCAGTGGCGGT

GGGCAGGTTCTCTTTCCAGCACCTAACCATCCCTGGTAAGTTCCTGCCAT

GTCAAAGGGGGGTAAGGTTTTTCGCGTTGCATCGAATTAACCACTCATCC

ACCGGCTTGTGCGGGCCCCCGTCAATTCCTTTGAGTTTCAACCTTGCGGC

CCTACTCCCAGGGGGTCAACTTCACGCGTTAGCTTCATTACTGACCTCTT

AAGACCCAACATCCAA

>SS59

TACATTGGCAAGGTCGAAGCGGGCAGCGCGTAAAGCAGATGGCGACTTTT

GCCGGACAGGGGAGGAAGGGTCGTAACGTGTGGGGACTGCGCAATCAAGG

GGGAAAGAGGTGGAAACGACTGCGATACCGCATACTGCCTACGGGGGACT

GAGGGGACTTTGCGGCCTTTCGCGATTGGATGAAATCAGGTGGGATTAGG

TGGATGGGGGGGTAATGGCTGACCTCGGCGACTATCCCTAGATGGTCTGA

GAGGATGATGGGACTGACTGGAACTGAAAACCCCTCCAGACTGCTACAGG

AGGCAATTTTGGAGAATGTTGCAAAACGTGGGAAACCCTGATGCAGCCAT

GCATCGTGGGCCTCGAATTGCTAACTGCTGTAGAGCAGTTCCAACAACCT

GGAAAGAACGGCGCCGGGTACCGACGAACTGGGACGATACACCCACAGTA

AGCTACGGGTAAGTCCCTGCCGTCATACGCAGGGAGAAGGAGGGTGTCAG

AATTAATGGGAATTACGCGGGGTCAGGCAGATTCATGAGACTGGAGTGAA

TTCATGGGAAAGCCCCGGGCTACAGCCGGTAATTCCGTTTAACACTGTCC

CGCTAGAGTCTTGTAGAATTCGGTAGAATTCCAGGAGTAGCGTAGATATG

CGCAGAGATCCCGATGACTACCGCTGGCCACTGCGGGCCCCCTGGACACT

CACTGACGATTACGTGCGGAGCCATGAGGATCAAACACGCTGAGAGCCCC

TGGTAGTCCCGATCGCAAATGATGGTTGGTTGGATACTGTCTCCTTACAA

CGGTTTTCTCGACCGTAGCCCCCCAGTCGAACTCCTGCGGAATGCCTGAC

ACCATGAAGCAATTTCAAGGTGACTTGCCCGGGGGCGCCGCATAACGGGG

GGTAGAATTGTAGGTCTAAATTCGAATGAACCCTCACAAACATTCTTAAC

CTGGCCTCGGAACTATGCCCTGAGGATGCCTGTGAGAGATTCGG

>SS60

GCGGGGGTTCTGGATACGAATTTCCCCCAGTCATGAATCACAAAGTGGTA

AGCGCCCTCCTGAAGGTTAGGAAAAGGCTTGCGGCAGCCTTCCCATGGGG

TGAAGGTGGCCGAAAGGGGGCCCGGGAAGTATTCCCGCGCATTCTGATCT

ACTTTTTACTAGCGATTCCGACTTCATGCATTCGAGTTGCAGACTGCGAT

CCGGACTACGATCGGTTTTTGAGGGGGGCTGGCTCTCGCGAGTTGCTTCC

CTTTGTACTGCCATTGTAGCACGTGTGTAGCCCAGCCGTAAGGGCCATGA

TGACTTGACGTCATCCCCACCTTCCTCCGGTTTGTCACCGGCAGTCTCCT

TAGAGTGCCCAACATTACTCGCTGGCTACTAAGGACAAGGGTTCCGCTCG

TTGCGGTACTTTACCCAACATTTCACAACAC

>SS61

GGATAACTACTCGAAAGAGTAGCTAATACCGCATGAGATCTACGGATGAA

AGCAGGGGACCTTCGGGCCTTGTGCTACTAGAGCGGCTGATGGCAGATTA

GGTAGTTGGTGGGGTAAAGGCTTACCAAGCCTGCGATCTGTAGCTGGTCT

GAGAGGACGACCAGCCACACTGGGACTGAGACACGGCCCAGACTCCTACG

GGAGGCAGCAGTGGGGAATTTTGGACAATGGGCGAAAGCCTGATCCAGCA

ATGCCGCGTGCAGGATGAAGGCCCTCGGGTTGTAAACTGCTTTTGTACGG

AACGAAAAGCCTGGGGCTAATATCCCCGGGTCATGACGGTACCGTAAGAA

TAAGCACCGGCTAACTACGTGCCAGCAGCCGCGGTAATACGTAGGGTGCA

AGCGTTAATCGGAATTACTGGGCGTAAAGCGTGCGCAGGCGGTTTTGTAA

GACAGTGGTGAAATCCCCGGGCTCAACCTGGGAACTGCCATTGTGACTGC

AAAGCTAGAGTGCGGCAGAGGGGGATGGAATTCCGCGTGTAGCAGTGAAA

TGCGTAGATATGCGGAGGAACACCGATGGCGAAGGCAATCCCCTGGGCCT

GCACTGACGCTCATGCACGAAAGCGTGGGGAGCAAACAGGATTAGATACC

CTGGTAGTCCACGCCCTAAACGATGTCAACTGGTTGTTGGGTCTTAACTG

ACTCAGTAACGAAGCTAACGCGTGAAGTTGACCGCCTGGGGAGTACGGCC

GCAAGGTTGAAACTCAAAGGAATTTGACGGGGACTCGCACAAGCGGTGGA

TGATGTGGTTTAATTTCGATGCTACGCGAAAAACCTTACCCACCTTTTGA

CATGGCAGAACTTACCAGAGATGTTTGGTGCTCGAAAGAAGACTGCACAC

AGTGCTGCATGCTGTCGTCAGCTCGTGTCGTGAGATGTGGGTAAGTCCCG

CACGACGCACCCTGCATAGTGCTACTTCAGTGAGCATCTATGGACTGCGT

GACAACCGAGGAAGTGGGATGACGTCAGTCCTCCATGCCCTTAG

>SS62

GGGGATAACTACTCCAAAGAGTAGCTAATACCGCATGAGATCTACGGATG

AAAGCAGGGGACCTTCGGGCCTTGTGCTACTAGAGCGGCTGATGGCAGAT

TAGGTAGTTGGTGGGGTAAAGGCTTACCAAGCCTGCGATCTGTAGCTGGT

CTGAGAGGACGACCAGCCACACTGGGACTGAGACACGGCCCAGACTCCTA

CGGGAGGCAGCAGTGGGGAATTTTGGACAATGGGCGAAAGCCTGATCCAG

CAATGCCGCGTGCAGGATGAAGGCCCTCGGGTTGTAAACTGCTTTTGTAC

GGAACGAAAAGCCTGGGGCTAATATCCCCGGGTCATGACGGTACCTTAAG

AATAAGCACCGGCTAACTACGTGCCAGCAGCCGCGGTAATACGTAGGGTG

CAAGCGTTAATCGGAATTACTGGGCGTAAAGCGTGCGCAGGCGGTTTTGT

AAGACAGTGGTGAAATCCCCGGGCTCAACCTGGGAACTGCCATTGTGACT

GCAAAGCTAGAGTGCGGGCAGAGGGGGGATGGAATTCCGCGTGTAGCAGT

GAAATGCGTAGATATGCGGAGGAACACCGATGGCGAAGGCAATCCCCTGG

GCCTGCACTGACGCTCATGCACGAAAGCGTGGGGAGCAACAGGATTAGAT

ACCCTGGTAGTCTACGCCTAACGATGTCAACTGGTTGTGGGGTCTTAACT

GACTCATAACAAAGCTAACGCGTGAAGTTGACCGCCTGGGAGTACGGCCG

CAAGGTTGAAACTCAAAGAATTGACGGGACCGCACAGCGCGGATGATGTG

GTTTATTCATGCACGCTAATAACCTTACCACCTTTGACATGGCGGAACTA

ACCGAATGGTTGGTGGCTCAAAAAAAACCTGCACACAGAGGCTGCATGGC

TTGTCGTCAGCTTCTGGT

>SS63

CTTACTATGGCGAGGGGGGGGAGGTAAAGAATGAGAGTGAGCCTGCCTGA

GGGAGAAAAGACCTACGGGGAACGGTACCTAATAATATCTGAGGAGGAGA

ACGGAGGAGATGTACGCTTCTTGCGCTTGCAGATGAATATGGGCCAATGG

GACTATGGGGGGGGAGAAGGTTACGCTCACGAGGAGATGGAGCTCTGGTG

GGAGTGAGAGGATCCACACTGTCACTGAGACACAGACCCGATACCCACTC

CTGGGGGAGGTGGGGAAGTGTGTAATTTGTGCGAAAGCCTGATCCTGAAG

TGCCGCTTGCACGAGGATGGCCTACGGGTTGTAAACTGCTTTTTTTTTCA

ACGAAGAGGAATCTCCTAATGTTAAAAGCCCTTGACTTTTTTTTTACACT

CAGCAGAGGCTAACTCGGACTCCGTGGCCGCGCCACACTTATGCTGAGAG

CGTTAATCGGAATTTCAGGGCTCGGGGGTGCGCGTCGGGGTGTGTTACAA

ATTATGAATTCAAATCCTCCTCCTCGGAACTGCATTTGTGTATGCATGTG

TCTGTACGATTGTATGTGGATGGAGGTACACCCTGTGGAGTGGCGTGCTA

ATAATACAGAGCAGGACCAATAGCGAAGGCGATCACCTGCCCCTGTACTA

ACACTCATGCTCTGGAGCGCGGGGGGGAGAAAACAATCATAAAATCCTAT

GCTCGTCGCCCCCGCAGATGACGACTGCTACTTGGTGGTTACCTTTTTAT

TTTACGACGTCAGCACATGAAGTAGATCGACCGGCCGCGTGCGGACGGCA

GGCTTGAAGTTAAAACAATTAATTGGTTACCGGCCCCCCCCGTGGTGGAG

CGGGTTGTTTCTTTGTACCCACCACACCAACCCCACCTGTGCTTCGCTGA

AATTACATTATATAGAAGATTGGTTGCATAGACACTGACTACAAGAGCGC

GCTGCGGTGGTCTCATCTCGTCTGGTAGAGTTAGGTTAAGTTCACCCACC

AGGACCAACCCCATTCCTTATTTTTTTTTACTTTCCAGTTGTATCGGCTC

GCGAAAGCCTTCTGGGGAAAAGTTGCGGAGACCTGAA

>SS64

GCGCCCTCCTTGCGGTTAGGCTACCTACTTCTGGCGAGACCCGCTCCCAT

GGTGTGACGGGCGGTGTGTACAAGACCCGGGAACGTATTCACCGTGACAT

TCTGATCCACGATTACTAGCGATTCCGACTTCACGCAGTCGAGTTGCAGA

CTGCGATCCGGACTACGACTGGCTTTATGGGATTAGCTCCCCCTCGCGGG

TTGGCAACCCTTTGTACCAGCCATTGTATGACGTGTGTAGCCCCACCTAT

AAGGGCCATGAGGACTTGACGTCATCCCCACCTTCCTCCGGTTTGTCACC

GGCAGTCCCATTAGAGTGCTCAACTGAATGTAGCAACTAATGGCAAGGGT

TGCGCTCGTTGCGGGACTTAACCCAACATCTCACGACACGAGCTGACGAC

AGCCATGCAGCACCTGTGTGCAGGTTCTCTTTCGAGCACCAAACCATCTC

TGGTAAGTTCCTGCCATGTCAAAGGTGGGTAAGGTTTTTCGCGTTGCATC

GAATTAAACCACATCATCCACCGCTTGTGCGGGTCCCCGTCAATTCCTTT

GAGTTTCAACCTTGCGGCCGTACTCCCCAGGCGGTCAACTTCACGCGTTA

GCTTCGTTACTGAGTCAGTTAAGACCCAACAACCAGTTGACATCGTTTAG

GGCGTGGACTACCAGGGTATCTAATCCTGTTTGCTCCCCACGCTTTCGTG

CATGAGCGTCAGTGCAGGCCCAGGGGATTGCCTTCGCCATCGGTGTTCCT

CCGCATATCTACGCATTTCACTGCTACACGCGGAATTCCATCCCCCTCTG

CCGCACTCTAGCTTTGCAGTCACAATGGCAGTTCCCAGGTTGAGCCCGGG

GATTTCACCACTGTCTTACAAAACCGCCTGCGCACGCTTTACGCCCAGTA

ATTTCCGATTAACGCTTGCACCCTACGTATTACCGCGGCTGCTGGCAC

>SS65

GCCCGGGGGGTCGTTGTTACAACTTCACCCCGGTTTTGATCACAAAGTGG

TAAGCGCCCTCCGGAAGGTTAAGCAAAAAGGGGAGGGGGCACCCACTCCC

ATGGTGTGACGGGGGCGAGTTAAAGGCCCGGGAACGTATTCACCGTGGCA

TTCTGATCCACGATTACTAGCGATTCCGACTTCACGGAGTCTAGTTGCAG

ACTCCGATCCGGACTACGACATACTTTATGAGGGGCGCGTGCTCTCGCGA

GGTCGCTTCTCTTTGTATATGCCATTGTAGCACGTGTGTAGCCCTACTCG

TAAGGGCCTTGATGACTTGACGTCATCCCCACCTTCCTCCAGTTTATCAC

TGGCAGTCTCCTTTGAGTTCCCGGCCGAACCGCTGGCAACAAAGGTTAAG

TGTTGCGCTCGTTGCGGGATTTATTTTAACATTTCACAATTTTTTTTTGA

CGACAGCCATGGGGGGGGTGACTCAAAG

>SS66

AAAGCAACTAAAAAAGGAAGGAGGGGGGGGAACCCATCCATGGGGTGACG

GGCTTCCGAGGGAGGGGCCCGGGAACGTATTCCCGCGACATTCTGATTCT

CTATTACTAGCGATTTCGACTTCACGCATTTTAGTTGCAGACTGCGATCC

GGATTACGATCGGCTTTGTGGGGTGAGCTCCACCTCGCGACTTGACAACC

CTCTGTACCGACCATTGTAGCACGTGTGTAGCCCAGGCCGTAAGGGCCAT

GATGACTTGACCTCCTCCCCACCTTCCTCCGGTTTGTCACCGGCAGTTTC

CTTAGAGTGCCCACCATAACGTGCTGTTAACTAAGGACAAGGGTTGCGCT

CGTTTCGGGTTTTAACCCAACATCTCACGACTTTTGTTGACGACGCCATG

GAGCACCTGTGGCAAGTTCCCGAAGGCACCTATCCATCTCTAGAAAGTTT

TCTGCATGTCAAGGCCTGGGAAGGTTCTTGGCGTTCTTCGAATTAAACCA

GATGCTCCACCGCTTGTGCTGGCCCCCGTCCTTCTTTTGTGTTTAATCTT

GCGGTCGTACTCCCCGGCGGTCAATTTAATGCGT

>SS67

ATCCGCGATTACTAGCGATTCCAGCTTCATGTAGGCAAGTTGCAGCCTAC

AATCCGAACTGAAAACGGTTTTATGAGATTAGCTCCACCTCGCGGTCTTG

CAGCTCTTTGTACCGTCCATTGTAGCACGTGTGTAGCCCAGGTCATAAGG

GGCATGATGATTTGACGTCATCCCCACCTTCCTCCGGTTTGTCACCGGCA

GTCACCTTAGAGTGCCCAACTTAATGATGGCAACTAAGATCAAGGGTTGC

GCTCGTTGCGGGACTTAACCCAACATCTCACGACACGAGCTGACGACAAC

CATGCACCACCTGTCACTCTGCTCCCGAAGGAGAAGCCCTATCTCTAGGG

TTTTCAGAGGATGTCAAGACCTGGTAAGGTTCTTCGCGTTGCTTCGAATT

AAACCACATGCTCCACCGCTTGTGCGGGCCCCCGTCAATTCCTTTGAGTT

TCAGCCTTGCGGCCGTACTCCCCAGGCGGAGTGCTTAATGCGTTAACTTC

AGCACTAAAAGGGCGGAAACCCTCTAACACTTAGCACTCATCGTTTACGG

CGTGGACTACCAGGGTATCTAATCCTGTTTGCTCCCCACGCTTTCGCGCC

TCAGTGTCAGTTACAGACCAGAGAGTCGCCTTCGCCACTGGTGTTCCTCC

ATATCTCTACGCATTTCACCGCTACACATGGAAATTCCACTTTCCTCTTC

TGCACTCAAGTCTGCCAGTTTCCAATGACCCTCCACGGTTGAGCCGTGGC

TTTCACATCAGACTGAAGAAACCACCTGCGCGCGCTTTACGCCCAATAAT

TCCGAGATAACGCTTGACACCTTACGTATTACGCCGCTGCTGCACGTAGT

TAGCCGTGCTTTTCTGGTTAGGTACCGTCAAGTGCAGCTTATTACACCTA

GGACTTGGTTCCTTCCCTAACAACGGAGTTTATACGACCGGAGAGCCTTT

CTTCGACTCCGCGCGATGGTTTGTGTCTCTCGGACTTCCGTCTATTGCGA

GATTCCCTTTCTGTCTGCTTCGTAAGGATCTTAGCCG

>SS68

ACATTCTGATCTACGATTACTAGCGATTCCGACTTCATGGAGTCGAGTTG

CAGACTCCAATCCGGACTACGACATACTTTATGAGGTCCGCTTGCTCTCG

CGAGTTGGCTTCTCTTTGTATATGCCATTGTAGCACGTGTGTAGCCCTAC

TCGTAAGGGCCATGATGACTTGACGTCATCCCCACCTTCCTCCGGTTTAT

CACCGGCAGTCTCCTTTGAGTTCCCACCATTACGTGCTGGCAACAAAGGA

TAAGGGTTGCGCTCGTTGCGGGACTTAACCCAACATTTCACAACTCGTTT

CTGACGACAGCCATGCAGCACCTGTCTCACGGTTCCCGAAGGCACTAAGC

CATCTCTGGCGAATTCCGTGGATGTCAAGAGTAGGTAAGGTTCTTCGCGT

TGCATCGAATTAAACCACATGCTCCACCGCTTGTGCGGGCCCCCGTCAAT

TCATTTGAGTTTTAACCTTGCGGCCGTACTCCCCAGGCGGTCGACTTAAC

GCGTTAGCTCCGGAAGCCACACCTCAAGGGCACAACCTCCTAGTCGACAT

CGTTTACAGCGTGGACTACCAGGGTATCTAATCCTGTTTGCTCCCCACGC

TTTCGCACCTTGAGCGTCAGTCTTTGTCCAGGGGGCCGCCTTCGCCACCG

GTATTCCTCCAGATCTCTACGCATTTCACCGCTACACCTGGAATTCTACC

CCCCTCTACAAGACTCTAGCTTTGCCAGTTTCAATGCAGTTCCCACGTTA

AGCGCGGGGATTTCCATCTGACTTACAACCCCCTGCGTGCGCTTTACGCC

CAGTAATTCGATTAACGCTTGCACCCCTCCGTATTACTGCGG

>SS69

GCATTCTGATCTACGATTACTAGCGATTCCGACTTCATGGAGTCGAGTTG

CAGACTCCAATCCGGACTACGACATACTTTATGAGGTCCGCTTGCTCTCG

CGAGTTCGCTTCTCTTTGTATATGCCATTGTAGCACGTGTGTAGCCCTAC

TCGTAAGGGCCATGATGACTTGACGTCATCCCCACCTTCCTCCGGTTTAT

CACCGGCAGTCTCCTTTGAGTTCCCACCATTACGTGCTGGCAACAAAGGA

TAAGGGTTGCGCTCGTTGCGGGACTTAACCCAACATTTCACAATACGAGC

TGACGACAGCCATGCAGCACCTGTCTCACGGTTCCCGAAGGCACTAAGCC

ATCTCTGGCGAATTCCGTGGATGTCAAGAGTAGGTAAGGTTCTTCGCGTT

GCATCGAATTAAACCACATGCTCCACCGCTTGTGCGGGCCCCCGTCAATT

CATTTGAGTTTTAACCTTGCGGCCGTACTCCCCAGGCGGTCGACTTAACG

CGTTAGCTCCGGAAGCCACGCCTCAAGGGCACAACCTCCAAGTCGACATC

GTTTACAGCGTGGACTACCAGGGTATCTAATCCTGTTTGCTCCCCACGCT

TTCGCACCTGAGCGTCAGTCTTTGTCCAGGGGGGCCGCCTTCGCCACCGG

TATTCCTCCAGATCTCTACGCATTTCACCGCTACACCTGGAATTCTACCC

CCCTCTACAAGACTCTAGCTTGCCAGTTTCAAATGCAGTTCCCACGTTAA

GCGCGGGGATTTCACATCTGACTTAACAACCGCCTGCGTGCGCTTTACGC

CCAGTAATTCCGATTAACGCTTGCACCCTCCGTATTACCGCGCTGCTGGC

ACGGAGTTAGGCCGGTGCTTCTTCTGGCGAGTAACGTCCATCACCACACG

TATAAAGGGTGGATGCCTTTCCTCCTTCGCTGAAAGTGCTTTACAACC

>SS70

GTGGTAAGCGCCCTCCCGAAGGTTAAGCTACTTACTTCTTTTGCAACCCA

CTCCCATGGTGTGACGGGCGGTGTGTACAAGGCCCGGGAACGTATTCACC

GTAGCATTCTGATCTACGATTACTAGCGATTCCGACTTCATGGAGTCGAG

TTGCAGACTCCAATCCGGACTACGACGTACTTTATGAGGTCCGCTTGCTC

TCGCGAGGTCGCTTCTCTTTGTATACGCCATTGTAGCACGTGTGTAGCCC

TACTCGTAAGGGCCATGATGACTTGACGTCATCCCCACCTTCCTCCAGTT

TATCACTGGCAGTCTCCTTTGAGTTCCCGGCCGAACCGCTGGCAACAAAG

GATAAGGGTTGCGCTCGTTGCGGGACTTAACCCAACATTTCACAACACGA

GCTGACGACAGCCATGCAGCACCTGGCTCACGGTTCCCGAAGGGACTAAA

GCATCTCTGCTAAATTCCGTGGATGTCAAGAGTAGGTAAGGGTCTTCGCG

TTGCATCGAATTAAACCACATGCTCCACCGCTTGTGCGGGCCCCCGTCAA

TTCATTTGAGTTTTAACCTTGCGGCCGTACTCCCCAGGCGGTCGACTTAA

CGCGTTAGCTCCGGAAGCCACTCCTCAAGGGAACAACCTCCAAGTCGACA

TCGTTTACGGCGTGGACTACCAGGGTATCTAATCCTGTTTGCTCCCCACG

CTTTCGCACCTGAGCGTCAGTCTTTGTCCAGGGGGCCGCCTTCGCCACCG

GTATTCCTCCAGATCTCTACGCATTTCACCGCTACACCTGGAAATCTACC

CCCCTCTACAAGACTCTAGCCTGGCAGTTTCGAATGCAGTTCCCAGGGTT

GAGCCCGGGGATTTCACCATCCTGACTTGACAGACCGGCCTGC

>SS71

TTTGTGGAAGGCCTCCCGTGCGCGTCGAGCGGTAGCGGGGAAGTAGCTTG

CTACTTTTGCCGGCGAGCGGGCACGGAGGAGTAATGTCTGGCAAAGTGCG

AAGTCCAGGGGCATAACAGTTGGAAACGACTGCGAATACCGCATACGCCC

TACGCGGGAAAGCAGGGCACCTTCGGGACTTGCGCGATTGTATATGCCCG

GGTGGGATTAGCTTGTTGGTGAGGCAATGGCTCACCCCGGCGACGATCCC

TAGCTGGGCTGAGAGGATGATCAGCCACACTGGAACTGACCCAGGTTCAA

CTCTTCTACGGGAGGCCGCAGTGGGGACTATTGCACAATGGCGGAAACCC

TGATGCAGCCATGCCGCGTGTGCTATTAAGGCCTTCAGGCTCTTAAGCAC

TTTCGGCGAGGAGGAAAGGTTATAACCTAATACGTTTCTTTAAAAATTTT

TACTCGCCAAAGAGGCACATGGCTAACTCCGTGCCAGCAGACGCGGTAAT

ACGGGAGGGTGAAGCGTTAAACGGAATTACTGGGCGTAAAGCGCACGCAG

GCGGTTGGATAAGTTAGATGTGAAAGCTCCCGGGCTCAACCTGGGAATTG

CATTTAAAACTGTCCGTCTAGAGTCTTGTGTGAGGGGTGTAGAATTCCTC

GTTGAACGGGTGAAATGCTTTAGAGATC

>SS72

GGGGCGGCATCCTCTACGTGCAAGTCGAGCGGTCGCGGGGAGTAGCTTGC

TACTTTTGCCGGCGAGCGGCGCAAGGATGAGTCCAGTCTGGCAAAGTGCG

CAGTCCAGGGGCTTAACAGTGGGAAACGACTGCGAATACCGCATACGCCC

TACGCTGGAAAGCAGGGCACCTTCGGGCCTTGCGCGATTGTATATGCCCG

GGTGGCATTAGCTTGTTGAAGAGGTAATGGCTGTCCCAGGCGACGATCCC

CATCTGGGCTGAGAGGATGATCCGCCCCACTGATCCTAACCCTGGACCAA

CTCTTCAACGGGAGGCAGCATTGGGGAATATTGCCCAATGGCTGAACCCC

TGATGCAGCCATGCTGAGTGTGTTATTCTGGCCTTCGGGCTGTTAAGCAT

TTTAAGAGAGGAGGAAAGGTTAAAAAATTAACGGGTGGTTTAAAAAAAAA

AATTTTCGGCACGAGTCGCAGGCTAACCTCCGTGCCACCAGAATACGGAA

ATACGGAGGGTGCATCCGTTATTCTGAATTACTGGGCCGTAAAGCGCACG

CAGTTAGTTG

>SS73

TCTTTCCGAATCCTATCGTGCGAGTCGAGCGGTCGACAAGAGCGTGCTTT

ACCTTTCCGAGTAGGTCGGGTGAGAAAAGCTCATCTGTTTGCCTGGTAGT

GGGGGACAACGGCTTTTAGGAACGCTAATACTGCGTACTTACTATGGGAT

AAAAAAGGTGATCTTCCGGCCTTGCGCTATTGATAGGCATAACTTCGAAT

TAGCTAGGTTGTGAGAAGAGAATCACCAAGGCGGCGGTCCGTCACTCTTT

TGTAAGGATGTCACCCCCACTCAATCTGAGCCATGATCAACCTCACAACC

>SS74

CCGTTCGGGTTCCTACAATGCGCTCGAGCGGTCGCCAAAGTGAGCCATTA

CCTCACGTACTAGGTAGTCCGATGGGGACACATCTGATGGCATGAGGGAG

GAAGAGGGACACTTTGGTCTTGCGACGTTATGCGGGATTAGCGACCGTTT

ACAGAAGTGATCCCCCTCCATCAGGCAGTTTCCCAGACATTAGCCACCCG

TCCGACGCACAACACGAGAGAAAAAGCCACCTGTGGGACAATACCTATCG

GGGGTCAGAGAATAGCCCCCCTCACTGTGTCTCAGACACGATCTCTCTCT

CT

>SS75

AAACGCCCTCCCGAAGGTTAAGCTATCTACTTCTGGTGCAACCCACTCCC

ATGGTGTGACGGGCGGTGTGTACAAGGCCCGGGAACGTATTCACCGCAAC

ATTCTGATTTGCGATTACTAGCGATTCCGACTTCACGGAGTCGAGTTGCA

GACTCCGATCCGGACTACGACGCGCTTTTTGGGATTCGCTCACTATCGCT

AGCTTGCAGCCCTCTGTACGCGCCATTGTAGCACGTGTGTAGCCCTGGCC

GTAAGGGCCATGATGACTTGACGTCATCCCCACCTTCCTCCGGTTTATCA

CCGGCAGTCTCCCTTGAGTTCCCACCATTACGTGCTGGCAACAAAGGACA

GGGGTTGCGCTCGTTGCGGGACTTAACCCAACATCTCACGACACGAGCTG

ACGACAGCCATGCAGCACCTGTGTTCTGATTCCCGAAGGCACTCCCGCAT

CTCTACAGGATTCCAGACATGTCAAGGCCAGGTAAGGTTCTTCGCGTTGC

ATCGAATTAAACCACATGCTCCACCGCTTGTGCGGGCCCCCGTCAATTCA

TTTGAGTTTTAACCTTGCGGCCGTACTCCCCAGGCGGTCGATTTAACGCG

TTAGCTCCGGAAGCCACGTCTCAAGGACACAGCCTCCAAATCGACATCGT

TTACGGCGTGGACTACCAGGGTATCTAATCCTGTTTGCTCCCCACGCTTT

CGCACCTGAGCGTCAGTCTTTGTCCAGGGGGCCGCCTTCGCCACCGGTAT

TCCTCCAGATCTCTACGCATTTCACCGCTACACCTGGAATTCTACCCCCC

TCTACAAGACTCTAGCTGGACAGTTTTAAATGCAATTCCCAGGTTGAGCC

CGGGGCTTTCACATCTAACTTATCCAACCGCCTGCGTGCGCTTTACGCCC

AGTAATTCCGATTAACGCTTGCACCCTCCGTATTACCGCGGCTGCTGGCA

CGGAGTTAGCCGGTGCTTCTTCTGCGAGTAACGTCACAGCTGATACGTAT

TAGGCATCAACCTTCTCTCGCTGAAGTGCTTACAACCGAATGCTTCTCCA

ACACGCGCATGACTGCATCAGGCTTCCTCATGATGCAATAT

>SS76

GGTAAGCGCCCTCCTTGCGGTTAGGCTACCTACTTCTGGCGAGACCCGCT

CCCATGGTGTGACGGGCGGTGTGTACAAGACCCGGGAACGTATTCACCGT

GACATTCTGATCCACGATTACTAGCGATTCCGACTTCACGCAGTCGAGTT

GCAGACTGCGATCCGGACTACGACTGGCTTTATGGGATTAGCTCCCCCTC

GCGGGTTGGCAACCCTTTGTACCAGCCATTGTATGACGTGTGTAGCCCCA

CCTATAAGGGCCATGAGGACTTGACGTCATCCCCACCTTCCTCCGGTTTG

TCACCGGCAGTCCCATTAGAGTGCTCAACTGAATGTAGCAACTAATGGCA

AGGGTTGCGCTCGTTGCGGGACTTAACCCAACATCTCACGACACGAGCTG

ACGACAGCCATGCAGCACCTGTGTGCAGGTTCTCTTTCGAGCACCAAACC

ATCTCTGGTAAGTTCCTGCCATGTCAAAGGTGGGTAAGGTTTTTCGCGTT

GCATCGAATTAAACCACATCATCCACCGCTTGTGCGGGTCCCCGTCAATT

CCTTTGAGTTTCAACCTTGCGGCCGTACTCCCCAGGCGGTCAACTTCACG

CGTTAGCTTCGTTACTGAGTCAGTTAAGACCCAACAACCAGTTGACATCG

TTTAGGGCGTGGACTACCAGGGTATCTAATCCTGTTTGCTCCCCACGCTT

TCGTGCATGAGCGTCAGTGCAGGCCCAGGGGATTGCCTTCGCCATCGGTG

TTCCTCCGCATATCTACGCATTTCACTGCTACACGCGGAATTCCATCCCC

CTCTGCCGCACTCTAGCTTTGCAGTCACAATGGCAGTTCCCAGGTTGAGC

CCGGGGATTTCACCACTGTCTTACAAAACCGCCTGCGCACGCTTTACGCC

CAGTAATTCCGATTAACGCTTGCACCCTACGTATTACCGCGGCTGCTGGC

ACGTAGTTAGGCCGGTGCTTATTCTTACGGTACCGTCATGACCCGGGATA

TTAAGCCCCAGGCTTTCGTTTCCGTACAAAAGCAGTTTACTACCTCGAGG

GCCTTCATCTGCACGCGCATGCCTGATCAAGGCTTTC

>SS77

CGGAGTGGGCCTTTTTTCAACTTCACCCAGTCACGAACCCCGCCGTGGTA

AAGCGCCCTCCTTGCGGTTAGGCTAAAACAGGAAGGCGAGACCCGCTCCC

ATGGTGTGACGGGCGGTGTGTACAAGACCCGGGAACGTATTCACCGTGAC

ATTCTGATCCACGATTACTAGCGATTCCGACTTCACGCAGTCGAGTTGCA

GACTGCGATCCGGACTACGACTGGCTTTATGGGATTAGCTCCCCCTCGCG

GGTTGGCAACCCTTTGTACCAGCCATTGTATGACGTGTGTAGCCCCACCT

ATAAGGGCCATGAGGACTTGACGTCATCCCCACCTTCCTCCGGTTTGTCA

CCGGCAGTCCCATTAGAGTGCTCAACTGAATGTAGCAACTAATGGCGAGG

GTTGCGCTCGTTGCGGGACTTAACCCTACATCTCACGACACGAGCTGACG

ACAGCCATGCAGCACCTGTGTGCAGGTTCTCTTTCGAGCACCAAACCATC

TCTGGTAAGTTCCTGCCATGTCAAAGGTGGGTAAGGTTTTTCGCGTTGCA

TCGAATTAAACCACATCATCCACCGCTTGTGCGGGTCCCCGTCAATTCCT

TTGAGTTTCAACCTTGCGGCCGTACTCCCCAGGCGGGTCAACTTCACGCG

TTAGCTTCGTTACTGAGTCAGTTAAGACTTCAACAACCAGTTGACATCGT

TTAGGGCGTGGACTACCAGGGTATCTAATCCTGTGTTGCTCCCCACGCTT

TCGTGCATGAGCGTCAGTGCAGGCCCAGGGGGATTGCCTTCGCCATCTGT

GTTCCTCCGCATATCTACGCATTTCACTGCTACACGCGGAATTCCATCCC

CCTCTGCCGCACTCTAGCTTTGCAGTCACAATGACCAGTTCCCAAGTTGA

GCCCGGGGATTTCACCACTGTCTTACAAAACCGCCTGCGGCACGCTTTAC

CGCCAAGTAATATCGAATAACCGCTTGCACCCTTACTTATTACCGCCGGC

CTGCTGGCACGTAAATTAAGCCGGTGCTTATTCCTAACGTAACCGTCCTG

AACCCAGGGATATTAGCCCCAGGGCTTTTTCGTTCCGTACATAAGCAGGT

TTAACAACCTCGGACCTTTCATTCCTGCACGCGGCAATGGCT

>SS78

ACCTTTCCTGCCAACCGATCCATCTGGACACATCCGATGGCGTCTGCCTG

GTAGAGGGGGACTTTACTGGAAACGGAGCTAATACCGCATGACCTCGAAA

GAGCAAAGAGGGGGATCTTCGGACCTCACGCCATCGGATGTGCAAGATGG

GATTAGCTAGTAGGTGAGGTAATGGCTCTTTCCGCTGACGATCCCTAGCT

GGTCTGAGAGGATGCCGGCCACGCTGGAACTGACCAGATCCACTCTCAAC

GGGAGGCAGCAGTGGGGAATATTGCACAATGGGCGCAAGCCTGATGCAGC

CATGCCGCGTGTGTGAAGAAGGCCTTAGGGTTGTAAAGCACTTTCAGCGA

GGAGGAAGGCATCATACTTAATACGTGTGGTGATTGACGTTACTCGCAGA

AGAAGCACGGGGTAACTCCGTGCCAGCAGCCGCGGTAATACGGAGGGTGC

AAGCGTTAATCGGAATTACTGGGGGGAAGCGCACGCAGGCGGTTTGTTAA

TCCGATGTGAAATCCCCGCGCTTAACGTGGAACTGCATTTTAAACTGGCA

GCTAGAGTCTTGGAGAGGGGGGAGAATTCCCGGTGTAGCGGTGAAATGCG

AGAGATCTGGAGGATACCGGTGGCGAAGCGGCCCCCTGGACAAACTGACG

CTCAGGTGCAAAGCG

>SS79

AGCTTCCGAACCGGTTAAGCACAGAGTTTTTTTTCTCTTGGGGCGAGAGA

CGGACGAGCGAAAAATATGAGGGACCTGGGCGAGGGGGAGGGATTTCTCT

AGTGGACGCTAGCGAAGACTACGTATCACGGCCTCAGAAAGGGTGGGACA

TTCTCGGCTCTCACACACGAGTATGCGCCGATGTGGTTAGATGGTGTGGG

GGGGGCTGGGTCACCCCTGGAACACTACCTGGTTGGGGTGAAAGAATAAG

CACACTCACTCTGACACAGAGACACGACTCATACTTCTGGGAGGAGTAGG

TGAGGGATATATTTCACTGTGCGCCCGCCCCAGACACGCCTGTGGCGTGT

ATAAGGAATGCGTCTTGGGTTTGACTTATATTTTGCGGGGAGGAAAGGGA

AGAGATAATTAACCCTTTCCATATTCCCGACCCCCTCAAAACACGCTCCC

TCTGTCCCCGCGCCCACCCCCGCTAAAACGGAGGGGGCGAAAGTTATTAT

TACTTTTTGTGGGCATAGCGCGCACCCGCGGTCTGTGTCGATCATATGTA

AAATCCGGGCTCTGCTCTGAACAGTGCTTGAGACGAGCTACACGAGACTC

TTGGTGAGGGAATACCTCCGGTGTGGAGCGGTGATGTGTATAGATCTCTG

GAACAATTCGCGATGCAAACGCCCCCCTCTGCAAAAAACTGTGGAGCACA

TGAGC

>SS80

TGATGGAGGGGCAGTTTAATGGAAACGGGAGCCAATGCCGTATAAAGTCT

GCGGACGAGAGTGGGGGACCCTCGGGCCTCACGCCTTCAGATGTGCCCAG

ATGGGATTAGCTGGTACGTGGGGGAATGGCGCACCTAGGCGACGATCTCT

AGCTGGGCTGAGAGGATGACAAGCCACACTGGAACTGAGACACGGTCCAA

ATCTCTACGGGAGGCAGCAGTGGGGAATATTGCACAATGGGCGCAAGCCT

GATGCAGCCATGCCGCGTGTGTGAAGAAGGCCTTAGGGTTGTAAAGCACT

TTCAGCGAGGAGGAAGGGTTCAGTGTTAATAGCACTGTTTTTTGACGTTA

CTCGCAGAAGAGGGACCGGCTAACTCCGTGCCAGCAGCCGCGGTAATACG

GAGGGTGCAAGCGTTAATCGGAATTACTGGGCGTAAAGCGCACGCAGGCG

GTTTGTTAAGTCAGATGTGAAATCCCCGCGCTTAACGTGGGAACTGCATT

TGAAACTGGCAAGCTAGAGTCTTGTAGAGGGGGGTAGAATTCCAGGTGTA

GCGGTGAAATGCGTAGAGATCTGGAGGAATACCGGTGGCGAAGGCGGCCC

CCTGGACAAAGACTGACGCTCACGTGCGAAAGCGTGGGGAGCAAACAGGA

TTAGATACCCTGTAGTCCACGCTGTAAACGATGTCGATTTGAGGTTGTGC

CCTTGAGGCGTGGCTTCCGGAGCTAACGCGTTAAATCGACCGCCTGGGGA

GTACGGCGCCAGTAAACTCAATGATTGAC

>SS81

GGTGGGTAACCTTGGTTACAATTTCCCCCATTCATGAATCAAAAGTGGTA

AGCGCCCTCCCGAAGGTACCTAAAAAAGGAAAGGGCGCAACCCATTCCCA

TGGTGTGACGGGCCCCCGAGGACAAGGGCCCGGGAACGTATTCACCGTAG

CATTCTGATCTACGATTACTAGCGATTCCGACTTCATGGAGTCGAGTTGC

AGACTCCAATCCGGACTACGACCTACTTTATGAGGGGGGGGGCTCTCGCG

AGTTCGCTTCTCTCTGTATATGCCATTGGAGCACGTGTGTAGCCCTACTC

GTAAGGGCCATGATGACTTGACGTCATCCCCACCTTCCTCCGGTTTATCA

CCGGCAGTCTCCTTTGAGTGCCCACCATAATGTGCTGGCAATAAAGGAAA

AGGGTTGGGCTCGTTGCGGAATTTACCCCACCTTTTCCAAACTTTTTTTT

GACGACAGCAATGCAGGGGCGGTCTCAATG

>SS82

CCTGATGGAGGGGGATATCTACTGGAAACGGTAGCTAATACCGCATGACC

TCGAAAGAGCAAAGTGGGGGATCTTCGGACCTCACGCCATCGGATGTGCC

CAGATGGGATTAGCTAGTAGGTGAGGTAATGGCTCACCTAGGCGACGATC

CCTAGCTGGTCTGAGAGGATGACCAGCCACACTGGAACTGAGACACGGTC

CAGACTCCTACGGGAGGCAGCAGTGGGGAATATTGCACAATGGGCGCAAG

CCTGATGCAGCCATGCCGCGTGTGTGAAGAAGGCCTTAGGGTTGTAAAGC

ACTTTCAGCGAGGAGGAAGGCATCACACTTAATACGTGTGGTGATTGACG

TTACTCGCAGAAGAAGCACCGGCTAACTCCGTGCCAGCAGCCGCGGTAAT

ACGGAGGGTGCAAGCGTTAATCGGAATTACTGGGCGTAAAGCGCACGCAG

GCGGTTTGTTAAATCAGATGTGAAATCCCCGCGCTTAACGTGGGGAACTG

CATTTGAAACTGGCAAGCTATAGTCTTGTAGAGGGGGGTAGAATTCCAGG

TGTAGCGGTGAAATGCGTAGAGATCTGGGAGGAATACCGGTGGCGAAGGC

GGCCCCCTGGACAAAAACTGACGCTCAGGTGCCAAAAGCGTGGGGGAGCA

AACAGGATTAGATACCCTGGTAGTCCACGCTGTAAACGATGTCGACTTGG

GAGGTTGTGCCCTTGAGGCGTGGCTTCCGGAGCTAACGCG

>SS83

GGTGTGACGGGCGGTGTGTACAAGGCCCGGGAACGTATTCACCGTAGCAT

TCTGATCTACGATTACTAGCGATTCCGACTTCATGGAGTCGAGTTGCAGA

CTCCAATCCGGACTACGACATACTTTATGAGGTCCGCTTGCTCTCGCGAG

TTCGCTTCTCTTTGTATATGCCATTGTAGCACGTGTGTAGCCCTACTCGT

AAGGGCCATGATGACTTGACGTCATCCCCACCTTCCTCCGGTTTATCACC

GGCAGTCTCCTTTGAGTTCCCACCATTACGTGCTGGCAACAAAGGATAAG

GGTTGCGCTCGTTGCGGGACTTAACCCAACATTTCACAACACGAGCTGAC

GACAGCCATGCAGCACCTGTCTCACGGTTCCCGAAGGCACTAAGCCATCT

CTGGCGAATTCCGTGGATGTCAAGAGTAGGTAAGGTTCTTCGCGTTGCAT

CGAATTAAACCACATGCTCCACCGCTTGTGCGGGCCCCCGTCAATTCATT

TGAGTTTTAACCTTGCGGCCGTACTCCCCAGGCGGTCGACTTAACGCGTT

AGCTCCGGAAGCCACGCCTCAAGGGCACAACCTCCAAGTCGACATCGTTT

ACAGCGTGGACTACCAGGGTATCTAATCCTGTTTGCTCCCCACGCTTTCG

CACCTGAGCGTCAGTCTTTGTCCAGGGGGCCGCCTTCGCCACCGGTATTC

CTCCAGATCTCTACGCATTTCACCGCTACACCTGGAATTCTACCCCCCTC

TACAGACTCTAGCTTGCCAGTTTCAAATGCAGTTCCCACGTTAAGCGCGG

GGATTTCACATCCTGACTTAACAAACCCGCCTGCGTGCGCTTTACGCCAG

TAATTCCCGATTAACGGCTTGCACCCCTCCGTATTACCGGCGGCTGCTGG

CACGGAGTTAGGCCGGTGCTTCTTCTGCGAGTAAACGTCAATCACCACAC

GTAATAAGAGATGATGCCCTTCCTCCTCCACTGAAAGGTGCTTTACAACC

CTTAGGACCTTTCTTCACCACACGCGAATGGCTTGAGATCAGGGTTTGCC

GCCAATTGTGCCAATATTCCCCCCATGCTGCTCCGGTAAGGAGT

>SS84

ATTCTGATCTACGATTACTAGCGATTCCGACTTCATGGAGTCGAGTTGCA

GACTCCAATCCGGACTACGACATACTTTATGAGGTCCGCTTGCTCTCGCG

AGTTCGCTTCTCTTTGTATATGCCATTGTAGCACGTGTGTAGCCCTACTC

GTAAGGGCCATGATGACTTGACGTCATCCCCACCTTCCTCCGGTTTATCA

CCGGCAGTCTCCTTTGAGTTCCCACCATTACGTGCTGGCAACAAAGGATA

AGGGTTGCGCTCGTTGCGGGACTTAACCCAACATTTCACAACACGAGCTG

ACGACAGCCATGCAGCACCTGTCTCACGGTTCCCGAAGGCACTAAGCCAT

CTCTGGCGAATTCCGTGGATGTCAAGAGTAGGTAAGGTTCTTCGCGTTGC

ATCGAATTAAACCACATGCTCCACCGCTTGTGCGGGCCCCCGTCAATTCA

TTTGAGTTTTAACCTTGCGGCCGTACTCCCCAGGCGGTCGACTTAACGCG

TTAGCTCCGGAAGCCACACCTCAAGGGCACAACCTCCAAGTCGACATCGT

TTACAGCGTGGACTACCAGGGTATCTAATCCTGTTTGCTCCCCACGCTTT

CGCACCTGAGCGTCAGTCTTTGTCCAGGGGGCCGCCTTCGCCACCGGTAT

TCCTCCAGATCTCTACGCATTTCACCGCTACACCTGGGAATTCTACCCCC

CTCTACAAGACTCTAGCTTGCCAGTTTCAAATGCAGTTCCCACGTTAAAC

GCGGGGATTTCACATCTGACTTAACAAACCGCCTGCGTGCGCTTTACGCC

CAGTAATTCCGATTAACGCTTGCACCCTCTGTATTACCGCGGCTGCTGGC

ACGGAAGTTAGCCGGGTGCTTCTTCTGGGAGTAACGTCAATCACCAATCA

CGTATTAAAGGGGAGATGCCTTTCCTTCCTCGACTGAAGTTGCTTTACAA

CCTAAGGACCTTCTTTCCAACCACGCGGCATGGCTGCAATCAGGGCTTGG

CGCCCAATGGGCAATATCCCAACTGCTTGGCTCCCGTAAGAA

>SS85

GTGGTAAGCGCCCTCCCGAAGGTTAAGCTACTTACTTCTTTTGCAACCCA

CTCCCATGGTGTGACGGGCGGTGTGTACAAGGCCCGGGAACGTATTCACC

GTAGCATTCTGATCTACGATTACTAGCGATTCCGACTTCATGGAGTCGAG

TTGCAGACTCCAATCCGGACTACGACGTACTTTATGAGGTCCGCTTGCTC

TCGCGAGGTCGCTTCTCTTTGTATACGCCATTGTAGCACGTGTGTAGCCC

TACTCGTAAGGGCCATGATGACTTGACGTCATCCCCACCTTCCTCCAGTT

TATCACTGGCAGTCTCCTTTGAGTTCCCGGCCGAACCGCTGGCAACAAAG

GATAAGGGTTGCGCTCGTTGCGGGACTTAACCCAACATTTCACAACACGA

GCTGACGACAGCCATGCAGCACCTGGCTCACGGTTCCCGAAGGGACTAAA

GCATCTCTGCTAAATTCCGTGGATGTCAAGAGTAGGTAAGGGTCTTCGCG

TTGCATCGAATTAAACCACATGCTCCACCGCTTGTGCGGGCCCCCGTCAA

TTCATTTGAGTTTTAACCTTGCGGCCGTACTCCCCAGGCGGTCGACTTAA

CGCGTTAGCTCCGGAAGCCACTCCTCAAGGGAACAACCTCCAAGTCGACA

TCGTTTACGGCGTGGACTACCAGGGTATCTAATCCTGTTTGCTCCCCACG

CTTTCGCACCTGAGCGTCAGTCTTTGTCCAGGGGGCCGCCTTCGCCACCG

GTATTCCTCCAGATCTCTACGCATTTCACCGCTACACCTGGAAATCTACC

CCCCTCTACAAGACTCTAGCCTGGCAGTTTCGAATGCAGTTCCCAGGGTT

GAGCCCGGGGATTTCACCATCCTGACTTGACAGACCGGCCTGCGTGCGCT

TTACGCCCAGTAATTCCCGATACGCTTTGCACCCTCCGTATACCGCGGCT

GCTGGCACGGAGTAGCCGTGCTTCTTCTGCGAGTAACGTCATCACTGAGT

TATTACTTGAATGCCTTCCTCCTCGCTGAAGTACTTACACCCGAGGCTTC

CTCATACACGCGCATGCCTGCATCAGCTGCGTCAATGGCATATTCCCTCT

GCTGCTTCGTAGGATCTGAACCCGGTTCCAGTCCAGTGGCTGACATCTCT
