# Supplementary material for: Initial nitrogen enrichment conditions determines variations in nitrogen substrate utilization by heterotrophic bacterial isolates
Source: BMC Microbiol. 2017 Apr 4;17:87. doi: 10.1186/s12866-017-0993-7 (PMC5381026; doi:10.1186/s12866-017-0993-7)
Supplement: Supplementary file 3 — Title: Growth rates of all bacterial isolates from the initial N-enrichments on each of the 12 substrates. Description of data: The growth rates of each bacterial isolate from each N-enrichment on the 12 N-substrates used in the study. (DOCX 55 kb) [file 12866_2017_993_MOESM3_ESM.docx]

**Table S2.** Growth rates (day^-1^) of all bacterial isolates from the initial N-enrichments on each of the 12 substrates

| **N-enrichment type** | **Isolates** | **Nitrate** | **Ammonium** | **Urea** | **Glycine** | **Proline** | **Tryptophan** | **Nucleic Acid** | **Peptidoglycan** | **Bacterial Protein** | **Polyamine** | **Algal Exudate** | **Humics** |
| --- | --- | --- | --- | --- | --- | --- | --- | --- | --- | --- | --- | --- | --- |
| Ammonium | J47 | 21.66 | 31.32 | 22.686 | 29.796 | 6.594 | 2.604 | 2.118 | 2.088 | 3.276 | 6.636 | 12.174 | 1.854 |
| Ammonium | SS68 | 40.608 | 37.218 | 36.912 | 21.39 | 26.91 | 49.392 | 1.548 | 9.348 | 27.3 | 36.84 | 43.47 | 7.236 |
| Ammonium | J44 | 24 | 31.8 | 32.292 | 33.102 | 26.04 | 69.078 | 1.602 | 25.284 | 23.166 | 36.396 | 46.5 | 14.496 |
| Ammonium | SS67 | 45.564 | 43.584 | 42.648 | 42.012 | 23.616 | 54.108 | -0.528 | 10.89 | 29.622 | 42.462 | 54.336 | 8.184 |
| Ammonium | SS65 | 29.148 | 47.094 | 36.564 | 42.168 | 24.744 | 71.874 | 2.844 | 23.31 | 23.67 | 39.228 | 49.188 | 13.356 |
| Ammonium | J45 | 44.748 | 59.466 | 45.174 | 28.476 | 28.392 | 58.158 | 5.778 | 12.876 | 25.722 | 42.18 | 43.764 | 9.402 |
| Ammonium | J49 | 67.74 | 50.022 | 59.304 | 52.908 | 38.394 | 27.906 | 4.428 | 6.96 | 27.546 | 41.346 | 48.366 | 7.014 |
| Ammonium | J51 | 56.262 | 66.426 | 61.962 | 46.878 | 48.06 | 22.344 | 13.062 | 11.982 | 24.318 | 36.312 | 37.32 | 8.43 |
| Ammonium | J48 | 70.536 | 62.052 | 72.852 | 44.016 | 44.664 | 20.964 | 4.374 | 6.036 | 24.636 | 35.88 | 44.31 | 4.938 |
| Ammonium | J42 | 66.198 | 60.522 | 74.574 | 47.286 | 46.14 | 27.3 | 3.642 | 5.724 | 30.51 | 39.72 | 52.176 | 5.346 |
| Ammonium | SYC40 | 40.296 | 52.56 | 47.58 | 44.322 | 50.196 | 73.542 | 2.304 | 22.698 | 26.742 | 38.952 | 53.088 | 11.976 |
| Ammonium | SS69 | 43.896 | 43.26 | 48.456 | 43.194 | 43.872 | 58.506 | 19.662 | 27.156 | 32.52 | 43.158 | 52.212 | 11.976 |
| Ammonium | J50 | 73.008 | 50.022 | 72.462 | 48.69 | 50.724 | 32.166 | 3.822 | 5.73 | 30.54 | 38.52 | 60.174 | 4.878 |
| Ammonium | J41 | 66.336 | 55.332 | 58.704 | 73.344 | 73.68 | 20.538 | 2.262 | 4.56 | 37.02 | 36.156 | 53.718 | 5.016 |
| Ammonium | SS71 | 50.04 | 49.5 | 54.414 | 43.566 | 46.362 | 62.616 | 3.036 | 30.342 | 29.64 | 42.336 | 59.106 | 19.11 |
| Ammonium | J43 | 56.232 | 65.61 | 68.256 | 64.422 | 43.986 | 54.804 | 3.354 | 7.266 | 25.908 | 42.096 | 53.712 | 5.88 |
| Ammonium | SS74 | 61.272 | 65.202 | 58.518 | 59.196 | 64.548 | 48.696 | 6.042 | 9.372 | 30.372 | 37.944 | 54.234 | 6.216 |
| Ammonium | SYC37 | 51.732 | 57.558 | 52.77 | 44.754 | 60.696 | 67.98 | 4.902 | 21.414 | 27.726 | 45.222 | 55.248 | 12.588 |
| Ammonium | SS66 | 53.328 | 57.282 | 58.17 | 67.566 | 62.298 | 49.356 | 6.696 | 18.102 | 30.948 | 37.74 | 49.602 | 12.432 |
| Ammonium | SYC38 | 57.462 | 57.27 | 53.988 | 46.134 | 60.672 | 68.94 | 3.252 | 20.514 | 26.946 | 43.974 | 55.236 | 12.762 |
| Ammonium | SS73 | 48.162 | 51.732 | 46.386 | 53.286 | 48.912 | 72.498 | 3.654 | 27.762 | 32.64 | 48.666 | 67.584 | 14.832 |
| Ammonium | SS72 | 49.074 | 54.546 | 63.75 | 67.104 | 69.204 | 65.184 | 3.612 | 15.804 | 26.616 | 38.34 | 56.184 | 10.986 |
| Ammonium | SYC35 | 47.712 | 66.174 | 66.012 | 52.686 | 66.84 | 73.98 | 3.858 | 21.69 | 26.292 | 43.644 | 58.83 | 12.03 |
| Ammonium | SS75 | 45.516 | 61.536 | 49.626 | 53.736 | 50.112 | 78.978 | 6.138 | 39.126 | 35.268 | 45.792 | 52.974 | 28.302 |
| Ammonium | SYC39 | 66.486 | 69.714 | 63.198 | 50.412 | 48.078 | 69.918 | 7.89 | 22.8 | 32.178 | 53.07 | 54.03 | 16.176 |
| Ammonium | SYC31 | 65.394 | 62.088 | 57.564 | 46.212 | 57.57 | 75.732 | 6.624 | 30.804 | 29.832 | 55.164 | 54.858 | 18.516 |
| Ammonium | SYC32 | 65.55 | 67.488 | 59.73 | 41.22 | 52.404 | 84.252 | 6.948 | 31.164 | 30.918 | 59.19 | 59.55 | 18.57 |
| Ammonium | SS70 | 57.99 | 61.734 | 58.434 | 46.722 | 59.55 | 87.87 | 9.972 | 44.904 | 37.542 | 51.63 | 61.758 | 23.616 |
| Ammonium | SYC33 | 63.654 | 68.502 | 61.458 | 52.032 | 56.1 | 86.436 | 5.874 | 32.544 | 33.624 | 59.67 | 61.992 | 21.114 |
| Ammonium | J46 | 79.668 | 57.666 | 64.56 | 56.994 | 66.21 | 43.134 | 55.59 | 33.426 | 36.258 | 49.242 | 48.066 | 22.326 |
| Ammonium | SYC36 | 79.26 | 71.508 | 63.162 | 48.432 | 66.132 | 78.288 | 11.142 | 32.196 | 35.298 | 57.882 | 59.544 | 20.07 |
| Ammonium | SYC34 | 74.472 | 68.964 | 60.48 | 60.18 | 66.696 | 77.652 | 11.982 | 32.328 | 32.676 | 58.2 | 60.522 | 20.928 |
| Bacterial Protein | J61 | 0.246 | 0.222 | 32.082 | 0.054 | 0.522 | 54.144 | 0.594 | 8.934 | 7.848 | 19.518 | 29.772 | 4.236 |
| Bacterial Protein | J63 | 38.022 | 22.35 | 42.108 | 26.982 | 12.978 | 1.77 | 2.286 | 1.392 | 0.984 | 7.236 | 12.564 | 0.534 |
| Bacterial Protein | J62 | 37.752 | 31.968 | 29.928 | 39.792 | 41.016 | 8.892 | 2.328 | 3.426 | 15.012 | 14.592 | 25.338 | 2.406 |
| Bacterial Protein | J66 | 37.866 | 34.392 | 32.556 | 21.15 | 27.9 | 39.972 | 0.27 | 8.304 | 14.892 | 27 | 32.574 | 5.292 |
| Bacterial Protein | J64 | 45.552 | 40.884 | 41.982 | 39.684 | 48.51 | 27.3 | 1.446 | 4.614 | 18 | 23.13 | 32.67 | 2.604 |
| Bacterial Protein | J65 | 41.028 | 38.34 | 41.832 | 35.82 | 42.084 | 41.862 | -0.408 | 8.952 | 17.652 | 27.516 | 41.388 | 4.848 |
| Bacterial Protein | SS17 | 41.844 | 48.264 | 48.72 | 40.08 | 37.626 | 34.68 | 2.664 | 6.84 | 21.756 | 25.446 | 35.598 | 6.06 |
| Bacterial Protein | SS11 | 55.176 | 10.068 | 44.946 | 32.454 | 37.788 | 43.644 | 13.272 | 17.766 | 18.258 | 29.43 | 39.984 | 7.986 |
| Bacterial Protein | SS13 | 57.138 | 53.472 | 50.646 | 45.702 | 37.686 | 31.752 | 2.796 | 4.374 | 14.712 | 21.324 | 36.702 | 2.544 |
| Bacterial Protein | SYC9 | 49.302 | 40.746 | 31.182 | 37.926 | 31.968 | 59.742 | 1.158 | 15.894 | 15.504 | 26.37 | 43.014 | 8.616 |
| Bacterial Protein | SS14 | 57.54 | 52.344 | 49.938 | 43.296 | 42.678 | 31.2 | 1.668 | 5.298 | 18.342 | 22.056 | 36.636 | 3.504 |
| Bacterial Protein | SS15 | 46.374 | 50.346 | 45.69 | 32.394 | 36.708 | 49.518 | 3.846 | 15.444 | 19.242 | 25.974 | 38.046 | 9.51 |
| Bacterial Protein | SYC10 | 51.456 | 37.164 | 30.948 | 51.258 | 33.9 | 57.804 | 1.614 | 13.44 | 15.756 | 28.398 | 45.6 | 7.374 |
| Bacterial Protein | SS18 | 48.744 | 44.634 | 41.478 | 36.234 | 37.08 | 54.258 | 3.174 | 8.334 | 22.152 | 30.192 | 47.712 | 5.598 |
| Bacterial Protein | SYC6 | 43.794 | 52.884 | 41.808 | 40.512 | 41.112 | 57.612 | 1.956 | 15.27 | 14.508 | 27.318 | 43.338 | 8.202 |
| Bacterial Protein | SYC2 | 50.952 | 47.682 | 46.344 | 42.972 | 36.996 | 57.54 | 0.684 | 13.956 | 15.9 | 32.316 | 44.418 | 8.7 |
| Bacterial Protein | SS16 | 55.62 | 56.586 | 50.562 | 37.776 | 36.21 | 44.328 | 4.242 | 13.704 | 22.02 | 38.442 | 38.832 | 9.306 |
| Bacterial Protein | SYC5 | 44.292 | 50.13 | 48.798 | 44.202 | 41.766 | 57.666 | 4.11 | 15.846 | 17.634 | 29.634 | 47.904 | 9.174 |
| Bacterial Protein | SYC8 | 51.216 | 49.368 | 47.82 | 43.908 | 41.688 | 57.852 | 2.406 | 15 | 19.17 | 31.698 | 47.91 | 9.096 |
| Bacterial Protein | SS12 | 64.89 | 64.332 | 52.98 | 44.82 | 42.39 | 41.418 | 3.324 | 9.444 | 21.03 | 30.228 | 36.54 | 6.096 |
| Bacterial Protein | SYC1 | 50.592 | 54.642 | 58.254 | 41.334 | 44.55 | 54.612 | 1.416 | 15.318 | 19.26 | 32.514 | 40.758 | 9.45 |
| Bacterial Protein | SYC3 | 52.68 | 53.16 | 51.696 | 45.858 | 44.172 | 61.002 | 4.746 | 16.296 | 17.172 | 31.266 | 47.784 | 8.856 |
| Bacterial Protein | SS19 | 58.086 | 61.332 | 50.838 | 37.542 | 42.864 | 49.86 | 4.416 | 14.16 | 26.352 | 40.482 | 44.142 | 7.986 |
| Bacterial Protein | SS20 | 68.1 | 35.496 | 55.896 | 46.752 | 51.156 | 50.298 | 3.834 | 16.644 | 20.88 | 36.774 | 42.636 | 10.506 |
| Bacterial Protein | SYC7 | 48.72 | 58.392 | 54.546 | 51.348 | 44.814 | 58.056 | 3.162 | 15.588 | 21.762 | 31.158 | 47.658 | 9.612 |
| Bacterial Protein | SYC4 | 59.934 | 50.928 | 49.974 | 46.848 | 51.3 | 62.118 | 5.136 | 15.066 | 18.678 | 33.648 | 50.544 | 8.25 |
| Defined-N-mixture | SS21 | 57.006 | 33.282 | 33.606 | 30.132 | 34.77 | 26.262 | 19.038 | 10.56 | 7.02 | 14.376 | 32.724 | 1.26 |
| Defined-N-mixture | J55 | 50.004 | 49.14 | 56.646 | 41.034 | 33.384 | 62.376 | 2.862 | 7.224 | 25.776 | 44.43 | 53.16 | 7.608 |
| Defined-N-mixture | SYC47 | 40.752 | 51.45 | 43.878 | 45.612 | 56.772 | 61.47 | 3.414 | 12.096 | 26.412 | 34.038 | 51.276 | 6.942 |
| Defined-N-mixture | J56 | 75.036 | 54.486 | 50.202 | 29.148 | 47.442 | 32.34 | 13.782 | 20.658 | 26.964 | 39.054 | 33.438 | 15.018 |
| Defined-N-mixture | SYC51 | 41.634 | 44.19 | 54.198 | 45.708 | 50.556 | 61.446 | 2.226 | 13.674 | 31.242 | 39.192 | 54.444 | 9.36 |
| Defined-N-mixture | J59 | 72.15 | 61.17 | 71.772 | 52.014 | 52.356 | 28.938 | 0.57 | 3.27 | 24.546 | 28.968 | 47.772 | 4.866 |
| Defined-N-mixture | SS23 | 48.186 | 53.898 | 50.646 | 52.092 | 32.244 | 51.336 | 21.81 | 29.328 | 25.068 | 39.834 | 46.668 | 13.908 |
| Defined-N-mixture | SS28 | 51.504 | 45.042 | 50.076 | 43.59 | 82.476 | 46.146 | 11.52 | 8.13 | 38.25 | 35.52 | 53.16 | 2.388 |
| Defined-N-mixture | J58 | 56.766 | 55.608 | 54.93 | 33.606 | 43.074 | 61.596 | 2.706 | 27.09 | 33.252 | 50.682 | 49.002 | 14.58 |
| Defined-N-mixture | SS24 | 50.91 | 66.222 | 62.112 | 50.502 | 42.972 | 55.188 | 18.444 | 22.008 | 25.596 | 34.47 | 50.688 | 10.788 |
| Defined-N-mixture | SYC49 | 60.102 | 67.356 | 60.99 | 53.658 | 63.114 | 45.798 | 11.19 | 16.998 | 28.59 | 43.236 | 41.868 | 14.25 |
| Defined-N-mixture | SS25 | 60.51 | 74.892 | 69.45 | 46.116 | 41.91 | 63.9 | 2.892 | 16.062 | 26.688 | 41.1 | 53.982 | 10.638 |
| Defined-N-mixture | SYC42 | 50.31 | 66.93 | 70.932 | 56.79 | 71.904 | 46.926 | 4.566 | 13.818 | 33.432 | 39.09 | 48.384 | 8.46 |
| Defined-N-mixture | SYC46 | 66.462 | 71.442 | 66.96 | 47.448 | 50.766 | 52.722 | 4.2 | 14.73 | 31.272 | 48.102 | 46.014 | 13.26 |
| Defined-N-mixture | SS22 | 69.828 | 71.58 | 68.838 | 46.242 | 43.968 | 47.04 | 24.288 | 15.504 | 28.374 | 42.732 | 53.478 | 4.674 |
| Defined-N-mixture | SYC48 | 64.158 | 67.284 | 52.464 | 54.876 | 60.756 | 66.162 | 5.118 | 14.214 | 26.532 | 38.616 | 59.334 | 10.098 |
| Defined-N-mixture | J57 | 51.582 | 59.574 | 57.084 | 45.324 | 52.968 | 71.898 | 5.31 | 30.018 | 29.916 | 48.996 | 51.156 | 17.406 |
| Defined-N-mixture | SYC50 | 50.58 | 62.388 | 60.984 | 58.908 | 73.152 | 56.97 | 4.068 | 15.972 | 35.13 | 43.908 | 59.07 | 11.55 |
| Defined-N-mixture | SYC41 | 57.924 | 68.832 | 58.62 | 58.476 | 62.976 | 56.892 | 11.076 | 18.426 | 35.118 | 43.248 | 48.306 | 14.58 |
| Defined-N-mixture | SS30 | 56.916 | 37.002 | 34.266 | 54.9 | 32.394 | 91.074 | 17.88 | 43.44 | 32.682 | 45.714 | 71.262 | 19.98 |
| Defined-N-mixture | SYC45 | 61.05 | 72.006 | 75.36 | 54.942 | 59.508 | 57.678 | 0.216 | 16.722 | 32.796 | 48.816 | 52.878 | 13.026 |
| Defined-N-mixture | SS27 | 55.2 | 65.46 | 89.664 | 80.538 | 61.062 | 40.848 | 19.008 | 12.27 | 26.016 | 40.35 | 56.49 | 1.224 |
| Defined-N-mixture | SYC44 | 68.046 | 70.668 | 68.154 | 56.406 | 60.348 | 54.714 | 8.028 | 16.716 | 32.502 | 47.934 | 50.472 | 14.382 |
| Defined-N-mixture | J53 | 58.29 | 54.942 | 55.848 | 50.778 | 44.652 | 83.238 | 5.904 | 35.802 | 28.626 | 56.076 | 58.434 | 22.068 |
| Defined-N-mixture | SYC43 | 63.576 | 71.898 | 69.714 | 56.25 | 69.654 | 59.574 | 11.082 | 14.592 | 30.654 | 48.33 | 53.22 | 12.03 |
| Defined-N-mixture | J60 | 71.454 | 60.324 | 55.032 | 59.922 | 44.598 | 75.816 | 7.404 | 34.482 | 30.732 | 53.238 | 60.066 | 21.9 |
| Defined-N-mixture | J54 | 57.906 | 60.144 | 52.344 | 53.16 | 59.46 | 82.47 | 12.348 | 34.734 | 34.95 | 53.94 | 61.026 | 20.76 |
| Defined-N-mixture | J52 | 65.286 | 56.496 | 48.006 | 43.152 | 62.748 | 69.3 | 13.638 | 41.994 | 45.822 | 57.552 | 55.302 | 30.426 |
| Defined-N-mixture | SYC52 | 51.312 | 69.588 | 48.474 | 62.832 | 112.692 | 67.32 | 4.332 | 14.202 | 45.936 | 50.424 | 66.318 | 10.5 |
| Defined-N-mixture | SS26 | 77.166 | 68.484 | 72.366 | 81.72 | 62.592 | 69.558 | 23.838 | 21.858 | 35.136 | 42.756 | 72.966 | 9.684 |
| Defined-N-mixture | SS29 | 102.258 | 78.87 | 83.07 | 81.564 | 100.356 | 66.534 | 9.408 | 25.53 | 41.496 | 50.766 | 70.032 | 15.432 |
| Glycine | SS49 | 60.6 | 52.692 | 47.682 | 60.876 | 41.496 | 15.582 | 2.196 | 3.99 | 18.402 | 25.08 | 36.492 | 3.924 |
| Glycine | J23 | 52.194 | 63.894 | 75.69 | 67.146 | 62.4 | 12.264 | 2.388 | 4.674 | 15.804 | 16.176 | 33.252 | 3.882 |
| Glycine | SYC69 | 43.5 | 37.662 | 40.704 | 53.304 | 45.174 | 52.35 | -0.198 | 8.592 | 37.086 | 44.328 | 56.874 | 8.754 |
| Glycine | J17 | 44.484 | 64.104 | 110.958 | 58.902 | 53.16 | 15.69 | 2.886 | 5.034 | 19.5 | 27.984 | 33.084 | 4.116 |
| Glycine | SYC73 | 48.942 | 49.758 | 39.69 | 40.818 | 47.586 | 55.83 | 3.318 | 13.788 | 36.75 | 44.01 | 50.112 | 9.876 |
| Glycine | SS42 | 62.838 | 67.428 | 61.104 | 43.776 | 40.698 | 34.974 | 10.368 | 12.462 | 24.624 | 38.808 | 37.44 | 9.888 |
| Glycine | SS44 | 62.424 | 75.432 | 61.56 | 42 | 40.422 | 33.852 | 8.412 | 12.666 | 27.066 | 38.388 | 35.388 | 10.83 |
| Glycine | SYC70 | 58.068 | 63.282 | 57.954 | 42.978 | 48.456 | 50.4 | 1.572 | 8.856 | 26.472 | 43.074 | 46.836 | 7.764 |
| Glycine | J19 | 49.71 | 53.646 | 66.702 | 60.828 | 53.874 | 40.95 | 2.058 | 6.384 | 31.65 | 39.204 | 54.396 | 7.422 |
| Glycine | SYC74 | 54.546 | 49.38 | 36.456 | 51.6 | 56.748 | 59.628 | 2.25 | 14.04 | 34.86 | 42.516 | 55.128 | 9.966 |
| Glycine | SYC72 | 56.124 | 59.604 | 58.176 | 47.886 | 45.912 | 54 | 1.722 | 11.622 | 32.052 | 42.792 | 48.672 | 8.82 |
| Glycine | J24 | 61.938 | 37.782 | 47.982 | 81.192 | 71.922 | 21.792 | 4.776 | 7.662 | 40.908 | 32.826 | 52.842 | 8.262 |
| Glycine | SYC68 | 61.428 | 65.634 | 54.318 | 49.524 | 50.418 | 55.236 | 5.538 | 13.08 | 29.898 | 44.688 | 49.752 | 10.128 |
| Glycine | SYC67 | 58.764 | 67.506 | 61.776 | 53.79 | 52.992 | 51.426 | 4.62 | 13.872 | 29.352 | 46.326 | 49.944 | 10.692 |
| Glycine | J22 | 66.366 | 60.93 | 77.154 | 70.086 | 60.162 | 27.312 | 3.246 | 8.124 | 29.868 | 39.87 | 53.034 | 7.548 |
| Glycine | SS45 | 79.89 | 88.92 | 67.332 | 52.068 | 53.76 | 27.972 | 6.522 | 8.91 | 30.654 | 42.114 | 38.094 | 10.35 |
| Glycine | SYC66 | 58.242 | 64.728 | 63.552 | 49.542 | 46.194 | 62.148 | 8.634 | 14.112 | 30.108 | 47.694 | 55.986 | 11.154 |
| Glycine | SYC65 | 57.462 | 62.514 | 57.342 | 54.768 | 62.772 | 52.14 | 9.834 | 15.306 | 34.35 | 44.79 | 52.014 | 11.856 |
| Glycine | SS41 | 61.35 | 67.614 | 75.474 | 62.964 | 58.698 | 29.796 | 11.592 | 14.166 | 34.176 | 42.804 | 45.06 | 12.324 |
| Glycine | SS50 | 50.976 | 57.606 | 54.792 | 61.296 | 60.522 | 60.84 | 10.158 | 25.32 | 36.48 | 45.39 | 55.38 | 17.694 |
| Glycine | SYC71 | 59.43 | 65.136 | 55.182 | 60.552 | 70.92 | 61.548 | 6.456 | 15.864 | 37.26 | 49.758 | 62.412 | 11.796 |
| Glycine | J21 | 75.93 | 71.958 | 76.194 | 76.704 | 35.142 | 43.866 | 4.536 | 10.446 | 40.578 | 47.376 | 69.762 | 11.13 |
| Glycine | J20 | 54.786 | 91.392 | 66.006 | 69.912 | 69.096 | 47.214 | 2.778 | 6.198 | 36.798 | 46.32 | 66.768 | 6.648 |
| Glycine | SS47 | 70.578 | 72.948 | 77.34 | 75.06 | 73.908 | 27.858 | 7.488 | 12.096 | 41.172 | 39.012 | 55.326 | 11.832 |
| Glycine | J16 | 77.706 | 76.896 | 92.628 | 85.128 | 45.786 | 47.028 | 3.264 | 8.724 | 48.954 | 54.756 | 80.604 | 9.102 |
| Glycine | SS48 | 80.364 | 79.506 | 78.99 | 88.086 | 77.268 | 52.326 | 5.658 | 11.226 | 40.95 | 47.946 | 70.248 | 10.554 |
| Glycine | SS43 | 100.41 | 88.218 | 88.284 | 81.306 | 63.414 | 64.32 | 5.31 | 10.158 | 40.35 | 50.91 | 76.938 | 9.498 |
| Glycine | SS46 | 76.752 | 87.87 | 101.298 | 104.166 | 77.352 | 51.504 | 5.25 | 7.914 | 34.314 | 54.672 | 75.324 | 11.502 |
| Glycine | J18 | 59.574 | 83.364 | 93.072 | 89.406 | 95.772 | 65.496 | 7.26 | 11.364 | 55.914 | 61.14 | 92.868 | 12 |
| Nitrate | J25 | 43.074 | 42.624 | 41.25 | 19.698 | 42.984 | 11.214 | 3.444 | 5.952 | 15.768 | 23.826 | 18.6 | 5.982 |
| Nitrate | J28 | 47.154 | 38.91 | 38.04 | 19.782 | 18.282 | 23.364 | 4.56 | 6.186 | 15.24 | 31.104 | 26.328 | 5.73 |
| Nitrate | SS39 | 42.138 | 59.076 | 58.614 | 51.528 | 30.738 | 10.752 | 4.746 | 5.598 | 21.312 | 25.812 | 26.28 | 5.64 |
| Nitrate | J27 | 57.45 | 46.824 | 54.204 | 36.33 | 44.838 | 20.46 | 3.822 | 4.152 | 23.292 | 30.162 | 44.262 | 2.748 |
| Nitrate | SS40 | 43.026 | 53.316 | 52.056 | 46.608 | 43.44 | 36.654 | 5.574 | 7.98 | 24.504 | 35.136 | 42.684 | 6.696 |
| Nitrate | SS37 | 46.416 | 62.718 | 50.484 | 32.652 | 32.616 | 47.436 | 5.13 | 21.834 | 25.482 | 38.52 | 37.554 | 13.86 |
| Nitrate | J30 | 57.384 | 62.316 | 60.576 | 41.838 | 31.488 | 28.512 | 15.036 | 18.222 | 21.51 | 38.208 | 31.998 | 13.638 |
| Nitrate | J31 | 72.75 | 60.618 | 55.536 | 61.134 | 45.426 | 9.816 | -4.896 | 0.366 | 33.876 | 33.258 | 51.6 | 3.12 |
| Nitrate | J32 | 74.244 | 68.496 | 61.272 | 44.562 | 33.69 | 18.204 | -6.756 | -1.194 | 37.398 | 34.506 | 58.2 | 1.14 |
| Nitrate | SS31 | 59.358 | 59.802 | 58.752 | 41.424 | 37.608 | 35.778 | 8.172 | 11.25 | 28.704 | 42.666 | 38.526 | 10.596 |
| Nitrate | SYC25 | 58.758 | 57.456 | 58.878 | 31.506 | 31.968 | 54.576 | 9.792 | 13.272 | 23.79 | 44.784 | 42.57 | 9.744 |
| Nitrate | SYC23 | 57.3 | 48.054 | 53.412 | 43.428 | 40.014 | 54.822 | 9.408 | 15.024 | 22.188 | 42.228 | 44.958 | 12.528 |
| Nitrate | J26 | 59.886 | 52.86 | 59.166 | 30.216 | 39.888 | 52.014 | 2.652 | 4.452 | 29.646 | 46.224 | 63.816 | 4.446 |
| Nitrate | SYC24 | 60.012 | 59.034 | 58.014 | 40.404 | 40.218 | 55.896 | 7.434 | 15.894 | 23.196 | 43.392 | 44.652 | 12.516 |
| Nitrate | SYC21 | 55.626 | 53.136 | 55.566 | 53.754 | 43.98 | 58.068 | 6.6 | 13.77 | 22.902 | 32.7 | 53.238 | 12.648 |
| Nitrate | SYC22 | 61.218 | 59.01 | 61.362 | 44.388 | 42.816 | 54.492 | 10.038 | 16.764 | 23.646 | 44.358 | 44.694 | 17.88 |
| Nitrate | SS32 | 58.41 | 62.334 | 64.998 | 58.752 | 71.412 | 33.654 | 6.324 | 10.002 | 29.904 | 35.538 | 49.824 | 6.504 |
| Nitrate | SYC27 | 62.142 | 61.914 | 62.358 | 38.766 | 53.724 | 55.524 | 10.356 | 18.552 | 23.454 | 45.84 | 42.822 | 12.888 |
| Nitrate | SS36 | 61.41 | 66.234 | 64.908 | 50.64 | 40.674 | 58.644 | 12.972 | 14.67 | 30.774 | 50.256 | 52.542 | 11.67 |
| Nitrate | SYC26 | 67.896 | 70.032 | 67.836 | 44.082 | 50.694 | 64.584 | 6.894 | 16.632 | 23.652 | 49.716 | 50.85 | 11.568 |
| Nitrate | SYC30 | 65.592 | 65.664 | 61.566 | 58.392 | 52.152 | 64.098 | 5.34 | 16.626 | 25.434 | 44.58 | 57.336 | 11.088 |
| Nitrate | SS35 | 60.96 | 67.734 | 66.582 | 55.332 | 61.014 | 55.23 | 11.106 | 15.93 | 34.722 | 45.93 | 50.922 | 11.424 |
| Nitrate | SYC29 | 65.856 | 67.452 | 69.828 | 51.174 | 66.114 | 61.488 | 10.002 | 20.172 | 28.002 | 46.686 | 44.796 | 13.854 |
| Nitrate | SS33 | 54.192 | 82.992 | 74.22 | 78.408 | 77.7 | 21.36 | 13.338 | 12.402 | 39.936 | 38.4 | 46.044 | 9.108 |
| Nitrate | SYC28 | 71.778 | 65.868 | 63.786 | 59.046 | 63.228 | 63.78 | 7.206 | 18.594 | 25.794 | 50.082 | 56.31 | 13.566 |
| Nitrate | SS38 | 57.378 | 67.374 | 72.12 | 56.406 | 46.662 | 69.822 | 13.056 | 30.906 | 27.648 | 48.384 | 54.96 | 18.078 |
| Nitrate | J29 | 87.006 | 82.338 | 97.806 | 92.862 | 84.966 | 30.066 | 5.766 | 11.034 | 45.252 | 47.406 | 71.79 | 11.94 |
| Nitrate | SS34 | 71.802 | 94.974 | 85.77 | 72.732 | 51.474 | 83.292 | 6.708 | 29.688 | 34.452 | 54.252 | 74.316 | 20.202 |
| Nutrient Broth | SS53 | 37.074 | 16.596 | 51.798 | 16.338 | 20.652 | 10.602 | 4.188 | 4.356 | 9.096 | 14.568 | 21.084 | 4.974 |
| Nutrient Broth | SS52 | 36.324 | 29.826 | 27.45 | 26.28 | 22.596 | 7.776 | 10.65 | 9 | 13.776 | 14.436 | 17.046 | 4.56 |
| Nutrient Broth | J9 | 40.884 | 40.404 | 36.606 | 33.378 | 25.95 | 14.106 | -0.672 | 4.788 | 18.426 | 28.458 | 24.612 | 3.468 |
| Nutrient Broth | SS51 | 19.854 | 33.414 | 29.676 | 29.28 | 23.592 | 50.166 | 3.198 | 13.86 | 22.194 | 29.454 | 40.02 | 9.384 |
| Nutrient Broth | SS58 | 25.47 | 53.514 | 49.206 | 42.222 | 38.046 | 33.378 | 1.758 | 7.866 | 17.232 | 29.292 | 31.098 | 5.61 |
| Nutrient Broth | SS57 | 33.558 | 55.488 | 52.836 | 42.612 | 32.724 | 38.544 | 3.612 | 9.18 | 19.362 | 27.396 | 36.93 | 5.85 |
| Nutrient Broth | SYC62 | 41.988 | 40.878 | 34.998 | 28.992 | 43.836 | 50.55 | 1.212 | 10.452 | 24.138 | 32.37 | 42.072 | 9.66 |
| Nutrient Broth | SS60 | 35.19 | 61.782 | 52.956 | 35.352 | 32.328 | 43.38 | 4.128 | 8.562 | 22.152 | 30.228 | 42.762 | 7.368 |
| Nutrient Broth | SYC54 | -0.048 | 48.306 | 50.568 | 37.398 | 40.932 | 63.798 | 5.424 | 18.894 | 21.45 | 37.866 | 45.144 | 11.232 |
| Nutrient Broth | SYC56 | 46.452 | 38.73 | 40.362 | 27.438 | 32.19 | 64.884 | 3.288 | 15.594 | 25.452 | 37.53 | 45.168 | 10.242 |
| Nutrient Broth | SYC53 | 40.254 | 38.724 | 43.11 | 28.098 | 39.138 | 60.972 | 3.324 | 16.146 | 27.888 | 34.716 | 44.874 | 10.53 |
| Nutrient Broth | SS62 | 43.212 | 44.628 | 40.884 | 30.594 | 48.894 | 42.852 | 18.246 | 10.248 | 29.13 | 31.836 | 45.438 | 3.72 |
| Nutrient Broth | SYC61 | 45.186 | 36.222 | 34.758 | 34.134 | 41.652 | 61.05 | 3.27 | 15.828 | 24.276 | 34.908 | 47.586 | 10.854 |
| Nutrient Broth | J11 | 42.204 | 33.996 | 35.628 | 35.016 | 47.19 | 42.624 | 2.628 | 3.69 | 36.498 | 42.972 | 66.132 | 4.896 |
| Nutrient Broth | SS54 | 39.354 | 67.08 | 54.606 | 32.028 | 38.16 | 49.722 | 4.344 | 10.356 | 24.258 | 36.006 | 39.696 | 7.35 |
| Nutrient Broth | J8 | 48.804 | 49.194 | 61.596 | 42.612 | 59.862 | 18.69 | 2.484 | 7.686 | 33.846 | 35.52 | 42.138 | 7.368 |
| Nutrient Broth | SS63 | 50.118 | 67.104 | 54.96 | 38.976 | 40.5 | 40.35 | 17.532 | 17.886 | 20.988 | 30.798 | 38.106 | 8.742 |
| Nutrient Broth | SS56 | 41.088 | 73.488 | 52.506 | 47.496 | 49.848 | 40.74 | 16.866 | 10.944 | 25.23 | 24.168 | 40.206 | 4.794 |
| Nutrient Broth | SYC58 | 38.946 | 53.244 | 54.75 | 41.586 | 50.772 | 58.122 | 3.102 | 17.778 | 19.638 | 32.856 | 46.248 | 12.426 |
| Nutrient Broth | J90 | 33.63 | 54.93 | 55.476 | 35.322 | 36.732 | 51.492 | 15.528 | 29.544 | 29.358 | 28.662 | 46.218 | 13.92 |
| Nutrient Broth | SYC60 | 41.214 | 60.666 | 52.368 | 35.046 | 45.324 | 66.108 | 1.764 | 18.312 | 21.762 | 37.092 | 51.42 | 12.606 |
| Nutrient Broth | SS61 | 52.35 | 46.224 | 45.39 | 29.334 | 34.926 | 64.14 | 0.474 | 27.864 | 34.734 | 47.406 | 48.564 | 17.856 |
| Nutrient Broth | SYC63 | 43.278 | 52.59 | 49.44 | 38.91 | 64.104 | 65.79 | 1.068 | 18.318 | 22.56 | 33.552 | 49.716 | 12.426 |
| Nutrient Broth | SYC64 | 47.46 | 55.512 | 51.318 | 46.308 | 55.452 | 64.212 | 1.128 | 17.208 | 20.346 | 34.038 | 50.406 | 11.442 |
| Nutrient Broth | SS59 | 38.868 | 63.768 | 52.86 | 47.952 | 49.716 | 56.136 | 3.138 | 17.958 | 28.404 | 39.252 | 52.998 | 13.182 |
| Nutrient Broth | SYC55 | 55.956 | 54.576 | 67.266 | 33.198 | 38.028 | 73.038 | 4.548 | 18.792 | 27 | 46.122 | 51.378 | 12.456 |
| Nutrient Broth | J82 | 48.858 | 60.066 | 55.578 | 57.576 | 61.716 | 61.554 | -1.746 | 16.794 | 26.502 | 36.126 | 50.178 | 11.676 |
| Nutrient Broth | SS64 | 52.764 | 66.108 | 60.438 | 67.548 | 45.246 | 54.3 | 5.682 | 11.49 | 25.758 | 33.012 | 55.554 | 9.432 |
| Nutrient Broth | J87 | 53.28 | 62.67 | 60.642 | 50.49 | 44.136 | 64.356 | 2.736 | 19.254 | 26.088 | 39.894 | 56.082 | 11.436 |
| Nutrient Broth | J3 | 57.156 | 70.278 | 63.216 | 54.816 | 58.35 | 47.322 | 1.98 | 8.628 | 34.38 | 31.866 | 61.746 | 3.648 |
| Nutrient Broth | J85 | 44.106 | 55.794 | 58.722 | 58.824 | 56.49 | 63.018 | 2.802 | 21.654 | 28.536 | 41.34 | 55.05 | 12.72 |
| Nutrient Broth | SYC59 | 33.4640485 | 60.3481755 | 52.9164475 | 39.288447 | 39.562815 | 70.055651 | 4.8917275 | 23.9896415 | 28.12154 | 50.8025135 | 77.0638015 | 18.911689 |
| Nutrient Broth | SYC57 | 44.088 | 58.974 | 51.492 | 55.992 | 58.956 | 62.124 | 2.322 | 22.716 | 33.69 | 40.062 | 58.848 | 18.27 |
| Nutrient Broth | J5 | 71.316 | 69.522 | 66.912 | 62.502 | 67.32 | 32.274 | -1.128 | 4.68 | 39.102 | 39.138 | 62.286 | 5.502 |
| Nutrient Broth | SS55 | 57.69 | 68.196 | 63.828 | 39.936 | 47.106 | 68.496 | 5.658 | 27.678 | 30.528 | 52.104 | 53.562 | 16.566 |
| Nutrient Broth | J89 | 64.206 | 61.986 | 68.886 | 62.742 | 60.09 | 39.978 | 17.388 | 19.872 | 37.038 | 34.86 | 54.534 | 9.888 |
| Nutrient Broth | J83 | 56.514 | 76.086 | 67.23 | 66.636 | 64.488 | 59.268 | 4.584 | 13.026 | 30.342 | 37.908 | 57 | 10.8 |
| Nutrient Broth | J10 | 70.23 | 61.494 | 70.452 | 80.028 | 63.822 | 46.098 | -0.666 | 3.654 | 36.09 | 45.9 | 66.48 | 2.58 |
| Nutrient Broth | J1 | 89.838 | 58.11 | 61.92 | 61.026 | 57.216 | 48.63 | 2.382 | 8.106 | 40.158 | 39.156 | 72.384 | 7.788 |
| Nutrient Broth | J81 | 74.124 | 70.86 | 71.982 | 70.872 | 65.058 | 55.698 | -2.946 | 7.8 | 31.47 | 40.608 | 56.952 | 6.78 |
| Nutrient Broth | J12 | 72.642 | 66.6 | 72.498 | 79.698 | 77.832 | 39.912 | 2.856 | 7.674 | 40.476 | 43.368 | 69.006 | 6.57 |
| Nutrient Broth | J6 | 94.086 | 55.524 | 64.5 | 63.264 | 74.448 | 52.854 | -1.974 | 4.506 | 47.496 | 46.77 | 77.856 | 5.52 |
| Nutrient Broth | J84 | 59.694 | 74.88 | 64.59 | 69.204 | 74.292 | 65.724 | 3.078 | 20.142 | 33.15 | 46.338 | 59.034 | 14.724 |
| Nutrient Broth | J4 | 93.894 | 58.824 | 68.322 | 65.934 | 70.626 | 51.81 | 0.696 | 5.664 | 44.286 | 43.866 | 80.37 | 4.338 |
| Nutrient Broth | J13 | 83.67 | 72.126 | 81.636 | 73.722 | 73.122 | 45.348 | 1.5 | 8.796 | 37.536 | 48.24 | 66.96 | 8.244 |
| Nutrient Broth | J2 | 104.502 | 63.108 | 86.052 | 68.322 | 68.628 | 46.758 | 1.968 | 7.56 | 45.618 | 42.24 | 73.41 | 7.872 |
| Nutrient Broth | J94 | 77.226 | 73.932 | 86.904 | 78.81 | 71.118 | 59.952 | 8.352 | 11.874 | 36.882 | 47.214 | 68.298 | 7.404 |
| Nutrient Broth | J93 | 80.85 | 79.59 | 81.27 | 74.82 | 69.912 | 60.108 | 4.95 | 13.926 | 39.87 | 48.54 | 72.528 | 10.83 |
| Nutrient Broth | J96 | 86.862 | 86.088 | 92.52 | 75.282 | 77.43 | 44.202 | 3.642 | 8.286 | 37.776 | 49.734 | 68.004 | 8.04 |
| Nutrient Broth | J14 | 86.868 | 69.102 | 77.1 | 76.218 | 93.498 | 43.896 | 3.438 | 9.546 | 49.188 | 47.388 | 78.306 | 9 |
| Nutrient Broth | J88 | 88.896 | 78.99 | 87.498 | 81.144 | 80.898 | 51.576 | 2.586 | 13.02 | 39.486 | 47.916 | 68.964 | 9.822 |
| Nutrient Broth | J7 | 72.666 | 69.048 | 67.956 | 64.236 | 62.676 | 88.734 | 3.078 | 43.962 | 52.212 | 56.166 | 78.036 | 6.852 |
| Nutrient Broth | J95 | 77.988 | 87.084 | 90.078 | 75.51 | 76.002 | 63.894 | 3.444 | 14.64 | 41.55 | 52.182 | 72.402 | 11.136 |
| Nutrient Broth | J86 | 88.032 | 81.942 | 92.7 | 88.782 | 83.49 | 57.708 | 2.46 | 7.098 | 40.092 | 50.748 | 72.57 | 7.476 |
| Nutrient Broth | J15 | 93.786 | 81.324 | 87.378 | 79.554 | 77.166 | 61.92 | 3.012 | 9.492 | 50.736 | 58.944 | 89.136 | 8.13 |
| Nutrient Broth | J92 | 96.378 | 86.514 | 91.338 | 76.23 | 78 | 66.042 | 5.016 | 22.398 | 40.422 | 54.132 | 72.558 | 15.018 |
| Nutrient Broth | J91 | 83.46 | 80.442 | 79.278 | 74.706 | 65.916 | 85.26 | 3.408 | 39.738 | 53.076 | 63.438 | 83.364 | 25.116 |
| Tryptophan | SS86 | -0.066 | 0.108 | 55.308 | 0.426 | 0.312 | 34.074 | 0.372 | 4.236 | 7.314 | 30.192 | 25.986 | 2.094 |
| Tryptophan | J71 | 33.012 | 24.606 | 20.088 | 18.534 | 16.062 | 35.622 | -11.184 | -2.55 | 13.788 | 16.644 | 25.428 | -1.896 |
| Tryptophan | J70 | 38.778 | 40.224 | 41.778 | 57.3 | 41.214 | 50.016 | 1.698 | 10.536 | 14.166 | 23.292 | 45.192 | 5.862 |
| Tryptophan | J69 | 40.656 | 41.262 | 32.688 | 23.79 | 39.546 | 53.028 | 2.148 | 22.194 | 30.312 | 37.29 | 40.338 | 11.49 |
| Tryptophan | J72 | 52.584 | 46.524 | 46.944 | 23.988 | 31.434 | 54.594 | -5.382 | 21.858 | 28.332 | 44.934 | 31.764 | 12.99 |
| Tryptophan | SYC78 | 36.336 | 50.886 | 47.274 | 23.862 | 38.316 | 64.128 | 3.252 | 24.276 | 27.276 | 38.274 | 46.434 | 15.162 |
| Tryptophan | J79 | 54.936 | 61.884 | 48.918 | 30.672 | 40.776 | 49.482 | 5.316 | 22.2 | 26.1 | 26.898 | 37.092 | 17.22 |
| Tryptophan | J80 | 67.176 | 49.41 | 51.552 | 30.408 | 36.252 | 47.376 | 4.65 | 17.262 | 26.97 | 47.508 | 38.37 | 7.644 |
| Tryptophan | SYC77 | 51.588 | 55.122 | 57.63 | 42.768 | 46.074 | 51.204 | 2.85 | 24.024 | 22.092 | 34.992 | 40.218 | 13.914 |
| Tryptophan | J74 | 46.296 | 41.178 | 43.662 | 39.432 | 36.984 | 72.12 | 2.538 | 28.752 | 25.404 | 41.802 | 52.626 | 17.664 |
| Tryptophan | J77 | 64.47 | 74.31 | 70.746 | 32.778 | 48.966 | 65.028 | 2.154 | 11.412 | 15.714 | 26.922 | 50.676 | 5.688 |
| Tryptophan | J76 | 45.852 | 55.62 | 49.302 | 31.158 | 38.22 | 69.684 | 1.764 | 36.942 | 32.028 | 51.498 | 45.234 | 20.244 |
| Tryptophan | SYC76 | 54.882 | 60.54 | 53.97 | 46.68 | 48.642 | 64.716 | 3.162 | 29.388 | 28.302 | 42.108 | 47.982 | 17.952 |
| Tryptophan | J68 | 62.232 | 59.22 | 57 | 38.394 | 43.998 | 62.502 | 7.35 | 30.12 | 31.506 | 50.538 | 45.798 | 18.588 |
| Tryptophan | SS77 | 46.578 | 53.088 | 46.872 | 55.566 | 50.64 | 72.114 | 14.502 | 38.466 | 29.082 | 44.676 | 60.12 | 18.846 |
| Tryptophan | J67 | 63.108 | 56.376 | 58.224 | 38.706 | 52.104 | 75.876 | 4.464 | 34.74 | 32.508 | 55.32 | 52.614 | 19.164 |
| Tryptophan | SYC79 | 59.724 | 61.962 | 59.4 | 44.79 | 49.92 | 77.442 | 3.684 | 36.324 | 31.884 | 47.85 | 51.948 | 20.1 |
| Tryptophan | J73 | 63.9 | 68.016 | 65.844 | 31.38 | 52.152 | 73.596 | 6.306 | 30.852 | 32.37 | 53.724 | 49.014 | 18.426 |
| Tryptophan | SYC82 | 59.706 | 60.78 | 54.558 | 47.28 | 49.554 | 77.07 | 0.672 | 33.672 | 34.29 | 53.994 | 56.19 | 20.49 |
| Tryptophan | SYC75 | 63.432 | 59.946 | 58.248 | 49.848 | 74.946 | 69.258 | 3.48 | 30.078 | 35.574 | 45.156 | 52.692 | 17.262 |
| Tryptophan | SS83 | 44.808 | 53.28 | 66.828 | 37.176 | 33.102 | 90.816 | 2.088 | 53.058 | 35.472 | 59.652 | 59.784 | 32.052 |
| Tryptophan | SYC81 | 62.076 | 60.708 | 59.886 | 44.856 | 64.074 | 75.036 | 1.23 | 33.834 | 34.944 | 52.608 | 60.576 | 21.168 |
| Tryptophan | SYC83 | 63.534 | 55.776 | 59.052 | 43.026 | 67.386 | 82.23 | 2.886 | 37.848 | 37.17 | 49.914 | 58.932 | 21.936 |
| Tryptophan | SS80 | 22.1 | 64 | 68.232 | 60.978 | 65.088 | 87.348 | 9.942 | 39.312 | 40.668 | 58.752 | 61.48 | 23.142 |
| Tryptophan | SYC84 | 68.724 | 68.064 | 62.346 | 46.452 | 69.21 | 86.208 | 1.83 | 38.088 | 38.946 | 52.152 | 61.494 | 22.386 |
| Tryptophan | SS85 | 62.43 | 75.198 | 68.844 | 50.826 | 60.312 | 78.654 | 7.878 | 39.258 | 36.456 | 58.302 | 56.34 | 23.412 |
| Tryptophan | SS81 | 58.218 | 76.212 | 74.016 | 50.586 | 60.348 | 82.536 | 7.458 | 38.802 | 33.762 | 56.76 | 57.936 | 22.998 |
| Tryptophan | SS84 | 75.39 | 75.576 | 45.144 | 50.586 | 54.918 | 89.088 | 7.398 | 43.596 | 38.034 | 54.876 | 61.23 | 25.854 |
| Tryptophan | J75 | 66.306 | 63.912 | 58.374 | 59.298 | 57.216 | 84.474 | 10.914 | 40.698 | 36.81 | 59.31 | 62.64 | 25.248 |
| Tryptophan | SS76 | 73.11 | 74.862 | 71.112 | 48.924 | 57.636 | 76.788 | 11.904 | 40.026 | 36.924 | 55.74 | 56.214 | 22.344 |
| Tryptophan | SS82 | 69.222 | 75.684 | 74.712 | 51.498 | 59.25 | 83.082 | 10.2 | 37.146 | 35.934 | 57.3 | 59.358 | 21.924 |
| Tryptophan | SYC80 | 77.082 | 74.886 | 66.282 | 58.692 | 57.6 | 89.19 | 1.734 | 36.744 | 38.514 | 62.418 | 65.034 | 23.976 |
| Tryptophan | SS79 | 54.26 | 65.4 | 72.702 | 52.77 | 65.628 | 89.514 | 20.004 | 52.278 | 45.75 | 64.35 | 61.7 | 27.51 |
| Tryptophan | J78 | 73.314202 | 65.395518 | 63.531491 | 50.798393 | 87.885382 | 76.8967045 | 8.149074 | 35.730488 | 44.361739 | 66.9588185 | 75.7369955 | 26.8502065 |
| Tryptophan | SS78 | 73.77 | 75.516 | 75.096 | 51.516 | 60.054 | 78.288 | 32.292 | 51.588 | 35.154 | 59.304 | 66.426 | 27.498 |
| Urea | SS9 | 47.916 | 24.312 | 25.422 | 41.706 | 35.136 | -6.468 | -4.89 | -5.85 | 8.874 | 4.776 | 20.748 | -7.89 |
| Urea | SS10 | 49.032 | 29.16 | 27.336 | 23.028 | 21.072 | 35.7 | 4.938 | 1.974 | 19.35 | 25.506 | 41.052 | -5.61 |
| Urea | SS4 | 58.644 | 25.974 | 29.964 | 41.724 | 27.186 | 48.378 | 2.502 | 16.692 | 21.51 | 29.934 | 48.348 | 9.888 |
| Urea | SYC19 | 40.986 | 40.296 | 39.27 | 26.712 | 26.88 | 65.208 | 3 | 16.404 | 25.344 | 39.288 | 48.384 | 10.218 |
| Urea | SYC20 | 30.33 | 30.156 | 27.93 | 34.86 | 33.63 | 68.814 | 1.752 | 32.592 | 28.824 | 36.678 | 55.416 | 17.526 |
| Urea | SYC18 | 58.338 | 60.9 | 40.854 | 50.85 | 41.004 | 55.41 | 2.712 | 9.828 | 24.456 | 38.886 | 48.432 | 6.864 |
| Urea | J35 | 52.092 | 35.712 | 36 | 32.778 | 37.044 | 64.362 | 3.132 | 38.214 | 31.788 | 40.932 | 50.682 | 20.514 |
| Urea | SS7 | 45.48 | 50.214 | 37.302 | 24.642 | 38.934 | 68.766 | 15.33 | 31.464 | 26.646 | 44.244 | 46.56 | 17.088 |
| Urea | SS3 | 49.218 | 74.082 | 58.38 | 40.326 | 35.01 | 62.358 | 7.572 | 13.644 | 26.928 | 35.22 | 50.448 | 9.204 |
| Urea | J34 | 31.284 | 30.642 | 31.71 | 48.954 | 30.888 | 86.262 | 2.796 | 44.604 | 33.174 | 47.214 | 60.582 | 21.678 |
| Urea | SYC15 | 47.76 | 56.166 | 59.838 | 45.78 | 38.106 | 55.968 | 6.936 | 17.244 | 34.404 | 43.518 | 54.18 | 12.54 |
| Urea | J33 | 50.328 | 57.12 | 60.108 | 45.066 | 40.26 | 55.074 | 8.778 | 22.356 | 30.852 | 48.774 | 47.364 | 16.656 |
| Urea | SS8 | 50.76 | 48.648 | 50.616 | 39.66 | 31.878 | 68.334 | 16.554 | 28.368 | 30.606 | 47.808 | 52.788 | 17.634 |
| Urea | SYC17 | 47.502 | 55.872 | 49.308 | 41.934 | 35.826 | 73.404 | 5.202 | 23.328 | 34.746 | 47.208 | 56.07 | 15.648 |
| Urea | SS5 | 76.614 | 87.876 | 86.736 | 61.602 | 58.044 | 17.328 | 3.684 | 7.176 | 25.764 | 34.752 | 35.724 | 6.888 |
| Urea | SYC11 | 53.634 | 62.292 | 59.346 | 54.042 | 40.452 | 63.306 | 12.96 | 26.154 | 27.864 | 45.372 | 50.088 | 19.956 |
| Urea | J37 | 71.526 | 61.068 | 60.492 | 49.956 | 31.848 | 78.006 | 3.252 | 19.272 | 35.124 | 53.448 | 59.622 | 12.594 |
| Urea | SYC16 | 69.042 | 71.1 | 68.856 | 55.662 | 40.476 | 65.016 | 8.652 | 23.394 | 30.066 | 51.648 | 53.652 | 19.746 |
| Urea | SYC12 | 55.23 | 63.162 | 62.268 | 61.194 | 63.396 | 64.488 | 5.28 | 16.662 | 36.864 | 51.612 | 62.244 | 14.97 |
| Urea | SS6 | 69.834 | 76.584 | 77.718 | 61.938 | 60.558 | 46.284 | 12.276 | 18.468 | 32.604 | 48.78 | 50.052 | 14.154 |
| Urea | SYC14 | 65.31 | 71.268 | 72.144 | 46.044 | 63.396 | 69.954 | 6.096 | 16.11 | 36.666 | 52.434 | 59.43 | 10.878 |
| Urea | J39 | 50.904 | 70.098 | 78.528 | 74.352 | 46.674 | 38.826 | 19.95 | 23.46 | 46.35 | 51.57 | 61.512 | 18.468 |
| Urea | SS1 | 68.424 | 84.714 | 68.628 | 52.14 | 65.166 | 62.928 | 19.08 | 31.674 | 38.76 | 55.902 | 54.96 | 19.26 |
| Urea | J38 | 71.358 | 71.742 | 70.926 | 39.906 | 42.726 | 78.57 | 26.862 | 39.882 | 37.656 | 60.912 | 55.818 | 28.578 |
| Urea | J40 | 62.664 | 70.914 | 69.456 | 75.522 | 72.42 | 89.658 | 3.354 | 14.862 | 43.596 | 59.424 | 82.644 | 10.542 |
| Urea | SYC13 | 64.782 | 70.182 | 72.42 | 74.076 | 73.974 | 91.008 | 5.352 | 36.156 | 47.034 | 60.744 | 75.408 | 23.304 |
| Urea | J36 | 89.73 | 74.994 | 93.57 | 89.73 | 94.578 | 51.9 | 9.024 | 13.002 | 52.056 | 53.622 | 78.69 | 13.71 |
| Urea | SS2 | 93.222 | 108.66 | 96.99 | 98.964 | 89.154 | 56.742 | 18.642 | 18.342 | 45.714 | 50.286 | 76.692 | 10.692 |
