# Supplementary material for: Initial nitrogen enrichment conditions determines variations in nitrogen substrate utilization by heterotrophic bacterial isolates
Source: BMC Microbiol. 2017 Apr 4;17:87. doi: 10.1186/s12866-017-0993-7 (PMC5381026; doi:10.1186/s12866-017-0993-7)
Supplement: Supplementary file 4 — Title: Score differences (total isolate score – mean) and substrate range classification of bacterial isolates from the initial N-enrichments across substrates. Description of data: The score difference between mean scaled growth rates and total scaled growth rates for each isolate on the 12 N-substrates. Positive score differences represent isolates with broad substrate range and negative score differences represent isolates with narrow substrate range. (DOCX 28 kb) [file 12866_2017_993_MOESM4_ESM.docx]

**Table S3.** Score differences (total isolate score – mean) and substrate range classification of bacterial isolates from the initial N-enrichments across substrates.

| **N-enrichment type** | **Isolates** | **Total sum of rank** | **Mean** | **Difference from mean** | **Substrate range** |
| --- | --- | --- | --- | --- | --- |
| Ammonium | J41 | 14 | 14.41 | -0.41 | narrow |
| Ammonium | J42 | 13 | 14.41 | -1.41 | narrow |
| Ammonium | J43 | 16 | 14.41 | 1.59 | broad |
| Ammonium | J44 | 8 | 14.41 | -6.41 | narrow |
| Ammonium | J45 | 12 | 14.41 | -2.41 | narrow |
| Ammonium | J46 | 20 | 14.41 | 5.59 | broad |
| Ammonium | J47 | 1 | 14.41 | -13.41 | narrow |
| Ammonium | J48 | 13 | 14.41 | -1.41 | narrow |
| Ammonium | J49 | 13 | 14.41 | -1.41 | narrow |
| Ammonium | J50 | 15 | 14.41 | 0.59 | broad |
| Ammonium | J51 | 12 | 14.41 | -2.41 | narrow |
| Ammonium | SS65 | 9 | 14.41 | -5.41 | narrow |
| Ammonium | SS66 | 16 | 14.41 | 1.59 | broad |
| Ammonium | SS67 | 13 | 14.41 | -1.41 | narrow |
| Ammonium | SS68 | 9 | 14.41 | -5.41 | narrow |
| Ammonium | SS69 | 17 | 14.41 | 2.59 | broad |
| Ammonium | SS70 | 19 | 14.41 | 4.59 | broad |
| Ammonium | SS71 | 17 | 14.41 | 2.59 | broad |
| Ammonium | SS72 | 15 | 14.41 | 0.59 | broad |
| Ammonium | SS73 | 17 | 14.41 | 2.59 | broad |
| Ammonium | SS74 | 16 | 14.41 | 1.59 | broad |
| Ammonium | SS75 | 17 | 14.41 | 2.59 | broad |
| Ammonium | SYC31 | 17 | 14.41 | 2.59 | broad |
| Ammonium | SYC32 | 18 | 14.41 | 3.59 | broad |
| Ammonium | SYC33 | 18 | 14.41 | 3.59 | broad |
| Ammonium | SYC34 | 18 | 14.41 | 3.59 | broad |
| Ammonium | SYC35 | 16 | 14.41 | 1.59 | broad |
| Ammonium | SYC36 | 18 | 14.41 | 3.59 | broad |
| Ammonium | SYC37 | 16 | 14.41 | 1.59 | broad |
| Ammonium | SYC38 | 16 | 14.41 | 1.59 | broad |
| Ammonium | SYC39 | 17 | 14.41 | 2.59 | broad |
| Ammonium | SYC40 | 15 | 14.41 | 0.59 | broad |
| Bacterial Protein | J61 | 3 | 14.41 | -11.41 | narrow |
| Bacterial Protein | J62 | 4 | 14.41 | -10.41 | narrow |
| Bacterial Protein | J63 | 3 | 14.41 | -11.41 | narrow |
| Bacterial Protein | J64 | 9 | 14.41 | -5.41 | narrow |
| Bacterial Protein | J65 | 11 | 14.41 | -3.41 | narrow |
| Bacterial Protein | J66 | 4 | 14.41 | -10.41 | narrow |
| Bacterial Protein | SS11 | 8 | 14.41 | -6.41 | narrow |
| Bacterial Protein | SS12 | 14 | 14.41 | -0.41 | narrow |
| Bacterial Protein | SS13 | 11 | 14.41 | -3.41 | narrow |
| Bacterial Protein | SS14 | 12 | 14.41 | -2.41 | narrow |
| Bacterial Protein | SS15 | 11 | 14.41 | -3.41 | narrow |
| Bacterial Protein | SS16 | 12 | 14.41 | -2.41 | narrow |
| Bacterial Protein | SS17 | 11 | 14.41 | -3.41 | narrow |
| Bacterial Protein | SS18 | 13 | 14.41 | -1.41 | narrow |
| Bacterial Protein | SS19 | 15 | 14.41 | 0.59 | broad |
| Bacterial Protein | SS20 | 14 | 14.41 | -0.41 | narrow |
| Bacterial Protein | SYC1 | 15 | 14.41 | 0.59 | broad |
| Bacterial Protein | SYC10 | 11 | 14.41 | -3.41 | narrow |
| Bacterial Protein | SYC2 | 14 | 14.41 | -0.41 | narrow |
| Bacterial Protein | SYC3 | 15 | 14.41 | 0.59 | broad |
| Bacterial Protein | SYC4 | 15 | 14.41 | 0.59 | broad |
| Bacterial Protein | SYC5 | 14 | 14.41 | -0.41 | narrow |
| Bacterial Protein | SYC6 | 14 | 14.41 | -0.41 | narrow |
| Bacterial Protein | SYC7 | 15 | 14.41 | 0.59 | broad |
| Bacterial Protein | SYC8 | 15 | 14.41 | 0.59 | broad |
| Bacterial Protein | SYC9 | 11 | 14.41 | -3.41 | narrow |
| Defined-N-mixture | J52 | 21 | 14.41 | 6.59 | broad |
| Defined-N-mixture | J53 | 17 | 14.41 | 2.59 | broad |
| Defined-N-mixture | J54 | 18 | 14.41 | 3.59 | broad |
| Defined-N-mixture | J55 | 15 | 14.41 | 0.59 | broad |
| Defined-N-mixture | J56 | 10 | 14.41 | -4.41 | narrow |
| Defined-N-mixture | J57 | 17 | 14.41 | 2.59 | broad |
| Defined-N-mixture | J58 | 16 | 14.41 | 1.59 | broad |
| Defined-N-mixture | J59 | 12 | 14.41 | -2.41 | narrow |
| Defined-N-mixture | J60 | 18 | 14.41 | 3.59 | broad |
| Defined-N-mixture | SS21 | 7 | 14.41 | -7.41 | narrow |
| Defined-N-mixture | SS22 | 16 | 14.41 | 1.59 | broad |
| Defined-N-mixture | SS23 | 13 | 14.41 | -1.41 | narrow |
| Defined-N-mixture | SS24 | 15 | 14.41 | 0.59 | broad |
| Defined-N-mixture | SS25 | 16 | 14.41 | 1.59 | broad |
| Defined-N-mixture | SS26 | 17 | 14.41 | 2.59 | broad |
| Defined-N-mixture | SS27 | 16 | 14.41 | 1.59 | broad |
| Defined-N-mixture | SS28 | 16 | 14.41 | 1.59 | broad |
| Defined-N-mixture | SS29 | 20 | 14.41 | 5.59 | broad |
| Defined-N-mixture | SS30 | 16 | 14.41 | 1.59 | broad |
| Defined-N-mixture | SYC41 | 17 | 14.41 | 2.59 | broad |
| Defined-N-mixture | SYC42 | 15 | 14.41 | 0.59 | broad |
| Defined-N-mixture | SYC43 | 17 | 14.41 | 2.59 | broad |
| Defined-N-mixture | SYC44 | 17 | 14.41 | 2.59 | broad |
| Defined-N-mixture | SYC45 | 17 | 14.41 | 2.59 | broad |
| Defined-N-mixture | SYC46 | 17 | 14.41 | 2.59 | broad |
| Defined-N-mixture | SYC47 | 15 | 14.41 | 0.59 | broad |
| Defined-N-mixture | SYC48 | 15 | 14.41 | 0.59 | broad |
| Defined-N-mixture | SYC49 | 16 | 14.41 | 1.59 | broad |
| Defined-N-mixture | SYC50 | 17 | 14.41 | 2.59 | broad |
| Defined-N-mixture | SYC51 | 15 | 14.41 | 0.59 | broad |
| Defined-N-mixture | SYC52 | 19 | 14.41 | 4.59 | broad |
| Glycine | J16 | 18 | 14.41 | 3.59 | broad |
| Glycine | J17 | 12 | 14.41 | -2.41 | narrow |
| Glycine | J18 | 18 | 14.41 | 3.59 | broad |
| Glycine | J19 | 15 | 14.41 | 0.59 | broad |
| Glycine | J20 | 17 | 14.41 | 2.59 | broad |
| Glycine | J21 | 17 | 14.41 | 2.59 | broad |
| Glycine | J22 | 12 | 14.41 | -2.41 | narrow |
| Glycine | J23 | 11 | 14.41 | -3.41 | narrow |
| Glycine | J24 | 14 | 14.41 | -0.41 | narrow |
| Glycine | SS41 | 15 | 14.41 | 0.59 | broad |
| Glycine | SS42 | 13 | 14.41 | -1.41 | narrow |
| Glycine | SS43 | 19 | 14.41 | 4.59 | broad |
| Glycine | SS44 | 13 | 14.41 | -1.41 | narrow |
| Glycine | SS45 | 14 | 14.41 | -0.41 | narrow |
| Glycine | SS46 | 19 | 14.41 | 4.59 | broad |
| Glycine | SS47 | 14 | 14.41 | -0.41 | narrow |
| Glycine | SS48 | 18 | 14.41 | 3.59 | broad |
| Glycine | SS49 | 11 | 14.41 | -3.41 | narrow |
| Glycine | SS50 | 17 | 14.41 | 2.59 | broad |
| Glycine | SYC65 | 17 | 14.41 | 2.59 | broad |
| Glycine | SYC66 | 17 | 14.41 | 2.59 | broad |
| Glycine | SYC67 | 16 | 14.41 | 1.59 | broad |
| Glycine | SYC68 | 16 | 14.41 | 1.59 | broad |
| Glycine | SYC69 | 15 | 14.41 | 0.59 | broad |
| Glycine | SYC70 | 16 | 14.41 | 1.59 | broad |
| Glycine | SYC71 | 17 | 14.41 | 2.59 | broad |
| Glycine | SYC72 | 17 | 14.41 | 2.59 | broad |
| Glycine | SYC73 | 15 | 14.41 | 0.59 | broad |
| Glycine | SYC74 | 16 | 14.41 | 1.59 | broad |
| Nitrate | J25 | 8 | 14.41 | -6.41 | narrow |
| Nitrate | J26 | 13 | 14.41 | -1.41 | narrow |
| Nitrate | J27 | 12 | 14.41 | -2.41 | narrow |
| Nitrate | J28 | 5 | 14.41 | -9.41 | narrow |
| Nitrate | J29 | 17 | 14.41 | 2.59 | broad |
| Nitrate | J30 | 11 | 14.41 | -3.41 | narrow |
| Nitrate | J31 | 13 | 14.41 | -1.41 | narrow |
| Nitrate | J32 | 11 | 14.41 | -3.41 | narrow |
| Nitrate | SS31 | 13 | 14.41 | -1.41 | narrow |
| Nitrate | SS32 | 14 | 14.41 | -0.41 | narrow |
| Nitrate | SS33 | 13 | 14.41 | -1.41 | narrow |
| Nitrate | SS34 | 17 | 14.41 | 2.59 | broad |
| Nitrate | SS35 | 17 | 14.41 | 2.59 | broad |
| Nitrate | SS36 | 17 | 14.41 | 2.59 | broad |
| Nitrate | SS37 | 12 | 14.41 | -2.41 | narrow |
| Nitrate | SS38 | 17 | 14.41 | 2.59 | broad |
| Nitrate | SS39 | 9 | 14.41 | -5.41 | narrow |
| Nitrate | SS40 | 14 | 14.41 | -0.41 | narrow |
| Nitrate | SYC21 | 15 | 14.41 | 0.59 | broad |
| Nitrate | SYC22 | 16 | 14.41 | 1.59 | broad |
| Nitrate | SYC23 | 16 | 14.41 | 1.59 | broad |
| Nitrate | SYC24 | 16 | 14.41 | 1.59 | broad |
| Nitrate | SYC25 | 14 | 14.41 | -0.41 | narrow |
| Nitrate | SYC26 | 16 | 14.41 | 1.59 | broad |
| Nitrate | SYC27 | 15 | 14.41 | 0.59 | broad |
| Nitrate | SYC28 | 16 | 14.41 | 1.59 | broad |
| Nitrate | SYC29 | 16 | 14.41 | 1.59 | broad |
| Nitrate | SYC30 | 16 | 14.41 | 1.59 | broad |
| Nutrient Broth | J1 | 16 | 14.41 | 1.59 | broad |
| Nutrient Broth | J10 | 16 | 14.41 | 1.59 | broad |
| Nutrient Broth | J11 | 14 | 14.41 | -0.41 | narrow |
| Nutrient Broth | J12 | 16 | 14.41 | 1.59 | broad |
| Nutrient Broth | J13 | 17 | 14.41 | 2.59 | broad |
| Nutrient Broth | J14 | 18 | 14.41 | 3.59 | broad |
| Nutrient Broth | J15 | 18 | 14.41 | 3.59 | broad |
| Nutrient Broth | J2 | 19 | 14.41 | 4.59 | broad |
| Nutrient Broth | J3 | 16 | 14.41 | 1.59 | broad |
| Nutrient Broth | J4 | 18 | 14.41 | 3.59 | broad |
| Nutrient Broth | J5 | 12 | 14.41 | -2.41 | narrow |
| Nutrient Broth | J6 | 17 | 14.41 | 2.59 | broad |
| Nutrient Broth | J7 | 20 | 14.41 | 5.59 | broad |
| Nutrient Broth | J8 | 14 | 14.41 | -0.41 | narrow |
| Nutrient Broth | J81 | 16 | 14.41 | 1.59 | broad |
| Nutrient Broth | J82 | 14 | 14.41 | -0.41 | narrow |
| Nutrient Broth | J83 | 16 | 14.41 | 1.59 | broad |
| Nutrient Broth | J84 | 17 | 14.41 | 2.59 | broad |
| Nutrient Broth | J85 | 16 | 14.41 | 1.59 | broad |
| Nutrient Broth | J86 | 18 | 14.41 | 3.59 | broad |
| Nutrient Broth | J87 | 14 | 14.41 | -0.41 | narrow |
| Nutrient Broth | J88 | 16 | 14.41 | 1.59 | broad |
| Nutrient Broth | J89 | 14 | 14.41 | -0.41 | narrow |
| Nutrient Broth | J9 | 5 | 14.41 | -9.41 | narrow |
| Nutrient Broth | J90 | 11 | 14.41 | -3.41 | narrow |
| Nutrient Broth | J91 | 18 | 14.41 | 3.59 | broad |
| Nutrient Broth | J92 | 18 | 14.41 | 3.59 | broad |
| Nutrient Broth | J93 | 16 | 14.41 | 1.59 | broad |
| Nutrient Broth | J94 | 17 | 14.41 | 2.59 | broad |
| Nutrient Broth | J95 | 18 | 14.41 | 3.59 | broad |
| Nutrient Broth | J96 | 17 | 14.41 | 2.59 | broad |
| Nutrient Broth | SS51 | 5 | 14.41 | -9.41 | narrow |
| Nutrient Broth | SS52 | 1 | 14.41 | -13.41 | narrow |
| Nutrient Broth | SS53 | 3 | 14.41 | -11.41 | narrow |
| Nutrient Broth | SS54 | 9 | 14.41 | -5.41 | narrow |
| Nutrient Broth | SS55 | 15 | 14.41 | 0.59 | broad |
| Nutrient Broth | SS56 | 14 | 14.41 | -0.41 | narrow |
| Nutrient Broth | SS57 | 10 | 14.41 | -4.41 | narrow |
| Nutrient Broth | SS58 | 9 | 14.41 | -5.41 | narrow |
| Nutrient Broth | SS59 | 13 | 14.41 | -1.41 | narrow |
| Nutrient Broth | SS60 | 12 | 14.41 | -2.41 | narrow |
| Nutrient Broth | SS61 | 14 | 14.41 | -0.41 | narrow |
| Nutrient Broth | SS62 | 14 | 14.41 | -0.41 | narrow |
| Nutrient Broth | SS63 | 13 | 14.41 | -1.41 | narrow |
| Nutrient Broth | SS64 | 15 | 14.41 | 0.59 | broad |
| Nutrient Broth | SYC53 | 10 | 14.41 | -4.41 | narrow |
| Nutrient Broth | SYC54 | 11 | 14.41 | -3.41 | narrow |
| Nutrient Broth | SYC55 | 14 | 14.41 | -0.41 | narrow |
| Nutrient Broth | SYC56 | 11 | 14.41 | -3.41 | narrow |
| Nutrient Broth | SYC57 | 17 | 14.41 | 2.59 | broad |
| Nutrient Broth | SYC58 | 14 | 14.41 | -0.41 | narrow |
| Nutrient Broth | SYC59 | 11 | 14.41 | -3.41 | narrow |
| Nutrient Broth | SYC60 | 14 | 14.41 | -0.41 | narrow |
| Nutrient Broth | SYC61 | 12 | 14.41 | -2.41 | narrow |
| Nutrient Broth | SYC62 | 12 | 14.41 | -2.41 | narrow |
| Nutrient Broth | SYC63 | 14 | 14.41 | -0.41 | narrow |
| Nutrient Broth | SYC64 | 15 | 14.41 | 0.59 | broad |
| Tryptophan | J67 | 17 | 14.41 | 2.59 | broad |
| Tryptophan | J68 | 17 | 14.41 | 2.59 | broad |
| Tryptophan | J69 | 11 | 14.41 | -3.41 | narrow |
| Tryptophan | J70 | 13 | 14.41 | -1.41 | narrow |
| Tryptophan | J71 | -1 | 14.41 | -15.41 | narrow |
| Tryptophan | J72 | 11 | 14.41 | -3.41 | narrow |
| Tryptophan | J73 | 17 | 14.41 | 2.59 | broad |
| Tryptophan | J74 | 13 | 14.41 | -1.41 | narrow |
| Tryptophan | J75 | 19 | 14.41 | 4.59 | broad |
| Tryptophan | J76 | 16 | 14.41 | 1.59 | broad |
| Tryptophan | J77 | 13 | 14.41 | -1.41 | narrow |
| Tryptophan | J78 | 19 | 14.41 | 4.59 | broad |
| Tryptophan | J79 | 12 | 14.41 | -2.41 | narrow |
| Tryptophan | J80 | 13 | 14.41 | -1.41 | narrow |
| Tryptophan | SS76 | 19 | 14.41 | 4.59 | broad |
| Tryptophan | SS77 | 17 | 14.41 | 2.59 | broad |
| Tryptophan | SS78 | 20 | 14.41 | 5.59 | broad |
| Tryptophan | SS79 | 20 | 14.41 | 5.59 | broad |
| Tryptophan | SS80 | 16 | 14.41 | 1.59 | broad |
| Tryptophan | SS81 | 18 | 14.41 | 3.59 | broad |
| Tryptophan | SS82 | 18 | 14.41 | 3.59 | broad |
| Tryptophan | SS83 | 18 | 14.41 | 3.59 | broad |
| Tryptophan | SS84 | 19 | 14.41 | 4.59 | broad |
| Tryptophan | SS85 | 17 | 14.41 | 2.59 | broad |
| Tryptophan | SS86 | 3 | 14.41 | -11.41 | narrow |
| Tryptophan | SYC75 | 18 | 14.41 | 3.59 | broad |
| Tryptophan | SYC76 | 16 | 14.41 | 1.59 | broad |
| Tryptophan | SYC77 | 15 | 14.41 | 0.59 | broad |
| Tryptophan | SYC78 | 11 | 14.41 | -3.41 | narrow |
| Tryptophan | SYC79 | 18 | 14.41 | 3.59 | broad |
| Tryptophan | SYC80 | 18 | 14.41 | 3.59 | broad |
| Tryptophan | SYC81 | 18 | 14.41 | 3.59 | broad |
| Tryptophan | SYC82 | 18 | 14.41 | 3.59 | broad |
| Tryptophan | SYC83 | 18 | 14.41 | 3.59 | broad |
| Tryptophan | SYC84 | 18 | 14.41 | 3.59 | broad |
| Urea | J33 | 17 | 14.41 | 2.59 | broad |
| Urea | J34 | 15 | 14.41 | 0.59 | broad |
| Urea | J35 | 14 | 14.41 | -0.41 | narrow |
| Urea | J36 | 18 | 14.41 | 3.59 | broad |
| Urea | J37 | 16 | 14.41 | 1.59 | broad |
| Urea | J38 | 15 | 14.41 | 0.59 | broad |
| Urea | J39 | 17 | 14.41 | 2.59 | broad |
| Urea | J40 | 18 | 14.41 | 3.59 | broad |
| Urea | SS1 | 18 | 14.41 | 3.59 | broad |
| Urea | SS10 | 4 | 14.41 | -10.41 | narrow |
| Urea | SS2 | 19 | 14.41 | 4.59 | broad |
| Urea | SS3 | 14 | 14.41 | -0.41 | narrow |
| Urea | SS4 | 8 | 14.41 | -6.41 | narrow |
| Urea | SS5 | 12 | 14.41 | -2.41 | narrow |
| Urea | SS6 | 17 | 14.41 | 2.59 | broad |
| Urea | SS7 | 13 | 14.41 | -1.41 | narrow |
| Urea | SS8 | 14 | 14.41 | -0.41 | narrow |
| Urea | SS9 | 1 | 14.41 | -13.41 | narrow |
| Urea | SYC11 | 16 | 14.41 | 1.59 | broad |
| Urea | SYC12 | 17 | 14.41 | 2.59 | broad |
| Urea | SYC13 | 19 | 14.41 | 4.59 | broad |
| Urea | SYC14 | 17 | 14.41 | 2.59 | broad |
| Urea | SYC15 | 16 | 14.41 | 1.59 | broad |
| Urea | SYC16 | 17 | 14.41 | 2.59 | broad |
| Urea | SYC17 | 16 | 14.41 | 1.59 | broad |
| Urea | SYC18 | 15 | 14.41 | 0.59 | broad |
| Urea | SYC19 | 8 | 14.41 | -6.41 | narrow |
| Urea | SYC20 | 10 | 14.41 | -4.41 | narrow |
